# The Impact of GFP Reporter Gene Transduction and Expression on Metabolomics of Placental Mesenchymal Stem Cells Determined by UHPLC-Q/TOF-MS

**DOI:** 10.1155/2017/3167985

**Published:** 2017-11-05

**Authors:** Jinfeng Yang, Nan Wang, Deying Chen, Jiong Yu, Qiaoling Pan, Dan Wang, Jingqi Liu, Xiaowei Shi, Xiaotian Dong, Hongcui Cao, Liang Li, Lanjuan Li

**Affiliations:** ^1^State Key Laboratory for the Diagnosis and Treatment of Infectious Diseases, The First Affiliated Hospital, College of Medicine, Zhejiang University, Hangzhou, China; ^2^Collaborative Innovation Center for the Diagnosis and Treatment of Infectious Diseases, Zhejiang University, Hangzhou 310003, China; ^3^Department of Chemistry, University of Alberta, Edmonton, AB, Canada T6G 2G2; ^4^Chu Kochen Honors College, Zhejiang University, 866 Yuhangtang Rd., Hangzhou 310058, China

## Abstract

**Introduction:**

Green fluorescent protein (GFP) is widely used as a reporter gene in regenerative medicine research to label and track stem cells. Here, we examined whether expressing GFP gene may impact the metabolism of human placental mesenchymal stem cells (hPMSCs).

**Methods:**

The GFP gene was transduced into hPMSCs using lentiviral-based infection to establish GFP^+^hPMSCs. A sensitive ^13^C/^12^C-dansyl labeling LC-MS method targeting the amine/phenol submetabolome was used for in-depth cell metabolome profiling.

**Results:**

A total of 1151 peak pairs or metabolites were detected from 12 LC-MS runs. Principal component analysis and partial least squares discriminant analysis showed poor separation, and the volcano plots demonstrated that most of the metabolites were not significantly changed when hPMSCs were tagged with GFP. Overall, 739 metabolites were positively or putatively identified. Only 11 metabolites showed significant changes. Metabolic pathway analyses indicated that three of the identified metabolites were involved in nine pathways. However, these metabolites are unlikely to have a large impact on the metabolic pathways due to their nonessential roles and limited hits in pathway analysis.

**Conclusion:**

This study indicated that the expression of ectopic GFP reporter gene did not significantly alter the metabolomics pathways covered by the amine/phenol submetabolome.

## 1. Introduction

Mesenchymal stem cells (MSCs) are the current focus for the development of cell-based therapies for various diseases due to their regenerative and immune regulatory potential [1, 2]. For the preclinical application of MSCs, labeling and tracking are crucial for evaluating the *in vivo* distribution and fate of transplanted stem cells over time. Green fluorescent protein (GFP) has been used to evaluate cellular engraftment and for long-term cell tracking [3, 4]. Our previous studies showed that transfection of human placental mesenchymal stem cells (hPMSCs) with the GFP gene did not affect their viability, phenotype profile, or pluripotency [5]. However, the impact of GFP labeling on the metabolism of MSCs remains unknown. Metabolomics provides a unique platform for the systematic study of small molecule metabolites from biological samples by allowing the simultaneous assessment of large numbers of metabolites. Metabolomics has been widely applied in various research fields, including biomarker discovery, drug discovery, disease diagnosis, and other biology-related fields [6, 7]. In the context of regenerative medicine, metabolomics analysis of cells has become an important technique in the study of cell biochemistry because large numbers of chemicals can be quantified to provide detailed insight into the metabolic status of cells [8, 9].

Here, we report the development and application of an effective metabolite extraction protocol, along with a highly sensitive metabolomic profiling method based on chemical isotope labeling liquid chromatography-mass spectrometry (CIL LC-MS) to investigate whether GFP gene transduction and expression impacts the metabolism of hPMSCs. High coverage of metabolites was achieved, which allowed better understanding of the potential cellular disturbances induced by GFP labeling.

## 2. Materials and Methods

### 2.1. GFP Gene Transduction

All protocols for human tissue and cell handling were approved by the Research Ethics Committee of the First Affiliated Hospital, School of Medicine, Zhejiang University (reference number 2013-272). The culture and labeling of hPMSCs were performed according to previously reported protocols [5, 10]. The GFP gene was transduced into hPMSCs with a lentivirus to establish GFP^+^hPMSCs. The lentivirus-based short hairpin RNA (shRNA) vector, pGLV3/H1/GFP^+^Puro, containing nonspecific shRNA driven by the H1 promoter containing GFP and puromycin, was purchased from GenePharma Inc. (Shanghai, China). The vectors were integrated and replicated in *Escherichia coli* (Sangon Biotech Corp., Shanghai, China) and purified using a plasmid preparation kit (Qiagen, Hilden, Germany). 293 T cells (Sangon Biotech Corp.) were separately transfected with this plasmid and three packaging plasmids (pGag/Pol, pRev, pVSV-G) using Lipofectamine 2000 (Thermo Fisher Scientific Inc., Waltham, MA, USA) according to the manufacturer's instructions and cultured in the presence of G418 (400 mg/L; Sigma-Aldrich Co., St. Louis, MO, USA). The lentivirus supernatant was collected after 293 T cells reached 80% confluence and stored at −80°C for future use; the titer was determined by GFP expression assay. hPMSCs were seeded and cultured in six-well plates at a density of 1 × 10^5^/well for 24 h in special medium (Mesen Cult® Human Basal Medium plus MesenCult® Human Supplement; STEMCELL Technologies Inc., Vancouver, BC, Canada). On the day of transduction, 2 mL of fresh culture medium containing 5 *μ*g/mL polybrene and lentiviral vectors was added to each well. After 24 h, the medium was then replaced with fresh medium containing 10 *μ*g/mL puromycin (Sigma-Aldrich). The puromycin-resistant cells were observed by a fluorescence phase contrast microscope (IX2-UCB-2; Olympus, Tokyo, Japan) and collected to produce a pool of hPMSCs.

### 2.2. Surface Antigen Detection

GFP^+^hPMSCs (passages 2–5) were collected and incubated in the dark with allophycocyanin-labeled antibodies against CD34, CD45, CD73, CD90, and CD105 (eBioscience, San Diego, CA, USA) for about 30 min. The cells were washed with phosphate-buffered saline and then examined using a flow cytometry (Cytomics FC 500 MPL; Beckman Coulter, Brea, CA, USA).

### 2.3. Metabolite Extraction

Metabolite extraction was performed according to the protocols described by Lorenz et al. [11, 12] with some modifications. Briefly, hPMSCs and GFP^+^hPMSCs at passages 3–5 were seeded on T175 tissue culture flasks (Nunc™ EasYFlasks™; Thermo Fisher Scientific Inc.) and cultured to 80–90% confluence. The metabolites were extracted from the cells using the following procedure: cells were removed from the media, rapidly rinsed using approximately 20 mL of 37°C ultrapure water, and quenched within 5 s by directly adding approximately 15 mL of liquid nitrogen (LN_2_) to the flask. For extraction method 1, 3 mL of ice-cold 9/1 (*v*/*v*) methanol (MeOH)/chloroform (CHCl_3_) (Fisher Chemical, Waltham, MA, USA) was immediately added to the flask. For extraction method 2, 1/1 (*v*/*v*) MeOH/H_2_O (−20°C, 3 mL) was directly added to the culture flask after the washing procedure. The adherent cells were then scraped from the culture flasks using cell scrapers (B-1420; Orange Scientific, Braine-l'Alleud, Belgium). The solutions were transferred to 15 mL centrifuge tubes (Thermo Fisher Scientific Inc.), ultrasonicated for 30 s (KH-250B; Kunshan, Jiangsu, China), and then transferred to 1.5 mL microcentrifuge tubes and spun down at 4°C for 10 min at 16100 ×g (Microfuge 22R; Beckman Coulter). The supernatants were dried using a refrigerated CentriVap concentrator system (Labconco, Kansas City, MO, USA), resuspended in H_2_O, and centrifuged at 4°C for 10 min at 16100 ×g. The supernatants were subsequently transferred to new tubes for labeling.

### 2.4. Derivatization, Normalization, and Mixing

Dansylation labeling was performed according to the protocol reported previously [12]. The ^12^C-dansyl chloride (light chain) was purchased from Sigma-Aldrich, and ^13^C-dansyl chloride (heavy chain) was synthesized in-house as previously described [12]. The individual samples were separately labeled by ^12^C-dansylation or ^13^C-dansylation. In brief, 50 *μ*L of the cell extract was mixed with 25 *μ*L of sodium carbonate/sodium bicarbonate buffer (0.5 mol/L, pH 9.5; Sigma-Aldrich) and 25 *μ*L of acetonitrile (CAN; Sigma-Aldrich) in a screw-capped vial. The vial was vortexed and centrifuged. Then, 50 *μ*L of freshly prepared ^12^C-dansyl chloride or ^13^C-dansyl chloride in ACN (20 mg/mL) was added for light or heavy labeling, respectively. The solution was vortexed and centrifuged again. The dansylation reactions were performed in a water bath at 60°C for 60 min, after which 10 *μ*L of sodium hydroxide solution (250 mM) was added to quench the excess dansyl chloride. After an additional 10 min incubation (60°C), 50 *μ*L of 425 mM formic acid in ACN/H_2_O (1 : 1,*v*/*v*) was added to neutralize the solution. This solution was diluted by adding 10% ACN/0.1% formic acid (FA; Sigma-Aldrich) at a ratio of 1 : 1 (*v*/*v*). The ^12^C-dansylated samples were then mixed with the corresponding ^13^C-dansylated sample from the same extraction method but using the other cell type in a molar ratio of 1 : 1.

After labeling with ^12^C/^13^C-dansyl chloride, the labeled metabolites were quantified by LC*-*UV [13] to control the amount of sample used for metabolome comparison. An ultra-high performance liquid chromatography (UHPLC) system (Agilent 1290; Agilent, Palo Alto, CA, USA) with a photodiode array detector (Agilent) was used for quantification based on the absorption at 338 nm. Briefly, 2 *μ*L of the labeled solution was injected into a Waters ACQUITY UPLC BEH C18 analytical column (i.d. 2.1 × 100 mm, 1.7 *μ*m, pore size 130 Å; Waters Co., Milford, MA, USA). LC solvent A consisted of 0.1% (*v*/*v*) formic acid in water, and LC solvent B consisted of 0.1% (*v*/*v*) formic acid in ACN. The following solvent gradient was used: 15% B for 1 min, increasing to 98% B within 0.01 min, holding at 98% B for 1 min, decreasing to 15% B within 0.5 min, and maintaining this condition for 3.5 min. The flow rate was 500 *μ*L/min.

According to the UV quantification results, appropriate volumes of the ^12^C-dansylated samples were then mixed with the corresponding ^13^C-dansylated sample from the same extraction method but using the other cell type in 1 : 1 molar ratios.

### 2.5. LC-MS

LC-MS was performed using a binary high-performance liquid chromatography system (Agilent 1290 series) connected to an electrospray ionization time-of-flight mass spectrometer (Agilent 6230). Chromatographic separation was carried out on a Waters ACQUITY UPLC BEH C18 analytical column (2.1 × 100 mm, 1.7 *μ*m, pore size 130 Å; Waters Co.); solvent A consisted of water with 0.1% (*v*/*v*) formic acid, and solvent B consisted of ACN with 0.1% (*v*/*v*) formic acid. The gradient was as follows: *t* = 0 min, 15% B; *t* = 2 min, 15% B; *t* = 15 min, 45% B; *t* = 20 min, 65% B; *t* = 26 min, 98% B; *t* = 29 min, 98% B; and *t* = 29.1 min, 15% B. The flow rate was 250 *μ*L/min.

### 2.6. Data Processing and Statistical Analyses

An R-based in-house software tool, IsoMS [14], was used to process the raw data generated from the multiple LC-MS runs. A zero-fill program [15] was used to replace a few intensity values that were missing in the LC-MS runs due to low MS sensitivity. Principal component analysis (PCA) and partial least squares discriminant analysis (PLS-DA) were used for multivariate statistical analyses. Volcano plots were prepared using OriginPro version 9.1 (OriginLab, Wellesley Hills, MA, USA). Hierarchical clustering and heat map generation were performed using R version 3.1 (http://www.r-project.org). The metabolites were identified based on retention time and accurate mass matching to a dansyl standard library or accurate mass matching to the human metabolome database (HMDB). The MyCompoundID program (http://www.mycompoundid.org) was used to search for accurate mass matches within the HMDB. The mass accuracy tolerance window was set at 10 ppm for the database search. Metabolite set enrichment analysis and pathway analysis were based on MetaboAnalyst (http://www.metaboanalyst.ca). The *Homo sapiens* pathway library was used. Cytoscape 3.4.0 on MetScape (http://www.cytoscape.org) was used for large-scale network analysis and the visualization of the integrated metabolism pathways [16].

## 3. Results

### 3.1. Transduction of hPMSCs with Green Fluorescent Protein

**Figure 1:**
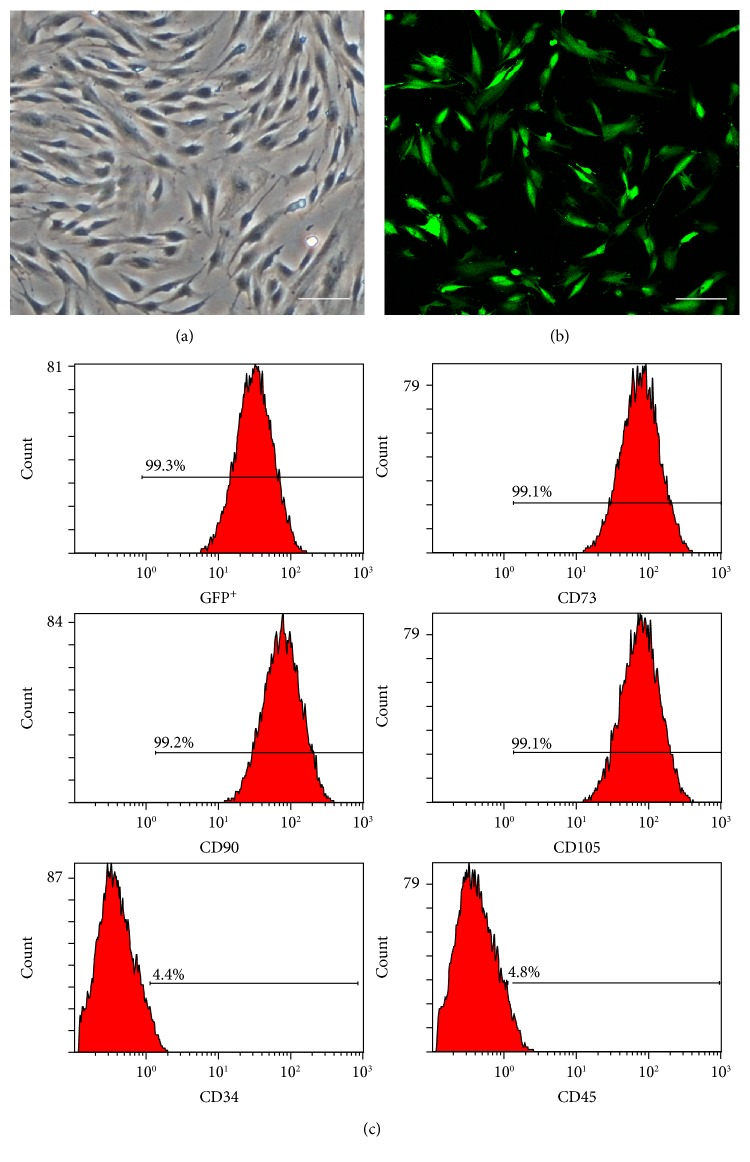
Human placental mesenchymal stem cell (hPMSC) morphology and phenotype profile. (a) hPMSCs without lentiviral transfection. (b) Three days after lentiviral transfection. (c) Surface antigen analysis of green fluorescent protein-positive (GFP^+^) hPMSCs (CD73, CD90, CD105, CD34, and CD45). Scale bars: 100 *μ*m.

hPMSCs were transduced with a lentiviral vector encoding GFP, and the transduction efficiency was assessed directly by fluorescence and confocal microscopy (LSM 710; Carl Zeiss, Jena, Germany). The vector which yielded transduction efficiency was >80% at a multiplicity of infection of 100 : 1. The immunophenotype of GFP^+^hPMSCs was analyzed by flow cytometry, which indicated that the cells were negative for CD45, CD34, and CD79 but expressed high levels of CD73, CD90, and CD105 (Figure 1). This was consistent with the general description of the phenotypic profile of classical hPMSCs [5, 10].

### 3.2. Workflow

In the overall experimental design, the two types of cells were cultured in triplicate. The metabolites were subsequently extracted using two different extraction solvents. Two aliquots were generated from each of the 12 samples, one of which was labeled with ^12^C-dansyl chloride and the other with ^13^C-dansyl chloride. Each ^12^C-dansylated sample was then mixed with the corresponding ^13^C-dansylated sample derived from the same extraction method but using the other cell type. Overall, 12 mixtures were prepared and individually analyzed by LC-MS.

### 3.3. Metabolites from Different Extraction Methods

For 9/1 MeOH/CHCl_3_ extraction (method 1), the numbers of peak pairs detected were 482, 470, 505, 435, 457, and 435 from the six samples (381 common pairs), and 852, 843, 859, 867, 858, and 856 were detected for 1/1 MeOH/H_2_O extraction (method 2) from the six samples (776 common pairs). Online Supplementary Figure S1 available online at https://doi.org/10.1155/2017/3167985 shows a comparison of the peak pairs detected between the two methods, and a larger fraction of the peak pairs was detected in method 2. That is, 327 of a total of 1151 peak pairs detected in all extracts (i.e., 28.41%) were common, 922 pairs (80.10%) were detected in method 2, and 556 pairs (48.30%) were detected in method 1. To obtain a comprehensive list of metabolites, the results from both extraction methods were combined for database search and statistical data analysis.

By searching the 1151 peak pairs against the dansyl standard library, which consists of 273 labeled standards [17], using a mass tolerance of 10 ppm and a retention time (RT) tolerance of 30 s, 89 metabolites were positively identified based on mass and RT matches (see online Supplementary Table S1A for the list). Using MyCompoundID [18] MS search based on the accurate mass of the peak pairs with a mass tolerance of 10 ppm, 281 metabolites were putatively identified using the HMDB library (see online Supplementary Table S1B), and 458 metabolites were putatively identified using the predicted human metabolite library (EML) with one reaction (see online Supplementary Table S1C). Thus, of the 1151 peak pairs, a total of 739 metabolites (64.2%) were positively or putatively identified. The above results indicated that dansylation LC-MS can be used to detect and quantify large numbers of metabolites in cell samples.

**Figure 2:**
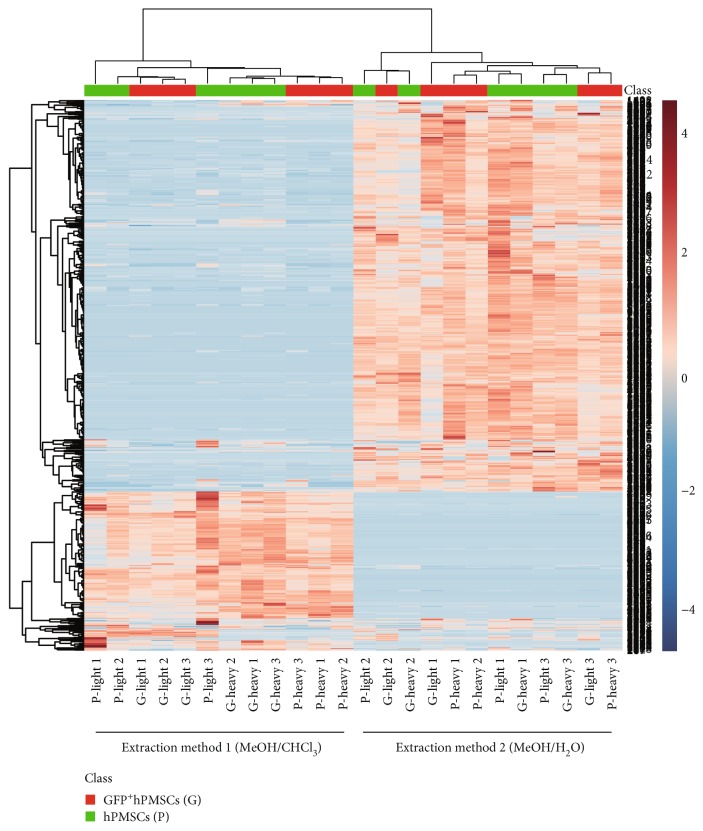
The 9/1 methanol (MeOH)/chloroform (CHCl_3_) and the 1/1 MeOH/H_2_O solvents were used to extract metabolites from the hPMSCs and GFP^+^hPMSCs. The extracts obtained by the two methods were assayed by positive-ion ultra-high performance liquid chromatography-electrospray ionization ultra-mass spectrometry (UHPLC-ESI-MS). A heat map was generated from the liquid chromatography-mass spectrometry (LC-MS) data by the hierarchical clustering algorithm. The hierarchical clusters were calculated from the individual values using 1 Pearson correlation coefficient as distance and complete linkage for agglomeration. Each data point represents the relative intensity of the metabolite in each sample. The red colors presented in the heat map indicate values above the normalized average value, while blue indicates those below the standard value. The color shade is directly proportional to the intensity.

Hierarchical clustering and heat maps were used to examine the metabolites detected from the two sample processing methods. The normalized intensities of the individual metabolites were used, and the metabolite peaks with similar intensity profiles over the LC-MS runs were clustered together. The clustering results are shown in Figure 2 with deeper red colors representing higher intensities and deeper blue colors representing lower intensities. It is interesting that the color distributions were similar within each type of cells (hPMSCs and GFP^+^hPMSCs) but were very different between the two extraction methods (9/1 MeOH/CHCl_3_ and 1/1 MeOH/H_2_O). The results of the hierarchical clustering analysis indicated that the hPMSCs and the GFP^+^hPMSCs did not show markedly distinct metabolite patterns. In addition, the two different extraction solvents were shown to differ significantly in the types of metabolites extracted, so that the metabolites from these two methods were clustered into two distinct groups. These observations indicated that different extraction solvents had marked effects on the metabolite patterns.

Based on the above results, the 1/1 MeOH/H_2_O solvent system provided better extraction, as indicated by the greater number of peak pairs detected and the higher relative peak intensities. However, an integration of the results from these two extraction methods improved the overall metabolome coverage.

### 3.4. Metabolome Profiling

**Figure 3:**
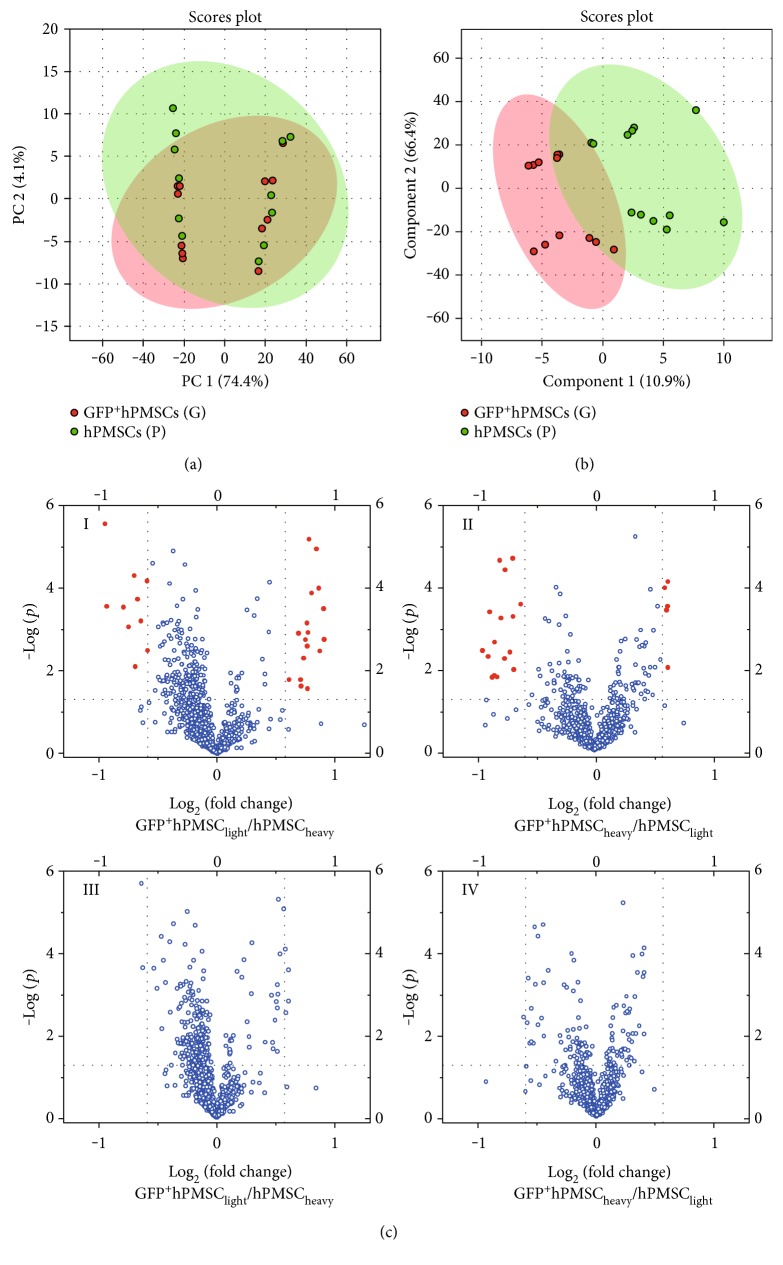
Metabolomics profiling. (a) Principal component analysis (PCA) plot of all of the data obtained from the LC-MS runs. The PCA score plot showed no separation between the hPMSCs and the GFP^+^hPMSCs. (b) Partial least squares discriminant analysis (PLS-DA) score plots of the hPMSCs and the GFP^+^hPMSCs. The PLS-DA score plot did not show a clear, valid separation between the two cell groups. (c) Volcano plots. Volcano plot analyses were used to determine the significant metabolites that separated the two groups (hPMSCs and GFP^+^hPMSCs). The *x*-axis represents the log_2_ of the fold change (FC), which was plotted against the −log of the *p* value. (c)I-II: *p* < 0.05; data points with fold changes > 1.50 or <0.67 are labeled red. (c)III-IV: *p* < 0.05; data points with fold changes > 2 or <0.5 are labeled red (no such data points were observed).

Multivariate statistical analysis was performed using PCA and PLS-DA to examine whether GFP labeling affected the metabolomic profiles of the hPMSCs.  Figure 3(a) shows the relevant PCA score scatter plot of the data (*R*^2^ = 0.496, *Q*^2^ = 0.0984). There was no clear separation between the two groups (hPMSCs and GFP^+^hPMSCs). The PLS-DA models (Figure 3(b)) also showed poor separation between the two experimental groups (*R*^2^ = 0.987, *Q*^2^ = 0.389). Volcano plot analyses, which combined the fold change (FC) and the *p* values from the *t*-tests, were also used to identify the unique metabolites that separated the two groups (hPMSCs and GFP^+^hPMSCs; Figure 3(c)). The blue dots in the volcano plots represent metabolites that are not statistically significant, whereas the red dots indicate those with significant changes, as defined by their *p* values and FC. Metabolites with *p* < 0.05 and FC either >1.5 or <0.67 (i.e., > ± 50% change) were considered significant (red dots). Using these thresholds, only 2.3% (27/1151; Figure 3(c)I) and 1.8% (21/1151; Figure 3(c)II) of the peak pairs changed significantly in response to GFP transfection.

Using the thresholds of FC> 2 or FC< 0.5 and *p* < 0.05, no peak pairs with significant changes were observed (Figure 3(c)III-IV). The volcano plots indicated that the majority of metabolites did not show dramatic changes when the stem cells were tagged with GFP.

### 3.5. Metabolite Quantification and Identification

In the present study, we applied a forward-and-reverse labeling strategy to ensure the confidence of the metabolite quantification results and a lower false-positive rate. The ^12^C-dansyl chloride-labeled metabolites extract from the hPMSCs (P) was mixed with the ^13^C-dansyl chloride-labeled metabolites extracted from the GFP^+^hPMSCs (G) (denoted as G_heavy_P_light_) in a 1 : 1 molar ratio based on the total metabolite content, as measured by LC-UV. Similarly, the ^13^C-dansyl chloride-labeled hPMSC metabolite extract was mixed with the ^12^C-dansyl chloride-labeled metabolites from GFP^+^hPMSCs (denoted as G_light_P_heavy_).

Ideally, the relative ratio of a metabolite peak pair determined from the forward labeled mixture would be the reciprocal of the ratio determined from the reverse labeled mixture. Any deviation from the reciprocal relation is mainly attributable to experimental variations. In our analysis, ratios of 1.50 and 0.67 were set as the thresholds for the selection of metabolites with significant changes. The quantification reproducibility, measured as the standard deviation from the mean for a matched pair in the two mixtures, was also taken into account in this case, and a threshold of 0.1 was used. We then narrowed down the 1151 detected metabolites to 11 correlated markers (Table 1), which were significantly changed in response to GFP transfection.

Five of the 11 potential biomarkers were positively identified in HMDB (i.e., taurine, DL-2-aminooctanoic acid, dityrosine, uracil, and gamma-aminobutyric acid). The concentrations of taurine and gamma-aminobutyric acid decreased after GFP labeling, whereas the concentrations of the other three metabolites increased. Three of these five metabolites (taurine, gamma-aminobutyric acid, and uracil) overlapped with the matching results from the dansyl standard compound library.

### 3.6. Metabolic Pathway Analysis

**Figure 4:**
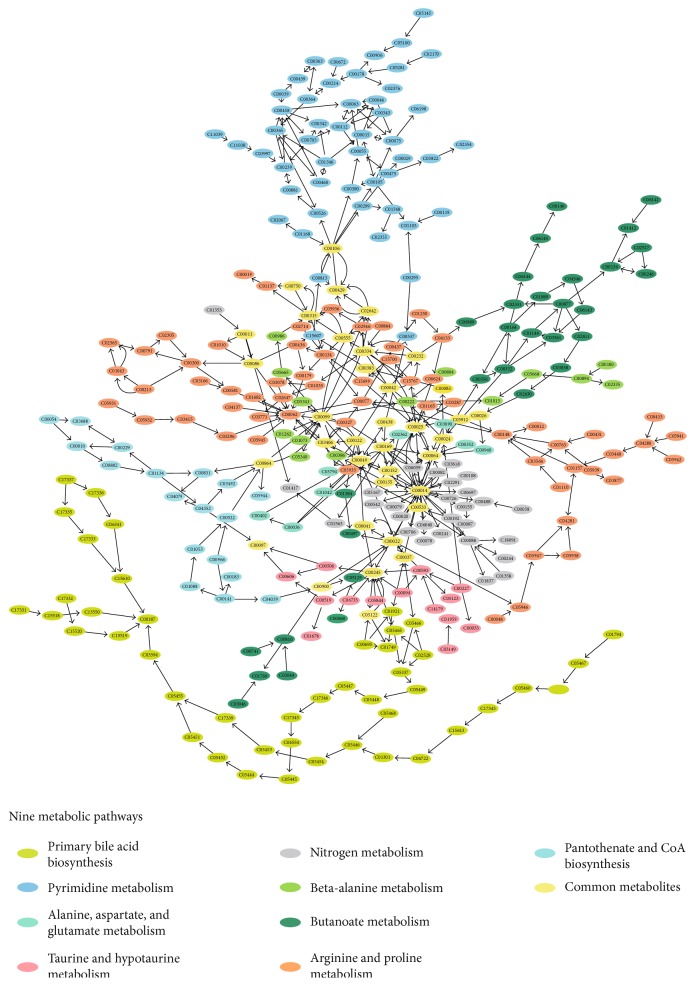
Kyoto Encyclopedia of Genes and Genomes (KEGG) datasets of the nine metabolic pathways were analyzed and visualized using Cytoscape 3.4.0 on MetScape. The network was integrated with 305 compounds from the nine metabolic pathways. A function-guided layout was used to organize the highly connected network. The different metabolic pathways are mapped to different node colors. The corresponding compound of each KEGG ID is shown in online Supplementary Table S2. The yellow nodes represent the common metabolites involved in the different metabolic pathways. The red asterisk-marked node represents the three identified metabolites (taurine, gamma-aminobutyric acid, and uracil).

Metabolomic profiling of the 12 cell samples resulted in the identification of a total of 739 metabolites in the hPMSCs and GFP^+^hPMSCs. Eleven metabolites showed significant changes after labeling of hPMSCs with GFP. Five specific metabolites with significantly higher or lower concentrations in the GFP^+^hPMSCs than the hPMSCs were identified. The relevant metabolic pathways of these five metabolites were determined using MetaboAnalyst. Three of the identified metabolites (taurine, gamma-aminobutyric acid, and uracil) mapped to nine biologically relevant pathways (Table 2): taurine and hypotaurine metabolism (taurine); nitrogen metabolism (taurine); primary bile acid biosynthesis (taurine); alanine, aspartate, and glutamate metabolism (gamma-aminobutyric acid); beta-alanine metabolism (uracil, gamma-aminobutyric acid); butanoate metabolism (gamma-aminobutyric acid); arginine and proline metabolism (gamma-aminobutyric acid); pantothenate and CoA biosynthesis (uracil); and pyrimidine metabolism (uracil). A metabolomics view containing all of the matched pathways based on the pathway enrichment and pathway topology analyses with the Kyoto Encyclopedia of Genes and Genomes (KEGG; http://www.genome.jp/kegg/) pathway database is shown in Figure 4. A higher *p* value and a higher impact value reflect the more relevant pathways affected by GFP labeling. Table 2 shows that these changed metabolites in the GFP^+^hPMSCs are not likely to have a large impact on the metabolic pathways due to their relatively unimportant positions and limited hits (1 or 2). A simplified metabolic network of these nine biologically relevant pathways is presented in Figure 4, covering 305 compounds (see online Supplementary Table S2). Overall, pathway enrichment analysis indicated that GFP labeling did not induce significant changes in metabolism in hPMSCs.

## 4. Discussion

In stem cell therapy, fluorescent protein labeling technique provides a valuable molecular imaging tool to monitor and track the fate and function of cells by providing information on their survival, migration, proliferation, and differentiation status in recipient animals [5, 19, 20]. A recent review listed a few general requirements for the use of agents for cell tracking; for example, these labels must be nontoxic to cells in culture and animal models [21]. To utilize a tracking reagent, such as GFP, it is necessary to determine whether lentiviral transduction and expression of the GFP gene would affect the gene expression, metabolism, and biological characteristics of the cell. Our previous study of MSCs labeled with GFP found no effects on cell viability, proliferation rate, or differentiation capacity [5]. However, there have been no previous studies addressing whether GFP labeling has an impact on the metabolism of MSCs. Quantitative metabolomics techniques can be used to identify changes in metabolite contents, which indicate host cellular responses to gene transfection.

To examine the cellular metabolic changes, it is important to achieve high coverage of metabolites and cover as many pathways as possible. In this regard, the successful extraction of metabolites from cells is a critical step [22]. There have been many in-depth studies on sample preparation for metabolomics analyses of adherent mammalian cells [11–13]. Lorenz et al. reported a convenient and adaptable workflow for the preparation of adherent mammalian cell samples [11]. This workflow was rapid and convenient and provided good sensitivity for detecting a variety of metabolites. The procedure included a rapid water rinse, LN_2_ quenching, and a single-step extraction. For the extraction step, the authors initially evaluated ethanol (EtOH), ACN, MeOH, and 9/1 MeOH/CHCl_3_ for their ability to extract and stabilize metabolites. Overall, the 9/1 MeOH/CHCl_3_ solvent provided superior metabolite recovery and extract stability. In another study, Wu and Li evaluated the performances of three extraction solvent systems (1/1 MeOH/H_2_O, 1/1 ACN/H_2_O, and 2/2/1 MeOH/ACN/H_2_O) [12]. They found that the 1/1 MeOH/H_2_O solvent system performed significantly better than the ACN or MeOH/ACN systems in terms of reproducibility and relative extraction efficiency. Based on these findings and given that different solvent mixtures may have different metabolite extraction and solubility properties, both of these reported sample preparation methods were applied in this study to extract the metabolites of hPMSCs before and after GFP gene transfection with 9/1 MeOH/CHCl_3_ and 1/1 MeOH/H_2_O as extraction solvents. Our results showed that the 1/1 MeOH/H_2_O solvent extracted more metabolites with higher peak intensities than the 9/1 MeOH/CHCl_3_ solvent_._ Even though 327 metabolites were detected in both methods, hundreds of unique metabolites were extracted from each of the two methods, indicating that metabolites with different chemical and physical properties could be extracted from different solvent systems. Integration of the metabolites extracted from both methods represented a better coverage of the metabolome and provided a more comprehensive view of the changes that happened in cellular metabolism.

In addition to achieving efficient metabolite extraction, metabolite detection is also critical to achieve high metabolome coverage. Among the various analytical platforms used for metabolome analysis, LC-MS is the most widely used [23]. Recently, we developed a chemical isotope labeling technique (CIL LC-MS) that is suitable for profiling metabolites containing primary or secondary amines or phenol groups [24]. For CIL LC-MS, ^12^C/^13^C-dansyl reagent is added to label the metabolites before loading the samples into an LC-MS system. These isotope-labeled metabolites are then detected as peak pairs in the mass spectra, and the peak intensity ratio is used for metabolic quantification. Due to the chemical properties of the dansyl group, this technique provides a 10–1000-fold increase in detection sensitivity and is capable of separating polar and ionic metabolites after labeling, enabling the detection of thousands of metabolites using one-dimensional LC-MS. The use of isotope labeling of metabolites from two different groups of cells also provides much-improved quantification accuracy as the light/heavy metabolite ion pairs always experience the same matrix when detected in the mass spectrometer [25]. Chemical isotope labeling has been successfully applied to metabolomics studies of various biological samples to discover potential biomarkers and investigate cellular metabolomics [24, 26, 27]. In this study, the differential ^12^C/^13^C-dansylation labeling strategy allowed 1151 metabolite peak pairs to be detected from adherent mammalian cells. In particular, the forward-and-reverse labeling strategy we have applied allowed us to minimize the impact of system errors and greatly enhanced the confidence of both the identification and the quantification results.

Multivariate statistical analyses of the quantitative data using PCA and PLS-DA showed poor separation between the hPMSCs and GFP^+^hPMSCs. Volcano plot analyses also demonstrated that, for thresholds of FC > 1.5 or FC < 0.67 and *p* < 0.05, the majority of the metabolites (98.0%) were not significantly changed when the hPMSCs were labeled with GFP. No metabolites showed significant changes for thresholds of FC > 2 or FC < 0.5 and *p* < 0.05. Hierarchical clustering and heat maps also revealed that the hPMSCs and the GFP^+^hPMSCs did not show markedly distinct metabolite patterns. In addition, among the 1151 metabolites detected, only 11 showed significant changes after GFP labeling, and five of these metabolites were identified. Metabolic pathway analysis indicated that three of these metabolites (taurine, uracil, and gamma-aminobutyric acid) were involved in nine pathways, with only one or two hits. And two other metabolites, DL-2-aminooctanoic acid and dityrosine, were not in the KEGG pathway database. However, due to their relatively nonessential positions, these changes were unlikely to markedly affect the metabolic pathways.

## 5. Conclusions

In this study, as well as in our previous studies, we report that the labeling of MSCs with GFP is safe and does not affect the metabolism or functions of MSCs. This is the first reported study of the metabolism of MSCs and GFP^+^hPMSCs using an isotope labeling LC-MS method, which provides a new method to examine the metabolism of stem cells for cell-based therapies.

## Supplementary Material

Supplemental Table S1A. Identification of cellular metabolites based on the mass and retention time matches against the dansyl standard library. Supplemental Table S1B. Identification of cellular metabolites by searching the accurate mass of the peak pairs against the HMDB library. Supplemental Table S1C. Identification of cellular metabolites by searching the accurate mass of the peak pairs against the EML library with one reaction in MCID.Figure S1. The Venn diagram shows the numbers of peak pairs detected using 9/1 MeOH/CHCl3 (method 1) and 1/1 MeOH/H2O (method 2) as extraction solvent respectively.

## Figures and Tables

**(a):** 

No.	Retention time (s) m/z		^∆^PG average	^†^GP average	Candidate name	HMDB umber	Formula
1	200.90	125.0152	1.89	0.47	Taurine^∗^	HMDB00251	C_2_H_7_NO_3_S
2	505.02	103.0634	2.02	0.55	Gamma-aminobutyric acid^∗^	HMDB00112	C_4_H_9_NO_2_
3	621.05	316.1400	0.69	1.58	Dityrosine	HMDB06045	C_18_H_20_N_2_O_6_
4	703.73	112.0278	0.42	2.39	Uracil^∗^	HMDB00300	C_4_H_4_N_2_O_2_
5	710.63	327.6295	0.57	1.83			
6	780.23	319.6334	0.62	1.51			
7	1046.74	380.5105	0.67	1.57			
8	1127.03	243.4506	0.61	1.71			
9	1156.70	87.1059	0.67	1.60			
10	1252.74	101.1210	0.56	1.65			
11	1336.96	115.1352	0.59	1.74	dl-2-aminooctanoic acid	HMDB00991	C_8_H_17_NO_2_

**(b):** 

No.	^∆^PG1-1 ratio	^∆^PG1-2 ratio	^∆^PG1-3 ratio	^∆^PG2-1 ratio	^∆^PG2-2 ratio	^∆^PG2-3 ratio	^†^GP1-1 ratio	^†^GP1-2 ratio	^†^GP1-3 ratio	^†^GP2-1 ratio	^†^GP2-2 ratio	^†^GP2-3 ratio
1	3.09	1.33	2.80	1.58	1.47	1.07	0.21	0.56	0.34	0.47	0.57	0.65
2	3.00	1.99	2.62	1.46	1.36	1.71	0.43	0.42	0.56	0.69	0.83	0.40
3	NA	NA	NA	0.83	0.74	0.50	NA	NA	NA	1.33	1.78	1.64
4	NA	NA	NA	0.23	0.56	0.46	NA	NA	NA	3.92	1.47	1.77
5	NA	NA	NA	0.76	0.34	0.62	NA	NA	NA	1.03	3.00	1.45
6	NA	NA	NA	0.95	0.36	0.54	NA	NA	NA	0.75	2.47	1.29
7	NA	NA	NA	0.89	0.42	0.69	NA	NA	NA	1.19	2.28	1.24
8	NA	NA	NA	0.71	0.69	0.43	NA	NA	NA	1.52	1.54	2.09
9	NA	NA	NA	0.98	0.44	0.59	NA	NA	NA	0.83	2.12	1.84
10	NA	NA	NA	0.77	0.45	0.46	NA	NA	NA	1.15	2.09	1.71
11	NA	NA	NA	0.82	0.37	0.58	NA	NA	NA	0.94	2.65	1.64

^∗^Metabolites that overlapped with the matching result from the dansyl standard compound library. ^∆†^List of 11 significant metabolites with correlated responses from the hPMSCs (P) and the GFP^+^hPMSCs (G) with the different extraction methods (1, 2) in triplicate (−1 to −3). HMDB: human metabolome database.

**Table 2:** Pathway analysis of the three identified metabolites (taurine, gamma-aminobutyric acid, and uracil) using MetaboAnalyst. These metabolites were related to nine metabolic pathways.

No.	Metabolic pathway	Total	Expected	Hits	FDR	Impact
1	Beta-alanine metabolism	28	0.034898	2	0.031104	0
2	Taurine and hypotaurine metabolism	20	0.024927	1	0.65401	0.33094
3	Alanine, aspartate, and glutamate metabolism	24	0.029913	1	0.65401	0.10256
4	Pantothenate and CoA biosynthesis	27	0.033652	1	0.65401	0
5	Nitrogen metabolism	39	0.048608	1	0.65401	0
6	Butanoate metabolism	40	0.049855	1	0.65401	0.01067
7	Primary bile acid biosynthesis	47	0.058579	1	0.65676	0.00822
8	Pyrimidine metabolism	60	0.074782	1	0.72963	0.07132
9	Arginine and proline metabolism	77	0.09597	1	0.8264	0.01905

## Abbreviations

MSCs: Mesenchymal stem cells
hPMSCs: Human placental mesenchymal stem cells
GFP: Green fluorescent protein
UHPLC-Q/TOF-MS: Ultra-high performance liquid chromatography-quadrupole/time-of-flight mass spectrometry
CIL LC-MS: Chemical isotope labeling liquid chromatography-mass spectrometry
PCA: Principal component analysis
PLS-DA: Partial least squares discriminant analysis
HMDB: Human metabolome database
BPI: Based peak intensity
LN_2_: Liquid nitrogen
MeOH: Methyl alcohol
CHCl_3_: Trichloromethane
ACN: Acetonitrile
FA: Formic acid
RT: Retention time.

## Additional Points

*Highlights.* (i) ^13^C/^12^C-dansyl labeling provides 10–1000-fold increases in sensitivity for relative quantification of individual metabolites. (ii) A total of 1151 peak pairs or metabolites were detected. Only 11 metabolites showed significant changes after labeling of human placental mesenchymal stem cells (hPMSCs) with green fluorescent protein (GFP). Pathway analyses using MetaboAnalyst indicated that GFP labeling did not induce significant changes in hPMSC metabolism. (iii) hPMSCs and GFP^+^hPMSCs did not show markedly distinct metabolite patterns, as determined based on the results of principal component analysis, partial least squares discriminant analysis, volcano plots, and hierarchical clustering analysis.
