# Supplementary material for: The Impact of GFP Reporter Gene Transduction and Expression on Metabolomics of Placental Mesenchymal Stem Cells Determined by UHPLC-Q/TOF-MS
Source: Stem Cells Int. 2017 Nov 5;2017:3167985. doi: 10.1155/2017/3167985 (PMC5694582; doi:10.1155/2017/3167985)
Supplement: Supplementary file 1 — Supplemental Table S1A. Identification of cellular metabolites based on the mass and retention time matches against the dansyl standard library. Supplemental Table S1B. Identification of cellular metabolites by searching the accurate mass of the peak pairs against the HMDB library. Supplemental Table S1C. Identification of cellular metabolites by searching the accurate mass of the peak pairs against the EML library with one reaction in MCID.Figure S1. The Venn diagram shows the numbers of peak pairs detected using 9/1 MeOH/CHCl3 (method 1) and 1/1 MeOH/H2O (method 2) as extraction solvent respectively. [file 3167985.f1.doc]

**Supplemental Data**

# The impact of GFP reporter gene transduction and expression on metabolomics of placental mesenchymal stem cells determined by UHPLC-Q/TOF-MS

Jinfeng Yang^1^*, Nan Wang^2^*, Deying Chen^1^, Jiong Yu^1^, Qiaoling Pan^1^, Dan Wang^1^, Jingqi Liu^1^, Xiaowei Shi^3^, Xiaotian Dong^1^, Hongcui Cao^1^†, Liang Li^2^, Lanjuan Li^1^

1 State Key Laboratory for the Diagnosis and Treatment of Infectious Diseases, The First Affiliated Hospital, College of Medicine, Zhejiang University; Collaborative Innovation Center for Diagnosis and Treatment of Infectious Diseases, 79 Qingchun Rd., Hangzhou City 310003, China

2 Collaborative Innovation Center for the Diagnosis and Treatment of Infectious Diseases, Zhejiang University, Hangzhou 310003, China; Department of Chemistry, University of Alberta, Edmonton, Alberta T6G 2G2, Canada

3 Chu Kochen Honors College, Zhejiang University, 866 Yuhangtang Rd., Hangzhou City310058, China

* These authors contributed equally to this work.

†Correspondence author:

Hongcui Cao, the State Key Laboratory for Diagnosis and Treatment of Infectious Diseases, The First Affiliated Hospital, College of Medicine, Zhejiang University; Collaborative Innovation Center for Diagnosis and Treatment of Infectious Diseases,79 Qingchun Rd., Hangzhou City 310003, China.Tel: 86-571-87236451; Fax: 86-571-87236459

E-mail: [hccao@zju.edu.cn](mailto:hccao@zju.edu.cn)

**Figure S1.**


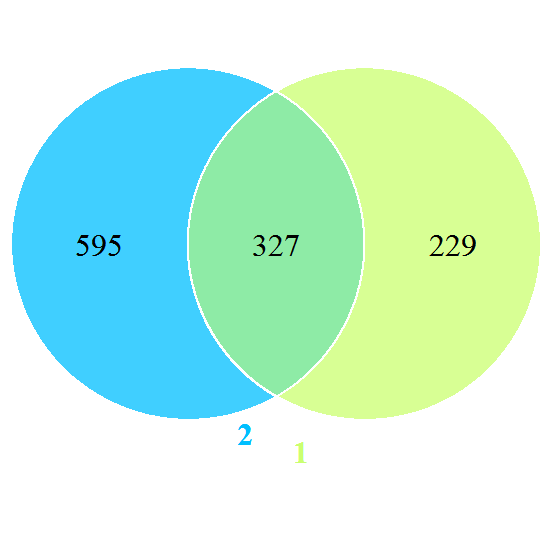


The Venn diagram shows the numbers of peak pairs detected using 9/1 MeOH/CHCl_3_ (method 1) and 1/1 MeOH/H_2_O (method 2) as extraction solvent respectively.

**Table S1A.** Identification of cellular metabolites based on the mass and retention time matches against the dansyl standard library.

Supplemental Table S1A. Identification of cellular metabolites based on the mass and retention time matches against the dansyl standard library.

| # | Input mass | Input RT(min) | Calibrated RT (min) | HMDB.No. | Name | Accurate mass | mz_light | library RT | Mass error ( Da) | RT error (min) |
| --- | --- | --- | --- | --- | --- | --- | --- | --- | --- | --- |
| 1 | 375.0800 | 3.08 | 1.80 | HMDB00224 | O-Phosphoethanolamine | 141.0191 | 375.0774 | 2.02 | 0.0026 | 0.22 |
| 2 | 359.0735 | 3.35 | 1.95 | HMDB00251 | Taurine | 125.0147 | 359.0730 | 2.24 | 0.0005 | 0.29 |
| 3 | 388.1080 | 3.48 | 2.03 | HMDB00157 | Hypoxanthine + H2O | 136.0385 | 388.1098 | 2.12 | 0.0018 | 0.09 |
| 4 | 517.1505 | 3.57 | 2.09 | HMDB00133 | Guanosine | 283.0917 | 517.1500 | 2.22 | 0.0005 | 0.13 |
| 5 | 359.0735 | 3.59 | 2.09 | HMDB00251 | Taurine | 125.0147 | 359.0730 | 2.24 | 0.0005 | 0.15 |
| 6 | 517.1506 | 3.78 | 2.21 | HMDB00133 | Guanosine | 283.0917 | 517.1500 | 2.22 | 0.0006 | 0.01 |
| 7 | 517.1502 | 3.99 | 2.33 | HMDB00133 | Guanosine | 283.0917 | 517.1500 | 2.22 | 0.0002 | 0.11 |
| 8 | 408.1709 | 4.03 | 2.35 | HMDB00517 | L-Arginine | 174.1117 | 408.1700 | 2.44 | 0.0009 | 0.09 |
| 9 | 403.1427 | 4.19 | 2.45 | HMDB00001 | 1-Methylhistidine | 169.0851 | 403.1434 | 2.17 | 0.0007 | 0.28 |
| 10 | 403.1427 | 4.19 | 2.45 | HMDB00479 | 3-methyl-histidine | 169.0851 | 403.1434 | 2.01 | 0.0007 | 0.44 |
| 11 | 408.1705 | 4.37 | 2.73 | HMDB00517 | L-Arginine | 174.1117 | 408.1700 | 2.44 | 0.0005 | 0.29 |
| 12 | 366.1125 | 4.45 | 2.87 | HMDB00168 | L-Asparagine | 132.0535 | 366.1118 | 3 | 0.0007 | 0.13 |
| 13 | 380.1279 | 4.91 | 3.63 | HMDB00641 | L-Glutamine | 146.0691 | 380.1275 | 3.32 | 0.0004 | 0.31 |
| 14 | 380.1279 | 4.91 | 3.63 | HMDB03423 | D-Glutamine | 146.0691 | 380.1275 | 3.32 | 0.0004 | 0.31 |
| 15 | 399.1044 | 5.14 | 4.00 | HMDB02005 | Methionine Sulfoxide | 165.0460 | 399.1043 | 3.72 | 0.0001 | 0.28 |
| 16 | 399.1044 | 5.14 | 4.00 | HMDB02005 | Methionine Sulfoxide - Isomer | 165.0460 | 399.1043 | 4.2 | 0.0001 | 0.20 |
| 17 | 353.1166 | 5.23 | 4.15 | HMDB00719 | L-Homoserine | 119.0582 | 353.1166 | 4.05 | 0.0000 | 0.10 |
| 18 | 339.1030 | 5.23 | 4.15 | HMDB00187 | L-Serine | 105.0426 | 339.1009 | 4.4 | 0.0021 | 0.25 |
| # | Input mass | Input RT(min) | Calibrated RT (min) | HMDB.No. | Name | Accurate mass | mz_light | library RT | Mass error ( Da) | RT error (min) |
| 19 | 399.1046 | 5.41 | 4.44 | HMDB02005 | Methionine Sulfoxide - Isomer | 165.0460 | 399.1043 | 4.2 | 0.0003 | 0.24 |
| 20 | 353.1157 | 5.47 | 4.50 | HMDB00719 | L-Homoserine | 119.0582 | 353.1166 | 4.05 | 0.0009 | 0.45 |
| 21 | 339.1016 | 5.58 | 4.61 | HMDB00187 | L-Serine | 105.0426 | 339.1009 | 4.4 | 0.0007 | 0.21 |
| 22 | 381.1122 | 5.61 | 4.63 | HMDB00148 | L-Glutamic Acid | 147.0532 | 381.1115 | 5.05 | 0.0007 | 0.42 |
| 23 | 367.0991 | 5.66 | 4.68 | HMDB00191 | L-Aspartic Acid | 133.0375 | 367.0958 | 5.16 | 0.0033 | 0.48 |
| 24 | 365.1186 | 5.68 | 4.70 | HMDB00725 | Trans-4-Hydroxyl-L-Proline | 131.0582 | 365.1166 | 5.17 | 0.0020 | 0.47 |
| 25 | 365.1187 | 5.86 | 4.87 | HMDB00725 | Trans-4-Hydroxyl-L-Proline | 131.0582 | 365.1166 | 5.17 | 0.0021 | 0.30 |
| 26 | 365.1183 | 6.06 | 5.06 | HMDB00725 | Trans-4-Hydroxyl-L-Proline | 131.0582 | 365.1166 | 5.17 | 0.0017 | 0.11 |
| 27 | 381.1125 | 6.12 | 5.12 | HMDB00148 | L-Glutamic Acid | 147.0532 | 381.1115 | 5.05 | 0.0010 | 0.07 |
| 28 | 365.1175 | 6.25 | 5.24 | HMDB00725 | Trans-4-Hydroxyl-L-Proline | 131.0582 | 365.1166 | 5.17 | 0.0009 | 0.07 |
| 29 | 353.1176 | 6.31 | 5.30 | HMDB00167 | L-Threonine | 119.0582 | 353.1166 | 5.79 | 0.0010 | 0.49 |
| 30 | 353.1178 | 6.62 | 5.59 | HMDB00167 | L-Threonine | 119.0582 | 353.1166 | 5.79 | 0.0012 | 0.20 |
| 31 | 353.1176 | 6.92 | 5.94 | HMDB00167 | L-Threonine | 119.0582 | 353.1166 | 5.79 | 0.0010 | 0.15 |
| 32 | 295.1116 | 7.02 | 6.09 | HMDB00149 | Ethanolamine | 61.0528 | 295.1111 | 6 | 0.0005 | 0.09 |
| 33 | 395.1274 | 7.07 | 6.18 | HMDB00510 | Aminoadipic acid | 161.0688 | 395.1271 | 5.97 | 0.0003 | 0.21 |
| 34 | 309.0913 | 7.14 | 6.29 | HMDB00123 | Glycine | 75.0320 | 309.0903 | 6.59 | 0.0010 | 0.30 |
| 35 | 323.1066 | 7.54 | 6.94 | HMDB00056 | Beta-Alanine | 89.0477 | 323.1060 | 7.24 | 0.0006 | 0.30 |
| 36 | 323.1069 | 7.91 | 7.55 | HMDB00056 | Beta-Alanine | 89.0477 | 323.1060 | 7.24 | 0.0009 | 0.31 |
| 37 | 323.1069 | 7.91 | 7.55 | HMDB00161 | L-Alanine | 89.0477 | 323.1060 | 7.57 | 0.0009 | 0.02 |
| 38 | 323.1073 | 8.36 | 7.97 | HMDB00161 | L-Alanine | 89.0477 | 323.1060 | 7.57 | 0.0013 | 0.40 |
| 39 | 337.1217 | 8.42 | 8.03 | HMDB00112 | Gamma-Aminobutyric acid | 103.0633 | 337.1216 | 7.79 | 0.0001 | 0.24 |
| # | Input mass | Input RT(min) | Calibrated RT (min) | HMDB.No. | Name | Accurate mass | mz_light | library RT | Mass error ( Da) | RT error (min) |
| 40 | 337.1243 | 8.62 | 8.21 | HMDB00112 | Gamma-Aminobutyric acid | 103.0633 | 337.1216 | 7.79 | 0.0027 | 0.42 |
| 41 | 337.1243 | 8.62 | 8.21 | HMDB03911 | 3-Aminoisobutanoic acid | 103.0633 | 337.1216 | 8.67 | 0.0027 | 0.46 |
| 42 | 351.1377 | 8.81 | 8.39 | HMDB03355 | 5-Aminopentanoic acid | 117.0790 | 351.1373 | 8.68 | 0.0004 | 0.29 |
| 43 | 363.1018 | 9.66 | 9.16 | HMDB00148 | L-Glutamic Acid - H2O | 147.0532 | 363.1009 | 9.46 | 0.0009 | 0.30 |
| 44 | 337.1234 | 9.78 | 9.27 | HMDB00452 | L-Alpha-aminobutyric acid | 103.0633 | 337.1216 | 9.13 | 0.0018 | 0.14 |
| 45 | 337.1234 | 9.78 | 9.27 | HMDB00650 | D-Alpha-aminobutyric acid | 103.0633 | 337.1216 | 9.23 | 0.0018 | 0.04 |
| 46 | 337.1234 | 9.78 | 9.27 | HMDB01906 | 2-Aminoisobutyric acid | 103.0633 | 337.1216 | 8.91 | 0.0018 | 0.36 |
| 47 | 370.0991 | 9.88 | 9.36 | HMDB00157 | Hypoxanthine - Isomer | 136.0385 | 370.0968 | 9.65 | 0.0023 | 0.29 |
| 48 | 363.1019 | 9.91 | 9.39 | HMDB00148 | L-Glutamic Acid - H2O | 147.0532 | 363.1009 | 9.46 | 0.0010 | 0.07 |
| 49 | 337.1233 | 9.96 | 9.43 | HMDB00452 | L-Alpha-aminobutyric acid | 103.0633 | 337.1216 | 9.13 | 0.0017 | 0.30 |
| 50 | 337.1233 | 9.96 | 9.43 | HMDB00650 | D-Alpha-aminobutyric acid | 103.0633 | 337.1216 | 9.23 | 0.0017 | 0.20 |
| 51 | 337.1216 | 10.12 | 9.58 | HMDB00452 | L-Alpha-aminobutyric acid | 103.0633 | 337.1216 | 9.13 | 0.0000 | 0.45 |
| 52 | 337.1216 | 10.12 | 9.58 | HMDB00650 | D-Alpha-aminobutyric acid | 103.0633 | 337.1216 | 9.23 | 0.0000 | 0.35 |
| 53 | 399.1235 | 10.48 | 9.90 | HMDB00897 | 7-Methylguanine | 165.0651 | 399.1234 | 10.32 | 0.0001 | 0.42 |
| 54 | 399.1235 | 10.48 | 9.90 | HMDB03282 | 1-Methylguanine | 165.0651 | 399.1234 | 9.57 | 0.0001 | 0.33 |
| 55 | 349.1229 | 10.64 | 10.05 | HMDB00162 | L-Proline | 115.0633 | 349.1216 | 10.18 | 0.0013 | 0.13 |
| 56 | 349.1226 | 10.99 | 10.35 | HMDB00162 | L-Proline | 115.0633 | 349.1216 | 10.18 | 0.0010 | 0.17 |
| 57 | 422.1703 | 11.29 | 10.59 | HMDB28844 | Glycyl-Isoleucine | 188.1161 | 422.1744 | 10.78 | 0.0041 | 0.19 |
| 58 | 351.1380 | 11.53 | 10.78 | HMDB00883 | L-Valine | 117.0790 | 351.1373 | 10.81 | 0.0007 | 0.03 |
| 59 | 383.1107 | 11.70 | 10.92 | HMDB00696 | L-Methionine | 149.0510 | 383.1094 | 10.89 | 0.0013 | 0.03 |
| 60 | 346.0862 | 11.73 | 10.95 | HMDB00300 | Uracil | 112.0273 | 346.0856 | 11.34 | 0.0006 | 0.39 |
| # | Input mass | Input RT(min) | Calibrated RT (min) | HMDB.No. | Name | Accurate mass | mz_light | library RT | Mass error ( Da) | RT error (min) |
| 61 | 351.1381 | 11.76 | 10.98 | HMDB00883 | L-Valine | 117.0790 | 351.1373 | 10.81 | 0.0008 | 0.17 |
| 62 | 422.1734 | 12.30 | 11.54 | HMDB00759 | Glycyl-L-Leucine | 188.1161 | 422.1744 | 11.22 | 0.0010 | 0.32 |
| 63 | 438.1492 | 12.46 | 11.71 | HMDB00929 | L-Tryptophan | 204.0899 | 438.1482 | 11.44 | 0.0010 | 0.27 |
| 64 | 456.1571 | 12.73 | 12.00 | HMDB28848 | Glycyl-Phenylalanine | 222.1004 | 456.1588 | 11.65 | 0.0017 | 0.35 |
| 65 | 383.1296 | 13.29 | 12.59 | HMDB02099 | 6-Methyladenine | 149.0701 | 383.1285 | 12.22 | 0.0011 | 0.37 |
| 66 | 383.1296 | 13.29 | 12.59 | HMDB02099 | 6-Methyladenine - Isomer | 149.0701 | 383.1285 | 12.74 | 0.0011 | 0.15 |
| 67 | 399.1387 | 13.46 | 12.77 | HMDB00159 | L-Phenylalanine | 165.0790 | 399.1373 | 12.74 | 0.0014 | 0.03 |
| 68 | 365.1540 | 13.69 | 12.96 | HMDB00172 | L-Isoleucine | 131.0946 | 365.1529 | 13.06 | 0.0011 | 0.10 |
| 69 | 365.1540 | 13.69 | 12.96 | HMDB00557 | L-Alloisoleucine | 131.0946 | 365.1529 | 13.2 | 0.0011 | 0.24 |
| 70 | 365.1540 | 13.69 | 12.96 | HMDB00687 | L-leucine | 131.0946 | 365.1529 | 13.36 | 0.0011 | 0.40 |
| 71 | 365.1538 | 13.95 | 13.18 | HMDB00172 | L-Isoleucine | 131.0946 | 365.1529 | 13.06 | 0.0009 | 0.12 |
| 72 | 365.1538 | 13.95 | 13.18 | HMDB00557 | L-Alloisoleucine | 131.0946 | 365.1529 | 13.2 | 0.0009 | 0.02 |
| 73 | 365.1538 | 13.95 | 13.18 | HMDB00687 | L-leucine | 131.0946 | 365.1529 | 13.36 | 0.0009 | 0.18 |
| 74 | 364.6260 | 14.16 | 13.36 | HMDB04987 | Alpha-Aspartyl-lysine | 261.1325 | 364.6246 | 13.61 | 0.0014 | 0.25 |
| 75 | 365.1495 | 14.24 | 13.42 | HMDB00172 | L-Isoleucine | 131.0946 | 365.1529 | 13.06 | 0.0034 | 0.36 |
| 76 | 365.1495 | 14.24 | 13.42 | HMDB00557 | L-Alloisoleucine | 131.0946 | 365.1529 | 13.2 | 0.0034 | 0.22 |
| 77 | 365.1495 | 14.24 | 13.42 | HMDB00687 | L-leucine | 131.0946 | 365.1529 | 13.36 | 0.0034 | 0.06 |
| 78 | 345.0932 | 14.65 | 13.77 | HMDB00099 | L-Cystathionine | 222.0674 | 345.0920 | 13.34 | 0.0012 | 0.43 |
| 79 | 345.0932 | 14.65 | 13.77 | HMDB00099 | L-Cystathionine - Isomer | 222.0674 | 345.0920 | 13.69 | 0.0012 | 0.08 |
| 80 | 345.0932 | 14.65 | 13.77 | HMDB00455 | Allocystathionine | 222.0674 | 345.0920 | 13.33 | 0.0012 | 0.44 |
| 81 | 345.0932 | 14.65 | 13.77 | HMDB00455 | Allocystathionine - Isomer | 222.0674 | 345.0920 | 13.61 | 0.0012 | 0.16 |
| # | Input mass | Input RT(min) | Calibrated RT (min) | HMDB.No. | Name | Accurate mass | mz_light | library RT | Mass error ( Da) | RT error (min) |
| 82 | 416.1171 | 14.80 | 13.90 | HMDB00755 | Hydroxyphenyllactici acid | 182.0579 | 416.1162 | 14.39 | 0.0009 | 0.49 |
| 83 | 354.0713 | 15.05 | 14.11 | HMDB00192 | L-Cystine | 240.0238 | 354.0702 | 14.11 | 0.0011 | 0.00 |
| 84 | 300.1039 | 16.35 | 16.29 | HMDB00214 | Ornithine | 132.0899 | 300.1033 | 16.58 | 0.0006 | 0.29 |
| 85 | 307.1128 | 17.06 | 17.47 | HMDB00182 | L-Lysine | 146.1055 | 307.1111 | 17.47 | 0.0017 | 0.00 |
| 86 | 389.1281 | 17.77 | 18.56 | HMDB00177 | L-Histidine | 155.0695 | 389.1278 | 18.09 | 0.0003 | 0.47 |
| 87 | 324.5968 | 20.44 | 22.66 | HMDB00158 | L-Tyrosine | 181.0739 | 324.5953 | 22.65 | 0.0015 | 0.01 |
| 88 | 324.5968 | 20.44 | 22.66 | HMDB06050 | o-Tyrosine | 181.0739 | 324.5953 | 22.38 | 0.0015 | 0.28 |
| 89 | 289.0777 | 24.19 | 26.41 | HMDB00957 | pyrocatechol | 110.0368 | 289.0767 | 26.7 | 0.0010 | 0.29 |

Table S1B. Identification of cellular metabolites by searching the accurate mass of the peak pairs against the HMDB library.

| # | RT (s) | mz_light | mz_heavy | mz | distance | int_light | nCharge | nTag | Possible hits |
| --- | --- | --- | --- | --- | --- | --- | --- | --- | --- |
| 1 | 128.55 | 375.07841 | 377.08489 | 141.02009 | 2.00648 | 1090000 | 1 | 1 | [1](http://www.mycompoundid.org/mycompoundid_IsoMS/myid_search_res/1481082226320-2016-12-6-8-43-46-858582/1481082226322-119-141.020086-Neutral.htm) |
| 2 | 130.55 | 367.09644 | 369.10373 | 133.03812 | 2.00730 | 1100000 | 1 | 1 | [3](http://www.mycompoundid.org/mycompoundid_IsoMS/myid_search_res/1481082226320-2016-12-6-8-43-46-858582/1481082226323-681-133.038116-Neutral.htm) |
| 3 | 135.60 | 449.11410 | 451.11910 | 215.05578 | 2.00500 | 29175 | 1 | 1 | [1](http://www.mycompoundid.org/mycompoundid_IsoMS/myid_search_res/1481082226320-2016-12-6-8-43-46-858582/1481082226323-525-215.0557801-Neutral.htm) |
| 4 | 135.83 | 381.11109 | 383.11661 | 147.05277 | 2.00552 | 14850 | 1 | 1 | [6](http://www.mycompoundid.org/mycompoundid_IsoMS/myid_search_res/1481082226320-2016-12-6-8-43-46-858582/1481082226324-123-147.05277-Neutral.htm) |
| 5 | 136.16 | 366.11233 | 368.11887 | 132.05401 | 2.00654 | 3630000 | 1 | 1 | [4](http://www.mycompoundid.org/mycompoundid_IsoMS/myid_search_res/1481082226320-2016-12-6-8-43-46-858582/1481082226324-406-132.0540115-Neutral.htm) |
| 6 | 151.91 | 380.12827 | 382.13458 | 146.06995 | 2.00631 | 5385000 | 1 | 1 | [4](http://www.mycompoundid.org/mycompoundid_IsoMS/myid_search_res/1481082226320-2016-12-6-8-43-46-858582/1481082226326-619-146.0699525-Neutral.htm) |
| 7 | 152.99 | 280.10057 | 282.10707 | 46.04225 | 2.00650 | 533125 | 1 | 1 | [1](http://www.mycompoundid.org/mycompoundid_IsoMS/myid_search_res/1481082226320-2016-12-6-8-43-46-858582/1481082226326-589-46.04224869-Neutral.htm) |
| 8 | 154.60 | 449.11495 | 451.12088 | 215.05663 | 2.00594 | 295375 | 1 | 1 | [1](http://www.mycompoundid.org/mycompoundid_IsoMS/myid_search_res/1481082226320-2016-12-6-8-43-46-858582/1481082226327-670-215.0566259-Neutral.htm) |
| 9 | 167.81 | 449.11502 | 451.12073 | 215.05670 | 2.00571 | 358969 | 1 | 1 | [1](http://www.mycompoundid.org/mycompoundid_IsoMS/myid_search_res/1481082226320-2016-12-6-8-43-46-858582/1481082226328-663-215.0567014-Neutral.htm) |
| 10 | 182.60 | 366.11259 | 368.11900 | 132.05427 | 2.00641 | 4930000 | 1 | 1 | [4](http://www.mycompoundid.org/mycompoundid_IsoMS/myid_search_res/1481082226320-2016-12-6-8-43-46-858582/1481082226329-67-132.054266-Neutral.htm) |
| 11 | 184.50 | 389.12732 | 391.13404 | 155.06900 | 2.00672 | 18850 | 1 | 1 | [1](http://www.mycompoundid.org/mycompoundid_IsoMS/myid_search_res/1481082226320-2016-12-6-8-43-46-858582/1481082226330-498-155.0690005-Neutral.htm) |
| 12 | 185.26 | 381.11133 | 383.11619 | 147.05301 | 2.00485 | 30650 | 1 | 1 | [6](http://www.mycompoundid.org/mycompoundid_IsoMS/myid_search_res/1481082226320-2016-12-6-8-43-46-858582/1481082226330-90-147.053014-Neutral.htm) |
| 13 | 197.89 | 389.12769 | 391.13316 | 155.06937 | 2.00546 | 14900 | 1 | 1 | [1](http://www.mycompoundid.org/mycompoundid_IsoMS/myid_search_res/1481082226320-2016-12-6-8-43-46-858582/1481082226332-945-155.0693748-Neutral.htm) |
| 14 | 200.90 | 359.07348 | 361.07845 | 125.01516 | 2.00497 | 150650 | 1 | 1 | [1](http://www.mycompoundid.org/mycompoundid_IsoMS/myid_search_res/1481082226320-2016-12-6-8-43-46-858582/1481082226332-242-125.0151573-Neutral.htm) |
| 15 | 211.31 | 389.12757 | 391.13309 | 155.06925 | 2.00552 | 23100 | 1 | 1 | [1](http://www.mycompoundid.org/mycompoundid_IsoMS/myid_search_res/1481082226320-2016-12-6-8-43-46-858582/1481082226334-137-155.0692523-Neutral.htm) |
| 16 | 212.64 | 380.12853 | 382.13487 | 146.07021 | 2.00634 | 7150000 | 1 | 1 | [4](http://www.mycompoundid.org/mycompoundid_IsoMS/myid_search_res/1481082226320-2016-12-6-8-43-46-858582/1481082226334-971-146.070205-Neutral.htm) |
| 17 | 214.39 | 517.15048 | 519.15673 | 283.09216 | 2.00626 | 57238 | 1 | 1 | [2](http://www.mycompoundid.org/mycompoundid_IsoMS/myid_search_res/1481082226320-2016-12-6-8-43-46-858582/1481082226335-99-283.0921552-Neutral.htm) |
| 18 | 215.35 | 359.07352 | 361.07922 | 125.01520 | 2.00569 | 257066 | 1 | 1 | [1](http://www.mycompoundid.org/mycompoundid_IsoMS/myid_search_res/1481082226320-2016-12-6-8-43-46-858582/1481082226336-630-125.0152037-Neutral.htm) |
| 19 | 220.65 | 339.10168 | 341.10819 | 105.04336 | 2.00652 | 947000 | 1 | 1 | [2](http://www.mycompoundid.org/mycompoundid_IsoMS/myid_search_res/1481082226320-2016-12-6-8-43-46-858582/1481082226336-511-105.043356-Neutral.htm) |
| 20 | 227.01 | 517.15056 | 519.15586 | 283.09224 | 2.00530 | 40500 | 1 | 1 | [2](http://www.mycompoundid.org/mycompoundid_IsoMS/myid_search_res/1481082226320-2016-12-6-8-43-46-858582/1481082226337-707-283.09224-Neutral.htm) |
| 21 | 230.26 | 365.11736 | 367.12365 | 131.05904 | 2.00628 | 3980000 | 1 | 1 | [8](http://www.mycompoundid.org/mycompoundid_IsoMS/myid_search_res/1481082226320-2016-12-6-8-43-46-858582/1481082226337-425-131.0590425-Neutral.htm) |
| # | RT (s) | mz_light | mz_heavy | mz | distance | int_light | nCharge | nTag | Possible hits |
| 22 | 239.27 | 517.15025 | 519.15680 | 283.09193 | 2.00655 | 123781 | 1 | 1 | [2](http://www.mycompoundid.org/mycompoundid_IsoMS/myid_search_res/1481082226320-2016-12-6-8-43-46-858582/1481082226339-829-283.0919288-Neutral.htm) |
| 23 | 241.67 | 408.17093 | 410.17733 | 174.11261 | 2.00640 | 422000 | 1 | 1 | [2](http://www.mycompoundid.org/mycompoundid_IsoMS/myid_search_res/1481082226320-2016-12-6-8-43-46-858582/1481082226339-693-174.112606-Neutral.htm) |
| 24 | 243.67 | 339.10156 | 341.10796 | 105.04324 | 2.00640 | 716000 | 1 | 1 | [2](http://www.mycompoundid.org/mycompoundid_IsoMS/myid_search_res/1481082226320-2016-12-6-8-43-46-858582/1481082226340-250-105.04324-Neutral.htm) |
| 25 | 245.84 | 293.10647 | 295.11258 | 59.04815 | 2.00611 | 36719 | 1 | 1 | [1](http://www.mycompoundid.org/mycompoundid_IsoMS/myid_search_res/1481082226320-2016-12-6-8-43-46-858582/1481082226340-885-59.04815006-Neutral.htm) |
| 26 | 245.96 | 380.12882 | 382.13501 | 146.07050 | 2.00619 | 1151250 | 1 | 1 | [4](http://www.mycompoundid.org/mycompoundid_IsoMS/myid_search_res/1481082226320-2016-12-6-8-43-46-858582/1481082226340-672-146.0704984-Neutral.htm) |
| 27 | 251.50 | 403.14269 | 405.14879 | 169.08437 | 2.00611 | 18700 | 1 | 1 | [2](http://www.mycompoundid.org/mycompoundid_IsoMS/myid_search_res/1481082226320-2016-12-6-8-43-46-858582/1481082226341-44-169.0843675-Neutral.htm) |
| 28 | 252.68 | 485.15814 | 487.16431 | 251.09982 | 2.00618 | 10600 | 1 | 1 | [3](http://www.mycompoundid.org/mycompoundid_IsoMS/myid_search_res/1481082226320-2016-12-6-8-43-46-858582/1481082226342-489-251.099815-Neutral.htm) |
| 29 | 253.98 | 397.12472 | 399.13073 | 163.06640 | 2.00602 | 70800 | 1 | 1 | [1](http://www.mycompoundid.org/mycompoundid_IsoMS/myid_search_res/1481082226320-2016-12-6-8-43-46-858582/1481082226342-17-163.066396-Neutral.htm) |
| 30 | 259.28 | 381.11129 | 383.11740 | 147.05297 | 2.00611 | 98400 | 1 | 1 | [6](http://www.mycompoundid.org/mycompoundid_IsoMS/myid_search_res/1481082226320-2016-12-6-8-43-46-858582/1481082226343-715-147.052967-Neutral.htm) |
| 31 | 261.28 | 424.11749 | 426.12373 | 190.05917 | 2.00624 | 12200 | 1 | 1 | [1](http://www.mycompoundid.org/mycompoundid_IsoMS/myid_search_res/1481082226320-2016-12-6-8-43-46-858582/1481082226344-426-190.059168-Neutral.htm) |
| 32 | 262.06 | 408.17053 | 410.17718 | 174.11221 | 2.00664 | 910375 | 1 | 1 | [2](http://www.mycompoundid.org/mycompoundid_IsoMS/myid_search_res/1481082226320-2016-12-6-8-43-46-858582/1481082226344-138-174.1122145-Neutral.htm) |
| 33 | 262.60 | 414.12169 | 416.12685 | 180.06337 | 2.00516 | 14375 | 1 | 1 | [17](http://www.mycompoundid.org/mycompoundid_IsoMS/myid_search_res/1481082226320-2016-12-6-8-43-46-858582/1481082226345-724-180.0633688-Neutral.htm) |
| 34 | 263.63 | 509.16934 | 511.17527 | 275.11102 | 2.00592 | 10886 | 1 | 1 | [2](http://www.mycompoundid.org/mycompoundid_IsoMS/myid_search_res/1481082226320-2016-12-6-8-43-46-858582/1481082226346-529-275.1110231-Neutral.htm) |
| 35 | 267.30 | 366.11246 | 368.11905 | 132.05414 | 2.00658 | 2732500 | 1 | 1 | [4](http://www.mycompoundid.org/mycompoundid_IsoMS/myid_search_res/1481082226320-2016-12-6-8-43-46-858582/1481082226346-568-132.0541443-Neutral.htm) |
| 36 | 270.46 | 502.13862 | 504.14406 | 268.08030 | 2.00544 | 48378 | 1 | 1 | [4](http://www.mycompoundid.org/mycompoundid_IsoMS/myid_search_res/1481082226320-2016-12-6-8-43-46-858582/1481082226347-522-268.0802963-Neutral.htm) |
| 37 | 274.87 | 414.12151 | 416.12753 | 180.06319 | 2.00602 | 25619 | 1 | 1 | [17](http://www.mycompoundid.org/mycompoundid_IsoMS/myid_search_res/1481082226320-2016-12-6-8-43-46-858582/1481082226349-683-180.063194-Neutral.htm) |
| 38 | 275.78 | 501.11490 | 505.12821 | 34.00556 | 4.01330 | 8610 | 1 | 2 | [1](http://www.mycompoundid.org/mycompoundid_IsoMS/myid_search_res/1481082226320-2016-12-6-8-43-46-858582/1481082226350-141-34.005562-Neutral.htm) |
| 39 | 278.70 | 365.11775 | 367.12414 | 131.05943 | 2.00639 | 5270000 | 1 | 1 | [8](http://www.mycompoundid.org/mycompoundid_IsoMS/myid_search_res/1481082226320-2016-12-6-8-43-46-858582/1481082226351-701-131.059432-Neutral.htm) |
| 40 | 288.82 | 502.13914 | 504.14539 | 268.08082 | 2.00625 | 67813 | 1 | 1 | [4](http://www.mycompoundid.org/mycompoundid_IsoMS/myid_search_res/1481082226320-2016-12-6-8-43-46-858582/1481082226352-964-268.0808221-Neutral.htm) |
| 41 | 294.79 | 380.12789 | 382.13457 | 146.06957 | 2.00668 | 8270000 | 1 | 1 | [4](http://www.mycompoundid.org/mycompoundid_IsoMS/myid_search_res/1481082226320-2016-12-6-8-43-46-858582/1481082226353-202-146.069574-Neutral.htm) |
| 42 | 303.29 | 422.20971 | 424.21525 | 188.15139 | 2.00555 | 12800 | 1 | 1 | [1](http://www.mycompoundid.org/mycompoundid_IsoMS/myid_search_res/1481082226320-2016-12-6-8-43-46-858582/1481082226354-40-188.151385-Neutral.htm) |
| 43 | 307.52 | 367.09617 | 369.10188 | 133.03785 | 2.00571 | 73800 | 1 | 1 | [3](http://www.mycompoundid.org/mycompoundid_IsoMS/myid_search_res/1481082226320-2016-12-6-8-43-46-858582/1481082226358-633-133.037849-Neutral.htm) |
| 44 | 308.56 | 399.10441 | 401.11003 | 165.04609 | 2.00562 | 87138 | 1 | 1 | [1](http://www.mycompoundid.org/mycompoundid_IsoMS/myid_search_res/1481082226320-2016-12-6-8-43-46-858582/1481082226358-230-165.0460868-Neutral.htm) |
| 45 | 313.95 | 353.11661 | 355.12308 | 119.05829 | 2.00647 | 17095 | 1 | 1 | [3](http://www.mycompoundid.org/mycompoundid_IsoMS/myid_search_res/1481082226320-2016-12-6-8-43-46-858582/1481082226359-518-119.0582949-Neutral.htm) |
| # | RT (s) | mz_light | mz_heavy | mz | distance | int_light | nCharge | nTag | Possible hits |
| 46 | 315.35 | 422.20975 | 424.21408 | 188.15143 | 2.00433 | 18800 | 1 | 1 | [1](http://www.mycompoundid.org/mycompoundid_IsoMS/myid_search_res/1481082226320-2016-12-6-8-43-46-858582/1481082226360-420-188.1514264-Neutral.htm) |
| 47 | 323.31 | 515.17349 | 517.17615 | 281.11517 | 2.00267 | 16400 | 1 | 1 | [4](http://www.mycompoundid.org/mycompoundid_IsoMS/myid_search_res/1481082226320-2016-12-6-8-43-46-858582/1481082226361-194-281.115166-Neutral.htm) |
| 48 | 324.66 | 399.10460 | 401.11050 | 165.04628 | 2.00591 | 108188 | 1 | 1 | [1](http://www.mycompoundid.org/mycompoundid_IsoMS/myid_search_res/1481082226320-2016-12-6-8-43-46-858582/1481082226361-18-165.0462789-Neutral.htm) |
| 49 | 328.26 | 353.11570 | 355.12276 | 119.05738 | 2.00706 | 29640 | 1 | 1 | [3](http://www.mycompoundid.org/mycompoundid_IsoMS/myid_search_res/1481082226320-2016-12-6-8-43-46-858582/1481082226363-380-119.0573849-Neutral.htm) |
| 50 | 329.51 | 436.20105 | 438.20708 | 202.14273 | 2.00603 | 152100 | 1 | 1 | [2](http://www.mycompoundid.org/mycompoundid_IsoMS/myid_search_res/1481082226320-2016-12-6-8-43-46-858582/1481082226364-348-202.1427264-Neutral.htm) |
| 51 | 334.98 | 339.10164 | 341.10806 | 105.04332 | 2.00642 | 4457328 | 1 | 1 | [2](http://www.mycompoundid.org/mycompoundid_IsoMS/myid_search_res/1481082226320-2016-12-6-8-43-46-858582/1481082226365-260-105.0433243-Neutral.htm) |
| 52 | 336.70 | 381.11222 | 383.11861 | 147.05390 | 2.00639 | 4248125 | 1 | 1 | [6](http://www.mycompoundid.org/mycompoundid_IsoMS/myid_search_res/1481082226320-2016-12-6-8-43-46-858582/1481082226366-589-147.0539015-Neutral.htm) |
| 53 | 341.05 | 485.15870 | 487.16574 | 251.10038 | 2.00704 | 5730 | 1 | 1 | [3](http://www.mycompoundid.org/mycompoundid_IsoMS/myid_search_res/1481082226320-2016-12-6-8-43-46-858582/1481082226367-862-251.10038-Neutral.htm) |
| 54 | 342.77 | 501.15562 | 503.16298 | 267.09730 | 2.00736 | 757001 | 1 | 1 | [3](http://www.mycompoundid.org/mycompoundid_IsoMS/myid_search_res/1481082226320-2016-12-6-8-43-46-858582/1481082226368-934-267.0972987-Neutral.htm) |
| 55 | 348.06 | 399.10454 | 401.11017 | 165.04622 | 2.00563 | 88742 | 1 | 1 | [1](http://www.mycompoundid.org/mycompoundid_IsoMS/myid_search_res/1481082226320-2016-12-6-8-43-46-858582/1481082226368-755-165.0462215-Neutral.htm) |
| 56 | 348.18 | 436.20196 | 438.20781 | 202.14364 | 2.00585 | 303438 | 1 | 1 | [2](http://www.mycompoundid.org/mycompoundid_IsoMS/myid_search_res/1481082226320-2016-12-6-8-43-46-858582/1481082226369-474-202.1436388-Neutral.htm) |
| 57 | 356.79 | 309.09095 | 311.09723 | 75.03263 | 2.00628 | 4960000 | 1 | 1 | [1](http://www.mycompoundid.org/mycompoundid_IsoMS/myid_search_res/1481082226320-2016-12-6-8-43-46-858582/1481082226370-644-75.032627-Neutral.htm) |
| 58 | 357.74 | 403.14387 | 405.14969 | 169.08555 | 2.00581 | 281438 | 1 | 1 | [2](http://www.mycompoundid.org/mycompoundid_IsoMS/myid_search_res/1481082226320-2016-12-6-8-43-46-858582/1481082226370-281-169.0855542-Neutral.htm) |
| 59 | 367.31 | 381.11247 | 383.11880 | 147.05415 | 2.00633 | 5609727 | 1 | 1 | [6](http://www.mycompoundid.org/mycompoundid_IsoMS/myid_search_res/1481082226320-2016-12-6-8-43-46-858582/1481082226440-646-147.0541468-Neutral.htm) |
| 60 | 374.81 | 365.11747 | 367.12385 | 131.05915 | 2.00639 | 4229414 | 1 | 1 | [8](http://www.mycompoundid.org/mycompoundid_IsoMS/myid_search_res/1481082226320-2016-12-6-8-43-46-858582/1481082226442-836-131.0591474-Neutral.htm) |
| 61 | 378.65 | 353.11761 | 355.12394 | 119.05929 | 2.00633 | 2992813 | 1 | 1 | [3](http://www.mycompoundid.org/mycompoundid_IsoMS/myid_search_res/1481082226320-2016-12-6-8-43-46-858582/1481082226443-522-119.0592869-Neutral.htm) |
| 62 | 381.46 | 348.10231 | 350.10893 | 114.04399 | 2.00662 | 725734 | 1 | 1 | [2](http://www.mycompoundid.org/mycompoundid_IsoMS/myid_search_res/1481082226320-2016-12-6-8-43-46-858582/1481082226444-955-114.0439913-Neutral.htm) |
| 63 | 388.10 | 462.16913 | 464.17576 | 228.11081 | 2.00663 | 12300 | 1 | 1 | [1](http://www.mycompoundid.org/mycompoundid_IsoMS/myid_search_res/1481082226320-2016-12-6-8-43-46-858582/1481082226444-601-228.110813-Neutral.htm) |
| 64 | 388.72 | 519.19061 | 521.19662 | 285.13229 | 2.00600 | 22688 | 1 | 1 | [1](http://www.mycompoundid.org/mycompoundid_IsoMS/myid_search_res/1481082226320-2016-12-6-8-43-46-858582/1481082226445-111-285.1322928-Neutral.htm) |
| 65 | 397.93 | 348.10208 | 350.10861 | 114.04376 | 2.00653 | 541836 | 1 | 1 | [2](http://www.mycompoundid.org/mycompoundid_IsoMS/myid_search_res/1481082226320-2016-12-6-8-43-46-858582/1481082226449-622-114.0437621-Neutral.htm) |
| 66 | 399.93 | 379.13280 | 381.13921 | 145.07448 | 2.00641 | 215625 | 1 | 1 | [6](http://www.mycompoundid.org/mycompoundid_IsoMS/myid_search_res/1481082226320-2016-12-6-8-43-46-858582/1481082226450-636-145.0744759-Neutral.htm) |
| 67 | 402.54 | 422.18553 | 424.19106 | 188.12721 | 2.00553 | 11240 | 1 | 1 | [1](http://www.mycompoundid.org/mycompoundid_IsoMS/myid_search_res/1481082226320-2016-12-6-8-43-46-858582/1481082226450-778-188.1272098-Neutral.htm) |
| 68 | 407.85 | 387.12216 | 389.12880 | 153.06384 | 2.00664 | 26000 | 1 | 1 | [1](http://www.mycompoundid.org/mycompoundid_IsoMS/myid_search_res/1481082226320-2016-12-6-8-43-46-858582/1481082226451-591-153.063837-Neutral.htm) |
| 69 | 408.69 | 519.19132 | 521.19627 | 285.13300 | 2.00495 | 21717 | 1 | 1 | [1](http://www.mycompoundid.org/mycompoundid_IsoMS/myid_search_res/1481082226320-2016-12-6-8-43-46-858582/1481082226452-970-285.1329982-Neutral.htm) |
| # | RT (s) | mz_light | mz_heavy | mz | distance | int_light | nCharge | nTag | Possible hits |
| 70 | 415.48 | 353.11755 | 355.12423 | 119.05923 | 2.00668 | 2729063 | 1 | 1 | [3](http://www.mycompoundid.org/mycompoundid_IsoMS/myid_search_res/1481082226320-2016-12-6-8-43-46-858582/1481082226453-694-119.0592332-Neutral.htm) |
| 71 | 421.02 | 295.11164 | 297.11822 | 61.05332 | 2.00658 | 5788438 | 1 | 1 | [1](http://www.mycompoundid.org/mycompoundid_IsoMS/myid_search_res/1481082226320-2016-12-6-8-43-46-858582/1481082226454-384-61.05331731-Neutral.htm) |
| 72 | 421.38 | 462.17009 | 464.17678 | 228.11177 | 2.00668 | 296900 | 1 | 1 | [1](http://www.mycompoundid.org/mycompoundid_IsoMS/myid_search_res/1481082226320-2016-12-6-8-43-46-858582/1481082226455-499-228.1117708-Neutral.htm) |
| 73 | 424.37 | 395.12736 | 397.13316 | 161.06904 | 2.00580 | 20125 | 1 | 1 | [1](http://www.mycompoundid.org/mycompoundid_IsoMS/myid_search_res/1481082226320-2016-12-6-8-43-46-858582/1481082226456-727-161.0690364-Neutral.htm) |
| 74 | 424.86 | 323.10599 | 325.11256 | 89.04767 | 2.00657 | 39700 | 1 | 1 | [4](http://www.mycompoundid.org/mycompoundid_IsoMS/myid_search_res/1481082226320-2016-12-6-8-43-46-858582/1481082226456-331-89.04767-Neutral.htm) |
| 75 | 445.30 | 362.11810 | 364.12644 | 128.05978 | 2.00834 | 1001738 | 1 | 1 | [1](http://www.mycompoundid.org/mycompoundid_IsoMS/myid_search_res/1481082226320-2016-12-6-8-43-46-858582/1481082226459-663-128.0597813-Neutral.htm) |
| 76 | 447.57 | 462.17023 | 464.17701 | 228.11191 | 2.00678 | 545286 | 1 | 1 | [1](http://www.mycompoundid.org/mycompoundid_IsoMS/myid_search_res/1481082226320-2016-12-6-8-43-46-858582/1481082226460-903-228.1119117-Neutral.htm) |
| 77 | 449.16 | 279.07998 | 281.08641 | 45.02166 | 2.00643 | 57400 | 1 | 1 | [1](http://www.mycompoundid.org/mycompoundid_IsoMS/myid_search_res/1481082226320-2016-12-6-8-43-46-858582/1481082226461-313-45.021662-Neutral.htm) |
| 78 | 452.34 | 323.10658 | 325.11269 | 89.04826 | 2.00611 | 208720 | 1 | 1 | [4](http://www.mycompoundid.org/mycompoundid_IsoMS/myid_search_res/1481082226320-2016-12-6-8-43-46-858582/1481082226462-62-89.04825863-Neutral.htm) |
| 79 | 453.95 | 350.15306 | 352.15991 | 116.09474 | 2.00684 | 269000 | 1 | 1 | [1](http://www.mycompoundid.org/mycompoundid_IsoMS/myid_search_res/1481082226320-2016-12-6-8-43-46-858582/1481082226462-796-116.094742-Neutral.htm) |
| 80 | 455.15 | 422.18630 | 424.19207 | 188.12798 | 2.00576 | 216875 | 1 | 1 | [1](http://www.mycompoundid.org/mycompoundid_IsoMS/myid_search_res/1481082226320-2016-12-6-8-43-46-858582/1481082226463-788-188.1279828-Neutral.htm) |
| 81 | 462.69 | 348.10128 | 350.10738 | 114.04296 | 2.00610 | 108031 | 1 | 1 | [2](http://www.mycompoundid.org/mycompoundid_IsoMS/myid_search_res/1481082226320-2016-12-6-8-43-46-858582/1481082226466-193-114.0429616-Neutral.htm) |
| 82 | 466.48 | 394.14419 | 396.15015 | 160.08587 | 2.00596 | 555063 | 1 | 1 | [1](http://www.mycompoundid.org/mycompoundid_IsoMS/myid_search_res/1481082226320-2016-12-6-8-43-46-858582/1481082226466-114-160.0858739-Neutral.htm) |
| 83 | 483.12 | 351.13712 | 353.14330 | 117.07880 | 2.00618 | 30788 | 1 | 1 | [5](http://www.mycompoundid.org/mycompoundid_IsoMS/myid_search_res/1481082226320-2016-12-6-8-43-46-858582/1481082226469-754-117.0788002-Neutral.htm) |
| 84 | 493.83 | 483.14478 | 485.14973 | 249.08646 | 2.00496 | 20163 | 1 | 1 | [2](http://www.mycompoundid.org/mycompoundid_IsoMS/myid_search_res/1481082226320-2016-12-6-8-43-46-858582/1481082226471-309-249.086458-Neutral.htm) |
| 85 | 504.20 | 406.14277 | 408.14745 | 172.08445 | 2.00468 | 28370 | 1 | 1 | [2](http://www.mycompoundid.org/mycompoundid_IsoMS/myid_search_res/1481082226320-2016-12-6-8-43-46-858582/1481082226473-59-172.084452-Neutral.htm) |
| 86 | 505.02 | 337.12174 | 339.12804 | 103.06342 | 2.00631 | 53450 | 1 | 1 | [8](http://www.mycompoundid.org/mycompoundid_IsoMS/myid_search_res/1481082226320-2016-12-6-8-43-46-858582/1481082226473-82-103.0634165-Neutral.htm) |
| 87 | 508.30 | 353.11728 | 355.12351 | 119.05896 | 2.00623 | 416250 | 1 | 1 | [3](http://www.mycompoundid.org/mycompoundid_IsoMS/myid_search_res/1481082226320-2016-12-6-8-43-46-858582/1481082226474-288-119.0589649-Neutral.htm) |
| 88 | 511.82 | 531.14922 | 533.15339 | 297.09090 | 2.00418 | 12890 | 1 | 1 | [1](http://www.mycompoundid.org/mycompoundid_IsoMS/myid_search_res/1481082226320-2016-12-6-8-43-46-858582/1481082226476-137-297.090895-Neutral.htm) |
| 89 | 516.31 | 395.12676 | 397.13366 | 161.06844 | 2.00690 | 13638 | 1 | 1 | [1](http://www.mycompoundid.org/mycompoundid_IsoMS/myid_search_res/1481082226320-2016-12-6-8-43-46-858582/1481082226479-124-161.0684375-Neutral.htm) |
| 90 | 526.08 | 406.14310 | 408.14897 | 172.08478 | 2.00587 | 42838 | 1 | 1 | [2](http://www.mycompoundid.org/mycompoundid_IsoMS/myid_search_res/1481082226320-2016-12-6-8-43-46-858582/1481082226480-417-172.084778-Neutral.htm) |
| 91 | 526.29 | 408.15879 | 410.16518 | 174.10047 | 2.00639 | 85094 | 1 | 1 | [1](http://www.mycompoundid.org/mycompoundid_IsoMS/myid_search_res/1481082226320-2016-12-6-8-43-46-858582/1481082226481-26-174.1004675-Neutral.htm) |
| 92 | 528.64 | 351.13765 | 353.14416 | 117.07933 | 2.00651 | 221750 | 1 | 1 | [5](http://www.mycompoundid.org/mycompoundid_IsoMS/myid_search_res/1481082226320-2016-12-6-8-43-46-858582/1481082226481-894-117.0793303-Neutral.htm) |
| 93 | 530.33 | 381.11275 | 383.11933 | 147.05443 | 2.00658 | 1034250 | 1 | 1 | [6](http://www.mycompoundid.org/mycompoundid_IsoMS/myid_search_res/1481082226320-2016-12-6-8-43-46-858582/1481082226482-430-147.0544251-Neutral.htm) |
| # | RT (s) | mz_light | mz_heavy | mz | distance | int_light | nCharge | nTag | Possible hits |
| 94 | 532.98 | 363.10161 | 365.10799 | 129.04329 | 2.00637 | 4520000 | 1 | 1 | [5](http://www.mycompoundid.org/mycompoundid_IsoMS/myid_search_res/1481082226320-2016-12-6-8-43-46-858582/1481082226483-597-129.043293-Neutral.htm) |
| 95 | 535.65 | 517.14990 | 519.15657 | 283.09158 | 2.00667 | 18878 | 1 | 1 | [2](http://www.mycompoundid.org/mycompoundid_IsoMS/myid_search_res/1481082226320-2016-12-6-8-43-46-858582/1481082226483-298-283.0915779-Neutral.htm) |
| 96 | 542.90 | 478.12954 | 480.13613 | 244.07122 | 2.00659 | 2737188 | 1 | 1 | [3](http://www.mycompoundid.org/mycompoundid_IsoMS/myid_search_res/1481082226320-2016-12-6-8-43-46-858582/1481082226485-288-244.0712195-Neutral.htm) |
| 97 | 543.40 | 366.10158 | 368.10735 | 132.04326 | 2.00578 | 128463 | 1 | 1 | [7](http://www.mycompoundid.org/mycompoundid_IsoMS/myid_search_res/1481082226320-2016-12-6-8-43-46-858582/1481082226485-404-132.0432552-Neutral.htm) |
| 98 | 547.62 | 457.08936 | 459.09627 | 446.06208 | 2.00691 | 26747 | 2 | 2 | [1](http://www.mycompoundid.org/mycompoundid_IsoMS/myid_search_res/1481082226320-2016-12-6-8-43-46-858582/1481082226486-304-446.062082-Neutral.htm) |
| 99 | 547.71 | 502.13779 | 504.14503 | 268.07947 | 2.00724 | 16053 | 1 | 1 | [4](http://www.mycompoundid.org/mycompoundid_IsoMS/myid_search_res/1481082226320-2016-12-6-8-43-46-858582/1481082226487-879-268.079466-Neutral.htm) |
| 100 | 547.94 | 385.10861 | 387.11500 | 151.05029 | 2.00639 | 732813 | 1 | 1 | [3](http://www.mycompoundid.org/mycompoundid_IsoMS/myid_search_res/1481082226320-2016-12-6-8-43-46-858582/1481082226487-366-151.0502903-Neutral.htm) |
| 101 | 553.31 | 344.10654 | 346.11273 | 110.04822 | 2.00619 | 56450 | 1 | 1 | [1](http://www.mycompoundid.org/mycompoundid_IsoMS/myid_search_res/1481082226320-2016-12-6-8-43-46-858582/1481082226489-73-110.0482187-Neutral.htm) |
| 102 | 556.07 | 363.10205 | 365.10843 | 129.04373 | 2.00638 | 6634063 | 1 | 1 | [5](http://www.mycompoundid.org/mycompoundid_IsoMS/myid_search_res/1481082226320-2016-12-6-8-43-46-858582/1481082226490-688-129.0437311-Neutral.htm) |
| 103 | 558.30 | 395.12784 | 397.13457 | 161.06952 | 2.00672 | 7980000 | 1 | 1 | [1](http://www.mycompoundid.org/mycompoundid_IsoMS/myid_search_res/1481082226320-2016-12-6-8-43-46-858582/1481082226490-5-161.069524-Neutral.htm) |
| 104 | 561.38 | 362.11731 | 364.12320 | 128.05899 | 2.00589 | 138625 | 1 | 1 | [1](http://www.mycompoundid.org/mycompoundid_IsoMS/myid_search_res/1481082226320-2016-12-6-8-43-46-858582/1481082226491-966-128.0589908-Neutral.htm) |
| 105 | 562.51 | 457.08994 | 459.09592 | 446.06323 | 2.00598 | 11015 | 2 | 2 | [1](http://www.mycompoundid.org/mycompoundid_IsoMS/myid_search_res/1481082226320-2016-12-6-8-43-46-858582/1481082226492-801-446.063234-Neutral.htm) |
| 106 | 565.43 | 309.12698 | 311.13281 | 75.06866 | 2.00583 | 35750 | 1 | 1 | [2](http://www.mycompoundid.org/mycompoundid_IsoMS/myid_search_res/1481082226320-2016-12-6-8-43-46-858582/1481082226493-103-75.06865906-Neutral.htm) |
| 107 | 574.29 | 321.09117 | 323.09805 | 87.03285 | 2.00688 | 24000 | 1 | 1 | [1](http://www.mycompoundid.org/mycompoundid_IsoMS/myid_search_res/1481082226320-2016-12-6-8-43-46-858582/1481082226494-745-87.032852-Neutral.htm) |
| 108 | 579.77 | 363.10184 | 365.10809 | 129.04352 | 2.00625 | 4109891 | 1 | 1 | [5](http://www.mycompoundid.org/mycompoundid_IsoMS/myid_search_res/1481082226320-2016-12-6-8-43-46-858582/1481082226495-6-129.0435195-Neutral.htm) |
| 109 | 588.15 | 436.20103 | 438.20623 | 202.14271 | 2.00520 | 94138 | 1 | 1 | [2](http://www.mycompoundid.org/mycompoundid_IsoMS/myid_search_res/1481082226320-2016-12-6-8-43-46-858582/1481082226497-528-202.142708-Neutral.htm) |
| 110 | 592.53 | 453.16876 | 455.17427 | 219.11044 | 2.00551 | 11355 | 1 | 1 | [2](http://www.mycompoundid.org/mycompoundid_IsoMS/myid_search_res/1481082226320-2016-12-6-8-43-46-858582/1481082226515-257-219.110439-Neutral.htm) |
| 111 | 594.61 | 363.10185 | 365.10838 | 129.04353 | 2.00652 | 6160379 | 1 | 1 | [5](http://www.mycompoundid.org/mycompoundid_IsoMS/myid_search_res/1481082226320-2016-12-6-8-43-46-858582/1481082226517-34-129.0435312-Neutral.htm) |
| 112 | 595.81 | 481.08373 | 483.08967 | 247.02541 | 2.00594 | 11970 | 1 | 1 | [1](http://www.mycompoundid.org/mycompoundid_IsoMS/myid_search_res/1481082226320-2016-12-6-8-43-46-858582/1481082226517-736-247.0254138-Neutral.htm) |
| 113 | 603.78 | 460.11871 | 462.12532 | 226.06039 | 2.00661 | 2648125 | 1 | 1 | [1](http://www.mycompoundid.org/mycompoundid_IsoMS/myid_search_res/1481082226320-2016-12-6-8-43-46-858582/1481082226520-252-226.0603891-Neutral.htm) |
| 114 | 607.40 | 337.12161 | 339.12866 | 103.06329 | 2.00705 | 30138 | 1 | 1 | [8](http://www.mycompoundid.org/mycompoundid_IsoMS/myid_search_res/1481082226320-2016-12-6-8-43-46-858582/1481082226521-339-103.0632903-Neutral.htm) |
| 115 | 608.53 | 379.13319 | 381.13957 | 145.07487 | 2.00638 | 4882500 | 1 | 1 | [6](http://www.mycompoundid.org/mycompoundid_IsoMS/myid_search_res/1481082226320-2016-12-6-8-43-46-858582/1481082226522-259-145.0748726-Neutral.htm) |
| 116 | 613.48 | 366.10172 | 368.10709 | 132.04340 | 2.00537 | 36813 | 1 | 1 | [7](http://www.mycompoundid.org/mycompoundid_IsoMS/myid_search_res/1481082226320-2016-12-6-8-43-46-858582/1481082226523-448-132.0434026-Neutral.htm) |
| 117 | 618.83 | 398.12742 | 400.13418 | 164.06910 | 2.00676 | 15704 | 1 | 1 | [6](http://www.mycompoundid.org/mycompoundid_IsoMS/myid_search_res/1481082226320-2016-12-6-8-43-46-858582/1481082226525-513-164.069096-Neutral.htm) |
| # | RT (s) | mz_light | mz_heavy | mz | distance | int_light | nCharge | nTag | Possible hits |
| 118 | 625.50 | 321.12682 | 323.13226 | 87.06850 | 2.00544 | 61700 | 1 | 1 | [1](http://www.mycompoundid.org/mycompoundid_IsoMS/myid_search_res/1481082226320-2016-12-6-8-43-46-858582/1481082226526-733-87.06849775-Neutral.htm) |
| 119 | 628.59 | 399.12352 | 401.13031 | 165.06520 | 2.00679 | 28125 | 1 | 1 | [4](http://www.mycompoundid.org/mycompoundid_IsoMS/myid_search_res/1481082226320-2016-12-6-8-43-46-858582/1481082226527-414-165.0651995-Neutral.htm) |
| 120 | 634.51 | 523.15941 | 525.16617 | 289.10109 | 2.00676 | 10650 | 1 | 1 | [1](http://www.mycompoundid.org/mycompoundid_IsoMS/myid_search_res/1481082226320-2016-12-6-8-43-46-858582/1481082226531-372-289.1010875-Neutral.htm) |
| 121 | 637.92 | 409.14260 | 411.14805 | 175.08428 | 2.00545 | 14850 | 1 | 1 | [1](http://www.mycompoundid.org/mycompoundid_IsoMS/myid_search_res/1481082226320-2016-12-6-8-43-46-858582/1481082226531-542-175.0842815-Neutral.htm) |
| 122 | 650.67 | 370.09800 | 372.10423 | 136.03968 | 2.00623 | 7773750 | 1 | 1 | [1](http://www.mycompoundid.org/mycompoundid_IsoMS/myid_search_res/1481082226320-2016-12-6-8-43-46-858582/1481082226535-319-136.0396762-Neutral.htm) |
| 123 | 651.38 | 428.13809 | 430.14467 | 194.07977 | 2.00658 | 6630 | 1 | 1 | [1](http://www.mycompoundid.org/mycompoundid_IsoMS/myid_search_res/1481082226320-2016-12-6-8-43-46-858582/1481082226535-424-194.079774-Neutral.htm) |
| 124 | 659.53 | 349.12263 | 351.12898 | 115.06431 | 2.00635 | 11411250 | 1 | 1 | [2](http://www.mycompoundid.org/mycompoundid_IsoMS/myid_search_res/1481082226320-2016-12-6-8-43-46-858582/1481082226537-998-115.0643133-Neutral.htm) |
| 125 | 661.17 | 477.16393 | 479.16971 | 243.10561 | 2.00578 | 2880000 | 1 | 1 | [1](http://www.mycompoundid.org/mycompoundid_IsoMS/myid_search_res/1481082226320-2016-12-6-8-43-46-858582/1481082226538-313-243.105612-Neutral.htm) |
| 126 | 661.65 | 629.21084 | 633.22356 | 162.10150 | 4.01272 | 46294 | 1 | 2 | [1](http://www.mycompoundid.org/mycompoundid_IsoMS/myid_search_res/1481082226320-2016-12-6-8-43-46-858582/1481082226538-54-162.101498-Neutral.htm) |
| 127 | 668.38 | 513.11465 | 517.13032 | 46.00531 | 4.01567 | 12400 | 1 | 2 | [1](http://www.mycompoundid.org/mycompoundid_IsoMS/myid_search_res/1481082226320-2016-12-6-8-43-46-858582/1481082226539-361-46.005306-Neutral.htm) |
| 128 | 680.64 | 380.16324 | 382.16945 | 146.10492 | 2.00621 | 23225 | 1 | 1 | [4](http://www.mycompoundid.org/mycompoundid_IsoMS/myid_search_res/1481082226320-2016-12-6-8-43-46-858582/1481082226544-712-146.1049215-Neutral.htm) |
| 129 | 681.13 | 447.10422 | 449.10983 | 426.09179 | 2.00562 | 97300 | 2 | 2 | [1](http://www.mycompoundid.org/mycompoundid_IsoMS/myid_search_res/1481082226320-2016-12-6-8-43-46-858582/1481082226544-428-426.09179-Neutral.htm) |
| 130 | 681.48 | 408.15890 | 410.16512 | 174.10058 | 2.00622 | 147438 | 1 | 1 | [1](http://www.mycompoundid.org/mycompoundid_IsoMS/myid_search_res/1481082226320-2016-12-6-8-43-46-858582/1481082226545-354-174.1005795-Neutral.htm) |
| 131 | 691.52 | 351.13804 | 353.14453 | 117.07972 | 2.00649 | 4883750 | 1 | 1 | [5](http://www.mycompoundid.org/mycompoundid_IsoMS/myid_search_res/1481082226320-2016-12-6-8-43-46-858582/1481082226549-766-117.07972-Neutral.htm) |
| 132 | 694.42 | 792.31510 | 794.32160 | 558.25678 | 2.00650 | 38200 | 1 | 1 | [1](http://www.mycompoundid.org/mycompoundid_IsoMS/myid_search_res/1481082226320-2016-12-6-8-43-46-858582/1481082226550-615-558.256784-Neutral.htm) |
| 133 | 696.70 | 337.12183 | 339.12738 | 103.06351 | 2.00555 | 71066 | 1 | 1 | [8](http://www.mycompoundid.org/mycompoundid_IsoMS/myid_search_res/1481082226320-2016-12-6-8-43-46-858582/1481082226550-676-103.0635144-Neutral.htm) |
| 134 | 701.98 | 383.11071 | 385.11669 | 149.05239 | 2.00598 | 3057500 | 1 | 1 | [1](http://www.mycompoundid.org/mycompoundid_IsoMS/myid_search_res/1481082226320-2016-12-6-8-43-46-858582/1481082226553-500-149.0523903-Neutral.htm) |
| 135 | 703.73 | 346.08617 | 348.09312 | 112.02785 | 2.00696 | 589969 | 1 | 1 | [1](http://www.mycompoundid.org/mycompoundid_IsoMS/myid_search_res/1481082226320-2016-12-6-8-43-46-858582/1481082226554-956-112.0278495-Neutral.htm) |
| 136 | 705.57 | 351.13814 | 353.14451 | 117.07982 | 2.00637 | 5506875 | 1 | 1 | [5](http://www.mycompoundid.org/mycompoundid_IsoMS/myid_search_res/1481082226320-2016-12-6-8-43-46-858582/1481082226567-439-117.0798171-Neutral.htm) |
| 137 | 713.83 | 321.09108 | 323.09733 | 87.03276 | 2.00625 | 99513 | 1 | 1 | [1](http://www.mycompoundid.org/mycompoundid_IsoMS/myid_search_res/1481082226320-2016-12-6-8-43-46-858582/1481082226571-154-87.03276263-Neutral.htm) |
| 138 | 722.45 | 660.14851 | 662.15682 | 426.09019 | 2.00831 | 6610 | 1 | 1 | [1](http://www.mycompoundid.org/mycompoundid_IsoMS/myid_search_res/1481082226320-2016-12-6-8-43-46-858582/1481082226574-800-426.090185-Neutral.htm) |
| 139 | 727.18 | 447.10405 | 449.11097 | 426.09146 | 2.00692 | 877000 | 2 | 2 | [1](http://www.mycompoundid.org/mycompoundid_IsoMS/myid_search_res/1481082226320-2016-12-6-8-43-46-858582/1481082226576-316-426.09146-Neutral.htm) |
| 140 | 728.14 | 377.10763 | 379.11261 | 286.09863 | 2.00498 | 6090 | 2 | 2 | [1](http://www.mycompoundid.org/mycompoundid_IsoMS/myid_search_res/1481082226320-2016-12-6-8-43-46-858582/1481082226577-443-286.098627-Neutral.htm) |
| 141 | 734.50 | 409.14398 | 411.15035 | 175.08566 | 2.00638 | 834750 | 1 | 1 | [1](http://www.mycompoundid.org/mycompoundid_IsoMS/myid_search_res/1481082226320-2016-12-6-8-43-46-858582/1481082226578-782-175.0856573-Neutral.htm) |
| # | RT (s) | mz_light | mz_heavy | mz | distance | int_light | nCharge | nTag | Possible hits |
| 142 | 736.07 | 508.18198 | 510.18851 | 274.12366 | 2.00652 | 139066 | 1 | 1 | [1](http://www.mycompoundid.org/mycompoundid_IsoMS/myid_search_res/1481082226320-2016-12-6-8-43-46-858582/1481082226581-336-274.1236649-Neutral.htm) |
| 143 | 737.72 | 422.17337 | 424.18035 | 188.11505 | 2.00698 | 27056 | 1 | 1 | [3](http://www.mycompoundid.org/mycompoundid_IsoMS/myid_search_res/1481082226320-2016-12-6-8-43-46-858582/1481082226582-525-188.1150483-Neutral.htm) |
| 144 | 740.86 | 395.12823 | 397.13450 | 161.06991 | 2.00627 | 423375 | 1 | 1 | [1](http://www.mycompoundid.org/mycompoundid_IsoMS/myid_search_res/1481082226320-2016-12-6-8-43-46-858582/1481082226584-988-161.0699113-Neutral.htm) |
| 145 | 741.29 | 447.10423 | 449.11084 | 426.09182 | 2.00661 | 337000 | 2 | 2 | [1](http://www.mycompoundid.org/mycompoundid_IsoMS/myid_search_res/1481082226320-2016-12-6-8-43-46-858582/1481082226585-461-426.0918238-Neutral.htm) |
| 146 | 747.39 | 438.14918 | 440.15526 | 204.09086 | 2.00608 | 369203 | 1 | 1 | [1](http://www.mycompoundid.org/mycompoundid_IsoMS/myid_search_res/1481082226320-2016-12-6-8-43-46-858582/1481082226587-452-204.0908567-Neutral.htm) |
| 147 | 748.48 | 629.21024 | 633.22259 | 162.10090 | 4.01235 | 28744 | 1 | 2 | [1](http://www.mycompoundid.org/mycompoundid_IsoMS/myid_search_res/1481082226320-2016-12-6-8-43-46-858582/1481082226588-20-162.1009012-Neutral.htm) |
| 148 | 754.58 | 508.18119 | 510.18854 | 274.12287 | 2.00735 | 16000 | 1 | 1 | [1](http://www.mycompoundid.org/mycompoundid_IsoMS/myid_search_res/1481082226320-2016-12-6-8-43-46-858582/1481082226590-828-274.122869-Neutral.htm) |
| 149 | 755.28 | 400.08638 | 402.09188 | 166.02806 | 2.00550 | 44934 | 1 | 1 | [3](http://www.mycompoundid.org/mycompoundid_IsoMS/myid_search_res/1481082226320-2016-12-6-8-43-46-858582/1481082226591-309-166.0280608-Neutral.htm) |
| 150 | 766.89 | 319.11190 | 321.11744 | 85.05358 | 2.00553 | 112100 | 1 | 1 | [1](http://www.mycompoundid.org/mycompoundid_IsoMS/myid_search_res/1481082226320-2016-12-6-8-43-46-858582/1481082226595-896-85.05358325-Neutral.htm) |
| 151 | 778.61 | 409.14393 | 411.15026 | 175.08561 | 2.00633 | 5070000 | 1 | 1 | [1](http://www.mycompoundid.org/mycompoundid_IsoMS/myid_search_res/1481082226320-2016-12-6-8-43-46-858582/1481082226598-950-175.085611-Neutral.htm) |
| 152 | 784.41 | 321.11600 | 323.12256 | 174.11536 | 2.00656 | 726929 | 2 | 2 | [1](http://www.mycompoundid.org/mycompoundid_IsoMS/myid_search_res/1481082226320-2016-12-6-8-43-46-858582/1481082226600-718-174.115361-Neutral.htm) |
| 153 | 784.70 | 426.12069 | 428.12700 | 192.06237 | 2.00631 | 30900 | 1 | 1 | [1](http://www.mycompoundid.org/mycompoundid_IsoMS/myid_search_res/1481082226320-2016-12-6-8-43-46-858582/1481082226601-127-192.0623665-Neutral.htm) |
| 154 | 792.72 | 448.19043 | 450.19675 | 214.13211 | 2.00632 | 14950 | 1 | 1 | [1](http://www.mycompoundid.org/mycompoundid_IsoMS/myid_search_res/1481082226320-2016-12-6-8-43-46-858582/1481082226603-666-214.132114-Neutral.htm) |
| 155 | 797.27 | 383.12959 | 385.13434 | 149.07127 | 2.00475 | 26300 | 1 | 1 | [4](http://www.mycompoundid.org/mycompoundid_IsoMS/myid_search_res/1481082226320-2016-12-6-8-43-46-858582/1481082226605-773-149.0712693-Neutral.htm) |
| 156 | 802.46 | 360.10231 | 362.11009 | 126.04399 | 2.00778 | 1398938 | 1 | 1 | [2](http://www.mycompoundid.org/mycompoundid_IsoMS/myid_search_res/1481082226320-2016-12-6-8-43-46-858582/1481082226606-134-126.0439872-Neutral.htm) |
| 157 | 805.99 | 371.63317 | 373.64010 | 275.14970 | 2.00693 | 24234 | 2 | 2 | [2](http://www.mycompoundid.org/mycompoundid_IsoMS/myid_search_res/1481082226320-2016-12-6-8-43-46-858582/1481082226607-587-275.1497017-Neutral.htm) |
| 158 | 807.82 | 399.13874 | 401.14507 | 165.08042 | 2.00633 | 6773984 | 1 | 1 | [4](http://www.mycompoundid.org/mycompoundid_IsoMS/myid_search_res/1481082226320-2016-12-6-8-43-46-858582/1481082226608-871-165.0804194-Neutral.htm) |
| 159 | 821.19 | 365.15396 | 367.16031 | 131.09564 | 2.00635 | 7231250 | 1 | 1 | [6](http://www.mycompoundid.org/mycompoundid_IsoMS/myid_search_res/1481082226320-2016-12-6-8-43-46-858582/1481082226610-276-131.0956365-Neutral.htm) |
| 160 | 829.88 | 377.11666 | 379.12301 | 143.05834 | 2.00635 | 101000 | 1 | 1 | [1](http://www.mycompoundid.org/mycompoundid_IsoMS/myid_search_res/1481082226320-2016-12-6-8-43-46-858582/1481082226612-849-143.058342-Neutral.htm) |
| 161 | 837.18 | 365.15381 | 367.16020 | 131.09549 | 2.00640 | 11103628 | 1 | 1 | [6](http://www.mycompoundid.org/mycompoundid_IsoMS/myid_search_res/1481082226320-2016-12-6-8-43-46-858582/1481082226613-988-131.0954869-Neutral.htm) |
| 162 | 861.13 | 307.11155 | 309.11857 | 73.05323 | 2.00702 | 486000 | 1 | 1 | [3](http://www.mycompoundid.org/mycompoundid_IsoMS/myid_search_res/1481082226320-2016-12-6-8-43-46-858582/1481082226617-906-73.0532285-Neutral.htm) |
| 163 | 862.62 | 397.12573 | 399.13122 | 163.06741 | 2.00550 | 15900 | 1 | 1 | [1](http://www.mycompoundid.org/mycompoundid_IsoMS/myid_search_res/1481082226320-2016-12-6-8-43-46-858582/1481082226618-111-163.067405-Neutral.htm) |
| 164 | 862.94 | 612.26765 | 616.28052 | 145.15831 | 4.01287 | 55600 | 1 | 2 | [1](http://www.mycompoundid.org/mycompoundid_IsoMS/myid_search_res/1481082226320-2016-12-6-8-43-46-858582/1481082226618-465-145.1583065-Neutral.htm) |
| 165 | 867.32 | 597.11651 | 599.12135 | 363.05819 | 2.00484 | 37600 | 1 | 1 | [2](http://www.mycompoundid.org/mycompoundid_IsoMS/myid_search_res/1481082226320-2016-12-6-8-43-46-858582/1481082226620-707-363.058187-Neutral.htm) |
| # | RT (s) | mz_light | mz_heavy | mz | distance | int_light | nCharge | nTag | Possible hits |
| 166 | 867.40 | 626.28276 | 630.29547 | 159.17342 | 4.01272 | 6790 | 1 | 2 | [1](http://www.mycompoundid.org/mycompoundid_IsoMS/myid_search_res/1481082226320-2016-12-6-8-43-46-858582/1481082226621-591-159.173418-Neutral.htm) |
| 167 | 867.75 | 322.07429 | 324.08036 | 88.01597 | 2.00607 | 19650 | 1 | 1 | [2](http://www.mycompoundid.org/mycompoundid_IsoMS/myid_search_res/1481082226320-2016-12-6-8-43-46-858582/1481082226621-9-88.015965-Neutral.htm) |
| 168 | 871.61 | 300.08643 | 302.09315 | 132.05623 | 2.00671 | 9500 | 2 | 2 | [1](http://www.mycompoundid.org/mycompoundid_IsoMS/myid_search_res/1481082226320-2016-12-6-8-43-46-858582/1481082226622-722-132.056228-Neutral.htm) |
| 169 | 872.61 | 309.58359 | 311.58829 | 151.05055 | 2.00469 | 13000 | 2 | 2 | [3](http://www.mycompoundid.org/mycompoundid_IsoMS/myid_search_res/1481082226320-2016-12-6-8-43-46-858582/1481082226622-771-151.050546-Neutral.htm) |
| 170 | 878.84 | 345.09318 | 347.10010 | 222.06971 | 2.00692 | 37000 | 2 | 2 | [1](http://www.mycompoundid.org/mycompoundid_IsoMS/myid_search_res/1481082226320-2016-12-6-8-43-46-858582/1481082226625-908-222.0697129-Neutral.htm) |
| 171 | 883.24 | 555.15155 | 557.15731 | 321.09323 | 2.00576 | 231500 | 1 | 1 | [1](http://www.mycompoundid.org/mycompoundid_IsoMS/myid_search_res/1481082226320-2016-12-6-8-43-46-858582/1481082226626-46-321.093229-Neutral.htm) |
| 172 | 888.22 | 416.11709 | 418.12230 | 182.05877 | 2.00521 | 12350 | 1 | 1 | [6](http://www.mycompoundid.org/mycompoundid_IsoMS/myid_search_res/1481082226320-2016-12-6-8-43-46-858582/1481082226627-37-182.0587673-Neutral.htm) |
| 173 | 892.63 | 612.26689 | 616.27972 | 145.15755 | 4.01283 | 15275 | 1 | 2 | [1](http://www.mycompoundid.org/mycompoundid_IsoMS/myid_search_res/1481082226320-2016-12-6-8-43-46-858582/1481082226628-776-145.1575525-Neutral.htm) |
| 174 | 892.89 | 629.21068 | 633.22377 | 162.10134 | 4.01309 | 43625 | 1 | 2 | [1](http://www.mycompoundid.org/mycompoundid_IsoMS/myid_search_res/1481082226320-2016-12-6-8-43-46-858582/1481082226628-924-162.1013438-Neutral.htm) |
| 175 | 893.58 | 462.20597 | 464.21145 | 228.14765 | 2.00548 | 21413 | 1 | 1 | [2](http://www.mycompoundid.org/mycompoundid_IsoMS/myid_search_res/1481082226320-2016-12-6-8-43-46-858582/1481082226629-174-228.1476499-Neutral.htm) |
| 176 | 902.75 | 354.07128 | 356.07781 | 240.02593 | 2.00653 | 182000 | 2 | 2 | [1](http://www.mycompoundid.org/mycompoundid_IsoMS/myid_search_res/1481082226320-2016-12-6-8-43-46-858582/1481082226632-751-240.0259275-Neutral.htm) |
| 177 | 905.49 | 387.10050 | 389.10642 | 153.04218 | 2.00592 | 48838 | 1 | 1 | [2](http://www.mycompoundid.org/mycompoundid_IsoMS/myid_search_res/1481082226320-2016-12-6-8-43-46-858582/1481082226633-26-153.0421832-Neutral.htm) |
| 178 | 905.98 | 363.13816 | 365.14459 | 129.07984 | 2.00643 | 3792500 | 1 | 1 | [4](http://www.mycompoundid.org/mycompoundid_IsoMS/myid_search_res/1481082226320-2016-12-6-8-43-46-858582/1481082226634-930-129.0798373-Neutral.htm) |
| 179 | 911.64 | 612.26781 | 616.28023 | 145.15847 | 4.01241 | 50188 | 1 | 2 | [1](http://www.mycompoundid.org/mycompoundid_IsoMS/myid_search_res/1481082226320-2016-12-6-8-43-46-858582/1481082226635-434-145.1584748-Neutral.htm) |
| 180 | 927.44 | 502.09665 | 504.10388 | 268.03833 | 2.00723 | 19863 | 1 | 1 | [1](http://www.mycompoundid.org/mycompoundid_IsoMS/myid_search_res/1481082226320-2016-12-6-8-43-46-858582/1481082226638-438-268.0383318-Neutral.htm) |
| 181 | 928.09 | 496.18981 | 498.19654 | 262.13149 | 2.00673 | 18156 | 1 | 1 | [2](http://www.mycompoundid.org/mycompoundid_IsoMS/myid_search_res/1481082226320-2016-12-6-8-43-46-858582/1481082226639-402-262.1314868-Neutral.htm) |
| 182 | 929.06 | 393.14769 | 395.15427 | 159.08937 | 2.00658 | 33403 | 1 | 1 | [6](http://www.mycompoundid.org/mycompoundid_IsoMS/myid_search_res/1481082226320-2016-12-6-8-43-46-858582/1481082226639-235-159.0893706-Neutral.htm) |
| 183 | 931.97 | 431.12581 | 433.13110 | 197.06749 | 2.00529 | 20250 | 1 | 1 | [2](http://www.mycompoundid.org/mycompoundid_IsoMS/myid_search_res/1481082226320-2016-12-6-8-43-46-858582/1481082226640-84-197.067485-Neutral.htm) |
| 184 | 938.40 | 411.10457 | 413.11191 | 177.04625 | 2.00734 | 8470 | 1 | 1 | [1](http://www.mycompoundid.org/mycompoundid_IsoMS/myid_search_res/1481082226320-2016-12-6-8-43-46-858582/1481082226641-442-177.046254-Neutral.htm) |
| 185 | 940.82 | 502.09687 | 504.10423 | 268.03855 | 2.00736 | 17363 | 1 | 1 | [1](http://www.mycompoundid.org/mycompoundid_IsoMS/myid_search_res/1481082226320-2016-12-6-8-43-46-858582/1481082226642-166-268.0385485-Neutral.htm) |
| 186 | 948.03 | 612.26605 | 616.27923 | 145.15671 | 4.01318 | 11100 | 1 | 2 | [1](http://www.mycompoundid.org/mycompoundid_IsoMS/myid_search_res/1481082226320-2016-12-6-8-43-46-858582/1481082226643-496-145.1567098-Neutral.htm) |
| 187 | 949.91 | 351.13795 | 353.14454 | 117.07963 | 2.00659 | 612125 | 1 | 1 | [5](http://www.mycompoundid.org/mycompoundid_IsoMS/myid_search_res/1481082226320-2016-12-6-8-43-46-858582/1481082226645-154-117.0796295-Neutral.htm) |
| 188 | 957.72 | 350.64614 | 352.65257 | 233.17564 | 2.00643 | 42431 | 2 | 2 | [1](http://www.mycompoundid.org/mycompoundid_IsoMS/myid_search_res/1481082226320-2016-12-6-8-43-46-858582/1481082226648-179-233.1756425-Neutral.htm) |
| 189 | 966.82 | 501.11493 | 505.12830 | 34.00559 | 4.01336 | 60559 | 1 | 2 | [1](http://www.mycompoundid.org/mycompoundid_IsoMS/myid_search_res/1481082226320-2016-12-6-8-43-46-858582/1481082226652-116-34.00559384-Neutral.htm) |
| # | RT (s) | mz_light | mz_heavy | mz | distance | int_light | nCharge | nTag | Possible hits |
| 190 | 966.88 | 368.09964 | 370.10638 | 268.08265 | 2.00673 | 61681 | 2 | 2 | [3](http://www.mycompoundid.org/mycompoundid_IsoMS/myid_search_res/1481082226320-2016-12-6-8-43-46-858582/1481082226652-760-268.0826471-Neutral.htm) |
| 191 | 967.86 | 413.15322 | 415.16196 | 179.09490 | 2.00874 | 26650 | 1 | 1 | [2](http://www.mycompoundid.org/mycompoundid_IsoMS/myid_search_res/1481082226320-2016-12-6-8-43-46-858582/1481082226654-715-179.094899-Neutral.htm) |
| 192 | 971.73 | 311.59287 | 313.59969 | 155.06910 | 2.00682 | 77994 | 2 | 2 | [1](http://www.mycompoundid.org/mycompoundid_IsoMS/myid_search_res/1481082226320-2016-12-6-8-43-46-858582/1481082226656-744-155.0691033-Neutral.htm) |
| 193 | 975.74 | 371.63248 | 373.63850 | 275.14832 | 2.00602 | 10238 | 2 | 2 | [2](http://www.mycompoundid.org/mycompoundid_IsoMS/myid_search_res/1481082226320-2016-12-6-8-43-46-858582/1481082226657-111-275.148322-Neutral.htm) |
| 194 | 977.06 | 342.63030 | 344.63687 | 217.14397 | 2.00657 | 14375 | 2 | 2 | [1](http://www.mycompoundid.org/mycompoundid_IsoMS/myid_search_res/1481082226320-2016-12-6-8-43-46-858582/1481082226658-993-217.143967-Neutral.htm) |
| 195 | 980.38 | 346.09891 | 348.10579 | 224.08118 | 2.00688 | 10500 | 2 | 2 | [3](http://www.mycompoundid.org/mycompoundid_IsoMS/myid_search_res/1481082226320-2016-12-6-8-43-46-858582/1481082226659-758-224.08118-Neutral.htm) |
| 196 | 980.97 | 300.10391 | 302.11049 | 132.09119 | 2.00657 | 40300 | 2 | 2 | [2](http://www.mycompoundid.org/mycompoundid_IsoMS/myid_search_res/1481082226320-2016-12-6-8-43-46-858582/1481082226660-940-132.091186-Neutral.htm) |
| 197 | 987.45 | 494.68875 | 496.69480 | 521.26086 | 2.00605 | 6890 | 2 | 2 | [1](http://www.mycompoundid.org/mycompoundid_IsoMS/myid_search_res/1481082226320-2016-12-6-8-43-46-858582/1481082226661-359-521.260862-Neutral.htm) |
| 198 | 1004.84 | 407.16383 | 409.17006 | 173.10551 | 2.00623 | 44875 | 1 | 1 | [4](http://www.mycompoundid.org/mycompoundid_IsoMS/myid_search_res/1481082226320-2016-12-6-8-43-46-858582/1481082226665-363-173.1055071-Neutral.htm) |
| 199 | 1005.29 | 379.16895 | 381.17392 | 145.11063 | 2.00498 | 26941 | 1 | 1 | [1](http://www.mycompoundid.org/mycompoundid_IsoMS/myid_search_res/1481082226320-2016-12-6-8-43-46-858582/1481082226665-496-145.1106264-Neutral.htm) |
| 200 | 1006.92 | 372.09032 | 374.09661 | 138.03200 | 2.00629 | 106109 | 1 | 1 | [4](http://www.mycompoundid.org/mycompoundid_IsoMS/myid_search_res/1481082226320-2016-12-6-8-43-46-858582/1481082226666-450-138.0320001-Neutral.htm) |
| 201 | 1014.64 | 441.14752 | 443.15374 | 207.08920 | 2.00623 | 35781 | 1 | 1 | [3](http://www.mycompoundid.org/mycompoundid_IsoMS/myid_search_res/1481082226320-2016-12-6-8-43-46-858582/1481082226667-125-207.089195-Neutral.htm) |
| 202 | 1016.48 | 427.13226 | 429.13809 | 193.07394 | 2.00583 | 18200 | 1 | 1 | [3](http://www.mycompoundid.org/mycompoundid_IsoMS/myid_search_res/1481082226320-2016-12-6-8-43-46-858582/1481082226667-472-193.073941-Neutral.htm) |
| 203 | 1019.82 | 312.08485 | 314.09115 | 156.05306 | 2.00630 | 839000 | 2 | 2 | [3](http://www.mycompoundid.org/mycompoundid_IsoMS/myid_search_res/1481082226320-2016-12-6-8-43-46-858582/1481082226668-942-156.0530585-Neutral.htm) |
| 204 | 1026.08 | 457.14136 | 459.14810 | 223.08304 | 2.00675 | 15000 | 1 | 1 | [1](http://www.mycompoundid.org/mycompoundid_IsoMS/myid_search_res/1481082226320-2016-12-6-8-43-46-858582/1481082226670-828-223.083035-Neutral.htm) |
| 205 | 1028.54 | 407.16419 | 409.17041 | 173.10587 | 2.00622 | 146750 | 1 | 1 | [4](http://www.mycompoundid.org/mycompoundid_IsoMS/myid_search_res/1481082226320-2016-12-6-8-43-46-858582/1481082226671-489-173.1058696-Neutral.htm) |
| 206 | 1034.98 | 413.15418 | 415.16000 | 179.09586 | 2.00581 | 573781 | 1 | 1 | [2](http://www.mycompoundid.org/mycompoundid_IsoMS/myid_search_res/1481082226320-2016-12-6-8-43-46-858582/1481082226672-546-179.0958644-Neutral.htm) |
| 207 | 1037.61 | 347.11234 | 349.11957 | 226.10804 | 2.00724 | 13122 | 2 | 2 | [1](http://www.mycompoundid.org/mycompoundid_IsoMS/myid_search_res/1481082226320-2016-12-6-8-43-46-858582/1481082226672-656-226.1080381-Neutral.htm) |
| 208 | 1039.57 | 367.60760 | 369.61494 | 267.09855 | 2.00734 | 318000 | 2 | 2 | [2](http://www.mycompoundid.org/mycompoundid_IsoMS/myid_search_res/1481082226320-2016-12-6-8-43-46-858582/1481082226673-502-267.098552-Neutral.htm) |
| 209 | 1042.57 | 356.09962 | 358.10434 | 244.08259 | 2.00472 | 7290 | 2 | 2 | [1](http://www.mycompoundid.org/mycompoundid_IsoMS/myid_search_res/1481082226320-2016-12-6-8-43-46-858582/1481082226688-888-244.082594-Neutral.htm) |
| 210 | 1047.28 | 375.60520 | 377.61174 | 283.09376 | 2.00654 | 88753 | 2 | 2 | [2](http://www.mycompoundid.org/mycompoundid_IsoMS/myid_search_res/1481082226320-2016-12-6-8-43-46-858582/1481082226689-68-283.0937638-Neutral.htm) |
| 211 | 1048.79 | 366.10141 | 368.10676 | 132.04309 | 2.00535 | 34500 | 1 | 1 | [7](http://www.mycompoundid.org/mycompoundid_IsoMS/myid_search_res/1481082226320-2016-12-6-8-43-46-858582/1481082226690-431-132.043086-Neutral.htm) |
| 212 | 1063.17 | 379.16995 | 381.17633 | 145.11163 | 2.00639 | 2143750 | 1 | 1 | [1](http://www.mycompoundid.org/mycompoundid_IsoMS/myid_search_res/1481082226320-2016-12-6-8-43-46-858582/1481082226694-722-145.1116256-Neutral.htm) |
| 213 | 1066.14 | 389.12813 | 391.13395 | 155.06981 | 2.00582 | 90838 | 1 | 1 | [1](http://www.mycompoundid.org/mycompoundid_IsoMS/myid_search_res/1481082226320-2016-12-6-8-43-46-858582/1481082226695-251-155.0698063-Neutral.htm) |
| # | RT (s) | mz_light | mz_heavy | mz | distance | int_light | nCharge | nTag | Possible hits |
| 214 | 1067.29 | 312.59492 | 314.60143 | 157.07320 | 2.00651 | 563481 | 2 | 2 | [2](http://www.mycompoundid.org/mycompoundid_IsoMS/myid_search_res/1481082226320-2016-12-6-8-43-46-858582/1481082226696-630-157.0732031-Neutral.htm) |
| 215 | 1070.56 | 339.09654 | 341.10372 | 210.07643 | 2.00718 | 11269 | 2 | 2 | [1](http://www.mycompoundid.org/mycompoundid_IsoMS/myid_search_res/1481082226320-2016-12-6-8-43-46-858582/1481082226697-288-210.0764318-Neutral.htm) |
| 216 | 1078.28 | 356.09410 | 358.10140 | 244.07156 | 2.00729 | 28069 | 2 | 2 | [3](http://www.mycompoundid.org/mycompoundid_IsoMS/myid_search_res/1481082226320-2016-12-6-8-43-46-858582/1481082226698-530-244.071564-Neutral.htm) |
| 217 | 1086.58 | 382.10946 | 384.11536 | 296.10227 | 2.00590 | 7533 | 2 | 2 | [1](http://www.mycompoundid.org/mycompoundid_IsoMS/myid_search_res/1481082226320-2016-12-6-8-43-46-858582/1481082226699-347-296.102271-Neutral.htm) |
| 218 | 1093.63 | 389.11939 | 391.12549 | 310.12215 | 2.00609 | 6790 | 2 | 2 | [1](http://www.mycompoundid.org/mycompoundid_IsoMS/myid_search_res/1481082226320-2016-12-6-8-43-46-858582/1481082226700-102-310.122148-Neutral.htm) |
| 219 | 1097.31 | 487.15238 | 489.15885 | 253.09406 | 2.00647 | 16150 | 1 | 1 | [1](http://www.mycompoundid.org/mycompoundid_IsoMS/myid_search_res/1481082226320-2016-12-6-8-43-46-858582/1481082226702-499-253.0940568-Neutral.htm) |
| 220 | 1098.67 | 541.14297 | 543.14710 | 307.08465 | 2.00413 | 9710 | 1 | 1 | [1](http://www.mycompoundid.org/mycompoundid_IsoMS/myid_search_res/1481082226320-2016-12-6-8-43-46-858582/1481082226703-702-307.0846515-Neutral.htm) |
| 221 | 1100.11 | 356.09358 | 358.10081 | 244.07053 | 2.00723 | 40294 | 2 | 2 | [2](http://www.mycompoundid.org/mycompoundid_IsoMS/myid_search_res/1481082226320-2016-12-6-8-43-46-858582/1481082226703-558-244.070525-Neutral.htm) |
| 222 | 1100.38 | 298.08804 | 300.09521 | 128.05945 | 2.00717 | 538375 | 2 | 2 | [1](http://www.mycompoundid.org/mycompoundid_IsoMS/myid_search_res/1481082226320-2016-12-6-8-43-46-858582/1481082226704-972-128.0594455-Neutral.htm) |
| 223 | 1109.55 | 466.17945 | 468.18461 | 232.12113 | 2.00516 | 11031 | 1 | 1 | [1](http://www.mycompoundid.org/mycompoundid_IsoMS/myid_search_res/1481082226320-2016-12-6-8-43-46-858582/1481082226706-225-232.1211274-Neutral.htm) |
| 224 | 1110.73 | 356.09422 | 358.10090 | 244.07180 | 2.00668 | 248125 | 2 | 2 | [3](http://www.mycompoundid.org/mycompoundid_IsoMS/myid_search_res/1481082226320-2016-12-6-8-43-46-858582/1481082226706-976-244.0717966-Neutral.htm) |
| 225 | 1117.69 | 328.13365 | 330.14152 | 188.15066 | 2.00786 | 9650 | 2 | 2 | [1](http://www.mycompoundid.org/mycompoundid_IsoMS/myid_search_res/1481082226320-2016-12-6-8-43-46-858582/1481082226707-799-188.150663-Neutral.htm) |
| 226 | 1124.66 | 423.14240 | 425.14653 | 189.08408 | 2.00414 | 8380 | 1 | 1 | [1](http://www.mycompoundid.org/mycompoundid_IsoMS/myid_search_res/1481082226320-2016-12-6-8-43-46-858582/1481082226708-728-189.084075-Neutral.htm) |
| 227 | 1130.88 | 348.11413 | 350.12264 | 228.11163 | 2.00850 | 32100 | 2 | 2 | [1](http://www.mycompoundid.org/mycompoundid_IsoMS/myid_search_res/1481082226320-2016-12-6-8-43-46-858582/1481082226710-611-228.111628-Neutral.htm) |
| 228 | 1134.33 | 328.11714 | 330.12356 | 188.11764 | 2.00643 | 57016 | 2 | 2 | [3](http://www.mycompoundid.org/mycompoundid_IsoMS/myid_search_res/1481082226320-2016-12-6-8-43-46-858582/1481082226710-785-188.1176385-Neutral.htm) |
| 229 | 1140.53 | 386.10673 | 388.11380 | 152.04841 | 2.00707 | 8720 | 1 | 1 | [9](http://www.mycompoundid.org/mycompoundid_IsoMS/myid_search_res/1481082226320-2016-12-6-8-43-46-858582/1481082226711-869-152.04841-Neutral.htm) |
| 230 | 1143.63 | 528.16246 | 532.17549 | 61.05312 | 4.01303 | 52644 | 1 | 2 | [1](http://www.mycompoundid.org/mycompoundid_IsoMS/myid_search_res/1481082226320-2016-12-6-8-43-46-858582/1481082226712-779-61.05311956-Neutral.htm) |
| 231 | 1145.62 | 321.10926 | 323.11535 | 174.10188 | 2.00609 | 35200 | 2 | 2 | [1](http://www.mycompoundid.org/mycompoundid_IsoMS/myid_search_res/1481082226320-2016-12-6-8-43-46-858582/1481082226713-483-174.101876-Neutral.htm) |
| 232 | 1153.10 | 292.10594 | 294.11229 | 116.09524 | 2.00635 | 41381 | 2 | 2 | [1](http://www.mycompoundid.org/mycompoundid_IsoMS/myid_search_res/1481082226320-2016-12-6-8-43-46-858582/1481082226714-529-116.0952381-Neutral.htm) |
| 233 | 1153.63 | 379.16944 | 381.17540 | 145.11112 | 2.00596 | 379156 | 1 | 1 | [1](http://www.mycompoundid.org/mycompoundid_IsoMS/myid_search_res/1481082226320-2016-12-6-8-43-46-858582/1481082226714-601-145.1111201-Neutral.htm) |
| 234 | 1183.58 | 693.16709 | 697.18175 | 226.05775 | 4.01466 | 12700 | 1 | 2 | [1](http://www.mycompoundid.org/mycompoundid_IsoMS/myid_search_res/1481082226320-2016-12-6-8-43-46-858582/1481082226718-582-226.057751-Neutral.htm) |
| 235 | 1187.80 | 346.08548 | 348.09120 | 112.02716 | 2.00572 | 32000 | 1 | 1 | [1](http://www.mycompoundid.org/mycompoundid_IsoMS/myid_search_res/1481082226320-2016-12-6-8-43-46-858582/1481082226719-437-112.0271575-Neutral.htm) |
| 236 | 1196.40 | 427.16945 | 429.17523 | 193.11113 | 2.00578 | 371219 | 1 | 1 | [2](http://www.mycompoundid.org/mycompoundid_IsoMS/myid_search_res/1481082226320-2016-12-6-8-43-46-858582/1481082226720-523-193.1111291-Neutral.htm) |
| 237 | 1199.57 | 335.16715 | 337.17351 | 202.21767 | 2.00635 | 20450 | 2 | 2 | [1](http://www.mycompoundid.org/mycompoundid_IsoMS/myid_search_res/1481082226320-2016-12-6-8-43-46-858582/1481082226721-579-202.2176685-Neutral.htm) |
| # | RT (s) | mz_light | mz_heavy | mz | distance | int_light | nCharge | nTag | Possible hits |
| 238 | 1215.62 | 403.14390 | 405.15022 | 169.08558 | 2.00633 | 11850 | 1 | 1 | [2](http://www.mycompoundid.org/mycompoundid_IsoMS/myid_search_res/1481082226320-2016-12-6-8-43-46-858582/1481082226723-413-169.085575-Neutral.htm) |
| 239 | 1225.89 | 325.09802 | 327.10475 | 182.07941 | 2.00673 | 661500 | 2 | 2 | [4](http://www.mycompoundid.org/mycompoundid_IsoMS/myid_search_res/1481082226320-2016-12-6-8-43-46-858582/1481082226726-324-182.079408-Neutral.htm) |
| 240 | 1227.31 | 414.12389 | 416.13048 | 180.06557 | 2.00658 | 35853 | 1 | 1 | [3](http://www.mycompoundid.org/mycompoundid_IsoMS/myid_search_res/1481082226320-2016-12-6-8-43-46-858582/1481082226726-200-180.0655725-Neutral.htm) |
| 241 | 1229.02 | 373.08570 | 375.09071 | 139.02738 | 2.00501 | 15050 | 1 | 1 | [2](http://www.mycompoundid.org/mycompoundid_IsoMS/myid_search_res/1481082226320-2016-12-6-8-43-46-858582/1481082226727-338-139.027379-Neutral.htm) |
| 242 | 1229.78 | 386.10619 | 388.11146 | 152.04787 | 2.00527 | 26500 | 1 | 1 | [9](http://www.mycompoundid.org/mycompoundid_IsoMS/myid_search_res/1481082226320-2016-12-6-8-43-46-858582/1481082226729-577-152.047867-Neutral.htm) |
| 243 | 1263.07 | 437.17342 | 439.18081 | 203.11510 | 2.00739 | 30275 | 1 | 1 | [1](http://www.mycompoundid.org/mycompoundid_IsoMS/myid_search_res/1481082226320-2016-12-6-8-43-46-858582/1481082226735-823-203.1151035-Neutral.htm) |
| 244 | 1269.01 | 373.12257 | 375.12872 | 278.12850 | 2.00615 | 17950 | 2 | 2 | [3](http://www.mycompoundid.org/mycompoundid_IsoMS/myid_search_res/1481082226320-2016-12-6-8-43-46-858582/1481082226735-424-278.1285035-Neutral.htm) |
| 245 | 1284.23 | 598.10105 | 600.10475 | 364.04273 | 2.00370 | 11340 | 1 | 1 | [1](http://www.mycompoundid.org/mycompoundid_IsoMS/myid_search_res/1481082226320-2016-12-6-8-43-46-858582/1481082226737-258-364.0427295-Neutral.htm) |
| 246 | 1298.78 | 302.07811 | 304.08615 | 136.03958 | 2.00803 | 6970 | 2 | 2 | [1](http://www.mycompoundid.org/mycompoundid_IsoMS/myid_search_res/1481082226320-2016-12-6-8-43-46-858582/1481082226739-953-136.039582-Neutral.htm) |
| 247 | 1306.20 | 435.19433 | 437.20016 | 201.13601 | 2.00583 | 23763 | 1 | 1 | [1](http://www.mycompoundid.org/mycompoundid_IsoMS/myid_search_res/1481082226320-2016-12-6-8-43-46-858582/1481082226741-655-201.1360106-Neutral.htm) |
| 248 | 1308.32 | 354.11613 | 356.12235 | 120.05781 | 2.00622 | 190583 | 1 | 1 | [2](http://www.mycompoundid.org/mycompoundid_IsoMS/myid_search_res/1481082226320-2016-12-6-8-43-46-858582/1481082226741-828-120.0578089-Neutral.htm) |
| 249 | 1320.01 | 354.11670 | 356.12358 | 120.05838 | 2.00689 | 821875 | 1 | 1 | [2](http://www.mycompoundid.org/mycompoundid_IsoMS/myid_search_res/1481082226320-2016-12-6-8-43-46-858582/1481082226744-946-120.0583782-Neutral.htm) |
| 250 | 1325.81 | 338.59329 | 340.60018 | 209.06993 | 2.00689 | 24400 | 2 | 2 | [1](http://www.mycompoundid.org/mycompoundid_IsoMS/myid_search_res/1481082226320-2016-12-6-8-43-46-858582/1481082226745-814-209.069934-Neutral.htm) |
| 251 | 1331.07 | 345.60138 | 347.60789 | 223.08612 | 2.00652 | 34638 | 2 | 2 | [1](http://www.mycompoundid.org/mycompoundid_IsoMS/myid_search_res/1481082226320-2016-12-6-8-43-46-858582/1481082226745-250-223.0861163-Neutral.htm) |
| 252 | 1352.47 | 297.59040 | 299.59735 | 127.06417 | 2.00694 | 57313 | 2 | 2 | [2](http://www.mycompoundid.org/mycompoundid_IsoMS/myid_search_res/1481082226320-2016-12-6-8-43-46-858582/1481082226750-655-127.0641674-Neutral.htm) |
| 253 | 1353.41 | 577.15698 | 581.16941 | 110.04764 | 4.01243 | 21538 | 1 | 2 | [1](http://www.mycompoundid.org/mycompoundid_IsoMS/myid_search_res/1481082226320-2016-12-6-8-43-46-858582/1481082226751-378-110.0476404-Neutral.htm) |
| 254 | 1362.27 | 308.58151 | 310.58939 | 149.04638 | 2.00788 | 29494 | 2 | 2 | [1](http://www.mycompoundid.org/mycompoundid_IsoMS/myid_search_res/1481082226320-2016-12-6-8-43-46-858582/1481082226754-175-149.0463824-Neutral.htm) |
| 255 | 1363.36 | 313.59304 | 315.59988 | 159.06944 | 2.00684 | 37797 | 2 | 2 | [1](http://www.mycompoundid.org/mycompoundid_IsoMS/myid_search_res/1481082226320-2016-12-6-8-43-46-858582/1481082226754-560-159.0694381-Neutral.htm) |
| 256 | 1367.48 | 577.15696 | 581.17035 | 110.04762 | 4.01339 | 27553 | 1 | 2 | [1](http://www.mycompoundid.org/mycompoundid_IsoMS/myid_search_res/1481082226320-2016-12-6-8-43-46-858582/1481082226755-553-110.0476247-Neutral.htm) |
| 257 | 1375.96 | 344.10595 | 346.11224 | 110.04763 | 2.00629 | 33750 | 1 | 1 | [1](http://www.mycompoundid.org/mycompoundid_IsoMS/myid_search_res/1481082226320-2016-12-6-8-43-46-858582/1481082226756-436-110.0476275-Neutral.htm) |
| 258 | 1378.47 | 328.13459 | 330.14174 | 188.15255 | 2.00715 | 9450 | 2 | 2 | [1](http://www.mycompoundid.org/mycompoundid_IsoMS/myid_search_res/1481082226320-2016-12-6-8-43-46-858582/1481082226756-955-188.1525475-Neutral.htm) |
| 259 | 1385.41 | 302.07785 | 304.08488 | 136.03906 | 2.00703 | 36913 | 2 | 2 | [1](http://www.mycompoundid.org/mycompoundid_IsoMS/myid_search_res/1481082226320-2016-12-6-8-43-46-858582/1481082226757-12-136.0390614-Neutral.htm) |
| 260 | 1394.42 | 612.26697 | 616.28060 | 145.15763 | 4.01364 | 55681 | 1 | 2 | [1](http://www.mycompoundid.org/mycompoundid_IsoMS/myid_search_res/1481082226320-2016-12-6-8-43-46-858582/1481082226760-205-145.1576293-Neutral.htm) |
| 261 | 1425.70 | 323.60645 | 325.61317 | 179.09626 | 2.00672 | 12753 | 2 | 2 | [2](http://www.mycompoundid.org/mycompoundid_IsoMS/myid_search_res/1481082226320-2016-12-6-8-43-46-858582/1481082226767-785-179.096262-Neutral.htm) |
| # | RT (s) | mz_light | mz_heavy | mz | distance | int_light | nCharge | nTag | Possible hits |
| 262 | 1486.25 | 669.32514 | 673.33790 | 202.21580 | 4.01276 | 15800 | 1 | 2 | [1](http://www.mycompoundid.org/mycompoundid_IsoMS/myid_search_res/1481082226320-2016-12-6-8-43-46-858582/1481082226779-3-202.215801-Neutral.htm) |
| 263 | 1486.89 | 570.29100 | 572.29316 | 336.23268 | 2.00216 | 11450 | 1 | 1 | [22](http://www.mycompoundid.org/mycompoundid_IsoMS/myid_search_res/1481082226320-2016-12-6-8-43-46-858582/1481082226779-890-336.232678-Neutral.htm) |
| 264 | 1493.26 | 685.32688 | 687.33352 | 451.26856 | 2.00664 | 11730 | 1 | 1 | [2](http://www.mycompoundid.org/mycompoundid_IsoMS/myid_search_res/1481082226320-2016-12-6-8-43-46-858582/1481082226781-982-451.2685635-Neutral.htm) |
| 265 | 1499.84 | 320.06730 | 322.07331 | 172.01796 | 2.00601 | 1060000 | 2 | 2 | [1](http://www.mycompoundid.org/mycompoundid_IsoMS/myid_search_res/1481082226320-2016-12-6-8-43-46-858582/1481082226782-210-172.0179641-Neutral.htm) |
| 266 | 1500.01 | 319.06653 | 321.07326 | 170.01643 | 2.00673 | 2623328 | 2 | 2 | [1](http://www.mycompoundid.org/mycompoundid_IsoMS/myid_search_res/1481082226320-2016-12-6-8-43-46-858582/1481082226783-543-170.0164286-Neutral.htm) |
| 267 | 1500.28 | 403.06362 | 405.06748 | 169.00530 | 2.00387 | 41691 | 1 | 1 | [1](http://www.mycompoundid.org/mycompoundid_IsoMS/myid_search_res/1481082226320-2016-12-6-8-43-46-858582/1481082226783-797-169.0052958-Neutral.htm) |
| 268 | 1506.67 | 584.30725 | 586.30957 | 350.24893 | 2.00232 | 9120 | 1 | 1 | [5](http://www.mycompoundid.org/mycompoundid_IsoMS/myid_search_res/1481082226320-2016-12-6-8-43-46-858582/1481082226784-739-350.2489345-Neutral.htm) |
| 269 | 1513.80 | 735.34388 | 737.35017 | 501.28556 | 2.00629 | 94963 | 1 | 1 | [5](http://www.mycompoundid.org/mycompoundid_IsoMS/myid_search_res/1481082226320-2016-12-6-8-43-46-858582/1481082226785-508-501.2855619-Neutral.htm) |
| 270 | 1523.31 | 711.34181 | 713.35179 | 477.28349 | 2.00998 | 7930 | 1 | 1 | [2](http://www.mycompoundid.org/mycompoundid_IsoMS/myid_search_res/1481082226320-2016-12-6-8-43-46-858582/1481082226788-349-477.283487-Neutral.htm) |
| 271 | 1532.61 | 761.35818 | 763.36785 | 527.29986 | 2.00967 | 13400 | 1 | 1 | [4](http://www.mycompoundid.org/mycompoundid_IsoMS/myid_search_res/1481082226320-2016-12-6-8-43-46-858582/1481082226790-872-527.299863-Neutral.htm) |
| 272 | 1541.38 | 470.23568 | 472.24189 | 236.17736 | 2.00621 | 69206 | 1 | 1 | [1](http://www.mycompoundid.org/mycompoundid_IsoMS/myid_search_res/1481082226320-2016-12-6-8-43-46-858582/1481082226791-882-236.1773577-Neutral.htm) |
| 273 | 1549.90 | 533.33992 | 535.34661 | 299.28160 | 2.00669 | 57257 | 1 | 1 | [3](http://www.mycompoundid.org/mycompoundid_IsoMS/myid_search_res/1481082226320-2016-12-6-8-43-46-858582/1481082226791-585-299.2815992-Neutral.htm) |
| 274 | 1566.31 | 535.35505 | 537.36188 | 301.29673 | 2.00683 | 21875 | 1 | 1 | [1](http://www.mycompoundid.org/mycompoundid_IsoMS/myid_search_res/1481082226320-2016-12-6-8-43-46-858582/1481082226793-626-301.2967315-Neutral.htm) |
| 275 | 1574.10 | 489.31422 | 491.32056 | 255.25590 | 2.00634 | 104750 | 1 | 1 | [1](http://www.mycompoundid.org/mycompoundid_IsoMS/myid_search_res/1481082226320-2016-12-6-8-43-46-858582/1481082226794-526-255.2558965-Neutral.htm) |
| 276 | 1580.99 | 515.32889 | 517.33456 | 281.27057 | 2.00567 | 7580 | 1 | 1 | [1](http://www.mycompoundid.org/mycompoundid_IsoMS/myid_search_res/1481082226320-2016-12-6-8-43-46-858582/1481082226795-415-281.270565-Neutral.htm) |
| 277 | 1602.43 | 687.34515 | 689.35050 | 453.28683 | 2.00535 | 6765 | 1 | 1 | [2](http://www.mycompoundid.org/mycompoundid_IsoMS/myid_search_res/1481082226320-2016-12-6-8-43-46-858582/1481082226797-684-453.286833-Neutral.htm) |
| 278 | 1619.19 | 713.35902 | 715.36405 | 479.30070 | 2.00503 | 21000 | 1 | 1 | [4](http://www.mycompoundid.org/mycompoundid_IsoMS/myid_search_res/1481082226320-2016-12-6-8-43-46-858582/1481082226798-657-479.300698-Neutral.htm) |
| 279 | 1631.72 | 713.35865 | 715.36492 | 479.30033 | 2.00626 | 19620 | 1 | 1 | [4](http://www.mycompoundid.org/mycompoundid_IsoMS/myid_search_res/1481082226320-2016-12-6-8-43-46-858582/1481082226798-902-479.3003345-Neutral.htm) |
| 280 | 1689.44 | 671.34698 | 673.35294 | 437.28866 | 2.00596 | 7760 | 1 | 1 | [1](http://www.mycompoundid.org/mycompoundid_IsoMS/myid_search_res/1481082226320-2016-12-6-8-43-46-858582/1481082226799-190-437.2886599-Neutral.htm) |
| 281 | 1707.69 | 671.34857 | 673.35182 | 437.29025 | 2.00325 | 7600 | 1 | 1 | [1](http://www.mycompoundid.org/mycompoundid_IsoMS/myid_search_res/1481082226320-2016-12-6-8-43-46-858582/1481082226799-955-437.290247-Neutral.htm) |
|  |  |  |  |  |  |  |  |  |  |

Table S1C. Identification of cellular metabolites by searching the accurate mass of the peak pairs against the EML library with one reaction in MCID.

| # | RT (s) | mz_light | mz_heavy | mz | distance | int_light | nCharge | nTag | Possible hits |
| --- | --- | --- | --- | --- | --- | --- | --- | --- | --- |
| 1 | 125.47 | 425.18512 | 427.19137 | 191.12680 | 2.00625 | 94200 | 1 | 1 | [2](http://www.mycompoundid.org/mycompoundid_IsoMS/myid_search_res/1481082404607-2016-12-6-8-46-44-116748/1481082404607-922-191.126801-Neutral.htm) |
| 2 | 126.55 | 426.16865 | 428.17552 | 192.11033 | 2.00687 | 15700 | 1 | 1 | [4](http://www.mycompoundid.org/mycompoundid_IsoMS/myid_search_res/1481082404607-2016-12-6-8-46-44-116748/1481082404608-455-192.110325-Neutral.htm) |
| 3 | 128.55 | 375.07841 | 377.08489 | 141.02009 | 2.00648 | 1090000 | 1 | 1 | [6](http://www.mycompoundid.org/mycompoundid_IsoMS/myid_search_res/1481082404607-2016-12-6-8-46-44-116748/1481082404609-266-141.020086-Neutral.htm) |
| 4 | 130.55 | 367.09644 | 369.10373 | 133.03812 | 2.00730 | 1100000 | 1 | 1 | [28](http://www.mycompoundid.org/mycompoundid_IsoMS/myid_search_res/1481082404607-2016-12-6-8-46-44-116748/1481082404609-664-133.038116-Neutral.htm) |
| 5 | 134.16 | 411.16900 | 413.17542 | 177.11068 | 2.00641 | 30500 | 1 | 1 | [4](http://www.mycompoundid.org/mycompoundid_IsoMS/myid_search_res/1481082404607-2016-12-6-8-46-44-116748/1481082404611-253-177.1106835-Neutral.htm) |
| 6 | 135.60 | 449.11410 | 451.11910 | 215.05578 | 2.00500 | 29175 | 1 | 1 | [4](http://www.mycompoundid.org/mycompoundid_IsoMS/myid_search_res/1481082404607-2016-12-6-8-46-44-116748/1481082404611-580-215.0557801-Neutral.htm) |
| 7 | 135.83 | 381.11109 | 383.11661 | 147.05277 | 2.00552 | 14850 | 1 | 1 | [51](http://www.mycompoundid.org/mycompoundid_IsoMS/myid_search_res/1481082404607-2016-12-6-8-46-44-116748/1481082404612-295-147.05277-Neutral.htm) |
| 8 | 136.16 | 366.11233 | 368.11887 | 132.05401 | 2.00654 | 3630000 | 1 | 1 | [12](http://www.mycompoundid.org/mycompoundid_IsoMS/myid_search_res/1481082404607-2016-12-6-8-46-44-116748/1481082404614-106-132.0540115-Neutral.htm) |
| 9 | 150.91 | 675.24145 | 677.24905 | 441.18313 | 2.00760 | 11385 | 1 | 1 | [7](http://www.mycompoundid.org/mycompoundid_IsoMS/myid_search_res/1481082404607-2016-12-6-8-46-44-116748/1481082404615-918-441.1831253-Neutral.htm) |
| 10 | 151.91 | 380.12827 | 382.13458 | 146.06995 | 2.00631 | 5385000 | 1 | 1 | [29](http://www.mycompoundid.org/mycompoundid_IsoMS/myid_search_res/1481082404607-2016-12-6-8-46-44-116748/1481082404616-59-146.0699525-Neutral.htm) |
| 11 | 152.99 | 280.10057 | 282.10707 | 46.04225 | 2.00650 | 533125 | 1 | 1 | [20](http://www.mycompoundid.org/mycompoundid_IsoMS/myid_search_res/1481082404607-2016-12-6-8-46-44-116748/1481082404618-705-46.04224869-Neutral.htm) |
| 12 | 154.60 | 449.11495 | 451.12088 | 215.05663 | 2.00594 | 295375 | 1 | 1 | [4](http://www.mycompoundid.org/mycompoundid_IsoMS/myid_search_res/1481082404607-2016-12-6-8-46-44-116748/1481082404621-479-215.0566259-Neutral.htm) |
| 13 | 157.45 | 474.14296 | 476.15005 | 240.08464 | 2.00708 | 11725 | 1 | 1 | [25](http://www.mycompoundid.org/mycompoundid_IsoMS/myid_search_res/1481082404607-2016-12-6-8-46-44-116748/1481082404621-693-240.084644-Neutral.htm) |
| 14 | 166.46 | 675.24238 | 677.24879 | 441.18406 | 2.00640 | 30724 | 1 | 1 | [7](http://www.mycompoundid.org/mycompoundid_IsoMS/myid_search_res/1481082404607-2016-12-6-8-46-44-116748/1481082404623-834-441.184061-Neutral.htm) |
| 15 | 167.81 | 449.11502 | 451.12073 | 215.05670 | 2.00571 | 358969 | 1 | 1 | [4](http://www.mycompoundid.org/mycompoundid_IsoMS/myid_search_res/1481082404607-2016-12-6-8-46-44-116748/1481082404624-394-215.0567014-Neutral.htm) |
| 16 | 170.33 | 474.14361 | 476.15037 | 240.08529 | 2.00676 | 13441 | 1 | 1 | [29](http://www.mycompoundid.org/mycompoundid_IsoMS/myid_search_res/1481082404607-2016-12-6-8-46-44-116748/1481082404624-507-240.0852931-Neutral.htm) |
| 17 | 182.09 | 573.13205 | 575.13766 | 339.07373 | 2.00561 | 121653 | 1 | 1 | [4](http://www.mycompoundid.org/mycompoundid_IsoMS/myid_search_res/1481082404607-2016-12-6-8-46-44-116748/1481082404625-530-339.0737274-Neutral.htm) |
| 18 | 182.60 | 366.11259 | 368.11900 | 132.05427 | 2.00641 | 4930000 | 1 | 1 | [12](http://www.mycompoundid.org/mycompoundid_IsoMS/myid_search_res/1481082404607-2016-12-6-8-46-44-116748/1481082404626-272-132.054266-Neutral.htm) |
| 19 | 182.93 | 589.12591 | 591.13139 | 355.06759 | 2.00548 | 13294 | 1 | 1 | [8](http://www.mycompoundid.org/mycompoundid_IsoMS/myid_search_res/1481082404607-2016-12-6-8-46-44-116748/1481082404626-499-355.0675869-Neutral.htm) |
| 20 | 184.50 | 389.12732 | 391.13404 | 155.06900 | 2.00672 | 18850 | 1 | 1 | [14](http://www.mycompoundid.org/mycompoundid_IsoMS/myid_search_res/1481082404607-2016-12-6-8-46-44-116748/1481082404627-232-155.0690005-Neutral.htm) |
| # | RT (s) | mz_light | mz_heavy | mz | distance | int_light | nCharge | nTag | Possible hits |
| 21 | 185.26 | 381.11133 | 383.11619 | 147.05301 | 2.00485 | 30650 | 1 | 1 | [51](http://www.mycompoundid.org/mycompoundid_IsoMS/myid_search_res/1481082404607-2016-12-6-8-46-44-116748/1481082404628-995-147.053014-Neutral.htm) |
| 22 | 187.53 | 410.17510 | 412.18165 | 176.11678 | 2.00655 | 8520 | 1 | 1 | [12](http://www.mycompoundid.org/mycompoundid_IsoMS/myid_search_res/1481082404607-2016-12-6-8-46-44-116748/1481082404630-554-176.116777-Neutral.htm) |
| 23 | 193.02 | 675.24287 | 677.24785 | 441.18455 | 2.00498 | 13244 | 1 | 1 | [7](http://www.mycompoundid.org/mycompoundid_IsoMS/myid_search_res/1481082404607-2016-12-6-8-46-44-116748/1481082404631-959-441.1845496-Neutral.htm) |
| 24 | 197.89 | 389.12769 | 391.13316 | 155.06937 | 2.00546 | 14900 | 1 | 1 | [14](http://www.mycompoundid.org/mycompoundid_IsoMS/myid_search_res/1481082404607-2016-12-6-8-46-44-116748/1481082404632-251-155.0693748-Neutral.htm) |
| 25 | 200.90 | 359.07348 | 361.07845 | 125.01516 | 2.00497 | 150650 | 1 | 1 | [5](http://www.mycompoundid.org/mycompoundid_IsoMS/myid_search_res/1481082404607-2016-12-6-8-46-44-116748/1481082404632-886-125.0151573-Neutral.htm) |
| 26 | 201.63 | 535.17213 | 537.17810 | 301.11381 | 2.00597 | 17100 | 1 | 1 | [10](http://www.mycompoundid.org/mycompoundid_IsoMS/myid_search_res/1481082404607-2016-12-6-8-46-44-116748/1481082404633-450-301.113814-Neutral.htm) |
| 27 | 201.90 | 582.25895 | 584.26552 | 348.20063 | 2.00657 | 15100 | 1 | 1 | [1](http://www.mycompoundid.org/mycompoundid_IsoMS/myid_search_res/1481082404607-2016-12-6-8-46-44-116748/1481082404633-731-348.20063-Neutral.htm) |
| 28 | 206.70 | 675.24158 | 677.24828 | 441.18326 | 2.00670 | 13200 | 1 | 1 | [7](http://www.mycompoundid.org/mycompoundid_IsoMS/myid_search_res/1481082404607-2016-12-6-8-46-44-116748/1481082404634-724-441.183258-Neutral.htm) |
| 29 | 208.77 | 388.10798 | 390.11436 | 154.04966 | 2.00638 | 591813 | 1 | 1 | [11](http://www.mycompoundid.org/mycompoundid_IsoMS/myid_search_res/1481082404607-2016-12-6-8-46-44-116748/1481082404634-416-154.0496574-Neutral.htm) |
| 30 | 211.31 | 389.12757 | 391.13309 | 155.06925 | 2.00552 | 23100 | 1 | 1 | [14](http://www.mycompoundid.org/mycompoundid_IsoMS/myid_search_res/1481082404607-2016-12-6-8-46-44-116748/1481082404635-400-155.0692523-Neutral.htm) |
| 31 | 212.64 | 380.12853 | 382.13487 | 146.07021 | 2.00634 | 7150000 | 1 | 1 | [29](http://www.mycompoundid.org/mycompoundid_IsoMS/myid_search_res/1481082404607-2016-12-6-8-46-44-116748/1481082404636-797-146.070205-Neutral.htm) |
| 32 | 214.39 | 517.15048 | 519.15673 | 283.09216 | 2.00626 | 57238 | 1 | 1 | [38](http://www.mycompoundid.org/mycompoundid_IsoMS/myid_search_res/1481082404607-2016-12-6-8-46-44-116748/1481082404637-363-283.0921552-Neutral.htm) |
| 33 | 215.35 | 359.07352 | 361.07922 | 125.01520 | 2.00569 | 257066 | 1 | 1 | [5](http://www.mycompoundid.org/mycompoundid_IsoMS/myid_search_res/1481082404607-2016-12-6-8-46-44-116748/1481082404639-727-125.0152037-Neutral.htm) |
| 34 | 216.54 | 513.18919 | 515.19486 | 279.13087 | 2.00566 | 16439 | 1 | 1 | [15](http://www.mycompoundid.org/mycompoundid_IsoMS/myid_search_res/1481082404607-2016-12-6-8-46-44-116748/1481082404639-128-279.1308741-Neutral.htm) |
| 35 | 218.29 | 675.24422 | 677.24892 | 441.18590 | 2.00470 | 17150 | 1 | 1 | [7](http://www.mycompoundid.org/mycompoundid_IsoMS/myid_search_res/1481082404607-2016-12-6-8-46-44-116748/1481082404640-359-441.185899-Neutral.htm) |
| 36 | 220.65 | 339.10168 | 341.10819 | 105.04336 | 2.00652 | 947000 | 1 | 1 | [21](http://www.mycompoundid.org/mycompoundid_IsoMS/myid_search_res/1481082404607-2016-12-6-8-46-44-116748/1481082404640-595-105.043356-Neutral.htm) |
| 37 | 223.22 | 389.09338 | 391.10004 | 155.03506 | 2.00666 | 185500 | 1 | 1 | [5](http://www.mycompoundid.org/mycompoundid_IsoMS/myid_search_res/1481082404607-2016-12-6-8-46-44-116748/1481082404641-452-155.0350635-Neutral.htm) |
| 38 | 227.01 | 517.15056 | 519.15586 | 283.09224 | 2.00530 | 40500 | 1 | 1 | [39](http://www.mycompoundid.org/mycompoundid_IsoMS/myid_search_res/1481082404607-2016-12-6-8-46-44-116748/1481082404642-379-283.09224-Neutral.htm) |
| 39 | 230.26 | 365.11736 | 367.12365 | 131.05904 | 2.00628 | 3980000 | 1 | 1 | [60](http://www.mycompoundid.org/mycompoundid_IsoMS/myid_search_res/1481082404607-2016-12-6-8-46-44-116748/1481082404645-646-131.0590425-Neutral.htm) |
| 40 | 231.94 | 715.24816 | 717.25234 | 481.18984 | 2.00418 | 7860 | 1 | 1 | [6](http://www.mycompoundid.org/mycompoundid_IsoMS/myid_search_res/1481082404607-2016-12-6-8-46-44-116748/1481082404648-412-481.189843-Neutral.htm) |
| 41 | 235.66 | 783.23635 | 785.24108 | 549.17803 | 2.00474 | 42800 | 1 | 1 | [2](http://www.mycompoundid.org/mycompoundid_IsoMS/myid_search_res/1481082404607-2016-12-6-8-46-44-116748/1481082404648-104-549.178025-Neutral.htm) |
| 42 | 239.27 | 517.15025 | 519.15680 | 283.09193 | 2.00655 | 123781 | 1 | 1 | [38](http://www.mycompoundid.org/mycompoundid_IsoMS/myid_search_res/1481082404607-2016-12-6-8-46-44-116748/1481082404648-518-283.0919288-Neutral.htm) |
| 43 | 241.67 | 408.17093 | 410.17733 | 174.11261 | 2.00640 | 422000 | 1 | 1 | [6](http://www.mycompoundid.org/mycompoundid_IsoMS/myid_search_res/1481082404607-2016-12-6-8-46-44-116748/1481082404650-293-174.112606-Neutral.htm) |
| 44 | 243.67 | 339.10156 | 341.10796 | 105.04324 | 2.00640 | 716000 | 1 | 1 | [21](http://www.mycompoundid.org/mycompoundid_IsoMS/myid_search_res/1481082404607-2016-12-6-8-46-44-116748/1481082404650-587-105.04324-Neutral.htm) |
| # | RT (s) | mz_light | mz_heavy | mz | distance | int_light | nCharge | nTag | Possible hits |
| 45 | 245.84 | 293.10647 | 295.11258 | 59.04815 | 2.00611 | 36719 | 1 | 1 | [1](http://www.mycompoundid.org/mycompoundid_IsoMS/myid_search_res/1481082404607-2016-12-6-8-46-44-116748/1481082404651-171-59.04815006-Neutral.htm) |
| 46 | 245.96 | 380.12882 | 382.13501 | 146.07050 | 2.00619 | 1151250 | 1 | 1 | [29](http://www.mycompoundid.org/mycompoundid_IsoMS/myid_search_res/1481082404607-2016-12-6-8-46-44-116748/1481082404652-436-146.0704984-Neutral.htm) |
| 47 | 246.46 | 463.12992 | 465.13591 | 229.07160 | 2.00599 | 21056 | 1 | 1 | [8](http://www.mycompoundid.org/mycompoundid_IsoMS/myid_search_res/1481082404607-2016-12-6-8-46-44-116748/1481082404653-615-229.0716042-Neutral.htm) |
| 48 | 251.50 | 403.14269 | 405.14879 | 169.08437 | 2.00611 | 18700 | 1 | 1 | [11](http://www.mycompoundid.org/mycompoundid_IsoMS/myid_search_res/1481082404607-2016-12-6-8-46-44-116748/1481082404653-22-169.0843675-Neutral.htm) |
| 49 | 252.68 | 485.15814 | 487.16431 | 251.09982 | 2.00618 | 10600 | 1 | 1 | [39](http://www.mycompoundid.org/mycompoundid_IsoMS/myid_search_res/1481082404607-2016-12-6-8-46-44-116748/1481082404654-62-251.099815-Neutral.htm) |
| 50 | 253.98 | 397.12472 | 399.13073 | 163.06640 | 2.00602 | 70800 | 1 | 1 | [5](http://www.mycompoundid.org/mycompoundid_IsoMS/myid_search_res/1481082404607-2016-12-6-8-46-44-116748/1481082404655-909-163.066396-Neutral.htm) |
| 51 | 256.27 | 675.24271 | 677.24920 | 441.18439 | 2.00649 | 48894 | 1 | 1 | [7](http://www.mycompoundid.org/mycompoundid_IsoMS/myid_search_res/1481082404607-2016-12-6-8-46-44-116748/1481082404656-700-441.1843863-Neutral.htm) |
| 52 | 257.36 | 380.13084 | 382.13712 | 146.07252 | 2.00627 | 1395000 | 1 | 1 | [10](http://www.mycompoundid.org/mycompoundid_IsoMS/myid_search_res/1481082404607-2016-12-6-8-46-44-116748/1481082404656-917-146.072521-Neutral.htm) |
| 53 | 258.35 | 513.18902 | 515.19378 | 279.13070 | 2.00476 | 9205 | 1 | 1 | [16](http://www.mycompoundid.org/mycompoundid_IsoMS/myid_search_res/1481082404607-2016-12-6-8-46-44-116748/1481082404657-215-279.1306979-Neutral.htm) |
| 54 | 259.28 | 381.11129 | 383.11740 | 147.05297 | 2.00611 | 98400 | 1 | 1 | [51](http://www.mycompoundid.org/mycompoundid_IsoMS/myid_search_res/1481082404607-2016-12-6-8-46-44-116748/1481082404658-811-147.052967-Neutral.htm) |
| 55 | 261.28 | 424.11749 | 426.12373 | 190.05917 | 2.00624 | 12200 | 1 | 1 | [15](http://www.mycompoundid.org/mycompoundid_IsoMS/myid_search_res/1481082404607-2016-12-6-8-46-44-116748/1481082404659-197-190.059168-Neutral.htm) |
| 56 | 262.06 | 408.17053 | 410.17718 | 174.11221 | 2.00664 | 910375 | 1 | 1 | [6](http://www.mycompoundid.org/mycompoundid_IsoMS/myid_search_res/1481082404607-2016-12-6-8-46-44-116748/1481082404660-112-174.1122145-Neutral.htm) |
| 57 | 262.21 | 493.17368 | 495.17912 | 259.11536 | 2.00544 | 11975 | 1 | 1 | [10](http://www.mycompoundid.org/mycompoundid_IsoMS/myid_search_res/1481082404607-2016-12-6-8-46-44-116748/1481082404660-635-259.115362-Neutral.htm) |
| 58 | 262.60 | 414.12169 | 416.12685 | 180.06337 | 2.00516 | 14375 | 1 | 1 | [87](http://www.mycompoundid.org/mycompoundid_IsoMS/myid_search_res/1481082404607-2016-12-6-8-46-44-116748/1481082404661-408-180.0633688-Neutral.htm) |
| 59 | 263.63 | 509.16934 | 511.17527 | 275.11102 | 2.00592 | 10886 | 1 | 1 | [16](http://www.mycompoundid.org/mycompoundid_IsoMS/myid_search_res/1481082404607-2016-12-6-8-46-44-116748/1481082404664-68-275.1110231-Neutral.htm) |
| 60 | 265.42 | 417.15810 | 419.16464 | 183.09978 | 2.00654 | 18738 | 1 | 1 | [8](http://www.mycompoundid.org/mycompoundid_IsoMS/myid_search_res/1481082404607-2016-12-6-8-46-44-116748/1481082404665-117-183.0997786-Neutral.htm) |
| 61 | 267.30 | 366.11246 | 368.11905 | 132.05414 | 2.00658 | 2732500 | 1 | 1 | [12](http://www.mycompoundid.org/mycompoundid_IsoMS/myid_search_res/1481082404607-2016-12-6-8-46-44-116748/1481082404665-491-132.0541443-Neutral.htm) |
| 62 | 267.96 | 587.14581 | 589.15073 | 353.08749 | 2.00492 | 7780 | 1 | 1 | [6](http://www.mycompoundid.org/mycompoundid_IsoMS/myid_search_res/1481082404607-2016-12-6-8-46-44-116748/1481082404668-869-353.087487-Neutral.htm) |
| 63 | 270.46 | 502.13862 | 504.14406 | 268.08030 | 2.00544 | 48378 | 1 | 1 | [23](http://www.mycompoundid.org/mycompoundid_IsoMS/myid_search_res/1481082404607-2016-12-6-8-46-44-116748/1481082404669-371-268.0802963-Neutral.htm) |
| 64 | 270.70 | 535.18251 | 537.18613 | 301.12419 | 2.00362 | 11400 | 1 | 1 | [2](http://www.mycompoundid.org/mycompoundid_IsoMS/myid_search_res/1481082404607-2016-12-6-8-46-44-116748/1481082404670-437-301.124187-Neutral.htm) |
| 65 | 273.53 | 675.24242 | 677.24899 | 441.18410 | 2.00656 | 57500 | 1 | 1 | [7](http://www.mycompoundid.org/mycompoundid_IsoMS/myid_search_res/1481082404607-2016-12-6-8-46-44-116748/1481082404670-688-441.1841038-Neutral.htm) |
| 66 | 274.20 | 657.23177 | 659.23769 | 423.17345 | 2.00592 | 14500 | 1 | 1 | [14](http://www.mycompoundid.org/mycompoundid_IsoMS/myid_search_res/1481082404607-2016-12-6-8-46-44-116748/1481082404670-71-423.173445-Neutral.htm) |
| 67 | 274.21 | 513.18572 | 515.19603 | 279.12740 | 2.01031 | 11050 | 1 | 1 | [3](http://www.mycompoundid.org/mycompoundid_IsoMS/myid_search_res/1481082404607-2016-12-6-8-46-44-116748/1481082404671-775-279.1274015-Neutral.htm) |
| 68 | 274.87 | 414.12151 | 416.12753 | 180.06319 | 2.00602 | 25619 | 1 | 1 | [87](http://www.mycompoundid.org/mycompoundid_IsoMS/myid_search_res/1481082404607-2016-12-6-8-46-44-116748/1481082404671-286-180.063194-Neutral.htm) |
| # | RT (s) | mz_light | mz_heavy | mz | distance | int_light | nCharge | nTag | Possible hits |
| 69 | 275.23 | 489.13251 | 491.13872 | 255.07419 | 2.00621 | 89125 | 1 | 1 | [16](http://www.mycompoundid.org/mycompoundid_IsoMS/myid_search_res/1481082404607-2016-12-6-8-46-44-116748/1481082404674-337-255.0741895-Neutral.htm) |
| 70 | 275.78 | 501.11490 | 505.12821 | 34.00556 | 4.01330 | 8610 | 1 | 2 | [8](http://www.mycompoundid.org/mycompoundid_IsoMS/myid_search_res/1481082404607-2016-12-6-8-46-44-116748/1481082404675-982-34.005562-Neutral.htm) |
| 71 | 277.53 | 417.15907 | 419.16418 | 183.10075 | 2.00510 | 38134 | 1 | 1 | [8](http://www.mycompoundid.org/mycompoundid_IsoMS/myid_search_res/1481082404607-2016-12-6-8-46-44-116748/1481082404676-259-183.100754-Neutral.htm) |
| 72 | 278.70 | 365.11775 | 367.12414 | 131.05943 | 2.00639 | 5270000 | 1 | 1 | [60](http://www.mycompoundid.org/mycompoundid_IsoMS/myid_search_res/1481082404607-2016-12-6-8-46-44-116748/1481082404676-959-131.059432-Neutral.htm) |
| 73 | 283.75 | 702.25257 | 704.25909 | 468.19425 | 2.00652 | 6130 | 1 | 1 | [5](http://www.mycompoundid.org/mycompoundid_IsoMS/myid_search_res/1481082404607-2016-12-6-8-46-44-116748/1481082404678-555-468.1942475-Neutral.htm) |
| 74 | 287.55 | 408.17227 | 410.17845 | 174.11395 | 2.00618 | 1516654 | 1 | 1 | [3](http://www.mycompoundid.org/mycompoundid_IsoMS/myid_search_res/1481082404607-2016-12-6-8-46-44-116748/1481082404679-326-174.1139522-Neutral.htm) |
| 75 | 288.82 | 502.13914 | 504.14539 | 268.08082 | 2.00625 | 67813 | 1 | 1 | [23](http://www.mycompoundid.org/mycompoundid_IsoMS/myid_search_res/1481082404607-2016-12-6-8-46-44-116748/1481082404679-979-268.0808221-Neutral.htm) |
| 76 | 290.72 | 479.15977 | 481.16644 | 245.10145 | 2.00667 | 10735 | 1 | 1 | [14](http://www.mycompoundid.org/mycompoundid_IsoMS/myid_search_res/1481082404607-2016-12-6-8-46-44-116748/1481082404680-331-245.1014505-Neutral.htm) |
| 77 | 294.79 | 380.12789 | 382.13457 | 146.06957 | 2.00668 | 8270000 | 1 | 1 | [29](http://www.mycompoundid.org/mycompoundid_IsoMS/myid_search_res/1481082404607-2016-12-6-8-46-44-116748/1481082404681-85-146.069574-Neutral.htm) |
| 78 | 297.76 | 702.25317 | 704.25987 | 468.19485 | 2.00670 | 14100 | 1 | 1 | [5](http://www.mycompoundid.org/mycompoundid_IsoMS/myid_search_res/1481082404607-2016-12-6-8-46-44-116748/1481082404682-944-468.1948508-Neutral.htm) |
| 79 | 300.73 | 535.18142 | 537.18586 | 301.12310 | 2.00444 | 24900 | 1 | 1 | [2](http://www.mycompoundid.org/mycompoundid_IsoMS/myid_search_res/1481082404607-2016-12-6-8-46-44-116748/1481082404682-504-301.123095-Neutral.htm) |
| 80 | 303.29 | 422.20971 | 424.21525 | 188.15139 | 2.00555 | 12800 | 1 | 1 | [4](http://www.mycompoundid.org/mycompoundid_IsoMS/myid_search_res/1481082404607-2016-12-6-8-46-44-116748/1481082404683-771-188.151385-Neutral.htm) |
| 81 | 304.28 | 489.13277 | 491.13910 | 255.07445 | 2.00633 | 119188 | 1 | 1 | [16](http://www.mycompoundid.org/mycompoundid_IsoMS/myid_search_res/1481082404607-2016-12-6-8-46-44-116748/1481082404683-494-255.0744541-Neutral.htm) |
| 82 | 305.88 | 513.18877 | 515.19398 | 279.13045 | 2.00521 | 21138 | 1 | 1 | [16](http://www.mycompoundid.org/mycompoundid_IsoMS/myid_search_res/1481082404607-2016-12-6-8-46-44-116748/1481082404684-498-279.1304475-Neutral.htm) |
| 83 | 307.52 | 367.09617 | 369.10188 | 133.03785 | 2.00571 | 73800 | 1 | 1 | [28](http://www.mycompoundid.org/mycompoundid_IsoMS/myid_search_res/1481082404607-2016-12-6-8-46-44-116748/1481082404685-319-133.037849-Neutral.htm) |
| 84 | 308.56 | 399.10441 | 401.11003 | 165.04609 | 2.00562 | 87138 | 1 | 1 | [7](http://www.mycompoundid.org/mycompoundid_IsoMS/myid_search_res/1481082404607-2016-12-6-8-46-44-116748/1481082404686-952-165.0460868-Neutral.htm) |
| 85 | 312.66 | 587.13034 | 589.13600 | 353.07202 | 2.00566 | 39200 | 1 | 1 | [6](http://www.mycompoundid.org/mycompoundid_IsoMS/myid_search_res/1481082404607-2016-12-6-8-46-44-116748/1481082404686-42-353.072017-Neutral.htm) |
| 86 | 313.95 | 353.11661 | 355.12308 | 119.05829 | 2.00647 | 17095 | 1 | 1 | [35](http://www.mycompoundid.org/mycompoundid_IsoMS/myid_search_res/1481082404607-2016-12-6-8-46-44-116748/1481082404687-353-119.0582949-Neutral.htm) |
| 87 | 315.35 | 422.20975 | 424.21408 | 188.15143 | 2.00433 | 18800 | 1 | 1 | [4](http://www.mycompoundid.org/mycompoundid_IsoMS/myid_search_res/1481082404607-2016-12-6-8-46-44-116748/1481082404688-67-188.1514264-Neutral.htm) |
| 88 | 316.47 | 372.14849 | 374.15487 | 138.09017 | 2.00638 | 18000 | 1 | 1 | [3](http://www.mycompoundid.org/mycompoundid_IsoMS/myid_search_res/1481082404607-2016-12-6-8-46-44-116748/1481082404689-613-138.0901684-Neutral.htm) |
| 89 | 319.31 | 381.11415 | 383.11874 | 147.05583 | 2.00459 | 1200625 | 1 | 1 | [4](http://www.mycompoundid.org/mycompoundid_IsoMS/myid_search_res/1481082404607-2016-12-6-8-46-44-116748/1481082404689-589-147.0558297-Neutral.htm) |
| 90 | 323.31 | 515.17349 | 517.17615 | 281.11517 | 2.00267 | 16400 | 1 | 1 | [32](http://www.mycompoundid.org/mycompoundid_IsoMS/myid_search_res/1481082404607-2016-12-6-8-46-44-116748/1481082404689-376-281.115166-Neutral.htm) |
| 91 | 324.58 | 436.15304 | 438.15853 | 202.09472 | 2.00549 | 15250 | 1 | 1 | [19](http://www.mycompoundid.org/mycompoundid_IsoMS/myid_search_res/1481082404607-2016-12-6-8-46-44-116748/1481082404691-428-202.0947245-Neutral.htm) |
| 92 | 324.66 | 399.10460 | 401.11050 | 165.04628 | 2.00591 | 108188 | 1 | 1 | [7](http://www.mycompoundid.org/mycompoundid_IsoMS/myid_search_res/1481082404607-2016-12-6-8-46-44-116748/1481082404691-955-165.0462789-Neutral.htm) |
| # | RT (s) | mz_light | mz_heavy | mz | distance | int_light | nCharge | nTag | Possible hits |
| 93 | 326.59 | 535.17258 | 537.17856 | 301.11426 | 2.00598 | 13145 | 1 | 1 | [10](http://www.mycompoundid.org/mycompoundid_IsoMS/myid_search_res/1481082404607-2016-12-6-8-46-44-116748/1481082404692-297-301.114261-Neutral.htm) |
| 94 | 326.75 | 499.17528 | 501.17960 | 265.11696 | 2.00433 | 22900 | 1 | 1 | [39](http://www.mycompoundid.org/mycompoundid_IsoMS/myid_search_res/1481082404607-2016-12-6-8-46-44-116748/1481082404693-992-265.116957-Neutral.htm) |
| 95 | 328.26 | 353.11570 | 355.12276 | 119.05738 | 2.00706 | 29640 | 1 | 1 | [35](http://www.mycompoundid.org/mycompoundid_IsoMS/myid_search_res/1481082404607-2016-12-6-8-46-44-116748/1481082404694-30-119.0573849-Neutral.htm) |
| 96 | 328.76 | 388.10771 | 390.11345 | 154.04939 | 2.00574 | 60800 | 1 | 1 | [11](http://www.mycompoundid.org/mycompoundid_IsoMS/myid_search_res/1481082404607-2016-12-6-8-46-44-116748/1481082404697-860-154.049385-Neutral.htm) |
| 97 | 329.03 | 513.19053 | 515.19597 | 279.13221 | 2.00544 | 91972 | 1 | 1 | [15](http://www.mycompoundid.org/mycompoundid_IsoMS/myid_search_res/1481082404607-2016-12-6-8-46-44-116748/1481082404698-480-279.1322088-Neutral.htm) |
| 98 | 329.51 | 436.20105 | 438.20708 | 202.14273 | 2.00603 | 152100 | 1 | 1 | [4](http://www.mycompoundid.org/mycompoundid_IsoMS/myid_search_res/1481082404607-2016-12-6-8-46-44-116748/1481082404699-267-202.1427264-Neutral.htm) |
| 99 | 331.76 | 691.26982 | 693.27982 | 457.21150 | 2.01000 | 11200 | 1 | 1 | [8](http://www.mycompoundid.org/mycompoundid_IsoMS/myid_search_res/1481082404607-2016-12-6-8-46-44-116748/1481082404699-166-457.211497-Neutral.htm) |
| 100 | 331.76 | 731.21454 | 733.22323 | 497.15622 | 2.00869 | 6310 | 1 | 1 | [8](http://www.mycompoundid.org/mycompoundid_IsoMS/myid_search_res/1481082404607-2016-12-6-8-46-44-116748/1481082404700-941-497.156219-Neutral.htm) |
| 101 | 334.98 | 339.10164 | 341.10806 | 105.04332 | 2.00642 | 4457328 | 1 | 1 | [21](http://www.mycompoundid.org/mycompoundid_IsoMS/myid_search_res/1481082404607-2016-12-6-8-46-44-116748/1481082404701-286-105.0433243-Neutral.htm) |
| 102 | 335.26 | 747.22248 | 749.22513 | 513.16416 | 2.00265 | 18500 | 1 | 1 | [7](http://www.mycompoundid.org/mycompoundid_IsoMS/myid_search_res/1481082404607-2016-12-6-8-46-44-116748/1481082404702-62-513.164159-Neutral.htm) |
| 103 | 336.70 | 381.11222 | 383.11861 | 147.05390 | 2.00639 | 4248125 | 1 | 1 | [55](http://www.mycompoundid.org/mycompoundid_IsoMS/myid_search_res/1481082404607-2016-12-6-8-46-44-116748/1481082404702-73-147.0539015-Neutral.htm) |
| 104 | 338.70 | 769.20559 | 771.20556 | 535.14727 | 1.99997 | 15800 | 1 | 1 | [2](http://www.mycompoundid.org/mycompoundid_IsoMS/myid_search_res/1481082404607-2016-12-6-8-46-44-116748/1481082404704-534-535.147269-Neutral.htm) |
| 105 | 341.05 | 485.15870 | 487.16574 | 251.10038 | 2.00704 | 5730 | 1 | 1 | [39](http://www.mycompoundid.org/mycompoundid_IsoMS/myid_search_res/1481082404607-2016-12-6-8-46-44-116748/1481082404705-632-251.10038-Neutral.htm) |
| 106 | 341.33 | 436.15645 | 438.16104 | 202.09813 | 2.00459 | 18200 | 1 | 1 | [1](http://www.mycompoundid.org/mycompoundid_IsoMS/myid_search_res/1481082404607-2016-12-6-8-46-44-116748/1481082404706-498-202.098131-Neutral.htm) |
| 107 | 342.77 | 501.15562 | 503.16298 | 267.09730 | 2.00736 | 757001 | 1 | 1 | [50](http://www.mycompoundid.org/mycompoundid_IsoMS/myid_search_res/1481082404607-2016-12-6-8-46-44-116748/1481082404706-969-267.0972987-Neutral.htm) |
| 108 | 348.06 | 399.10454 | 401.11017 | 165.04622 | 2.00563 | 88742 | 1 | 1 | [7](http://www.mycompoundid.org/mycompoundid_IsoMS/myid_search_res/1481082404607-2016-12-6-8-46-44-116748/1481082404708-231-165.0462215-Neutral.htm) |
| 109 | 348.18 | 436.20196 | 438.20781 | 202.14364 | 2.00585 | 303438 | 1 | 1 | [4](http://www.mycompoundid.org/mycompoundid_IsoMS/myid_search_res/1481082404607-2016-12-6-8-46-44-116748/1481082404708-38-202.1436388-Neutral.htm) |
| 110 | 353.42 | 691.27264 | 693.28047 | 457.21432 | 2.00784 | 11950 | 1 | 1 | [7](http://www.mycompoundid.org/mycompoundid_IsoMS/myid_search_res/1481082404607-2016-12-6-8-46-44-116748/1481082404709-715-457.214315-Neutral.htm) |
| 111 | 354.25 | 576.21113 | 578.21645 | 342.15281 | 2.00531 | 10695 | 1 | 1 | [11](http://www.mycompoundid.org/mycompoundid_IsoMS/myid_search_res/1481082404607-2016-12-6-8-46-44-116748/1481082404710-294-342.152813-Neutral.htm) |
| 112 | 356.79 | 309.09095 | 311.09723 | 75.03263 | 2.00628 | 4960000 | 1 | 1 | [21](http://www.mycompoundid.org/mycompoundid_IsoMS/myid_search_res/1481082404607-2016-12-6-8-46-44-116748/1481082404710-77-75.032627-Neutral.htm) |
| 113 | 357.74 | 403.14387 | 405.14969 | 169.08555 | 2.00581 | 281438 | 1 | 1 | [11](http://www.mycompoundid.org/mycompoundid_IsoMS/myid_search_res/1481082404607-2016-12-6-8-46-44-116748/1481082404711-256-169.0855542-Neutral.htm) |
| 114 | 358.65 | 438.13543 | 440.13997 | 204.07711 | 2.00454 | 21425 | 1 | 1 | [5](http://www.mycompoundid.org/mycompoundid_IsoMS/myid_search_res/1481082404607-2016-12-6-8-46-44-116748/1481082404712-271-204.0771128-Neutral.htm) |
| 115 | 364.88 | 587.12900 | 589.13489 | 353.07068 | 2.00589 | 42675 | 1 | 1 | [4](http://www.mycompoundid.org/mycompoundid_IsoMS/myid_search_res/1481082404607-2016-12-6-8-46-44-116748/1481082404753-262-353.070682-Neutral.htm) |
| 116 | 367.31 | 381.11247 | 383.11880 | 147.05415 | 2.00633 | 5609727 | 1 | 1 | [55](http://www.mycompoundid.org/mycompoundid_IsoMS/myid_search_res/1481082404607-2016-12-6-8-46-44-116748/1481082404754-82-147.0541468-Neutral.htm) |
| # | RT (s) | mz_light | mz_heavy | mz | distance | int_light | nCharge | nTag | Possible hits |
| 117 | 372.45 | 449.25752 | 451.26373 | 215.19920 | 2.00621 | 10503 | 1 | 1 | [2](http://www.mycompoundid.org/mycompoundid_IsoMS/myid_search_res/1481082404607-2016-12-6-8-46-44-116748/1481082404756-624-215.1992045-Neutral.htm) |
| 118 | 372.65 | 576.21203 | 578.21794 | 342.15371 | 2.00592 | 13803 | 1 | 1 | [11](http://www.mycompoundid.org/mycompoundid_IsoMS/myid_search_res/1481082404607-2016-12-6-8-46-44-116748/1481082404757-402-342.1537051-Neutral.htm) |
| 119 | 374.81 | 365.11747 | 367.12385 | 131.05915 | 2.00639 | 4229414 | 1 | 1 | [60](http://www.mycompoundid.org/mycompoundid_IsoMS/myid_search_res/1481082404607-2016-12-6-8-46-44-116748/1481082404758-758-131.0591474-Neutral.htm) |
| 120 | 378.65 | 353.11761 | 355.12394 | 119.05929 | 2.00633 | 2992813 | 1 | 1 | [35](http://www.mycompoundid.org/mycompoundid_IsoMS/myid_search_res/1481082404607-2016-12-6-8-46-44-116748/1481082404760-566-119.0592869-Neutral.htm) |
| 121 | 379.30 | 292.11189 | 294.11810 | 58.05357 | 2.00621 | 263000 | 1 | 1 | [1](http://www.mycompoundid.org/mycompoundid_IsoMS/myid_search_res/1481082404607-2016-12-6-8-46-44-116748/1481082404761-333-58.053571-Neutral.htm) |
| 122 | 379.46 | 478.16426 | 480.16989 | 244.10594 | 2.00562 | 7360 | 1 | 1 | [15](http://www.mycompoundid.org/mycompoundid_IsoMS/myid_search_res/1481082404607-2016-12-6-8-46-44-116748/1481082404762-245-244.105941-Neutral.htm) |
| 123 | 381.46 | 348.10231 | 350.10893 | 114.04399 | 2.00662 | 725734 | 1 | 1 | [12](http://www.mycompoundid.org/mycompoundid_IsoMS/myid_search_res/1481082404607-2016-12-6-8-46-44-116748/1481082404762-259-114.0439913-Neutral.htm) |
| 124 | 388.10 | 462.16913 | 464.17576 | 228.11081 | 2.00663 | 12300 | 1 | 1 | [8](http://www.mycompoundid.org/mycompoundid_IsoMS/myid_search_res/1481082404607-2016-12-6-8-46-44-116748/1481082404763-283-228.110813-Neutral.htm) |
| 125 | 388.42 | 417.15819 | 419.16465 | 183.09987 | 2.00646 | 29669 | 1 | 1 | [8](http://www.mycompoundid.org/mycompoundid_IsoMS/myid_search_res/1481082404607-2016-12-6-8-46-44-116748/1481082404764-505-183.0998656-Neutral.htm) |
| 126 | 388.72 | 519.19061 | 521.19662 | 285.13229 | 2.00600 | 22688 | 1 | 1 | [9](http://www.mycompoundid.org/mycompoundid_IsoMS/myid_search_res/1481082404607-2016-12-6-8-46-44-116748/1481082404764-996-285.1322928-Neutral.htm) |
| 127 | 389.47 | 290.56655 | 292.57175 | 113.01646 | 2.00520 | 15850 | 2 | 2 | [1](http://www.mycompoundid.org/mycompoundid_IsoMS/myid_search_res/1481082404607-2016-12-6-8-46-44-116748/1481082404765-959-113.016456-Neutral.htm) |
| 128 | 390.47 | 319.58780 | 321.59381 | 171.05896 | 2.00601 | 18225 | 2 | 2 | [1](http://www.mycompoundid.org/mycompoundid_IsoMS/myid_search_res/1481082404607-2016-12-6-8-46-44-116748/1481082404766-273-171.058964-Neutral.htm) |
| 129 | 393.56 | 555.15756 | 557.16224 | 321.09924 | 2.00468 | 27994 | 1 | 1 | [9](http://www.mycompoundid.org/mycompoundid_IsoMS/myid_search_res/1481082404607-2016-12-6-8-46-44-116748/1481082404767-625-321.0992412-Neutral.htm) |
| 130 | 395.40 | 373.14270 | 375.14998 | 139.08438 | 2.00728 | 12800 | 1 | 1 | [2](http://www.mycompoundid.org/mycompoundid_IsoMS/myid_search_res/1481082404607-2016-12-6-8-46-44-116748/1481082404767-177-139.0843845-Neutral.htm) |
| 131 | 397.93 | 348.10208 | 350.10861 | 114.04376 | 2.00653 | 541836 | 1 | 1 | [12](http://www.mycompoundid.org/mycompoundid_IsoMS/myid_search_res/1481082404607-2016-12-6-8-46-44-116748/1481082404768-789-114.0437621-Neutral.htm) |
| 132 | 398.11 | 587.12937 | 589.13618 | 353.07105 | 2.00681 | 94400 | 1 | 1 | [4](http://www.mycompoundid.org/mycompoundid_IsoMS/myid_search_res/1481082404607-2016-12-6-8-46-44-116748/1481082404769-434-353.071047-Neutral.htm) |
| 133 | 399.69 | 290.56659 | 292.57278 | 113.01654 | 2.00619 | 10397 | 2 | 2 | [1](http://www.mycompoundid.org/mycompoundid_IsoMS/myid_search_res/1481082404607-2016-12-6-8-46-44-116748/1481082404769-845-113.0165409-Neutral.htm) |
| 134 | 399.93 | 379.13280 | 381.13921 | 145.07448 | 2.00641 | 215625 | 1 | 1 | [54](http://www.mycompoundid.org/mycompoundid_IsoMS/myid_search_res/1481082404607-2016-12-6-8-46-44-116748/1481082404769-719-145.0744759-Neutral.htm) |
| 135 | 402.54 | 422.18553 | 424.19106 | 188.12721 | 2.00553 | 11240 | 1 | 1 | [7](http://www.mycompoundid.org/mycompoundid_IsoMS/myid_search_res/1481082404607-2016-12-6-8-46-44-116748/1481082404771-787-188.1272098-Neutral.htm) |
| 136 | 406.94 | 319.58814 | 321.59383 | 171.05964 | 2.00569 | 12450 | 2 | 2 | [1](http://www.mycompoundid.org/mycompoundid_IsoMS/myid_search_res/1481082404607-2016-12-6-8-46-44-116748/1481082404772-227-171.059637-Neutral.htm) |
| 137 | 407.85 | 387.12216 | 389.12880 | 153.06384 | 2.00664 | 26000 | 1 | 1 | [10](http://www.mycompoundid.org/mycompoundid_IsoMS/myid_search_res/1481082404607-2016-12-6-8-46-44-116748/1481082404773-444-153.063837-Neutral.htm) |
| 138 | 408.69 | 519.19132 | 521.19627 | 285.13300 | 2.00495 | 21717 | 1 | 1 | [10](http://www.mycompoundid.org/mycompoundid_IsoMS/myid_search_res/1481082404607-2016-12-6-8-46-44-116748/1481082404773-970-285.1329982-Neutral.htm) |
| 139 | 413.02 | 647.24939 | 649.25565 | 413.19107 | 2.00625 | 8815 | 1 | 1 | [6](http://www.mycompoundid.org/mycompoundid_IsoMS/myid_search_res/1481082404607-2016-12-6-8-46-44-116748/1481082404774-875-413.1910735-Neutral.htm) |
| 140 | 415.11 | 411.14062 | 413.14637 | 177.08230 | 2.00576 | 27588 | 1 | 1 | [6](http://www.mycompoundid.org/mycompoundid_IsoMS/myid_search_res/1481082404607-2016-12-6-8-46-44-116748/1481082404775-840-177.0822952-Neutral.htm) |
| # | RT (s) | mz_light | mz_heavy | mz | distance | int_light | nCharge | nTag | Possible hits |
| 141 | 415.48 | 353.11755 | 355.12423 | 119.05923 | 2.00668 | 2729063 | 1 | 1 | [35](http://www.mycompoundid.org/mycompoundid_IsoMS/myid_search_res/1481082404607-2016-12-6-8-46-44-116748/1481082404775-911-119.0592332-Neutral.htm) |
| 142 | 420.86 | 431.17413 | 433.17983 | 197.11581 | 2.00570 | 15038 | 1 | 1 | [3](http://www.mycompoundid.org/mycompoundid_IsoMS/myid_search_res/1481082404607-2016-12-6-8-46-44-116748/1481082404778-641-197.115814-Neutral.htm) |
| 143 | 421.02 | 295.11164 | 297.11822 | 61.05332 | 2.00658 | 5788438 | 1 | 1 | [15](http://www.mycompoundid.org/mycompoundid_IsoMS/myid_search_res/1481082404607-2016-12-6-8-46-44-116748/1481082404779-278-61.05331731-Neutral.htm) |
| 144 | 421.38 | 462.17009 | 464.17678 | 228.11177 | 2.00668 | 296900 | 1 | 1 | [8](http://www.mycompoundid.org/mycompoundid_IsoMS/myid_search_res/1481082404607-2016-12-6-8-46-44-116748/1481082404780-837-228.1117708-Neutral.htm) |
| 145 | 421.46 | 405.15862 | 407.16505 | 171.10030 | 2.00644 | 14600 | 1 | 1 | [6](http://www.mycompoundid.org/mycompoundid_IsoMS/myid_search_res/1481082404607-2016-12-6-8-46-44-116748/1481082404780-666-171.100295-Neutral.htm) |
| 146 | 421.87 | 673.26040 | 675.27058 | 439.20208 | 2.01018 | 23475 | 1 | 1 | [5](http://www.mycompoundid.org/mycompoundid_IsoMS/myid_search_res/1481082404607-2016-12-6-8-46-44-116748/1481082404781-673-439.2020833-Neutral.htm) |
| 147 | 424.37 | 395.12736 | 397.13316 | 161.06904 | 2.00580 | 20125 | 1 | 1 | [48](http://www.mycompoundid.org/mycompoundid_IsoMS/myid_search_res/1481082404607-2016-12-6-8-46-44-116748/1481082404781-698-161.0690364-Neutral.htm) |
| 148 | 424.86 | 323.10599 | 325.11256 | 89.04767 | 2.00657 | 39700 | 1 | 1 | [38](http://www.mycompoundid.org/mycompoundid_IsoMS/myid_search_res/1481082404607-2016-12-6-8-46-44-116748/1481082404783-730-89.04767-Neutral.htm) |
| 149 | 427.87 | 625.19888 | 627.20489 | 391.14056 | 2.00602 | 12600 | 1 | 1 | [21](http://www.mycompoundid.org/mycompoundid_IsoMS/myid_search_res/1481082404607-2016-12-6-8-46-44-116748/1481082404785-265-391.140556-Neutral.htm) |
| 150 | 429.28 | 363.14903 | 365.15467 | 129.09071 | 2.00563 | 298375 | 1 | 1 | [4](http://www.mycompoundid.org/mycompoundid_IsoMS/myid_search_res/1481082404607-2016-12-6-8-46-44-116748/1481082404786-863-129.0907129-Neutral.htm) |
| 151 | 430.94 | 555.15792 | 557.16372 | 321.09960 | 2.00580 | 13672 | 1 | 1 | [8](http://www.mycompoundid.org/mycompoundid_IsoMS/myid_search_res/1481082404607-2016-12-6-8-46-44-116748/1481082404786-598-321.099598-Neutral.htm) |
| 152 | 433.15 | 491.19429 | 493.20221 | 257.13597 | 2.00792 | 16100 | 1 | 1 | [6](http://www.mycompoundid.org/mycompoundid_IsoMS/myid_search_res/1481082404607-2016-12-6-8-46-44-116748/1481082404787-183-257.1359733-Neutral.htm) |
| 153 | 433.20 | 505.22178 | 507.22515 | 271.16346 | 2.00336 | 20065 | 1 | 1 | [1](http://www.mycompoundid.org/mycompoundid_IsoMS/myid_search_res/1481082404607-2016-12-6-8-46-44-116748/1481082404787-389-271.1634621-Neutral.htm) |
| 154 | 433.40 | 479.23225 | 481.23903 | 245.17393 | 2.00678 | 245810 | 1 | 1 | [6](http://www.mycompoundid.org/mycompoundid_IsoMS/myid_search_res/1481082404607-2016-12-6-8-46-44-116748/1481082404788-978-245.1739313-Neutral.htm) |
| 155 | 439.02 | 600.20052 | 602.20699 | 366.14220 | 2.00646 | 10503 | 1 | 1 | [11](http://www.mycompoundid.org/mycompoundid_IsoMS/myid_search_res/1481082404607-2016-12-6-8-46-44-116748/1481082404788-938-366.142204-Neutral.htm) |
| 156 | 445.30 | 362.11810 | 364.12644 | 128.05978 | 2.00834 | 1001738 | 1 | 1 | [16](http://www.mycompoundid.org/mycompoundid_IsoMS/myid_search_res/1481082404607-2016-12-6-8-46-44-116748/1481082404789-147-128.0597813-Neutral.htm) |
| 157 | 447.57 | 462.17023 | 464.17701 | 228.11191 | 2.00678 | 545286 | 1 | 1 | [8](http://www.mycompoundid.org/mycompoundid_IsoMS/myid_search_res/1481082404607-2016-12-6-8-46-44-116748/1481082404790-93-228.1119117-Neutral.htm) |
| 158 | 448.02 | 303.11583 | 305.12255 | 69.05751 | 2.00671 | 9495 | 1 | 1 | [2](http://www.mycompoundid.org/mycompoundid_IsoMS/myid_search_res/1481082404607-2016-12-6-8-46-44-116748/1481082404790-857-69.0575135-Neutral.htm) |
| 159 | 448.90 | 477.18100 | 479.18642 | 243.12268 | 2.00541 | 33975 | 1 | 1 | [8](http://www.mycompoundid.org/mycompoundid_IsoMS/myid_search_res/1481082404607-2016-12-6-8-46-44-116748/1481082404791-882-243.1226826-Neutral.htm) |
| 160 | 449.16 | 279.07998 | 281.08641 | 45.02166 | 2.00643 | 57400 | 1 | 1 | [8](http://www.mycompoundid.org/mycompoundid_IsoMS/myid_search_res/1481082404607-2016-12-6-8-46-44-116748/1481082404791-317-45.021662-Neutral.htm) |
| 161 | 450.73 | 334.12235 | 336.12878 | 100.06403 | 2.00643 | 374194 | 1 | 1 | [3](http://www.mycompoundid.org/mycompoundid_IsoMS/myid_search_res/1481082404607-2016-12-6-8-46-44-116748/1481082404792-770-100.0640326-Neutral.htm) |
| 162 | 452.34 | 323.10658 | 325.11269 | 89.04826 | 2.00611 | 208720 | 1 | 1 | [38](http://www.mycompoundid.org/mycompoundid_IsoMS/myid_search_res/1481082404607-2016-12-6-8-46-44-116748/1481082404792-120-89.04825863-Neutral.htm) |
| 163 | 453.89 | 587.12965 | 589.13567 | 353.07133 | 2.00602 | 102000 | 1 | 1 | [6](http://www.mycompoundid.org/mycompoundid_IsoMS/myid_search_res/1481082404607-2016-12-6-8-46-44-116748/1481082404794-160-353.071334-Neutral.htm) |
| 164 | 453.95 | 350.15306 | 352.15991 | 116.09474 | 2.00684 | 269000 | 1 | 1 | [9](http://www.mycompoundid.org/mycompoundid_IsoMS/myid_search_res/1481082404607-2016-12-6-8-46-44-116748/1481082404794-587-116.094742-Neutral.htm) |
| # | RT (s) | mz_light | mz_heavy | mz | distance | int_light | nCharge | nTag | Possible hits |
| 165 | 454.49 | 491.19474 | 493.20177 | 257.13642 | 2.00703 | 23984 | 1 | 1 | [6](http://www.mycompoundid.org/mycompoundid_IsoMS/myid_search_res/1481082404607-2016-12-6-8-46-44-116748/1481082404795-218-257.1364207-Neutral.htm) |
| 166 | 455.15 | 422.18630 | 424.19207 | 188.12798 | 2.00576 | 216875 | 1 | 1 | [7](http://www.mycompoundid.org/mycompoundid_IsoMS/myid_search_res/1481082404607-2016-12-6-8-46-44-116748/1481082404795-128-188.1279828-Neutral.htm) |
| 167 | 456.17 | 378.22080 | 380.22800 | 144.16248 | 2.00720 | 28700 | 1 | 1 | [2](http://www.mycompoundid.org/mycompoundid_IsoMS/myid_search_res/1481082404607-2016-12-6-8-46-44-116748/1481082404796-788-144.1624793-Neutral.htm) |
| 168 | 456.67 | 436.15357 | 438.16042 | 202.09525 | 2.00685 | 11839 | 1 | 1 | [19](http://www.mycompoundid.org/mycompoundid_IsoMS/myid_search_res/1481082404607-2016-12-6-8-46-44-116748/1481082404796-170-202.095247-Neutral.htm) |
| 169 | 458.73 | 337.15906 | 339.16563 | 103.10074 | 2.00657 | 3258477 | 1 | 1 | [5](http://www.mycompoundid.org/mycompoundid_IsoMS/myid_search_res/1481082404607-2016-12-6-8-46-44-116748/1481082404797-980-103.1007362-Neutral.htm) |
| 170 | 461.16 | 789.30865 | 791.31321 | 555.25033 | 2.00457 | 27581 | 1 | 1 | [3](http://www.mycompoundid.org/mycompoundid_IsoMS/myid_search_res/1481082404607-2016-12-6-8-46-44-116748/1481082404798-768-555.2503252-Neutral.htm) |
| 171 | 462.18 | 787.30244 | 789.30771 | 553.24412 | 2.00528 | 66400 | 1 | 1 | [4](http://www.mycompoundid.org/mycompoundid_IsoMS/myid_search_res/1481082404607-2016-12-6-8-46-44-116748/1481082404798-635-553.244116-Neutral.htm) |
| 172 | 462.69 | 348.10128 | 350.10738 | 114.04296 | 2.00610 | 108031 | 1 | 1 | [12](http://www.mycompoundid.org/mycompoundid_IsoMS/myid_search_res/1481082404607-2016-12-6-8-46-44-116748/1481082404799-96-114.0429616-Neutral.htm) |
| 173 | 466.48 | 394.14419 | 396.15015 | 160.08587 | 2.00596 | 555063 | 1 | 1 | [35](http://www.mycompoundid.org/mycompoundid_IsoMS/myid_search_res/1481082404607-2016-12-6-8-46-44-116748/1481082404799-92-160.0858739-Neutral.htm) |
| 174 | 470.91 | 450.16884 | 452.17380 | 216.11052 | 2.00496 | 15625 | 1 | 1 | [15](http://www.mycompoundid.org/mycompoundid_IsoMS/myid_search_res/1481082404607-2016-12-6-8-46-44-116748/1481082404801-883-216.1105198-Neutral.htm) |
| 175 | 471.48 | 600.20092 | 602.20808 | 366.14260 | 2.00716 | 17388 | 1 | 1 | [12](http://www.mycompoundid.org/mycompoundid_IsoMS/myid_search_res/1481082404607-2016-12-6-8-46-44-116748/1481082404802-76-366.1425955-Neutral.htm) |
| 176 | 473.45 | 463.23755 | 465.24337 | 229.17923 | 2.00581 | 32647 | 1 | 1 | [2](http://www.mycompoundid.org/mycompoundid_IsoMS/myid_search_res/1481082404607-2016-12-6-8-46-44-116748/1481082404802-411-229.1792315-Neutral.htm) |
| 177 | 474.92 | 539.21007 | 541.21466 | 305.15175 | 2.00459 | 9565 | 1 | 1 | [8](http://www.mycompoundid.org/mycompoundid_IsoMS/myid_search_res/1481082404607-2016-12-6-8-46-44-116748/1481082404803-291-305.151749-Neutral.htm) |
| 178 | 478.71 | 436.15412 | 438.15983 | 202.09580 | 2.00571 | 15388 | 1 | 1 | [19](http://www.mycompoundid.org/mycompoundid_IsoMS/myid_search_res/1481082404607-2016-12-6-8-46-44-116748/1481082404803-515-202.0957961-Neutral.htm) |
| 179 | 479.43 | 436.22513 | 438.23001 | 202.16681 | 2.00488 | 10288 | 1 | 1 | [3](http://www.mycompoundid.org/mycompoundid_IsoMS/myid_search_res/1481082404607-2016-12-6-8-46-44-116748/1481082404804-690-202.1668063-Neutral.htm) |
| 180 | 483.12 | 351.13712 | 353.14330 | 117.07880 | 2.00618 | 30788 | 1 | 1 | [38](http://www.mycompoundid.org/mycompoundid_IsoMS/myid_search_res/1481082404607-2016-12-6-8-46-44-116748/1481082404805-596-117.0788002-Neutral.htm) |
| 181 | 493.83 | 483.14478 | 485.14973 | 249.08646 | 2.00496 | 20163 | 1 | 1 | [41](http://www.mycompoundid.org/mycompoundid_IsoMS/myid_search_res/1481082404607-2016-12-6-8-46-44-116748/1481082404807-968-249.086458-Neutral.htm) |
| 182 | 496.63 | 431.17472 | 433.18042 | 197.11640 | 2.00570 | 12825 | 1 | 1 | [3](http://www.mycompoundid.org/mycompoundid_IsoMS/myid_search_res/1481082404607-2016-12-6-8-46-44-116748/1481082404808-808-197.116398-Neutral.htm) |
| 183 | 500.00 | 413.11983 | 415.12594 | 179.06151 | 2.00611 | 37131 | 1 | 1 | [11](http://www.mycompoundid.org/mycompoundid_IsoMS/myid_search_res/1481082404607-2016-12-6-8-46-44-116748/1481082404809-864-179.0615068-Neutral.htm) |
| 184 | 504.20 | 406.14277 | 408.14745 | 172.08445 | 2.00468 | 28370 | 1 | 1 | [9](http://www.mycompoundid.org/mycompoundid_IsoMS/myid_search_res/1481082404607-2016-12-6-8-46-44-116748/1481082404810-698-172.084452-Neutral.htm) |
| 185 | 505.02 | 337.12174 | 339.12804 | 103.06342 | 2.00631 | 53450 | 1 | 1 | [45](http://www.mycompoundid.org/mycompoundid_IsoMS/myid_search_res/1481082404607-2016-12-6-8-46-44-116748/1481082404810-187-103.0634165-Neutral.htm) |
| 186 | 508.30 | 353.11728 | 355.12351 | 119.05896 | 2.00623 | 416250 | 1 | 1 | [35](http://www.mycompoundid.org/mycompoundid_IsoMS/myid_search_res/1481082404607-2016-12-6-8-46-44-116748/1481082404813-498-119.0589649-Neutral.htm) |
| 187 | 509.37 | 361.19461 | 363.20096 | 127.13629 | 2.00635 | 96453 | 1 | 1 | [1](http://www.mycompoundid.org/mycompoundid_IsoMS/myid_search_res/1481082404607-2016-12-6-8-46-44-116748/1481082404814-928-127.1362871-Neutral.htm) |
| 188 | 510.88 | 587.12257 | 589.13433 | 353.06425 | 2.01176 | 8330 | 1 | 1 | [28](http://www.mycompoundid.org/mycompoundid_IsoMS/myid_search_res/1481082404607-2016-12-6-8-46-44-116748/1481082404815-494-353.064248-Neutral.htm) |
| # | RT (s) | mz_light | mz_heavy | mz | distance | int_light | nCharge | nTag | Possible hits |
| 189 | 511.82 | 531.14922 | 533.15339 | 297.09090 | 2.00418 | 12890 | 1 | 1 | [10](http://www.mycompoundid.org/mycompoundid_IsoMS/myid_search_res/1481082404607-2016-12-6-8-46-44-116748/1481082404816-26-297.090895-Neutral.htm) |
| 190 | 512.08 | 478.16402 | 480.17014 | 244.10570 | 2.00611 | 19550 | 1 | 1 | [15](http://www.mycompoundid.org/mycompoundid_IsoMS/myid_search_res/1481082404607-2016-12-6-8-46-44-116748/1481082404817-193-244.1057043-Neutral.htm) |
| 191 | 512.43 | 367.13185 | 369.13840 | 133.07353 | 2.00655 | 16581 | 1 | 1 | [40](http://www.mycompoundid.org/mycompoundid_IsoMS/myid_search_res/1481082404607-2016-12-6-8-46-44-116748/1481082404817-412-133.0735296-Neutral.htm) |
| 192 | 514.37 | 392.16390 | 394.17012 | 158.10558 | 2.00622 | 30991 | 1 | 1 | [6](http://www.mycompoundid.org/mycompoundid_IsoMS/myid_search_res/1481082404607-2016-12-6-8-46-44-116748/1481082404819-668-158.105581-Neutral.htm) |
| 193 | 516.31 | 503.19628 | 505.20026 | 269.13796 | 2.00398 | 10105 | 1 | 1 | [8](http://www.mycompoundid.org/mycompoundid_IsoMS/myid_search_res/1481082404607-2016-12-6-8-46-44-116748/1481082404820-263-269.1379625-Neutral.htm) |
| 194 | 516.31 | 395.12676 | 397.13366 | 161.06844 | 2.00690 | 13638 | 1 | 1 | [46](http://www.mycompoundid.org/mycompoundid_IsoMS/myid_search_res/1481082404607-2016-12-6-8-46-44-116748/1481082404820-184-161.0684375-Neutral.htm) |
| 195 | 523.10 | 625.19991 | 627.20721 | 391.14159 | 2.00730 | 12050 | 1 | 1 | [22](http://www.mycompoundid.org/mycompoundid_IsoMS/myid_search_res/1481082404607-2016-12-6-8-46-44-116748/1481082404822-367-391.141589-Neutral.htm) |
| 196 | 523.74 | 476.22200 | 478.22800 | 242.16368 | 2.00600 | 20650 | 1 | 1 | [4](http://www.mycompoundid.org/mycompoundid_IsoMS/myid_search_res/1481082404607-2016-12-6-8-46-44-116748/1481082404823-744-242.1636848-Neutral.htm) |
| 197 | 523.80 | 450.16974 | 452.17552 | 216.11142 | 2.00578 | 14538 | 1 | 1 | [15](http://www.mycompoundid.org/mycompoundid_IsoMS/myid_search_res/1481082404607-2016-12-6-8-46-44-116748/1481082404823-544-216.1114235-Neutral.htm) |
| 198 | 526.08 | 406.14310 | 408.14897 | 172.08478 | 2.00587 | 42838 | 1 | 1 | [9](http://www.mycompoundid.org/mycompoundid_IsoMS/myid_search_res/1481082404607-2016-12-6-8-46-44-116748/1481082404824-219-172.084778-Neutral.htm) |
| 199 | 526.29 | 408.15879 | 410.16518 | 174.10047 | 2.00639 | 85094 | 1 | 1 | [35](http://www.mycompoundid.org/mycompoundid_IsoMS/myid_search_res/1481082404607-2016-12-6-8-46-44-116748/1481082404824-310-174.1004675-Neutral.htm) |
| 200 | 528.64 | 351.13765 | 353.14416 | 117.07933 | 2.00651 | 221750 | 1 | 1 | [38](http://www.mycompoundid.org/mycompoundid_IsoMS/myid_search_res/1481082404607-2016-12-6-8-46-44-116748/1481082404827-80-117.0793303-Neutral.htm) |
| 201 | 528.76 | 587.12950 | 589.13479 | 353.07118 | 2.00529 | 24994 | 1 | 1 | [6](http://www.mycompoundid.org/mycompoundid_IsoMS/myid_search_res/1481082404607-2016-12-6-8-46-44-116748/1481082404829-979-353.0711818-Neutral.htm) |
| 202 | 530.33 | 381.11275 | 383.11933 | 147.05443 | 2.00658 | 1034250 | 1 | 1 | [55](http://www.mycompoundid.org/mycompoundid_IsoMS/myid_search_res/1481082404607-2016-12-6-8-46-44-116748/1481082404829-291-147.0544251-Neutral.htm) |
| 203 | 532.04 | 503.19910 | 505.20247 | 269.14078 | 2.00337 | 12200 | 1 | 1 | [1](http://www.mycompoundid.org/mycompoundid_IsoMS/myid_search_res/1481082404607-2016-12-6-8-46-44-116748/1481082404831-614-269.140776-Neutral.htm) |
| 204 | 532.98 | 363.10161 | 365.10799 | 129.04329 | 2.00637 | 4520000 | 1 | 1 | [28](http://www.mycompoundid.org/mycompoundid_IsoMS/myid_search_res/1481082404607-2016-12-6-8-46-44-116748/1481082404831-501-129.043293-Neutral.htm) |
| 205 | 535.65 | 517.14990 | 519.15657 | 283.09158 | 2.00667 | 18878 | 1 | 1 | [38](http://www.mycompoundid.org/mycompoundid_IsoMS/myid_search_res/1481082404607-2016-12-6-8-46-44-116748/1481082404832-394-283.0915779-Neutral.htm) |
| 206 | 539.26 | 450.17027 | 452.17599 | 216.11195 | 2.00573 | 21500 | 1 | 1 | [15](http://www.mycompoundid.org/mycompoundid_IsoMS/myid_search_res/1481082404607-2016-12-6-8-46-44-116748/1481082404833-254-216.111949-Neutral.htm) |
| 207 | 541.82 | 578.21176 | 580.21804 | 344.15344 | 2.00629 | 26750 | 1 | 1 | [1](http://www.mycompoundid.org/mycompoundid_IsoMS/myid_search_res/1481082404607-2016-12-6-8-46-44-116748/1481082404834-128-344.153438-Neutral.htm) |
| 208 | 542.90 | 478.12954 | 480.13613 | 244.07122 | 2.00659 | 2737188 | 1 | 1 | [31](http://www.mycompoundid.org/mycompoundid_IsoMS/myid_search_res/1481082404607-2016-12-6-8-46-44-116748/1481082404835-113-244.0712195-Neutral.htm) |
| 209 | 543.40 | 366.10158 | 368.10735 | 132.04326 | 2.00578 | 128463 | 1 | 1 | [69](http://www.mycompoundid.org/mycompoundid_IsoMS/myid_search_res/1481082404607-2016-12-6-8-46-44-116748/1481082404836-643-132.0432552-Neutral.htm) |
| 210 | 547.71 | 502.13779 | 504.14503 | 268.07947 | 2.00724 | 16053 | 1 | 1 | [20](http://www.mycompoundid.org/mycompoundid_IsoMS/myid_search_res/1481082404607-2016-12-6-8-46-44-116748/1481082404839-699-268.079466-Neutral.htm) |
| 211 | 547.86 | 535.17745 | 537.18634 | 301.11913 | 2.00889 | 14325 | 1 | 1 | [15](http://www.mycompoundid.org/mycompoundid_IsoMS/myid_search_res/1481082404607-2016-12-6-8-46-44-116748/1481082404840-832-301.1191295-Neutral.htm) |
| 212 | 547.94 | 385.10861 | 387.11500 | 151.05029 | 2.00639 | 732813 | 1 | 1 | [11](http://www.mycompoundid.org/mycompoundid_IsoMS/myid_search_res/1481082404607-2016-12-6-8-46-44-116748/1481082404840-515-151.0502903-Neutral.htm) |
| # | RT (s) | mz_light | mz_heavy | mz | distance | int_light | nCharge | nTag | Possible hits |
| 213 | 552.27 | 587.12946 | 589.13574 | 353.07114 | 2.00628 | 156000 | 1 | 1 | [4](http://www.mycompoundid.org/mycompoundid_IsoMS/myid_search_res/1481082404607-2016-12-6-8-46-44-116748/1481082404841-422-353.071142-Neutral.htm) |
| 214 | 553.31 | 344.10654 | 346.11273 | 110.04822 | 2.00619 | 56450 | 1 | 1 | [8](http://www.mycompoundid.org/mycompoundid_IsoMS/myid_search_res/1481082404607-2016-12-6-8-46-44-116748/1481082404842-265-110.0482187-Neutral.htm) |
| 215 | 554.14 | 558.19607 | 560.20463 | 324.13775 | 2.00856 | 10245 | 1 | 1 | [5](http://www.mycompoundid.org/mycompoundid_IsoMS/myid_search_res/1481082404607-2016-12-6-8-46-44-116748/1481082404842-869-324.1377515-Neutral.htm) |
| 216 | 555.42 | 367.13277 | 369.13812 | 133.07445 | 2.00535 | 16944 | 1 | 1 | [40](http://www.mycompoundid.org/mycompoundid_IsoMS/myid_search_res/1481082404607-2016-12-6-8-46-44-116748/1481082404843-448-133.0744536-Neutral.htm) |
| 217 | 556.07 | 363.10205 | 365.10843 | 129.04373 | 2.00638 | 6634063 | 1 | 1 | [28](http://www.mycompoundid.org/mycompoundid_IsoMS/myid_search_res/1481082404607-2016-12-6-8-46-44-116748/1481082404844-688-129.0437311-Neutral.htm) |
| 218 | 558.30 | 395.12784 | 397.13457 | 161.06952 | 2.00672 | 7980000 | 1 | 1 | [48](http://www.mycompoundid.org/mycompoundid_IsoMS/myid_search_res/1481082404607-2016-12-6-8-46-44-116748/1481082404845-58-161.069524-Neutral.htm) |
| 219 | 559.32 | 463.23679 | 465.24223 | 229.17847 | 2.00544 | 10650 | 1 | 1 | [2](http://www.mycompoundid.org/mycompoundid_IsoMS/myid_search_res/1481082404607-2016-12-6-8-46-44-116748/1481082404847-506-229.17847-Neutral.htm) |
| 220 | 560.51 | 513.18949 | 515.19453 | 279.13117 | 2.00504 | 11713 | 1 | 1 | [15](http://www.mycompoundid.org/mycompoundid_IsoMS/myid_search_res/1481082404607-2016-12-6-8-46-44-116748/1481082404847-484-279.1311668-Neutral.htm) |
| 221 | 561.38 | 362.11731 | 364.12320 | 128.05899 | 2.00589 | 138625 | 1 | 1 | [16](http://www.mycompoundid.org/mycompoundid_IsoMS/myid_search_res/1481082404607-2016-12-6-8-46-44-116748/1481082404848-743-128.0589908-Neutral.htm) |
| 222 | 563.01 | 732.33810 | 734.34289 | 498.27978 | 2.00480 | 17800 | 1 | 1 | [33](http://www.mycompoundid.org/mycompoundid_IsoMS/myid_search_res/1481082404607-2016-12-6-8-46-44-116748/1481082404848-428-498.279776-Neutral.htm) |
| 223 | 564.36 | 549.19295 | 551.19793 | 315.13463 | 2.00498 | 11139 | 1 | 1 | [13](http://www.mycompoundid.org/mycompoundid_IsoMS/myid_search_res/1481082404607-2016-12-6-8-46-44-116748/1481082404850-261-315.1346309-Neutral.htm) |
| 224 | 565.43 | 309.12698 | 311.13281 | 75.06866 | 2.00583 | 35750 | 1 | 1 | [13](http://www.mycompoundid.org/mycompoundid_IsoMS/myid_search_res/1481082404607-2016-12-6-8-46-44-116748/1481082404850-186-75.06865906-Neutral.htm) |
| 225 | 574.29 | 321.09117 | 323.09805 | 87.03285 | 2.00688 | 24000 | 1 | 1 | [18](http://www.mycompoundid.org/mycompoundid_IsoMS/myid_search_res/1481082404607-2016-12-6-8-46-44-116748/1481082404851-973-87.032852-Neutral.htm) |
| 226 | 579.10 | 438.16918 | 440.17402 | 204.11086 | 2.00484 | 8060 | 1 | 1 | [11](http://www.mycompoundid.org/mycompoundid_IsoMS/myid_search_res/1481082404607-2016-12-6-8-46-44-116748/1481082404853-323-204.11086-Neutral.htm) |
| 227 | 579.77 | 363.10184 | 365.10809 | 129.04352 | 2.00625 | 4109891 | 1 | 1 | [28](http://www.mycompoundid.org/mycompoundid_IsoMS/myid_search_res/1481082404607-2016-12-6-8-46-44-116748/1481082404853-28-129.0435195-Neutral.htm) |
| 228 | 581.51 | 551.17596 | 553.18254 | 317.11764 | 2.00659 | 99375 | 1 | 1 | [3](http://www.mycompoundid.org/mycompoundid_IsoMS/myid_search_res/1481082404607-2016-12-6-8-46-44-116748/1481082404854-813-317.1176358-Neutral.htm) |
| 229 | 584.85 | 476.18510 | 478.19128 | 242.12678 | 2.00618 | 141875 | 1 | 1 | [6](http://www.mycompoundid.org/mycompoundid_IsoMS/myid_search_res/1481082404607-2016-12-6-8-46-44-116748/1481082404855-143-242.1267814-Neutral.htm) |
| 230 | 584.94 | 569.17431 | 571.17938 | 335.11599 | 2.00507 | 13688 | 1 | 1 | [9](http://www.mycompoundid.org/mycompoundid_IsoMS/myid_search_res/1481082404607-2016-12-6-8-46-44-116748/1481082404855-687-335.1159931-Neutral.htm) |
| 231 | 585.03 | 578.21321 | 580.21622 | 344.15489 | 2.00300 | 11700 | 1 | 1 | [1](http://www.mycompoundid.org/mycompoundid_IsoMS/myid_search_res/1481082404607-2016-12-6-8-46-44-116748/1481082404856-787-344.154894-Neutral.htm) |
| 232 | 588.15 | 436.20103 | 438.20623 | 202.14271 | 2.00520 | 94138 | 1 | 1 | [4](http://www.mycompoundid.org/mycompoundid_IsoMS/myid_search_res/1481082404607-2016-12-6-8-46-44-116748/1481082404857-434-202.142708-Neutral.htm) |
| 233 | 592.53 | 453.16876 | 455.17427 | 219.11044 | 2.00551 | 11355 | 1 | 1 | [18](http://www.mycompoundid.org/mycompoundid_IsoMS/myid_search_res/1481082404607-2016-12-6-8-46-44-116748/1481082404857-464-219.110439-Neutral.htm) |
| 234 | 594.09 | 691.16617 | 695.17843 | 224.05683 | 4.01226 | 12338 | 1 | 2 | [2](http://www.mycompoundid.org/mycompoundid_IsoMS/myid_search_res/1481082404607-2016-12-6-8-46-44-116748/1481082404859-511-224.0568263-Neutral.htm) |
| 235 | 594.61 | 363.10185 | 365.10838 | 129.04353 | 2.00652 | 6160379 | 1 | 1 | [28](http://www.mycompoundid.org/mycompoundid_IsoMS/myid_search_res/1481082404607-2016-12-6-8-46-44-116748/1481082404859-639-129.0435312-Neutral.htm) |
| 236 | 595.81 | 481.08373 | 483.08967 | 247.02541 | 2.00594 | 11970 | 1 | 1 | [3](http://www.mycompoundid.org/mycompoundid_IsoMS/myid_search_res/1481082404607-2016-12-6-8-46-44-116748/1481082404860-797-247.0254138-Neutral.htm) |
| # | RT (s) | mz_light | mz_heavy | mz | distance | int_light | nCharge | nTag | Possible hits |
| 237 | 596.07 | 478.20075 | 480.20758 | 244.14243 | 2.00682 | 25897 | 1 | 1 | [6](http://www.mycompoundid.org/mycompoundid_IsoMS/myid_search_res/1481082404607-2016-12-6-8-46-44-116748/1481082404860-149-244.1424347-Neutral.htm) |
| 238 | 598.45 | 671.24212 | 673.24947 | 437.18380 | 2.00734 | 34500 | 1 | 1 | [2](http://www.mycompoundid.org/mycompoundid_IsoMS/myid_search_res/1481082404607-2016-12-6-8-46-44-116748/1481082404862-824-437.1838021-Neutral.htm) |
| 239 | 599.55 | 376.17954 | 378.18633 | 142.12122 | 2.00679 | 29025 | 1 | 1 | [2](http://www.mycompoundid.org/mycompoundid_IsoMS/myid_search_res/1481082404607-2016-12-6-8-46-44-116748/1481082404862-522-142.1212188-Neutral.htm) |
| 240 | 603.78 | 460.11871 | 462.12532 | 226.06039 | 2.00661 | 2648125 | 1 | 1 | [26](http://www.mycompoundid.org/mycompoundid_IsoMS/myid_search_res/1481082404607-2016-12-6-8-46-44-116748/1481082404863-363-226.0603891-Neutral.htm) |
| 241 | 606.93 | 540.13514 | 542.14105 | 306.07682 | 2.00591 | 16914 | 1 | 1 | [4](http://www.mycompoundid.org/mycompoundid_IsoMS/myid_search_res/1481082404607-2016-12-6-8-46-44-116748/1481082404864-633-306.0768196-Neutral.htm) |
| 242 | 607.40 | 337.12161 | 339.12866 | 103.06329 | 2.00705 | 30138 | 1 | 1 | [45](http://www.mycompoundid.org/mycompoundid_IsoMS/myid_search_res/1481082404607-2016-12-6-8-46-44-116748/1481082404864-567-103.0632903-Neutral.htm) |
| 243 | 608.13 | 420.15869 | 422.16536 | 186.10037 | 2.00667 | 36350 | 1 | 1 | [15](http://www.mycompoundid.org/mycompoundid_IsoMS/myid_search_res/1481082404607-2016-12-6-8-46-44-116748/1481082404866-619-186.100368-Neutral.htm) |
| 244 | 608.53 | 379.13319 | 381.13957 | 145.07487 | 2.00638 | 4882500 | 1 | 1 | [54](http://www.mycompoundid.org/mycompoundid_IsoMS/myid_search_res/1481082404607-2016-12-6-8-46-44-116748/1481082404866-448-145.0748726-Neutral.htm) |
| 245 | 613.48 | 366.10172 | 368.10709 | 132.04340 | 2.00537 | 36813 | 1 | 1 | [69](http://www.mycompoundid.org/mycompoundid_IsoMS/myid_search_res/1481082404607-2016-12-6-8-46-44-116748/1481082404868-999-132.0434026-Neutral.htm) |
| 246 | 614.16 | 438.16979 | 440.17505 | 204.11147 | 2.00526 | 32606 | 1 | 1 | [11](http://www.mycompoundid.org/mycompoundid_IsoMS/myid_search_res/1481082404607-2016-12-6-8-46-44-116748/1481082404870-621-204.1114693-Neutral.htm) |
| 247 | 614.43 | 346.08759 | 348.09384 | 224.05853 | 2.00625 | 12625 | 2 | 2 | [3](http://www.mycompoundid.org/mycompoundid_IsoMS/myid_search_res/1481082404607-2016-12-6-8-46-44-116748/1481082404871-507-224.0585325-Neutral.htm) |
| 248 | 617.42 | 478.19988 | 480.20616 | 244.14156 | 2.00628 | 65075 | 1 | 1 | [6](http://www.mycompoundid.org/mycompoundid_IsoMS/myid_search_res/1481082404607-2016-12-6-8-46-44-116748/1481082404871-144-244.1415616-Neutral.htm) |
| 249 | 618.81 | 334.12196 | 336.12847 | 100.06364 | 2.00651 | 39788 | 1 | 1 | [3](http://www.mycompoundid.org/mycompoundid_IsoMS/myid_search_res/1481082404607-2016-12-6-8-46-44-116748/1481082404871-544-100.0636383-Neutral.htm) |
| 250 | 618.83 | 398.12742 | 400.13418 | 164.06910 | 2.00676 | 15704 | 1 | 1 | [74](http://www.mycompoundid.org/mycompoundid_IsoMS/myid_search_res/1481082404607-2016-12-6-8-46-44-116748/1481082404872-147-164.069096-Neutral.htm) |
| 251 | 621.05 | 550.19827 | 552.20489 | 316.13995 | 2.00662 | 214214 | 1 | 1 | [4](http://www.mycompoundid.org/mycompoundid_IsoMS/myid_search_res/1481082404607-2016-12-6-8-46-44-116748/1481082404876-454-316.1399513-Neutral.htm) |
| 252 | 625.50 | 321.12682 | 323.13226 | 87.06850 | 2.00544 | 61700 | 1 | 1 | [29](http://www.mycompoundid.org/mycompoundid_IsoMS/myid_search_res/1481082404607-2016-12-6-8-46-44-116748/1481082404877-136-87.06849775-Neutral.htm) |
| 253 | 625.72 | 571.20509 | 573.21510 | 337.14677 | 2.01001 | 25078 | 1 | 1 | [7](http://www.mycompoundid.org/mycompoundid_IsoMS/myid_search_res/1481082404607-2016-12-6-8-46-44-116748/1481082404878-850-337.146766-Neutral.htm) |
| 254 | 628.03 | 558.19572 | 560.20162 | 324.13740 | 2.00590 | 17055 | 1 | 1 | [5](http://www.mycompoundid.org/mycompoundid_IsoMS/myid_search_res/1481082404607-2016-12-6-8-46-44-116748/1481082404878-682-324.1373998-Neutral.htm) |
| 255 | 628.59 | 399.12352 | 401.13031 | 165.06520 | 2.00679 | 28125 | 1 | 1 | [36](http://www.mycompoundid.org/mycompoundid_IsoMS/myid_search_res/1481082404607-2016-12-6-8-46-44-116748/1481082404879-78-165.0651995-Neutral.htm) |
| 256 | 629.05 | 355.09357 | 357.10015 | 242.07050 | 2.00658 | 848267 | 2 | 2 | [11](http://www.mycompoundid.org/mycompoundid_IsoMS/myid_search_res/1481082404607-2016-12-6-8-46-44-116748/1481082404880-918-242.0705017-Neutral.htm) |
| 257 | 630.08 | 628.22743 | 630.23068 | 394.16911 | 2.00324 | 14600 | 1 | 1 | [4](http://www.mycompoundid.org/mycompoundid_IsoMS/myid_search_res/1481082404607-2016-12-6-8-46-44-116748/1481082404881-414-394.169113-Neutral.htm) |
| 258 | 631.62 | 506.17530 | 508.18207 | 272.11698 | 2.00677 | 14688 | 1 | 1 | [10](http://www.mycompoundid.org/mycompoundid_IsoMS/myid_search_res/1481082404607-2016-12-6-8-46-44-116748/1481082404882-604-272.1169803-Neutral.htm) |
| 259 | 634.25 | 550.19819 | 552.20519 | 316.13987 | 2.00700 | 364500 | 1 | 1 | [4](http://www.mycompoundid.org/mycompoundid_IsoMS/myid_search_res/1481082404607-2016-12-6-8-46-44-116748/1481082404883-871-316.1398675-Neutral.htm) |
| 260 | 634.62 | 540.13524 | 542.14216 | 306.07692 | 2.00692 | 52522 | 1 | 1 | [4](http://www.mycompoundid.org/mycompoundid_IsoMS/myid_search_res/1481082404607-2016-12-6-8-46-44-116748/1481082404884-510-306.0769236-Neutral.htm) |
| # | RT (s) | mz_light | mz_heavy | mz | distance | int_light | nCharge | nTag | Possible hits |
| 261 | 637.92 | 409.14260 | 411.14805 | 175.08428 | 2.00545 | 14850 | 1 | 1 | [41](http://www.mycompoundid.org/mycompoundid_IsoMS/myid_search_res/1481082404607-2016-12-6-8-46-44-116748/1481082404884-541-175.0842815-Neutral.htm) |
| 262 | 638.36 | 820.31584 | 822.32288 | 586.25752 | 2.00704 | 12800 | 1 | 1 | [8](http://www.mycompoundid.org/mycompoundid_IsoMS/myid_search_res/1481082404607-2016-12-6-8-46-44-116748/1481082404885-648-586.257516-Neutral.htm) |
| 263 | 639.06 | 427.13570 | 429.14172 | 193.07738 | 2.00602 | 23281 | 1 | 1 | [7](http://www.mycompoundid.org/mycompoundid_IsoMS/myid_search_res/1481082404607-2016-12-6-8-46-44-116748/1481082404886-433-193.0773763-Neutral.htm) |
| 264 | 639.25 | 376.13238 | 378.13899 | 142.07406 | 2.00661 | 50213 | 1 | 1 | [13](http://www.mycompoundid.org/mycompoundid_IsoMS/myid_search_res/1481082404607-2016-12-6-8-46-44-116748/1481082404886-380-142.0740583-Neutral.htm) |
| 265 | 643.67 | 355.09417 | 357.09975 | 242.07170 | 2.00558 | 153213 | 2 | 2 | [7](http://www.mycompoundid.org/mycompoundid_IsoMS/myid_search_res/1481082404607-2016-12-6-8-46-44-116748/1481082404888-356-242.0716985-Neutral.htm) |
| 266 | 648.89 | 578.21120 | 580.21766 | 344.15288 | 2.00646 | 24962 | 1 | 1 | [1](http://www.mycompoundid.org/mycompoundid_IsoMS/myid_search_res/1481082404607-2016-12-6-8-46-44-116748/1481082404888-702-344.1528789-Neutral.htm) |
| 267 | 650.67 | 370.09800 | 372.10423 | 136.03968 | 2.00623 | 7773750 | 1 | 1 | [10](http://www.mycompoundid.org/mycompoundid_IsoMS/myid_search_res/1481082404607-2016-12-6-8-46-44-116748/1481082404889-588-136.0396762-Neutral.htm) |
| 268 | 650.67 | 603.14825 | 605.15460 | 369.08993 | 2.00635 | 82197 | 1 | 1 | [7](http://www.mycompoundid.org/mycompoundid_IsoMS/myid_search_res/1481082404607-2016-12-6-8-46-44-116748/1481082404889-360-369.0899319-Neutral.htm) |
| 269 | 651.38 | 428.13809 | 430.14467 | 194.07977 | 2.00658 | 6630 | 1 | 1 | [33](http://www.mycompoundid.org/mycompoundid_IsoMS/myid_search_res/1481082404607-2016-12-6-8-46-44-116748/1481082404890-484-194.079774-Neutral.htm) |
| 270 | 656.17 | 805.32125 | 807.32686 | 571.26293 | 2.00561 | 16900 | 1 | 1 | [4](http://www.mycompoundid.org/mycompoundid_IsoMS/myid_search_res/1481082404607-2016-12-6-8-46-44-116748/1481082404891-466-571.262928-Neutral.htm) |
| 271 | 657.15 | 563.21558 | 565.22122 | 329.15726 | 2.00565 | 14823 | 1 | 1 | [3](http://www.mycompoundid.org/mycompoundid_IsoMS/myid_search_res/1481082404607-2016-12-6-8-46-44-116748/1481082404892-63-329.1572559-Neutral.htm) |
| 272 | 659.53 | 349.12263 | 351.12898 | 115.06431 | 2.00635 | 11411250 | 1 | 1 | [33](http://www.mycompoundid.org/mycompoundid_IsoMS/myid_search_res/1481082404607-2016-12-6-8-46-44-116748/1481082404892-444-115.0643133-Neutral.htm) |
| 273 | 661.17 | 477.16393 | 479.16971 | 243.10561 | 2.00578 | 2880000 | 1 | 1 | [1](http://www.mycompoundid.org/mycompoundid_IsoMS/myid_search_res/1481082404607-2016-12-6-8-46-44-116748/1481082404894-43-243.105612-Neutral.htm) |
| 274 | 661.65 | 629.21084 | 633.22356 | 162.10150 | 4.01272 | 46294 | 1 | 2 | [15](http://www.mycompoundid.org/mycompoundid_IsoMS/myid_search_res/1481082404607-2016-12-6-8-46-44-116748/1481082404894-459-162.101498-Neutral.htm) |
| 275 | 662.03 | 496.13527 | 498.14096 | 524.15389 | 2.00570 | 8080 | 2 | 2 | [1](http://www.mycompoundid.org/mycompoundid_IsoMS/myid_search_res/1481082404607-2016-12-6-8-46-44-116748/1481082404895-414-524.15389-Neutral.htm) |
| 276 | 666.62 | 515.13002 | 519.14308 | 48.02068 | 4.01305 | 33563 | 1 | 2 | [18](http://www.mycompoundid.org/mycompoundid_IsoMS/myid_search_res/1481082404607-2016-12-6-8-46-44-116748/1481082404895-636-48.0206825-Neutral.htm) |
| 277 | 668.38 | 513.11465 | 517.13032 | 46.00531 | 4.01567 | 12400 | 1 | 2 | [20](http://www.mycompoundid.org/mycompoundid_IsoMS/myid_search_res/1481082404607-2016-12-6-8-46-44-116748/1481082404896-236-46.005306-Neutral.htm) |
| 278 | 671.02 | 362.10080 | 364.10711 | 256.08497 | 2.00631 | 7163 | 2 | 2 | [3](http://www.mycompoundid.org/mycompoundid_IsoMS/myid_search_res/1481082404607-2016-12-6-8-46-44-116748/1481082404897-260-256.0849655-Neutral.htm) |
| 279 | 672.40 | 599.22560 | 601.23295 | 365.16728 | 2.00736 | 87600 | 1 | 1 | [19](http://www.mycompoundid.org/mycompoundid_IsoMS/myid_search_res/1481082404607-2016-12-6-8-46-44-116748/1481082404898-92-365.167275-Neutral.htm) |
| 280 | 674.19 | 550.19817 | 552.20432 | 316.13985 | 2.00614 | 55000 | 1 | 1 | [4](http://www.mycompoundid.org/mycompoundid_IsoMS/myid_search_res/1481082404607-2016-12-6-8-46-44-116748/1481082404899-101-316.139854-Neutral.htm) |
| 281 | 676.19 | 649.25401 | 651.25491 | 415.19569 | 2.00090 | 11700 | 1 | 1 | [5](http://www.mycompoundid.org/mycompoundid_IsoMS/myid_search_res/1481082404607-2016-12-6-8-46-44-116748/1481082404900-681-415.195687-Neutral.htm) |
| 282 | 677.13 | 570.21298 | 572.22247 | 336.15466 | 2.00949 | 11800 | 1 | 1 | [8](http://www.mycompoundid.org/mycompoundid_IsoMS/myid_search_res/1481082404607-2016-12-6-8-46-44-116748/1481082404900-114-336.154655-Neutral.htm) |
| 283 | 678.30 | 515.13076 | 519.14331 | 48.02142 | 4.01255 | 36200 | 1 | 2 | [18](http://www.mycompoundid.org/mycompoundid_IsoMS/myid_search_res/1481082404607-2016-12-6-8-46-44-116748/1481082404901-254-48.021423-Neutral.htm) |
| 284 | 678.75 | 678.25991 | 680.26688 | 444.20159 | 2.00697 | 43325 | 1 | 1 | [1](http://www.mycompoundid.org/mycompoundid_IsoMS/myid_search_res/1481082404607-2016-12-6-8-46-44-116748/1481082404902-240-444.201591-Neutral.htm) |
| # | RT (s) | mz_light | mz_heavy | mz | distance | int_light | nCharge | nTag | Possible hits |
| 285 | 680.35 | 420.15876 | 422.16564 | 186.10044 | 2.00688 | 63300 | 1 | 1 | [15](http://www.mycompoundid.org/mycompoundid_IsoMS/myid_search_res/1481082404607-2016-12-6-8-46-44-116748/1481082404902-477-186.1004393-Neutral.htm) |
| 286 | 680.64 | 380.16324 | 382.16945 | 146.10492 | 2.00621 | 23225 | 1 | 1 | [23](http://www.mycompoundid.org/mycompoundid_IsoMS/myid_search_res/1481082404607-2016-12-6-8-46-44-116748/1481082404903-807-146.1049215-Neutral.htm) |
| 287 | 681.13 | 447.10422 | 449.10983 | 426.09179 | 2.00562 | 97300 | 2 | 2 | [9](http://www.mycompoundid.org/mycompoundid_IsoMS/myid_search_res/1481082404607-2016-12-6-8-46-44-116748/1481082404904-99-426.09179-Neutral.htm) |
| 288 | 681.48 | 408.15890 | 410.16512 | 174.10058 | 2.00622 | 147438 | 1 | 1 | [35](http://www.mycompoundid.org/mycompoundid_IsoMS/myid_search_res/1481082404607-2016-12-6-8-46-44-116748/1481082404904-269-174.1005795-Neutral.htm) |
| 289 | 682.61 | 695.34016 | 697.34159 | 461.28184 | 2.00143 | 7380 | 1 | 1 | [10](http://www.mycompoundid.org/mycompoundid_IsoMS/myid_search_res/1481082404607-2016-12-6-8-46-44-116748/1481082404905-558-461.281842-Neutral.htm) |
| 290 | 682.80 | 622.22753 | 624.23503 | 388.16921 | 2.00751 | 24450 | 1 | 1 | [1](http://www.mycompoundid.org/mycompoundid_IsoMS/myid_search_res/1481082404607-2016-12-6-8-46-44-116748/1481082404906-269-388.1692075-Neutral.htm) |
| 291 | 685.20 | 335.14252 | 337.14806 | 101.08420 | 2.00554 | 79800 | 1 | 1 | [20](http://www.mycompoundid.org/mycompoundid_IsoMS/myid_search_res/1481082404607-2016-12-6-8-46-44-116748/1481082404907-787-101.084199-Neutral.htm) |
| 292 | 685.64 | 376.13304 | 378.13938 | 142.07472 | 2.00634 | 181500 | 1 | 1 | [13](http://www.mycompoundid.org/mycompoundid_IsoMS/myid_search_res/1481082404607-2016-12-6-8-46-44-116748/1481082404908-350-142.074715-Neutral.htm) |
| 293 | 687.98 | 805.32424 | 807.33089 | 571.26592 | 2.00665 | 115225 | 1 | 1 | [3](http://www.mycompoundid.org/mycompoundid_IsoMS/myid_search_res/1481082404607-2016-12-6-8-46-44-116748/1481082404909-687-571.26592-Neutral.htm) |
| 294 | 688.41 | 486.16893 | 488.17453 | 252.11061 | 2.00560 | 13500 | 1 | 1 | [9](http://www.mycompoundid.org/mycompoundid_IsoMS/myid_search_res/1481082404607-2016-12-6-8-46-44-116748/1481082404909-403-252.110611-Neutral.htm) |
| 295 | 691.52 | 351.13804 | 353.14453 | 117.07972 | 2.00649 | 4883750 | 1 | 1 | [38](http://www.mycompoundid.org/mycompoundid_IsoMS/myid_search_res/1481082404607-2016-12-6-8-46-44-116748/1481082404910-565-117.07972-Neutral.htm) |
| 296 | 693.11 | 578.21074 | 580.21793 | 344.15242 | 2.00719 | 15550 | 1 | 1 | [1](http://www.mycompoundid.org/mycompoundid_IsoMS/myid_search_res/1481082404607-2016-12-6-8-46-44-116748/1481082404911-894-344.152424-Neutral.htm) |
| 297 | 694.42 | 792.31510 | 794.32160 | 558.25678 | 2.00650 | 38200 | 1 | 1 | [12](http://www.mycompoundid.org/mycompoundid_IsoMS/myid_search_res/1481082404607-2016-12-6-8-46-44-116748/1481082404912-527-558.256784-Neutral.htm) |
| 298 | 696.70 | 337.12183 | 339.12738 | 103.06351 | 2.00555 | 71066 | 1 | 1 | [45](http://www.mycompoundid.org/mycompoundid_IsoMS/myid_search_res/1481082404607-2016-12-6-8-46-44-116748/1481082404912-584-103.0635144-Neutral.htm) |
| 299 | 697.36 | 706.27382 | 708.27929 | 472.21550 | 2.00547 | 31391 | 1 | 1 | [2](http://www.mycompoundid.org/mycompoundid_IsoMS/myid_search_res/1481082404607-2016-12-6-8-46-44-116748/1481082404914-293-472.2154977-Neutral.htm) |
| 300 | 698.11 | 605.15542 | 607.16323 | 371.09710 | 2.00781 | 7215 | 1 | 1 | [9](http://www.mycompoundid.org/mycompoundid_IsoMS/myid_search_res/1481082404607-2016-12-6-8-46-44-116748/1481082404914-829-371.0970965-Neutral.htm) |
| 301 | 698.99 | 601.14498 | 603.15104 | 367.08666 | 2.00605 | 16688 | 1 | 1 | [8](http://www.mycompoundid.org/mycompoundid_IsoMS/myid_search_res/1481082404607-2016-12-6-8-46-44-116748/1481082404915-837-367.0866648-Neutral.htm) |
| 302 | 701.87 | 654.25767 | 656.26397 | 420.19935 | 2.00630 | 22788 | 1 | 1 | [2](http://www.mycompoundid.org/mycompoundid_IsoMS/myid_search_res/1481082404607-2016-12-6-8-46-44-116748/1481082404916-692-420.1993481-Neutral.htm) |
| 303 | 701.98 | 383.11071 | 385.11669 | 149.05239 | 2.00598 | 3057500 | 1 | 1 | [9](http://www.mycompoundid.org/mycompoundid_IsoMS/myid_search_res/1481082404607-2016-12-6-8-46-44-116748/1481082404917-546-149.0523903-Neutral.htm) |
| 304 | 703.45 | 578.22115 | 580.22578 | 344.16283 | 2.00463 | 11800 | 1 | 1 | [9](http://www.mycompoundid.org/mycompoundid_IsoMS/myid_search_res/1481082404607-2016-12-6-8-46-44-116748/1481082404918-467-344.162829-Neutral.htm) |
| 305 | 703.73 | 346.08617 | 348.09312 | 112.02785 | 2.00696 | 589969 | 1 | 1 | [10](http://www.mycompoundid.org/mycompoundid_IsoMS/myid_search_res/1481082404607-2016-12-6-8-46-44-116748/1481082404918-708-112.0278495-Neutral.htm) |
| 306 | 704.92 | 691.26804 | 693.27476 | 457.20972 | 2.00672 | 8290 | 1 | 1 | [11](http://www.mycompoundid.org/mycompoundid_IsoMS/myid_search_res/1481082404607-2016-12-6-8-46-44-116748/1481082404919-809-457.2097245-Neutral.htm) |
| 307 | 705.57 | 351.13814 | 353.14451 | 117.07982 | 2.00637 | 5506875 | 1 | 1 | [38](http://www.mycompoundid.org/mycompoundid_IsoMS/myid_search_res/1481082404607-2016-12-6-8-46-44-116748/1481082404919-112-117.0798171-Neutral.htm) |
| 308 | 709.16 | 657.24312 | 659.25336 | 423.18480 | 2.01024 | 9410 | 1 | 1 | [6](http://www.mycompoundid.org/mycompoundid_IsoMS/myid_search_res/1481082404607-2016-12-6-8-46-44-116748/1481082404921-614-423.184804-Neutral.htm) |
| # | RT (s) | mz_light | mz_heavy | mz | distance | int_light | nCharge | nTag | Possible hits |
| 309 | 711.08 | 447.10361 | 449.11050 | 213.04529 | 2.00689 | 39900 | 1 | 1 | [4](http://www.mycompoundid.org/mycompoundid_IsoMS/myid_search_res/1481082404607-2016-12-6-8-46-44-116748/1481082404922-284-213.045292-Neutral.htm) |
| 310 | 713.17 | 599.22892 | 601.23610 | 365.17060 | 2.00718 | 19250 | 1 | 1 | [19](http://www.mycompoundid.org/mycompoundid_IsoMS/myid_search_res/1481082404607-2016-12-6-8-46-44-116748/1481082404923-377-365.170596-Neutral.htm) |
| 311 | 713.83 | 321.09108 | 323.09733 | 87.03276 | 2.00625 | 99513 | 1 | 1 | [18](http://www.mycompoundid.org/mycompoundid_IsoMS/myid_search_res/1481082404607-2016-12-6-8-46-44-116748/1481082404924-228-87.03276263-Neutral.htm) |
| 312 | 721.17 | 614.23327 | 616.23907 | 380.17495 | 2.00580 | 9450 | 1 | 1 | [9](http://www.mycompoundid.org/mycompoundid_IsoMS/myid_search_res/1481082404607-2016-12-6-8-46-44-116748/1481082404926-261-380.174948-Neutral.htm) |
| 313 | 722.45 | 660.14851 | 662.15682 | 426.09019 | 2.00831 | 6610 | 1 | 1 | [8](http://www.mycompoundid.org/mycompoundid_IsoMS/myid_search_res/1481082404607-2016-12-6-8-46-44-116748/1481082404927-600-426.090185-Neutral.htm) |
| 314 | 724.12 | 335.14244 | 337.14875 | 101.08412 | 2.00632 | 49275 | 1 | 1 | [20](http://www.mycompoundid.org/mycompoundid_IsoMS/myid_search_res/1481082404607-2016-12-6-8-46-44-116748/1481082404928-466-101.084115-Neutral.htm) |
| 315 | 726.66 | 367.07878 | 369.08469 | 133.02046 | 2.00591 | 185845 | 1 | 1 | [4](http://www.mycompoundid.org/mycompoundid_IsoMS/myid_search_res/1481082404607-2016-12-6-8-46-44-116748/1481082404930-717-133.0204624-Neutral.htm) |
| 316 | 727.18 | 447.10405 | 449.11097 | 426.09146 | 2.00692 | 877000 | 2 | 2 | [9](http://www.mycompoundid.org/mycompoundid_IsoMS/myid_search_res/1481082404607-2016-12-6-8-46-44-116748/1481082404930-377-426.09146-Neutral.htm) |
| 317 | 727.46 | 783.17843 | 785.18340 | 549.12011 | 2.00498 | 6100 | 1 | 1 | [2](http://www.mycompoundid.org/mycompoundid_IsoMS/myid_search_res/1481082404607-2016-12-6-8-46-44-116748/1481082404930-321-549.120105-Neutral.htm) |
| 318 | 727.51 | 578.21502 | 580.21962 | 344.15670 | 2.00460 | 13280 | 1 | 1 | [2](http://www.mycompoundid.org/mycompoundid_IsoMS/myid_search_res/1481082404607-2016-12-6-8-46-44-116748/1481082404931-692-344.1567013-Neutral.htm) |
| 319 | 728.14 | 377.10763 | 379.11261 | 286.09863 | 2.00498 | 6090 | 2 | 2 | [12](http://www.mycompoundid.org/mycompoundid_IsoMS/myid_search_res/1481082404607-2016-12-6-8-46-44-116748/1481082404931-86-286.098627-Neutral.htm) |
| 320 | 730.10 | 457.16462 | 459.17205 | 223.10630 | 2.00743 | 30800 | 1 | 1 | [20](http://www.mycompoundid.org/mycompoundid_IsoMS/myid_search_res/1481082404607-2016-12-6-8-46-44-116748/1481082404932-366-223.106302-Neutral.htm) |
| 321 | 730.32 | 641.25014 | 643.25686 | 407.19182 | 2.00671 | 46400 | 1 | 1 | [4](http://www.mycompoundid.org/mycompoundid_IsoMS/myid_search_res/1481082404607-2016-12-6-8-46-44-116748/1481082404932-682-407.191823-Neutral.htm) |
| 322 | 734.50 | 409.14398 | 411.15035 | 175.08566 | 2.00638 | 834750 | 1 | 1 | [41](http://www.mycompoundid.org/mycompoundid_IsoMS/myid_search_res/1481082404607-2016-12-6-8-46-44-116748/1481082404933-872-175.0856573-Neutral.htm) |
| 323 | 734.70 | 527.19868 | 529.20534 | 293.14036 | 2.00666 | 91413 | 1 | 1 | [10](http://www.mycompoundid.org/mycompoundid_IsoMS/myid_search_res/1481082404607-2016-12-6-8-46-44-116748/1481082404945-18-293.140359-Neutral.htm) |
| 324 | 736.07 | 508.18198 | 510.18851 | 274.12366 | 2.00652 | 139066 | 1 | 1 | [1](http://www.mycompoundid.org/mycompoundid_IsoMS/myid_search_res/1481082404607-2016-12-6-8-46-44-116748/1481082404946-98-274.1236649-Neutral.htm) |
| 325 | 736.41 | 335.14219 | 337.14889 | 101.08387 | 2.00669 | 50306 | 1 | 1 | [20](http://www.mycompoundid.org/mycompoundid_IsoMS/myid_search_res/1481082404607-2016-12-6-8-46-44-116748/1481082404946-7-101.0838739-Neutral.htm) |
| 326 | 736.76 | 585.21978 | 587.22641 | 351.16146 | 2.00663 | 507248 | 1 | 1 | [3](http://www.mycompoundid.org/mycompoundid_IsoMS/myid_search_res/1481082404607-2016-12-6-8-46-44-116748/1481082404947-514-351.1614636-Neutral.htm) |
| 327 | 737.72 | 422.17337 | 424.18035 | 188.11505 | 2.00698 | 27056 | 1 | 1 | [23](http://www.mycompoundid.org/mycompoundid_IsoMS/myid_search_res/1481082404607-2016-12-6-8-46-44-116748/1481082404947-233-188.1150483-Neutral.htm) |
| 328 | 738.87 | 641.25814 | 643.26461 | 407.19982 | 2.00647 | 11100 | 1 | 1 | [1](http://www.mycompoundid.org/mycompoundid_IsoMS/myid_search_res/1481082404607-2016-12-6-8-46-44-116748/1481082404948-134-407.1998235-Neutral.htm) |
| 329 | 739.11 | 469.15806 | 471.16534 | 470.19948 | 2.00728 | 9060 | 2 | 2 | [5](http://www.mycompoundid.org/mycompoundid_IsoMS/myid_search_res/1481082404607-2016-12-6-8-46-44-116748/1481082404948-132-470.19948-Neutral.htm) |
| 330 | 739.75 | 367.07858 | 369.08466 | 133.02026 | 2.00608 | 194884 | 1 | 1 | [4](http://www.mycompoundid.org/mycompoundid_IsoMS/myid_search_res/1481082404607-2016-12-6-8-46-44-116748/1481082404949-585-133.020259-Neutral.htm) |
| 331 | 740.65 | 479.17446 | 481.18111 | 245.11614 | 2.00665 | 9580 | 1 | 1 | [7](http://www.mycompoundid.org/mycompoundid_IsoMS/myid_search_res/1481082404607-2016-12-6-8-46-44-116748/1481082404949-559-245.116136-Neutral.htm) |
| 332 | 740.86 | 395.12823 | 397.13450 | 161.06991 | 2.00627 | 423375 | 1 | 1 | [48](http://www.mycompoundid.org/mycompoundid_IsoMS/myid_search_res/1481082404607-2016-12-6-8-46-44-116748/1481082404950-815-161.0699113-Neutral.htm) |
| # | RT (s) | mz_light | mz_heavy | mz | distance | int_light | nCharge | nTag | Possible hits |
| 333 | 741.29 | 447.10423 | 449.11084 | 426.09182 | 2.00661 | 337000 | 2 | 2 | [9](http://www.mycompoundid.org/mycompoundid_IsoMS/myid_search_res/1481082404607-2016-12-6-8-46-44-116748/1481082404952-890-426.0918238-Neutral.htm) |
| 334 | 742.78 | 676.27248 | 678.27837 | 442.21416 | 2.00590 | 96675 | 1 | 1 | [3](http://www.mycompoundid.org/mycompoundid_IsoMS/myid_search_res/1481082404607-2016-12-6-8-46-44-116748/1481082404953-648-442.2141568-Neutral.htm) |
| 335 | 742.80 | 448.10391 | 450.11023 | 428.09119 | 2.00631 | 76550 | 2 | 2 | [2](http://www.mycompoundid.org/mycompoundid_IsoMS/myid_search_res/1481082404607-2016-12-6-8-46-44-116748/1481082404953-927-428.091186-Neutral.htm) |
| 336 | 747.39 | 438.14918 | 440.15526 | 204.09086 | 2.00608 | 369203 | 1 | 1 | [11](http://www.mycompoundid.org/mycompoundid_IsoMS/myid_search_res/1481082404607-2016-12-6-8-46-44-116748/1481082404954-135-204.0908567-Neutral.htm) |
| 337 | 747.69 | 415.11075 | 417.11678 | 362.10486 | 2.00603 | 9695 | 2 | 2 | [3](http://www.mycompoundid.org/mycompoundid_IsoMS/myid_search_res/1481082404607-2016-12-6-8-46-44-116748/1481082404955-673-362.10486-Neutral.htm) |
| 338 | 747.78 | 396.13705 | 398.14364 | 324.15746 | 2.00659 | 3176922 | 2 | 2 | [9](http://www.mycompoundid.org/mycompoundid_IsoMS/myid_search_res/1481082404607-2016-12-6-8-46-44-116748/1481082404955-31-324.1574602-Neutral.htm) |
| 339 | 748.20 | 397.13817 | 399.14448 | 326.15970 | 2.00630 | 713000 | 2 | 2 | [2](http://www.mycompoundid.org/mycompoundid_IsoMS/myid_search_res/1481082404607-2016-12-6-8-46-44-116748/1481082404956-240-326.159704-Neutral.htm) |
| 340 | 748.48 | 629.21024 | 633.22259 | 162.10090 | 4.01235 | 28744 | 1 | 2 | [15](http://www.mycompoundid.org/mycompoundid_IsoMS/myid_search_res/1481082404607-2016-12-6-8-46-44-116748/1481082404956-225-162.1009012-Neutral.htm) |
| 341 | 750.37 | 614.23032 | 616.23736 | 380.17200 | 2.00704 | 29297 | 1 | 1 | [12](http://www.mycompoundid.org/mycompoundid_IsoMS/myid_search_res/1481082404607-2016-12-6-8-46-44-116748/1481082404957-971-380.1720024-Neutral.htm) |
| 342 | 750.43 | 376.13226 | 378.13863 | 142.07394 | 2.00637 | 26238 | 1 | 1 | [13](http://www.mycompoundid.org/mycompoundid_IsoMS/myid_search_res/1481082404607-2016-12-6-8-46-44-116748/1481082404958-775-142.0739397-Neutral.htm) |
| 343 | 754.58 | 508.18119 | 510.18854 | 274.12287 | 2.00735 | 16000 | 1 | 1 | [2](http://www.mycompoundid.org/mycompoundid_IsoMS/myid_search_res/1481082404607-2016-12-6-8-46-44-116748/1481082404961-627-274.122869-Neutral.htm) |
| 344 | 755.02 | 812.33035 | 814.33889 | 578.27203 | 2.00854 | 8675 | 1 | 1 | [1](http://www.mycompoundid.org/mycompoundid_IsoMS/myid_search_res/1481082404607-2016-12-6-8-46-44-116748/1481082404961-596-578.272025-Neutral.htm) |
| 345 | 755.28 | 400.08638 | 402.09188 | 166.02806 | 2.00550 | 44934 | 1 | 1 | [18](http://www.mycompoundid.org/mycompoundid_IsoMS/myid_search_res/1481082404607-2016-12-6-8-46-44-116748/1481082404961-745-166.0280608-Neutral.htm) |
| 346 | 756.50 | 362.10149 | 364.10718 | 256.08635 | 2.00569 | 7800 | 2 | 2 | [11](http://www.mycompoundid.org/mycompoundid_IsoMS/myid_search_res/1481082404607-2016-12-6-8-46-44-116748/1481082404962-12-256.086345-Neutral.htm) |
| 347 | 757.00 | 547.14276 | 549.14991 | 313.08444 | 2.00715 | 11715 | 1 | 1 | [11](http://www.mycompoundid.org/mycompoundid_IsoMS/myid_search_res/1481082404607-2016-12-6-8-46-44-116748/1481082404962-952-313.0844441-Neutral.htm) |
| 348 | 762.09 | 335.14241 | 337.14891 | 101.08409 | 2.00651 | 70341 | 1 | 1 | [20](http://www.mycompoundid.org/mycompoundid_IsoMS/myid_search_res/1481082404607-2016-12-6-8-46-44-116748/1481082404964-467-101.0840858-Neutral.htm) |
| 349 | 762.22 | 624.25196 | 626.25838 | 390.19364 | 2.00643 | 12300 | 1 | 1 | [4](http://www.mycompoundid.org/mycompoundid_IsoMS/myid_search_res/1481082404607-2016-12-6-8-46-44-116748/1481082404964-279-390.193635-Neutral.htm) |
| 350 | 763.81 | 456.15706 | 458.16104 | 222.09874 | 2.00399 | 12300 | 1 | 1 | [23](http://www.mycompoundid.org/mycompoundid_IsoMS/myid_search_res/1481082404607-2016-12-6-8-46-44-116748/1481082404965-719-222.098736-Neutral.htm) |
| 351 | 764.25 | 464.17393 | 466.17955 | 230.11561 | 2.00562 | 12400 | 1 | 1 | [9](http://www.mycompoundid.org/mycompoundid_IsoMS/myid_search_res/1481082404607-2016-12-6-8-46-44-116748/1481082404966-839-230.115606-Neutral.htm) |
| 352 | 765.75 | 381.14841 | 383.15460 | 147.09009 | 2.00618 | 207281 | 1 | 1 | [39](http://www.mycompoundid.org/mycompoundid_IsoMS/myid_search_res/1481082404607-2016-12-6-8-46-44-116748/1481082404967-222-147.0900912-Neutral.htm) |
| 353 | 766.89 | 319.11190 | 321.11744 | 85.05358 | 2.00553 | 112100 | 1 | 1 | [16](http://www.mycompoundid.org/mycompoundid_IsoMS/myid_search_res/1481082404607-2016-12-6-8-46-44-116748/1481082404968-645-85.05358325-Neutral.htm) |
| 354 | 768.19 | 477.18137 | 479.18770 | 243.12305 | 2.00634 | 54691 | 1 | 1 | [8](http://www.mycompoundid.org/mycompoundid_IsoMS/myid_search_res/1481082404607-2016-12-6-8-46-44-116748/1481082404969-257-243.1230452-Neutral.htm) |
| 355 | 768.40 | 526.16437 | 528.17133 | 292.10605 | 2.00695 | 20563 | 1 | 1 | [14](http://www.mycompoundid.org/mycompoundid_IsoMS/myid_search_res/1481082404607-2016-12-6-8-46-44-116748/1481082404970-714-292.1060539-Neutral.htm) |
| 356 | 772.95 | 452.18433 | 454.19186 | 218.12601 | 2.00752 | 12081 | 1 | 1 | [6](http://www.mycompoundid.org/mycompoundid_IsoMS/myid_search_res/1481082404607-2016-12-6-8-46-44-116748/1481082404971-884-218.1260148-Neutral.htm) |
| # | RT (s) | mz_light | mz_heavy | mz | distance | int_light | nCharge | nTag | Possible hits |
| 357 | 776.08 | 527.19909 | 529.20616 | 293.14077 | 2.00707 | 13200 | 1 | 1 | [5](http://www.mycompoundid.org/mycompoundid_IsoMS/myid_search_res/1481082404607-2016-12-6-8-46-44-116748/1481082404972-518-293.1407705-Neutral.htm) |
| 358 | 776.15 | 454.19720 | 456.20543 | 220.13888 | 2.00823 | 10549 | 1 | 1 | [1](http://www.mycompoundid.org/mycompoundid_IsoMS/myid_search_res/1481082404607-2016-12-6-8-46-44-116748/1481082404972-539-220.1388776-Neutral.htm) |
| 359 | 778.61 | 409.14393 | 411.15026 | 175.08561 | 2.00633 | 5070000 | 1 | 1 | [41](http://www.mycompoundid.org/mycompoundid_IsoMS/myid_search_res/1481082404607-2016-12-6-8-46-44-116748/1481082404973-823-175.085611-Neutral.htm) |
| 360 | 781.51 | 860.38274 | 862.39057 | 626.32442 | 2.00783 | 6760 | 1 | 1 | [7](http://www.mycompoundid.org/mycompoundid_IsoMS/myid_search_res/1481082404607-2016-12-6-8-46-44-116748/1481082404974-580-626.32442-Neutral.htm) |
| 361 | 781.90 | 335.14242 | 337.14930 | 101.08410 | 2.00688 | 99600 | 1 | 1 | [20](http://www.mycompoundid.org/mycompoundid_IsoMS/myid_search_res/1481082404607-2016-12-6-8-46-44-116748/1481082404975-136-101.0841043-Neutral.htm) |
| 362 | 782.16 | 477.18115 | 479.18681 | 243.12283 | 2.00566 | 11300 | 1 | 1 | [8](http://www.mycompoundid.org/mycompoundid_IsoMS/myid_search_res/1481082404607-2016-12-6-8-46-44-116748/1481082404976-775-243.122825-Neutral.htm) |
| 363 | 784.30 | 469.18384 | 471.18967 | 470.25104 | 2.00582 | 5370 | 2 | 2 | [8](http://www.mycompoundid.org/mycompoundid_IsoMS/myid_search_res/1481082404607-2016-12-6-8-46-44-116748/1481082404976-907-470.251044-Neutral.htm) |
| 364 | 784.41 | 321.11600 | 323.12256 | 174.11536 | 2.00656 | 726929 | 2 | 2 | [5](http://www.mycompoundid.org/mycompoundid_IsoMS/myid_search_res/1481082404607-2016-12-6-8-46-44-116748/1481082404977-345-174.115361-Neutral.htm) |
| 365 | 784.70 | 426.12069 | 428.12700 | 192.06237 | 2.00631 | 30900 | 1 | 1 | [36](http://www.mycompoundid.org/mycompoundid_IsoMS/myid_search_res/1481082404607-2016-12-6-8-46-44-116748/1481082404977-233-192.0623665-Neutral.htm) |
| 366 | 785.16 | 393.13701 | 395.14349 | 318.15739 | 2.00648 | 287482 | 2 | 2 | [3](http://www.mycompoundid.org/mycompoundid_IsoMS/myid_search_res/1481082404607-2016-12-6-8-46-44-116748/1481082404979-219-318.1573871-Neutral.htm) |
| 367 | 788.47 | 452.18432 | 454.19119 | 218.12600 | 2.00687 | 31444 | 1 | 1 | [6](http://www.mycompoundid.org/mycompoundid_IsoMS/myid_search_res/1481082404607-2016-12-6-8-46-44-116748/1481082404979-167-218.1259963-Neutral.htm) |
| 368 | 790.21 | 458.07414 | 460.07965 | 224.01582 | 2.00552 | 8630 | 1 | 1 | [3](http://www.mycompoundid.org/mycompoundid_IsoMS/myid_search_res/1481082404607-2016-12-6-8-46-44-116748/1481082404980-533-224.015815-Neutral.htm) |
| 369 | 792.25 | 680.06674 | 682.07652 | 446.00842 | 2.00978 | 23000 | 1 | 1 | [3](http://www.mycompoundid.org/mycompoundid_IsoMS/myid_search_res/1481082404607-2016-12-6-8-46-44-116748/1481082404980-76-446.00842-Neutral.htm) |
| 370 | 792.72 | 448.19043 | 450.19675 | 214.13211 | 2.00632 | 14950 | 1 | 1 | [7](http://www.mycompoundid.org/mycompoundid_IsoMS/myid_search_res/1481082404607-2016-12-6-8-46-44-116748/1481082404981-909-214.132114-Neutral.htm) |
| 371 | 793.25 | 351.10226 | 353.11063 | 117.04394 | 2.00837 | 17700 | 1 | 1 | [1](http://www.mycompoundid.org/mycompoundid_IsoMS/myid_search_res/1481082404607-2016-12-6-8-46-44-116748/1481082404981-680-117.043939-Neutral.htm) |
| 372 | 795.39 | 392.12912 | 394.13686 | 316.14159 | 2.00774 | 39000 | 2 | 2 | [3](http://www.mycompoundid.org/mycompoundid_IsoMS/myid_search_res/1481082404607-2016-12-6-8-46-44-116748/1481082404982-323-316.141593-Neutral.htm) |
| 373 | 796.29 | 592.22619 | 594.23353 | 358.16787 | 2.00734 | 17900 | 1 | 1 | [1](http://www.mycompoundid.org/mycompoundid_IsoMS/myid_search_res/1481082404607-2016-12-6-8-46-44-116748/1481082404982-246-358.1678737-Neutral.htm) |
| 374 | 797.27 | 383.12959 | 385.13434 | 149.07127 | 2.00475 | 26300 | 1 | 1 | [7](http://www.mycompoundid.org/mycompoundid_IsoMS/myid_search_res/1481082404607-2016-12-6-8-46-44-116748/1481082404983-387-149.0712693-Neutral.htm) |
| 375 | 798.45 | 458.17665 | 460.18294 | 224.11833 | 2.00630 | 23025 | 1 | 1 | [10](http://www.mycompoundid.org/mycompoundid_IsoMS/myid_search_res/1481082404607-2016-12-6-8-46-44-116748/1481082404983-281-224.118328-Neutral.htm) |
| 376 | 799.33 | 335.14272 | 337.14927 | 101.08440 | 2.00655 | 11100 | 1 | 1 | [20](http://www.mycompoundid.org/mycompoundid_IsoMS/myid_search_res/1481082404607-2016-12-6-8-46-44-116748/1481082404984-429-101.084396-Neutral.htm) |
| 377 | 802.46 | 360.10231 | 362.11009 | 126.04399 | 2.00778 | 1398938 | 1 | 1 | [14](http://www.mycompoundid.org/mycompoundid_IsoMS/myid_search_res/1481082404607-2016-12-6-8-46-44-116748/1481082404985-404-126.0439872-Neutral.htm) |
| 378 | 802.61 | 311.08412 | 313.09081 | 154.05160 | 2.00669 | 625969 | 2 | 2 | [2](http://www.mycompoundid.org/mycompoundid_IsoMS/myid_search_res/1481082404607-2016-12-6-8-46-44-116748/1481082404985-529-154.0515991-Neutral.htm) |
| 379 | 805.99 | 371.63317 | 373.64010 | 275.14970 | 2.00693 | 24234 | 2 | 2 | [13](http://www.mycompoundid.org/mycompoundid_IsoMS/myid_search_res/1481082404607-2016-12-6-8-46-44-116748/1481082404986-4-275.1497017-Neutral.htm) |
| 380 | 807.82 | 399.13874 | 401.14507 | 165.08042 | 2.00633 | 6773984 | 1 | 1 | [48](http://www.mycompoundid.org/mycompoundid_IsoMS/myid_search_res/1481082404607-2016-12-6-8-46-44-116748/1481082404987-354-165.0804194-Neutral.htm) |
| # | RT (s) | mz_light | mz_heavy | mz | distance | int_light | nCharge | nTag | Possible hits |
| 381 | 810.68 | 592.22670 | 594.23280 | 358.16838 | 2.00611 | 18078 | 1 | 1 | [2](http://www.mycompoundid.org/mycompoundid_IsoMS/myid_search_res/1481082404607-2016-12-6-8-46-44-116748/1481082404988-901-358.1683786-Neutral.htm) |
| 382 | 819.61 | 453.13465 | 455.13834 | 219.07633 | 2.00369 | 13650 | 1 | 1 | [41](http://www.mycompoundid.org/mycompoundid_IsoMS/myid_search_res/1481082404607-2016-12-6-8-46-44-116748/1481082404989-675-219.076331-Neutral.htm) |
| 383 | 821.19 | 365.15396 | 367.16031 | 131.09564 | 2.00635 | 7231250 | 1 | 1 | [36](http://www.mycompoundid.org/mycompoundid_IsoMS/myid_search_res/1481082404607-2016-12-6-8-46-44-116748/1481082404991-233-131.0956365-Neutral.htm) |
| 384 | 827.89 | 815.30511 | 817.31591 | 581.24679 | 2.01080 | 14550 | 1 | 1 | [19](http://www.mycompoundid.org/mycompoundid_IsoMS/myid_search_res/1481082404607-2016-12-6-8-46-44-116748/1481082404992-798-581.246793-Neutral.htm) |
| 385 | 828.22 | 731.28506 | 733.29231 | 497.22674 | 2.00726 | 14700 | 1 | 1 | [1](http://www.mycompoundid.org/mycompoundid_IsoMS/myid_search_res/1481082404607-2016-12-6-8-46-44-116748/1481082404993-893-497.226737-Neutral.htm) |
| 386 | 828.28 | 727.25793 | 731.27375 | 260.14859 | 4.01582 | 12700 | 1 | 2 | [1](http://www.mycompoundid.org/mycompoundid_IsoMS/myid_search_res/1481082404607-2016-12-6-8-46-44-116748/1481082404993-471-260.148588-Neutral.htm) |
| 387 | 829.56 | 729.27325 | 733.28755 | 262.16391 | 4.01430 | 16200 | 1 | 2 | [2](http://www.mycompoundid.org/mycompoundid_IsoMS/myid_search_res/1481082404607-2016-12-6-8-46-44-116748/1481082404994-498-262.163907-Neutral.htm) |
| 388 | 829.88 | 377.11666 | 379.12301 | 143.05834 | 2.00635 | 101000 | 1 | 1 | [25](http://www.mycompoundid.org/mycompoundid_IsoMS/myid_search_res/1481082404607-2016-12-6-8-46-44-116748/1481082404994-433-143.058342-Neutral.htm) |
| 389 | 837.18 | 365.15381 | 367.16020 | 131.09549 | 2.00640 | 11103628 | 1 | 1 | [36](http://www.mycompoundid.org/mycompoundid_IsoMS/myid_search_res/1481082404607-2016-12-6-8-46-44-116748/1481082404995-936-131.0954869-Neutral.htm) |
| 390 | 843.36 | 392.14721 | 394.15303 | 316.17778 | 2.00582 | 12500 | 2 | 2 | [3](http://www.mycompoundid.org/mycompoundid_IsoMS/myid_search_res/1481082404607-2016-12-6-8-46-44-116748/1481082404997-587-316.177784-Neutral.htm) |
| 391 | 843.55 | 423.15840 | 425.16419 | 189.10008 | 2.00580 | 15175 | 1 | 1 | [28](http://www.mycompoundid.org/mycompoundid_IsoMS/myid_search_res/1481082404607-2016-12-6-8-46-44-116748/1481082404997-687-189.100076-Neutral.htm) |
| 392 | 846.22 | 381.09418 | 383.10011 | 147.03586 | 2.00593 | 173772 | 1 | 1 | [4](http://www.mycompoundid.org/mycompoundid_IsoMS/myid_search_res/1481082404607-2016-12-6-8-46-44-116748/1481082404998-284-147.035863-Neutral.htm) |
| 393 | 849.58 | 681.31077 | 683.31661 | 447.25245 | 2.00583 | 13700 | 1 | 1 | [7](http://www.mycompoundid.org/mycompoundid_IsoMS/myid_search_res/1481082404607-2016-12-6-8-46-44-116748/1481082404999-966-447.252454-Neutral.htm) |
| 394 | 861.13 | 307.11155 | 309.11857 | 73.05323 | 2.00702 | 486000 | 1 | 1 | [18](http://www.mycompoundid.org/mycompoundid_IsoMS/myid_search_res/1481082404607-2016-12-6-8-46-44-116748/1481082405001-100-73.0532285-Neutral.htm) |
| 395 | 862.24 | 377.10525 | 379.11226 | 286.09386 | 2.00701 | 34981 | 2 | 2 | [9](http://www.mycompoundid.org/mycompoundid_IsoMS/myid_search_res/1481082404607-2016-12-6-8-46-44-116748/1481082405001-500-286.0938568-Neutral.htm) |
| 396 | 862.62 | 397.12573 | 399.13122 | 163.06741 | 2.00550 | 15900 | 1 | 1 | [5](http://www.mycompoundid.org/mycompoundid_IsoMS/myid_search_res/1481082404607-2016-12-6-8-46-44-116748/1481082405002-841-163.067405-Neutral.htm) |
| 397 | 862.65 | 403.14363 | 405.15039 | 338.17063 | 2.00676 | 45588 | 2 | 2 | [7](http://www.mycompoundid.org/mycompoundid_IsoMS/myid_search_res/1481082404607-2016-12-6-8-46-44-116748/1481082405002-245-338.1706258-Neutral.htm) |
| 398 | 862.94 | 612.26765 | 616.28052 | 145.15831 | 4.01287 | 55600 | 1 | 2 | [3](http://www.mycompoundid.org/mycompoundid_IsoMS/myid_search_res/1481082404607-2016-12-6-8-46-44-116748/1481082405003-816-145.1583065-Neutral.htm) |
| 399 | 864.42 | 539.19087 | 541.19735 | 305.13255 | 2.00648 | 28775 | 1 | 1 | [7](http://www.mycompoundid.org/mycompoundid_IsoMS/myid_search_res/1481082404607-2016-12-6-8-46-44-116748/1481082405003-973-305.1325488-Neutral.htm) |
| 400 | 866.68 | 335.10656 | 337.11286 | 101.04824 | 2.00630 | 172125 | 1 | 1 | [29](http://www.mycompoundid.org/mycompoundid_IsoMS/myid_search_res/1481082404607-2016-12-6-8-46-44-116748/1481082405004-547-101.0482437-Neutral.htm) |
| 401 | 867.32 | 597.11651 | 599.12135 | 363.05819 | 2.00484 | 37600 | 1 | 1 | [24](http://www.mycompoundid.org/mycompoundid_IsoMS/myid_search_res/1481082404607-2016-12-6-8-46-44-116748/1481082405005-952-363.058187-Neutral.htm) |
| 402 | 867.40 | 626.28276 | 630.29547 | 159.17342 | 4.01272 | 6790 | 1 | 2 | [2](http://www.mycompoundid.org/mycompoundid_IsoMS/myid_search_res/1481082404607-2016-12-6-8-46-44-116748/1481082405007-218-159.173418-Neutral.htm) |
| 403 | 867.75 | 322.07429 | 324.08036 | 88.01597 | 2.00607 | 19650 | 1 | 1 | [30](http://www.mycompoundid.org/mycompoundid_IsoMS/myid_search_res/1481082404607-2016-12-6-8-46-44-116748/1481082405007-481-88.015965-Neutral.htm) |
| 404 | 870.47 | 442.16381 | 444.17074 | 208.10549 | 2.00692 | 19975 | 1 | 1 | [3](http://www.mycompoundid.org/mycompoundid_IsoMS/myid_search_res/1481082404607-2016-12-6-8-46-44-116748/1481082405008-45-208.1054945-Neutral.htm) |
| # | RT (s) | mz_light | mz_heavy | mz | distance | int_light | nCharge | nTag | Possible hits |
| 405 | 871.61 | 300.08643 | 302.09315 | 132.05623 | 2.00671 | 9500 | 2 | 2 | [8](http://www.mycompoundid.org/mycompoundid_IsoMS/myid_search_res/1481082404607-2016-12-6-8-46-44-116748/1481082405009-587-132.056228-Neutral.htm) |
| 406 | 872.61 | 309.58359 | 311.58829 | 151.05055 | 2.00469 | 13000 | 2 | 2 | [11](http://www.mycompoundid.org/mycompoundid_IsoMS/myid_search_res/1481082404607-2016-12-6-8-46-44-116748/1481082405009-989-151.050546-Neutral.htm) |
| 407 | 873.34 | 549.16267 | 551.16896 | 315.10435 | 2.00628 | 10920 | 1 | 1 | [21](http://www.mycompoundid.org/mycompoundid_IsoMS/myid_search_res/1481082404607-2016-12-6-8-46-44-116748/1481082405010-751-315.1043543-Neutral.htm) |
| 408 | 873.43 | 344.14207 | 346.14833 | 110.08375 | 2.00627 | 31025 | 1 | 1 | [3](http://www.mycompoundid.org/mycompoundid_IsoMS/myid_search_res/1481082404607-2016-12-6-8-46-44-116748/1481082405010-977-110.0837471-Neutral.htm) |
| 409 | 875.13 | 404.19996 | 406.20562 | 170.14164 | 2.00566 | 38622 | 1 | 1 | [4](http://www.mycompoundid.org/mycompoundid_IsoMS/myid_search_res/1481082404607-2016-12-6-8-46-44-116748/1481082405011-324-170.1416371-Neutral.htm) |
| 410 | 876.59 | 320.65375 | 322.66024 | 173.19086 | 2.00649 | 39325 | 2 | 2 | [3](http://www.mycompoundid.org/mycompoundid_IsoMS/myid_search_res/1481082404607-2016-12-6-8-46-44-116748/1481082405011-17-173.1908645-Neutral.htm) |
| 411 | 876.64 | 531.15325 | 533.16055 | 297.09493 | 2.00730 | 10400 | 1 | 1 | [8](http://www.mycompoundid.org/mycompoundid_IsoMS/myid_search_res/1481082404607-2016-12-6-8-46-44-116748/1481082405011-849-297.094929-Neutral.htm) |
| 412 | 876.80 | 373.62652 | 375.63122 | 279.13640 | 2.00471 | 16400 | 2 | 2 | [1](http://www.mycompoundid.org/mycompoundid_IsoMS/myid_search_res/1481082404607-2016-12-6-8-46-44-116748/1481082405012-806-279.136398-Neutral.htm) |
| 413 | 881.41 | 531.16020 | 533.16827 | 297.10188 | 2.00807 | 146000 | 1 | 1 | [3](http://www.mycompoundid.org/mycompoundid_IsoMS/myid_search_res/1481082404607-2016-12-6-8-46-44-116748/1481082405013-957-297.1018828-Neutral.htm) |
| 414 | 882.64 | 532.20464 | 534.21182 | 298.14632 | 2.00718 | 14425 | 1 | 1 | [2](http://www.mycompoundid.org/mycompoundid_IsoMS/myid_search_res/1481082404607-2016-12-6-8-46-44-116748/1481082405013-575-298.1463216-Neutral.htm) |
| 415 | 883.24 | 555.15155 | 557.15731 | 321.09323 | 2.00576 | 231500 | 1 | 1 | [1](http://www.mycompoundid.org/mycompoundid_IsoMS/myid_search_res/1481082404607-2016-12-6-8-46-44-116748/1481082405013-588-321.093229-Neutral.htm) |
| 416 | 883.89 | 641.33796 | 643.34475 | 407.27964 | 2.00679 | 389486 | 1 | 1 | [4](http://www.mycompoundid.org/mycompoundid_IsoMS/myid_search_res/1481082404607-2016-12-6-8-46-44-116748/1481082405014-818-407.2796402-Neutral.htm) |
| 417 | 886.90 | 311.14183 | 313.14841 | 77.08351 | 2.00658 | 32300 | 1 | 1 | [6](http://www.mycompoundid.org/mycompoundid_IsoMS/myid_search_res/1481082404607-2016-12-6-8-46-44-116748/1481082405014-189-77.083505-Neutral.htm) |
| 418 | 887.71 | 577.15819 | 579.16426 | 343.09987 | 2.00606 | 28050 | 1 | 1 | [15](http://www.mycompoundid.org/mycompoundid_IsoMS/myid_search_res/1481082404607-2016-12-6-8-46-44-116748/1481082405014-257-343.0998713-Neutral.htm) |
| 419 | 888.22 | 416.11709 | 418.12230 | 182.05877 | 2.00521 | 12350 | 1 | 1 | [45](http://www.mycompoundid.org/mycompoundid_IsoMS/myid_search_res/1481082404607-2016-12-6-8-46-44-116748/1481082405015-408-182.0587673-Neutral.htm) |
| 420 | 892.63 | 612.26689 | 616.27972 | 145.15755 | 4.01283 | 15275 | 1 | 2 | [3](http://www.mycompoundid.org/mycompoundid_IsoMS/myid_search_res/1481082404607-2016-12-6-8-46-44-116748/1481082405017-154-145.1575525-Neutral.htm) |
| 421 | 892.89 | 629.21068 | 633.22377 | 162.10134 | 4.01309 | 43625 | 1 | 2 | [15](http://www.mycompoundid.org/mycompoundid_IsoMS/myid_search_res/1481082404607-2016-12-6-8-46-44-116748/1481082405017-771-162.1013438-Neutral.htm) |
| 422 | 893.52 | 547.15553 | 549.16331 | 313.09721 | 2.00778 | 113909 | 1 | 1 | [1](http://www.mycompoundid.org/mycompoundid_IsoMS/myid_search_res/1481082404607-2016-12-6-8-46-44-116748/1481082405018-96-313.0972135-Neutral.htm) |
| 423 | 893.58 | 462.20597 | 464.21145 | 228.14765 | 2.00548 | 21413 | 1 | 1 | [3](http://www.mycompoundid.org/mycompoundid_IsoMS/myid_search_res/1481082404607-2016-12-6-8-46-44-116748/1481082405018-307-228.1476499-Neutral.htm) |
| 424 | 894.27 | 575.15253 | 577.15904 | 341.09421 | 2.00651 | 157531 | 1 | 1 | [17](http://www.mycompoundid.org/mycompoundid_IsoMS/myid_search_res/1481082404607-2016-12-6-8-46-44-116748/1481082405019-852-341.0942147-Neutral.htm) |
| 425 | 898.52 | 307.09538 | 309.10136 | 146.07412 | 2.00598 | 37775 | 2 | 2 | [10](http://www.mycompoundid.org/mycompoundid_IsoMS/myid_search_res/1481082404607-2016-12-6-8-46-44-116748/1481082405020-680-146.0741154-Neutral.htm) |
| 426 | 898.76 | 296.09557 | 298.10130 | 62.03725 | 2.00573 | 36038 | 1 | 1 | [19](http://www.mycompoundid.org/mycompoundid_IsoMS/myid_search_res/1481082404607-2016-12-6-8-46-44-116748/1481082405020-956-62.03725063-Neutral.htm) |
| 427 | 901.44 | 355.07114 | 357.07674 | 242.02564 | 2.00560 | 25100 | 2 | 2 | [1](http://www.mycompoundid.org/mycompoundid_IsoMS/myid_search_res/1481082404607-2016-12-6-8-46-44-116748/1481082405021-526-242.0256425-Neutral.htm) |
| 428 | 901.65 | 377.10569 | 379.11283 | 286.09474 | 2.00714 | 14888 | 2 | 2 | [3](http://www.mycompoundid.org/mycompoundid_IsoMS/myid_search_res/1481082404607-2016-12-6-8-46-44-116748/1481082405021-428-286.0947385-Neutral.htm) |
| # | RT (s) | mz_light | mz_heavy | mz | distance | int_light | nCharge | nTag | Possible hits |
| 429 | 902.66 | 577.16032 | 579.16296 | 343.10200 | 2.00264 | 22900 | 1 | 1 | [16](http://www.mycompoundid.org/mycompoundid_IsoMS/myid_search_res/1481082404607-2016-12-6-8-46-44-116748/1481082405022-252-343.101998-Neutral.htm) |
| 430 | 902.75 | 354.07128 | 356.07781 | 240.02593 | 2.00653 | 182000 | 2 | 2 | [3](http://www.mycompoundid.org/mycompoundid_IsoMS/myid_search_res/1481082404607-2016-12-6-8-46-44-116748/1481082405022-909-240.0259275-Neutral.htm) |
| 431 | 903.66 | 547.15317 | 549.16149 | 313.09485 | 2.00832 | 25900 | 1 | 1 | [7](http://www.mycompoundid.org/mycompoundid_IsoMS/myid_search_res/1481082404607-2016-12-6-8-46-44-116748/1481082405023-972-313.094848-Neutral.htm) |
| 432 | 905.14 | 553.14761 | 555.15005 | 319.08929 | 2.00244 | 36900 | 1 | 1 | [8](http://www.mycompoundid.org/mycompoundid_IsoMS/myid_search_res/1481082404607-2016-12-6-8-46-44-116748/1481082405024-161-319.0892855-Neutral.htm) |
| 433 | 905.49 | 387.10050 | 389.10642 | 153.04218 | 2.00592 | 48838 | 1 | 1 | [20](http://www.mycompoundid.org/mycompoundid_IsoMS/myid_search_res/1481082404607-2016-12-6-8-46-44-116748/1481082405024-557-153.0421832-Neutral.htm) |
| 434 | 905.67 | 630.22134 | 632.22736 | 396.16302 | 2.00602 | 7580 | 1 | 1 | [16](http://www.mycompoundid.org/mycompoundid_IsoMS/myid_search_res/1481082404607-2016-12-6-8-46-44-116748/1481082405025-632-396.163023-Neutral.htm) |
| 435 | 905.90 | 408.19522 | 410.20151 | 174.13690 | 2.00629 | 32319 | 1 | 1 | [9](http://www.mycompoundid.org/mycompoundid_IsoMS/myid_search_res/1481082404607-2016-12-6-8-46-44-116748/1481082405026-661-174.136898-Neutral.htm) |
| 436 | 905.98 | 363.13816 | 365.14459 | 129.07984 | 2.00643 | 3792500 | 1 | 1 | [30](http://www.mycompoundid.org/mycompoundid_IsoMS/myid_search_res/1481082404607-2016-12-6-8-46-44-116748/1481082405026-885-129.0798373-Neutral.htm) |
| 437 | 909.12 | 385.12002 | 387.12659 | 151.06170 | 2.00657 | 239100 | 1 | 1 | [1](http://www.mycompoundid.org/mycompoundid_IsoMS/myid_search_res/1481082404607-2016-12-6-8-46-44-116748/1481082405027-42-151.0617045-Neutral.htm) |
| 438 | 911.64 | 612.26781 | 616.28023 | 145.15847 | 4.01241 | 50188 | 1 | 2 | [3](http://www.mycompoundid.org/mycompoundid_IsoMS/myid_search_res/1481082404607-2016-12-6-8-46-44-116748/1481082405028-168-145.1584748-Neutral.htm) |
| 439 | 911.76 | 549.16349 | 551.16956 | 315.10517 | 2.00607 | 36300 | 1 | 1 | [25](http://www.mycompoundid.org/mycompoundid_IsoMS/myid_search_res/1481082404607-2016-12-6-8-46-44-116748/1481082405028-245-315.105168-Neutral.htm) |
| 440 | 913.07 | 375.60561 | 377.61117 | 283.09459 | 2.00556 | 145594 | 2 | 2 | [5](http://www.mycompoundid.org/mycompoundid_IsoMS/myid_search_res/1481082404607-2016-12-6-8-46-44-116748/1481082405029-701-283.0945853-Neutral.htm) |
| 441 | 913.71 | 311.08429 | 313.09097 | 154.05195 | 2.00668 | 564250 | 2 | 2 | [2](http://www.mycompoundid.org/mycompoundid_IsoMS/myid_search_res/1481082404607-2016-12-6-8-46-44-116748/1481082405030-346-154.0519475-Neutral.htm) |
| 442 | 914.91 | 368.11636 | 370.12331 | 268.11608 | 2.00695 | 29650 | 2 | 2 | [11](http://www.mycompoundid.org/mycompoundid_IsoMS/myid_search_res/1481082404607-2016-12-6-8-46-44-116748/1481082405030-133-268.116082-Neutral.htm) |
| 443 | 915.53 | 419.10733 | 421.11317 | 185.04901 | 2.00583 | 19150 | 1 | 1 | [1](http://www.mycompoundid.org/mycompoundid_IsoMS/myid_search_res/1481082404607-2016-12-6-8-46-44-116748/1481082405031-230-185.0490138-Neutral.htm) |
| 444 | 918.93 | 553.14887 | 555.15162 | 319.09055 | 2.00274 | 15400 | 1 | 1 | [8](http://www.mycompoundid.org/mycompoundid_IsoMS/myid_search_res/1481082404607-2016-12-6-8-46-44-116748/1481082405031-329-319.090552-Neutral.htm) |
| 445 | 920.46 | 547.15576 | 549.16231 | 313.09744 | 2.00655 | 51000 | 1 | 1 | [1](http://www.mycompoundid.org/mycompoundid_IsoMS/myid_search_res/1481082404607-2016-12-6-8-46-44-116748/1481082405032-30-313.097435-Neutral.htm) |
| 446 | 922.46 | 377.10491 | 379.11250 | 286.09318 | 2.00759 | 28931 | 2 | 2 | [9](http://www.mycompoundid.org/mycompoundid_IsoMS/myid_search_res/1481082404607-2016-12-6-8-46-44-116748/1481082405032-325-286.0931841-Neutral.htm) |
| 447 | 922.66 | 581.14068 | 585.15624 | 114.03134 | 4.01556 | 6650 | 1 | 2 | [28](http://www.mycompoundid.org/mycompoundid_IsoMS/myid_search_res/1481082404607-2016-12-6-8-46-44-116748/1481082405032-503-114.031336-Neutral.htm) |
| 448 | 927.44 | 502.09665 | 504.10388 | 268.03833 | 2.00723 | 19863 | 1 | 1 | [8](http://www.mycompoundid.org/mycompoundid_IsoMS/myid_search_res/1481082404607-2016-12-6-8-46-44-116748/1481082405033-376-268.0383318-Neutral.htm) |
| 449 | 928.09 | 496.18981 | 498.19654 | 262.13149 | 2.00673 | 18156 | 1 | 1 | [6](http://www.mycompoundid.org/mycompoundid_IsoMS/myid_search_res/1481082404607-2016-12-6-8-46-44-116748/1481082405034-145-262.1314868-Neutral.htm) |
| 450 | 929.06 | 393.14769 | 395.15427 | 159.08937 | 2.00658 | 33403 | 1 | 1 | [42](http://www.mycompoundid.org/mycompoundid_IsoMS/myid_search_res/1481082404607-2016-12-6-8-46-44-116748/1481082405034-863-159.0893706-Neutral.htm) |
| 451 | 931.87 | 335.62226 | 337.62879 | 203.12789 | 2.00653 | 24719 | 2 | 2 | [11](http://www.mycompoundid.org/mycompoundid_IsoMS/myid_search_res/1481082404607-2016-12-6-8-46-44-116748/1481082405036-848-203.127889-Neutral.htm) |
| 452 | 931.97 | 431.12581 | 433.13110 | 197.06749 | 2.00529 | 20250 | 1 | 1 | [39](http://www.mycompoundid.org/mycompoundid_IsoMS/myid_search_res/1481082404607-2016-12-6-8-46-44-116748/1481082405037-585-197.067485-Neutral.htm) |
| # | RT (s) | mz_light | mz_heavy | mz | distance | int_light | nCharge | nTag | Possible hits |
| 453 | 932.57 | 349.61986 | 351.62644 | 231.12307 | 2.00659 | 39338 | 2 | 2 | [5](http://www.mycompoundid.org/mycompoundid_IsoMS/myid_search_res/1481082404607-2016-12-6-8-46-44-116748/1481082405038-672-231.123071-Neutral.htm) |
| 454 | 938.40 | 411.10457 | 413.11191 | 177.04625 | 2.00734 | 8470 | 1 | 1 | [16](http://www.mycompoundid.org/mycompoundid_IsoMS/myid_search_res/1481082404607-2016-12-6-8-46-44-116748/1481082405038-697-177.046254-Neutral.htm) |
| 455 | 938.70 | 330.12701 | 332.13301 | 96.06869 | 2.00601 | 38466 | 1 | 1 | [7](http://www.mycompoundid.org/mycompoundid_IsoMS/myid_search_res/1481082404607-2016-12-6-8-46-44-116748/1481082405039-722-96.06868628-Neutral.htm) |
| 456 | 938.75 | 369.10803 | 371.11399 | 270.09942 | 2.00596 | 15325 | 2 | 2 | [2](http://www.mycompoundid.org/mycompoundid_IsoMS/myid_search_res/1481082404607-2016-12-6-8-46-44-116748/1481082405040-629-270.0994215-Neutral.htm) |
| 457 | 939.31 | 531.15215 | 533.15871 | 297.09383 | 2.00656 | 28613 | 1 | 1 | [5](http://www.mycompoundid.org/mycompoundid_IsoMS/myid_search_res/1481082404607-2016-12-6-8-46-44-116748/1481082405040-288-297.0938343-Neutral.htm) |
| 458 | 940.82 | 502.09687 | 504.10423 | 268.03855 | 2.00736 | 17363 | 1 | 1 | [8](http://www.mycompoundid.org/mycompoundid_IsoMS/myid_search_res/1481082404607-2016-12-6-8-46-44-116748/1481082405040-678-268.0385485-Neutral.htm) |
| 459 | 946.26 | 588.11535 | 590.12096 | 354.05703 | 2.00561 | 22850 | 1 | 1 | [4](http://www.mycompoundid.org/mycompoundid_IsoMS/myid_search_res/1481082404607-2016-12-6-8-46-44-116748/1481082405041-469-354.0570301-Neutral.htm) |
| 460 | 946.41 | 376.11681 | 378.12213 | 284.11699 | 2.00532 | 5010 | 2 | 2 | [2](http://www.mycompoundid.org/mycompoundid_IsoMS/myid_search_res/1481082404607-2016-12-6-8-46-44-116748/1481082405041-714-284.116986-Neutral.htm) |
| 461 | 948.03 | 612.26605 | 616.27923 | 145.15671 | 4.01318 | 11100 | 1 | 2 | [3](http://www.mycompoundid.org/mycompoundid_IsoMS/myid_search_res/1481082404607-2016-12-6-8-46-44-116748/1481082405042-768-145.1567098-Neutral.htm) |
| 462 | 949.05 | 354.10245 | 356.10966 | 240.08827 | 2.00720 | 5845 | 2 | 2 | [5](http://www.mycompoundid.org/mycompoundid_IsoMS/myid_search_res/1481082404607-2016-12-6-8-46-44-116748/1481082405042-163-240.088267-Neutral.htm) |
| 463 | 949.68 | 396.19421 | 398.20137 | 162.13589 | 2.00716 | 14300 | 1 | 1 | [5](http://www.mycompoundid.org/mycompoundid_IsoMS/myid_search_res/1481082404607-2016-12-6-8-46-44-116748/1481082405044-680-162.13589-Neutral.htm) |
| 464 | 949.91 | 351.13795 | 353.14454 | 117.07963 | 2.00659 | 612125 | 1 | 1 | [38](http://www.mycompoundid.org/mycompoundid_IsoMS/myid_search_res/1481082404607-2016-12-6-8-46-44-116748/1481082405045-91-117.0796295-Neutral.htm) |
| 465 | 951.93 | 473.13769 | 475.14418 | 239.07937 | 2.00649 | 62000 | 1 | 1 | [17](http://www.mycompoundid.org/mycompoundid_IsoMS/myid_search_res/1481082404607-2016-12-6-8-46-44-116748/1481082405046-778-239.0793716-Neutral.htm) |
| 466 | 952.27 | 395.10982 | 397.11534 | 161.05150 | 2.00552 | 177273 | 1 | 1 | [4](http://www.mycompoundid.org/mycompoundid_IsoMS/myid_search_res/1481082404607-2016-12-6-8-46-44-116748/1481082405047-956-161.0514957-Neutral.htm) |
| 467 | 952.50 | 373.62452 | 375.63130 | 279.13240 | 2.00677 | 17275 | 2 | 2 | [15](http://www.mycompoundid.org/mycompoundid_IsoMS/myid_search_res/1481082404607-2016-12-6-8-46-44-116748/1481082405048-131-279.1324035-Neutral.htm) |
| 468 | 952.81 | 531.15248 | 533.15894 | 297.09416 | 2.00647 | 19200 | 1 | 1 | [5](http://www.mycompoundid.org/mycompoundid_IsoMS/myid_search_res/1481082404607-2016-12-6-8-46-44-116748/1481082405048-446-297.094155-Neutral.htm) |
| 469 | 954.46 | 975.42724 | 977.43386 | 741.36892 | 2.00663 | 66403 | 1 | 1 | [1](http://www.mycompoundid.org/mycompoundid_IsoMS/myid_search_res/1481082404607-2016-12-6-8-46-44-116748/1481082405049-9-741.3689181-Neutral.htm) |
| 470 | 954.70 | 410.21035 | 412.21610 | 176.15203 | 2.00575 | 16000 | 1 | 1 | [3](http://www.mycompoundid.org/mycompoundid_IsoMS/myid_search_res/1481082404607-2016-12-6-8-46-44-116748/1481082405049-919-176.152034-Neutral.htm) |
| 471 | 957.72 | 350.64614 | 352.65257 | 233.17564 | 2.00643 | 42431 | 2 | 2 | [2](http://www.mycompoundid.org/mycompoundid_IsoMS/myid_search_res/1481082404607-2016-12-6-8-46-44-116748/1481082405050-493-233.1756425-Neutral.htm) |
| 472 | 961.60 | 336.09837 | 338.10343 | 204.08010 | 2.00506 | 12000 | 2 | 2 | [2](http://www.mycompoundid.org/mycompoundid_IsoMS/myid_search_res/1481082404607-2016-12-6-8-46-44-116748/1481082405051-381-204.080095-Neutral.htm) |
| 473 | 961.72 | 384.14912 | 386.15526 | 300.18161 | 2.00614 | 26781 | 2 | 2 | [1](http://www.mycompoundid.org/mycompoundid_IsoMS/myid_search_res/1481082404607-2016-12-6-8-46-44-116748/1481082405051-241-300.1816054-Neutral.htm) |
| 474 | 962.70 | 547.15402 | 549.16222 | 313.09570 | 2.00820 | 16863 | 1 | 1 | [5](http://www.mycompoundid.org/mycompoundid_IsoMS/myid_search_res/1481082404607-2016-12-6-8-46-44-116748/1481082405051-602-313.0957011-Neutral.htm) |
| 475 | 963.36 | 406.16400 | 408.17079 | 344.21136 | 2.00679 | 10100 | 2 | 2 | [1](http://www.mycompoundid.org/mycompoundid_IsoMS/myid_search_res/1481082404607-2016-12-6-8-46-44-116748/1481082405052-574-344.211358-Neutral.htm) |
| 476 | 964.43 | 652.27057 | 654.27866 | 418.21225 | 2.00809 | 85325 | 1 | 1 | [6](http://www.mycompoundid.org/mycompoundid_IsoMS/myid_search_res/1481082404607-2016-12-6-8-46-44-116748/1481082405053-506-418.212247-Neutral.htm) |
| # | RT (s) | mz_light | mz_heavy | mz | distance | int_light | nCharge | nTag | Possible hits |
| 477 | 965.82 | 549.16203 | 551.16848 | 315.10371 | 2.00645 | 12750 | 1 | 1 | [22](http://www.mycompoundid.org/mycompoundid_IsoMS/myid_search_res/1481082404607-2016-12-6-8-46-44-116748/1481082405053-65-315.103714-Neutral.htm) |
| 478 | 965.91 | 638.28272 | 640.28315 | 404.22440 | 2.00042 | 14600 | 1 | 1 | [3](http://www.mycompoundid.org/mycompoundid_IsoMS/myid_search_res/1481082404607-2016-12-6-8-46-44-116748/1481082405054-643-404.224404-Neutral.htm) |
| 479 | 966.44 | 423.16065 | 425.16662 | 189.10233 | 2.00597 | 941125 | 1 | 1 | [2](http://www.mycompoundid.org/mycompoundid_IsoMS/myid_search_res/1481082404607-2016-12-6-8-46-44-116748/1481082405054-625-189.1023316-Neutral.htm) |
| 480 | 966.80 | 468.21629 | 470.22236 | 234.15797 | 2.00607 | 17025 | 1 | 1 | [3](http://www.mycompoundid.org/mycompoundid_IsoMS/myid_search_res/1481082404607-2016-12-6-8-46-44-116748/1481082405055-876-234.1579709-Neutral.htm) |
| 481 | 966.82 | 501.11493 | 505.12830 | 34.00559 | 4.01336 | 60559 | 1 | 2 | [8](http://www.mycompoundid.org/mycompoundid_IsoMS/myid_search_res/1481082404607-2016-12-6-8-46-44-116748/1481082405055-114-34.00559384-Neutral.htm) |
| 482 | 966.88 | 368.09964 | 370.10638 | 268.08265 | 2.00673 | 61681 | 2 | 2 | [19](http://www.mycompoundid.org/mycompoundid_IsoMS/myid_search_res/1481082404607-2016-12-6-8-46-44-116748/1481082405056-949-268.0826471-Neutral.htm) |
| 483 | 967.62 | 288.57292 | 290.57910 | 109.02919 | 2.00618 | 8278 | 2 | 2 | [1](http://www.mycompoundid.org/mycompoundid_IsoMS/myid_search_res/1481082404607-2016-12-6-8-46-44-116748/1481082405056-479-109.0291915-Neutral.htm) |
| 484 | 967.71 | 369.10361 | 371.11033 | 270.09058 | 2.00672 | 16900 | 2 | 2 | [9](http://www.mycompoundid.org/mycompoundid_IsoMS/myid_search_res/1481082404607-2016-12-6-8-46-44-116748/1481082405057-828-270.090579-Neutral.htm) |
| 485 | 967.73 | 658.32700 | 660.32767 | 424.26868 | 2.00067 | 8040 | 1 | 1 | [13](http://www.mycompoundid.org/mycompoundid_IsoMS/myid_search_res/1481082404607-2016-12-6-8-46-44-116748/1481082405057-546-424.268679-Neutral.htm) |
| 486 | 967.86 | 413.15322 | 415.16196 | 179.09490 | 2.00874 | 26650 | 1 | 1 | [27](http://www.mycompoundid.org/mycompoundid_IsoMS/myid_search_res/1481082404607-2016-12-6-8-46-44-116748/1481082405058-19-179.094899-Neutral.htm) |
| 487 | 969.36 | 405.13650 | 407.14416 | 342.15636 | 2.00766 | 6730 | 2 | 2 | [10](http://www.mycompoundid.org/mycompoundid_IsoMS/myid_search_res/1481082404607-2016-12-6-8-46-44-116748/1481082405059-911-342.156356-Neutral.htm) |
| 488 | 970.53 | 439.13607 | 441.14411 | 205.07775 | 2.00804 | 19925 | 1 | 1 | [9](http://www.mycompoundid.org/mycompoundid_IsoMS/myid_search_res/1481082404607-2016-12-6-8-46-44-116748/1481082405060-178-205.077751-Neutral.htm) |
| 489 | 971.73 | 311.59287 | 313.59969 | 155.06910 | 2.00682 | 77994 | 2 | 2 | [14](http://www.mycompoundid.org/mycompoundid_IsoMS/myid_search_res/1481082404607-2016-12-6-8-46-44-116748/1481082405061-149-155.0691033-Neutral.htm) |
| 490 | 972.10 | 652.28883 | 654.29284 | 418.23051 | 2.00402 | 9550 | 1 | 1 | [2](http://www.mycompoundid.org/mycompoundid_IsoMS/myid_search_res/1481082404607-2016-12-6-8-46-44-116748/1481082405061-836-418.2305073-Neutral.htm) |
| 491 | 972.44 | 680.28829 | 682.29486 | 446.22997 | 2.00657 | 50625 | 1 | 1 | [6](http://www.mycompoundid.org/mycompoundid_IsoMS/myid_search_res/1481082404607-2016-12-6-8-46-44-116748/1481082405062-565-446.229967-Neutral.htm) |
| 492 | 975.74 | 371.63248 | 373.63850 | 275.14832 | 2.00602 | 10238 | 2 | 2 | [10](http://www.mycompoundid.org/mycompoundid_IsoMS/myid_search_res/1481082404607-2016-12-6-8-46-44-116748/1481082405062-387-275.148322-Neutral.htm) |
| 493 | 976.01 | 547.15392 | 549.16252 | 313.09560 | 2.00860 | 13400 | 1 | 1 | [5](http://www.mycompoundid.org/mycompoundid_IsoMS/myid_search_res/1481082404607-2016-12-6-8-46-44-116748/1481082405063-387-313.0956015-Neutral.htm) |
| 494 | 976.26 | 654.31392 | 658.32739 | 187.20458 | 4.01347 | 15910 | 1 | 2 | [3](http://www.mycompoundid.org/mycompoundid_IsoMS/myid_search_res/1481082404607-2016-12-6-8-46-44-116748/1481082405063-401-187.2045828-Neutral.htm) |
| 495 | 977.06 | 342.63030 | 344.63687 | 217.14397 | 2.00657 | 14375 | 2 | 2 | [6](http://www.mycompoundid.org/mycompoundid_IsoMS/myid_search_res/1481082404607-2016-12-6-8-46-44-116748/1481082405064-172-217.143967-Neutral.htm) |
| 496 | 978.92 | 656.31406 | 658.32610 | 422.25574 | 2.01205 | 11700 | 1 | 1 | [5](http://www.mycompoundid.org/mycompoundid_IsoMS/myid_search_res/1481082404607-2016-12-6-8-46-44-116748/1481082405064-420-422.255735-Neutral.htm) |
| 497 | 980.38 | 346.09891 | 348.10579 | 224.08118 | 2.00688 | 10500 | 2 | 2 | [36](http://www.mycompoundid.org/mycompoundid_IsoMS/myid_search_res/1481082404607-2016-12-6-8-46-44-116748/1481082405065-986-224.08118-Neutral.htm) |
| 498 | 980.97 | 300.10391 | 302.11049 | 132.09119 | 2.00657 | 40300 | 2 | 2 | [17](http://www.mycompoundid.org/mycompoundid_IsoMS/myid_search_res/1481082404607-2016-12-6-8-46-44-116748/1481082405066-84-132.091186-Neutral.htm) |
| 499 | 984.42 | 421.15291 | 423.16061 | 187.09459 | 2.00770 | 14750 | 1 | 1 | [7](http://www.mycompoundid.org/mycompoundid_IsoMS/myid_search_res/1481082404607-2016-12-6-8-46-44-116748/1481082405067-290-187.0945925-Neutral.htm) |
| 500 | 986.49 | 659.25008 | 661.25609 | 425.19176 | 2.00602 | 25250 | 1 | 1 | [11](http://www.mycompoundid.org/mycompoundid_IsoMS/myid_search_res/1481082404607-2016-12-6-8-46-44-116748/1481082405068-912-425.1917581-Neutral.htm) |
| # | RT (s) | mz_light | mz_heavy | mz | distance | int_light | nCharge | nTag | Possible hits |
| 501 | 987.45 | 494.68875 | 496.69480 | 521.26086 | 2.00605 | 6890 | 2 | 2 | [8](http://www.mycompoundid.org/mycompoundid_IsoMS/myid_search_res/1481082404607-2016-12-6-8-46-44-116748/1481082405069-345-521.260862-Neutral.htm) |
| 502 | 990.39 | 395.12300 | 397.13138 | 322.12937 | 2.00837 | 7260 | 2 | 2 | [8](http://www.mycompoundid.org/mycompoundid_IsoMS/myid_search_res/1481082404607-2016-12-6-8-46-44-116748/1481082405070-422-322.129368-Neutral.htm) |
| 503 | 993.03 | 553.14345 | 555.14980 | 319.08513 | 2.00635 | 12250 | 1 | 1 | [4](http://www.mycompoundid.org/mycompoundid_IsoMS/myid_search_res/1481082404607-2016-12-6-8-46-44-116748/1481082405070-102-319.0851255-Neutral.htm) |
| 504 | 994.46 | 297.57796 | 299.58451 | 127.03929 | 2.00654 | 27500 | 2 | 2 | [6](http://www.mycompoundid.org/mycompoundid_IsoMS/myid_search_res/1481082404607-2016-12-6-8-46-44-116748/1481082405070-991-127.039288-Neutral.htm) |
| 505 | 995.99 | 322.11739 | 324.12401 | 176.11815 | 2.00661 | 10248 | 2 | 2 | [1](http://www.mycompoundid.org/mycompoundid_IsoMS/myid_search_res/1481082404607-2016-12-6-8-46-44-116748/1481082405071-582-176.118147-Neutral.htm) |
| 506 | 1001.48 | 488.17759 | 490.18306 | 254.11927 | 2.00548 | 33175 | 1 | 1 | [5](http://www.mycompoundid.org/mycompoundid_IsoMS/myid_search_res/1481082404607-2016-12-6-8-46-44-116748/1481082405071-206-254.1192673-Neutral.htm) |
| 507 | 1004.47 | 445.15332 | 447.16018 | 211.09500 | 2.00686 | 19300 | 1 | 1 | [29](http://www.mycompoundid.org/mycompoundid_IsoMS/myid_search_res/1481082404607-2016-12-6-8-46-44-116748/1481082405072-19-211.094996-Neutral.htm) |
| 508 | 1004.84 | 407.16383 | 409.17006 | 173.10551 | 2.00623 | 44875 | 1 | 1 | [29](http://www.mycompoundid.org/mycompoundid_IsoMS/myid_search_res/1481082404607-2016-12-6-8-46-44-116748/1481082405073-650-173.1055071-Neutral.htm) |
| 509 | 1005.29 | 379.16895 | 381.17392 | 145.11063 | 2.00498 | 26941 | 1 | 1 | [19](http://www.mycompoundid.org/mycompoundid_IsoMS/myid_search_res/1481082404607-2016-12-6-8-46-44-116748/1481082405074-529-145.1106264-Neutral.htm) |
| 510 | 1006.47 | 363.61797 | 365.62474 | 259.11930 | 2.00677 | 8575 | 2 | 2 | [12](http://www.mycompoundid.org/mycompoundid_IsoMS/myid_search_res/1481082404607-2016-12-6-8-46-44-116748/1481082405075-658-259.119296-Neutral.htm) |
| 511 | 1006.92 | 372.09032 | 374.09661 | 138.03200 | 2.00629 | 106109 | 1 | 1 | [19](http://www.mycompoundid.org/mycompoundid_IsoMS/myid_search_res/1481082404607-2016-12-6-8-46-44-116748/1481082405076-718-138.0320001-Neutral.htm) |
| 512 | 1014.64 | 441.14752 | 443.15374 | 207.08920 | 2.00623 | 35781 | 1 | 1 | [25](http://www.mycompoundid.org/mycompoundid_IsoMS/myid_search_res/1481082404607-2016-12-6-8-46-44-116748/1481082405077-105-207.089195-Neutral.htm) |
| 513 | 1016.48 | 427.13226 | 429.13809 | 193.07394 | 2.00583 | 18200 | 1 | 1 | [24](http://www.mycompoundid.org/mycompoundid_IsoMS/myid_search_res/1481082404607-2016-12-6-8-46-44-116748/1481082405078-848-193.073941-Neutral.htm) |
| 514 | 1019.82 | 312.08485 | 314.09115 | 156.05306 | 2.00630 | 839000 | 2 | 2 | [17](http://www.mycompoundid.org/mycompoundid_IsoMS/myid_search_res/1481082404607-2016-12-6-8-46-44-116748/1481082405079-401-156.0530585-Neutral.htm) |
| 515 | 1020.29 | 311.08446 | 313.09110 | 154.05228 | 2.00663 | 6781875 | 2 | 2 | [1](http://www.mycompoundid.org/mycompoundid_IsoMS/myid_search_res/1481082404607-2016-12-6-8-46-44-116748/1481082405080-241-154.0522829-Neutral.htm) |
| 516 | 1022.50 | 308.11324 | 310.11967 | 148.10984 | 2.00643 | 904500 | 2 | 2 | [2](http://www.mycompoundid.org/mycompoundid_IsoMS/myid_search_res/1481082404607-2016-12-6-8-46-44-116748/1481082405081-346-148.1098413-Neutral.htm) |
| 517 | 1026.08 | 457.14136 | 459.14810 | 223.08304 | 2.00675 | 15000 | 1 | 1 | [28](http://www.mycompoundid.org/mycompoundid_IsoMS/myid_search_res/1481082404607-2016-12-6-8-46-44-116748/1481082405082-341-223.083035-Neutral.htm) |
| 518 | 1028.54 | 407.16419 | 409.17041 | 173.10587 | 2.00622 | 146750 | 1 | 1 | [29](http://www.mycompoundid.org/mycompoundid_IsoMS/myid_search_res/1481082404607-2016-12-6-8-46-44-116748/1481082405083-516-173.1058696-Neutral.htm) |
| 519 | 1030.24 | 760.26788 | 762.27474 | 526.20956 | 2.00686 | 84600 | 1 | 1 | [3](http://www.mycompoundid.org/mycompoundid_IsoMS/myid_search_res/1481082404607-2016-12-6-8-46-44-116748/1481082405084-343-526.2095601-Neutral.htm) |
| 520 | 1034.98 | 413.15418 | 415.16000 | 179.09586 | 2.00581 | 573781 | 1 | 1 | [27](http://www.mycompoundid.org/mycompoundid_IsoMS/myid_search_res/1481082404607-2016-12-6-8-46-44-116748/1481082405084-986-179.0958644-Neutral.htm) |
| 521 | 1037.61 | 347.11234 | 349.11957 | 226.10804 | 2.00724 | 13122 | 2 | 2 | [12](http://www.mycompoundid.org/mycompoundid_IsoMS/myid_search_res/1481082404607-2016-12-6-8-46-44-116748/1481082405086-350-226.1080381-Neutral.htm) |
| 522 | 1038.06 | 485.17523 | 487.18131 | 251.11691 | 2.00608 | 8820 | 1 | 1 | [5](http://www.mycompoundid.org/mycompoundid_IsoMS/myid_search_res/1481082404607-2016-12-6-8-46-44-116748/1481082405086-803-251.116906-Neutral.htm) |
| 523 | 1038.57 | 738.22036 | 740.22077 | 504.16204 | 2.00042 | 12800 | 1 | 1 | [2](http://www.mycompoundid.org/mycompoundid_IsoMS/myid_search_res/1481082404607-2016-12-6-8-46-44-116748/1481082405086-268-504.162035-Neutral.htm) |
| 524 | 1039.57 | 367.60760 | 369.61494 | 267.09855 | 2.00734 | 318000 | 2 | 2 | [45](http://www.mycompoundid.org/mycompoundid_IsoMS/myid_search_res/1481082404607-2016-12-6-8-46-44-116748/1481082405087-992-267.098552-Neutral.htm) |
| # | RT (s) | mz_light | mz_heavy | mz | distance | int_light | nCharge | nTag | Possible hits |
| 525 | 1041.76 | 320.63544 | 322.64184 | 173.15424 | 2.00640 | 22450 | 2 | 2 | [5](http://www.mycompoundid.org/mycompoundid_IsoMS/myid_search_res/1481082404607-2016-12-6-8-46-44-116748/1481082405089-126-173.15424-Neutral.htm) |
| 526 | 1042.57 | 356.09962 | 358.10434 | 244.08259 | 2.00472 | 7290 | 2 | 2 | [13](http://www.mycompoundid.org/mycompoundid_IsoMS/myid_search_res/1481082404607-2016-12-6-8-46-44-116748/1481082405089-574-244.082594-Neutral.htm) |
| 527 | 1043.27 | 355.62002 | 357.62708 | 243.12339 | 2.00706 | 10045 | 2 | 2 | [8](http://www.mycompoundid.org/mycompoundid_IsoMS/myid_search_res/1481082404607-2016-12-6-8-46-44-116748/1481082405090-907-243.1233945-Neutral.htm) |
| 528 | 1045.56 | 477.21655 | 479.22447 | 243.15823 | 2.00793 | 19638 | 1 | 1 | [2](http://www.mycompoundid.org/mycompoundid_IsoMS/myid_search_res/1481082404607-2016-12-6-8-46-44-116748/1481082405090-587-243.1582273-Neutral.htm) |
| 529 | 1047.28 | 375.60520 | 377.61174 | 283.09376 | 2.00654 | 88753 | 2 | 2 | [40](http://www.mycompoundid.org/mycompoundid_IsoMS/myid_search_res/1481082404607-2016-12-6-8-46-44-116748/1481082405091-744-283.0937638-Neutral.htm) |
| 530 | 1048.45 | 409.12535 | 411.13071 | 175.06703 | 2.00536 | 65332 | 1 | 1 | [6](http://www.mycompoundid.org/mycompoundid_IsoMS/myid_search_res/1481082404607-2016-12-6-8-46-44-116748/1481082405093-646-175.0670336-Neutral.htm) |
| 531 | 1048.79 | 366.10141 | 368.10676 | 132.04309 | 2.00535 | 34500 | 1 | 1 | [69](http://www.mycompoundid.org/mycompoundid_IsoMS/myid_search_res/1481082404607-2016-12-6-8-46-44-116748/1481082405093-723-132.043086-Neutral.htm) |
| 532 | 1056.57 | 444.15898 | 446.16564 | 210.10066 | 2.00666 | 13613 | 1 | 1 | [23](http://www.mycompoundid.org/mycompoundid_IsoMS/myid_search_res/1481082404607-2016-12-6-8-46-44-116748/1481082405095-94-210.1006611-Neutral.htm) |
| 533 | 1058.57 | 355.63779 | 357.64365 | 243.15894 | 2.00585 | 23969 | 2 | 2 | [2](http://www.mycompoundid.org/mycompoundid_IsoMS/myid_search_res/1481082404607-2016-12-6-8-46-44-116748/1481082405096-206-243.1589434-Neutral.htm) |
| 534 | 1059.90 | 424.22565 | 426.23126 | 190.16733 | 2.00561 | 10000 | 1 | 1 | [1](http://www.mycompoundid.org/mycompoundid_IsoMS/myid_search_res/1481082404607-2016-12-6-8-46-44-116748/1481082405096-861-190.1673295-Neutral.htm) |
| 535 | 1060.28 | 652.29872 | 656.31119 | 185.18938 | 4.01247 | 50459 | 1 | 2 | [1](http://www.mycompoundid.org/mycompoundid_IsoMS/myid_search_res/1481082404607-2016-12-6-8-46-44-116748/1481082405097-916-185.1893801-Neutral.htm) |
| 536 | 1061.25 | 728.24226 | 730.24889 | 494.18394 | 2.00663 | 198156 | 1 | 1 | [3](http://www.mycompoundid.org/mycompoundid_IsoMS/myid_search_res/1481082404607-2016-12-6-8-46-44-116748/1481082405097-123-494.1839408-Neutral.htm) |
| 537 | 1061.46 | 347.14263 | 349.14677 | 113.08431 | 2.00415 | 7780 | 1 | 1 | [16](http://www.mycompoundid.org/mycompoundid_IsoMS/myid_search_res/1481082404607-2016-12-6-8-46-44-116748/1481082405097-45-113.084307-Neutral.htm) |
| 538 | 1063.17 | 379.16995 | 381.17633 | 145.11163 | 2.00639 | 2143750 | 1 | 1 | [19](http://www.mycompoundid.org/mycompoundid_IsoMS/myid_search_res/1481082404607-2016-12-6-8-46-44-116748/1481082405099-382-145.1116256-Neutral.htm) |
| 539 | 1063.71 | 341.11110 | 343.11957 | 214.10556 | 2.00847 | 21950 | 2 | 2 | [1](http://www.mycompoundid.org/mycompoundid_IsoMS/myid_search_res/1481082404607-2016-12-6-8-46-44-116748/1481082405100-443-214.105557-Neutral.htm) |
| 540 | 1066.14 | 389.12813 | 391.13395 | 155.06981 | 2.00582 | 90838 | 1 | 1 | [14](http://www.mycompoundid.org/mycompoundid_IsoMS/myid_search_res/1481082404607-2016-12-6-8-46-44-116748/1481082405100-300-155.0698063-Neutral.htm) |
| 541 | 1067.29 | 312.59492 | 314.60143 | 157.07320 | 2.00651 | 563481 | 2 | 2 | [28](http://www.mycompoundid.org/mycompoundid_IsoMS/myid_search_res/1481082404607-2016-12-6-8-46-44-116748/1481082405102-645-157.0732031-Neutral.htm) |
| 542 | 1069.45 | 934.41386 | 936.42235 | 700.35554 | 2.00849 | 5980 | 1 | 1 | [5](http://www.mycompoundid.org/mycompoundid_IsoMS/myid_search_res/1481082404607-2016-12-6-8-46-44-116748/1481082405102-469-700.355536-Neutral.htm) |
| 543 | 1070.30 | 299.09585 | 301.10258 | 130.07506 | 2.00673 | 22216 | 2 | 2 | [18](http://www.mycompoundid.org/mycompoundid_IsoMS/myid_search_res/1481082404607-2016-12-6-8-46-44-116748/1481082405103-541-130.0750583-Neutral.htm) |
| 544 | 1070.56 | 339.09654 | 341.10372 | 210.07643 | 2.00718 | 11269 | 2 | 2 | [16](http://www.mycompoundid.org/mycompoundid_IsoMS/myid_search_res/1481082404607-2016-12-6-8-46-44-116748/1481082405104-840-210.0764318-Neutral.htm) |
| 545 | 1077.06 | 455.16362 | 457.16953 | 221.10530 | 2.00591 | 26306 | 1 | 1 | [16](http://www.mycompoundid.org/mycompoundid_IsoMS/myid_search_res/1481082404607-2016-12-6-8-46-44-116748/1481082405105-87-221.1052959-Neutral.htm) |
| 546 | 1077.31 | 347.08938 | 349.09607 | 226.06212 | 2.00670 | 667375 | 2 | 2 | [11](http://www.mycompoundid.org/mycompoundid_IsoMS/myid_search_res/1481082404607-2016-12-6-8-46-44-116748/1481082405105-877-226.0621154-Neutral.htm) |
| 547 | 1078.28 | 356.09410 | 358.10140 | 244.07156 | 2.00729 | 28069 | 2 | 2 | [32](http://www.mycompoundid.org/mycompoundid_IsoMS/myid_search_res/1481082404607-2016-12-6-8-46-44-116748/1481082405106-478-244.071564-Neutral.htm) |
| 548 | 1083.48 | 348.11498 | 350.11959 | 228.11331 | 2.00461 | 7400 | 2 | 2 | [2](http://www.mycompoundid.org/mycompoundid_IsoMS/myid_search_res/1481082404607-2016-12-6-8-46-44-116748/1481082405107-786-228.11331-Neutral.htm) |
| # | RT (s) | mz_light | mz_heavy | mz | distance | int_light | nCharge | nTag | Possible hits |
| 549 | 1086.58 | 382.10946 | 384.11536 | 296.10227 | 2.00590 | 7533 | 2 | 2 | [18](http://www.mycompoundid.org/mycompoundid_IsoMS/myid_search_res/1481082404607-2016-12-6-8-46-44-116748/1481082405107-295-296.102271-Neutral.htm) |
| 550 | 1091.08 | 429.15197 | 431.15815 | 195.09365 | 2.00618 | 17263 | 1 | 1 | [1](http://www.mycompoundid.org/mycompoundid_IsoMS/myid_search_res/1481082404607-2016-12-6-8-46-44-116748/1481082405108-102-195.093651-Neutral.htm) |
| 551 | 1093.29 | 411.14110 | 413.14666 | 177.08278 | 2.00556 | 130438 | 1 | 1 | [6](http://www.mycompoundid.org/mycompoundid_IsoMS/myid_search_res/1481082404607-2016-12-6-8-46-44-116748/1481082405109-603-177.0827763-Neutral.htm) |
| 552 | 1093.63 | 389.11939 | 391.12549 | 310.12215 | 2.00609 | 6790 | 2 | 2 | [6](http://www.mycompoundid.org/mycompoundid_IsoMS/myid_search_res/1481082404607-2016-12-6-8-46-44-116748/1481082405109-460-310.122148-Neutral.htm) |
| 553 | 1094.23 | 372.17318 | 374.18009 | 138.11486 | 2.00691 | 11744 | 1 | 1 | [3](http://www.mycompoundid.org/mycompoundid_IsoMS/myid_search_res/1481082404607-2016-12-6-8-46-44-116748/1481082405110-539-138.1148589-Neutral.htm) |
| 554 | 1096.58 | 341.64071 | 343.64715 | 215.16477 | 2.00644 | 17750 | 2 | 2 | [5](http://www.mycompoundid.org/mycompoundid_IsoMS/myid_search_res/1481082404607-2016-12-6-8-46-44-116748/1481082405110-43-215.1647707-Neutral.htm) |
| 555 | 1097.31 | 487.15238 | 489.15885 | 253.09406 | 2.00647 | 16150 | 1 | 1 | [9](http://www.mycompoundid.org/mycompoundid_IsoMS/myid_search_res/1481082404607-2016-12-6-8-46-44-116748/1481082405111-748-253.0940568-Neutral.htm) |
| 556 | 1097.57 | 761.31529 | 763.32157 | 527.25697 | 2.00629 | 5790 | 1 | 1 | [12](http://www.mycompoundid.org/mycompoundid_IsoMS/myid_search_res/1481082404607-2016-12-6-8-46-44-116748/1481082405112-112-527.256969-Neutral.htm) |
| 557 | 1098.63 | 759.30885 | 761.31309 | 525.25053 | 2.00424 | 21400 | 1 | 1 | [7](http://www.mycompoundid.org/mycompoundid_IsoMS/myid_search_res/1481082404607-2016-12-6-8-46-44-116748/1481082405112-230-525.250531-Neutral.htm) |
| 558 | 1098.67 | 541.14297 | 543.14710 | 307.08465 | 2.00413 | 9710 | 1 | 1 | [14](http://www.mycompoundid.org/mycompoundid_IsoMS/myid_search_res/1481082404607-2016-12-6-8-46-44-116748/1481082405113-880-307.0846515-Neutral.htm) |
| 559 | 1100.11 | 356.09358 | 358.10081 | 244.07053 | 2.00723 | 40294 | 2 | 2 | [29](http://www.mycompoundid.org/mycompoundid_IsoMS/myid_search_res/1481082404607-2016-12-6-8-46-44-116748/1481082405113-326-244.070525-Neutral.htm) |
| 560 | 1100.38 | 298.08804 | 300.09521 | 128.05945 | 2.00717 | 538375 | 2 | 2 | [16](http://www.mycompoundid.org/mycompoundid_IsoMS/myid_search_res/1481082404607-2016-12-6-8-46-44-116748/1481082405114-58-128.0594455-Neutral.htm) |
| 561 | 1104.59 | 335.10835 | 337.11448 | 202.10007 | 2.00612 | 16400 | 2 | 2 | [1](http://www.mycompoundid.org/mycompoundid_IsoMS/myid_search_res/1481082404607-2016-12-6-8-46-44-116748/1481082405115-195-202.1000685-Neutral.htm) |
| 562 | 1107.66 | 421.17993 | 423.18596 | 187.12161 | 2.00603 | 299000 | 1 | 1 | [22](http://www.mycompoundid.org/mycompoundid_IsoMS/myid_search_res/1481082404607-2016-12-6-8-46-44-116748/1481082405117-298-187.1216088-Neutral.htm) |
| 563 | 1109.55 | 466.17945 | 468.18461 | 232.12113 | 2.00516 | 11031 | 1 | 1 | [6](http://www.mycompoundid.org/mycompoundid_IsoMS/myid_search_res/1481082404607-2016-12-6-8-46-44-116748/1481082405117-617-232.1211274-Neutral.htm) |
| 564 | 1110.73 | 356.09422 | 358.10090 | 244.07180 | 2.00668 | 248125 | 2 | 2 | [32](http://www.mycompoundid.org/mycompoundid_IsoMS/myid_search_res/1481082404607-2016-12-6-8-46-44-116748/1481082405118-930-244.0717966-Neutral.htm) |
| 565 | 1117.69 | 328.13365 | 330.14152 | 188.15066 | 2.00786 | 9650 | 2 | 2 | [4](http://www.mycompoundid.org/mycompoundid_IsoMS/myid_search_res/1481082404607-2016-12-6-8-46-44-116748/1481082405119-370-188.150663-Neutral.htm) |
| 566 | 1124.66 | 423.14240 | 425.14653 | 189.08408 | 2.00414 | 8380 | 1 | 1 | [1](http://www.mycompoundid.org/mycompoundid_IsoMS/myid_search_res/1481082404607-2016-12-6-8-46-44-116748/1481082405120-9-189.084075-Neutral.htm) |
| 567 | 1128.03 | 326.63526 | 328.64112 | 185.15387 | 2.00586 | 13700 | 2 | 2 | [2](http://www.mycompoundid.org/mycompoundid_IsoMS/myid_search_res/1481082404607-2016-12-6-8-46-44-116748/1481082405121-77-185.153874-Neutral.htm) |
| 568 | 1129.61 | 321.16280 | 323.16964 | 87.10448 | 2.00684 | 148638 | 1 | 1 | [10](http://www.mycompoundid.org/mycompoundid_IsoMS/myid_search_res/1481082404607-2016-12-6-8-46-44-116748/1481082405121-109-87.10447856-Neutral.htm) |
| 569 | 1130.88 | 348.11413 | 350.12264 | 228.11163 | 2.00850 | 32100 | 2 | 2 | [8](http://www.mycompoundid.org/mycompoundid_IsoMS/myid_search_res/1481082404607-2016-12-6-8-46-44-116748/1481082405122-582-228.111628-Neutral.htm) |
| 570 | 1134.33 | 328.11714 | 330.12356 | 188.11764 | 2.00643 | 57016 | 2 | 2 | [24](http://www.mycompoundid.org/mycompoundid_IsoMS/myid_search_res/1481082404607-2016-12-6-8-46-44-116748/1481082405122-372-188.1176385-Neutral.htm) |
| 571 | 1140.53 | 386.10673 | 388.11380 | 152.04841 | 2.00707 | 8720 | 1 | 1 | [31](http://www.mycompoundid.org/mycompoundid_IsoMS/myid_search_res/1481082404607-2016-12-6-8-46-44-116748/1481082405124-391-152.04841-Neutral.htm) |
| 572 | 1143.63 | 528.16246 | 532.17549 | 61.05312 | 4.01303 | 52644 | 1 | 2 | [15](http://www.mycompoundid.org/mycompoundid_IsoMS/myid_search_res/1481082404607-2016-12-6-8-46-44-116748/1481082405125-282-61.05311956-Neutral.htm) |
| # | RT (s) | mz_light | mz_heavy | mz | distance | int_light | nCharge | nTag | Possible hits |
| 573 | 1145.62 | 321.10926 | 323.11535 | 174.10188 | 2.00609 | 35200 | 2 | 2 | [35](http://www.mycompoundid.org/mycompoundid_IsoMS/myid_search_res/1481082404607-2016-12-6-8-46-44-116748/1481082405126-384-174.101876-Neutral.htm) |
| 574 | 1146.24 | 532.24870 | 534.25537 | 298.19038 | 2.00667 | 15350 | 1 | 1 | [18](http://www.mycompoundid.org/mycompoundid_IsoMS/myid_search_res/1481082404607-2016-12-6-8-46-44-116748/1481082405127-699-298.1903806-Neutral.htm) |
| 575 | 1148.74 | 349.15752 | 351.16314 | 115.09920 | 2.00562 | 10925 | 1 | 1 | [21](http://www.mycompoundid.org/mycompoundid_IsoMS/myid_search_res/1481082404607-2016-12-6-8-46-44-116748/1481082405128-5-115.0992025-Neutral.htm) |
| 576 | 1150.70 | 336.11439 | 338.12125 | 204.11214 | 2.00686 | 7110 | 2 | 2 | [11](http://www.mycompoundid.org/mycompoundid_IsoMS/myid_search_res/1481082404607-2016-12-6-8-46-44-116748/1481082405128-567-204.112144-Neutral.htm) |
| 577 | 1153.10 | 292.10594 | 294.11229 | 116.09524 | 2.00635 | 41381 | 2 | 2 | [9](http://www.mycompoundid.org/mycompoundid_IsoMS/myid_search_res/1481082404607-2016-12-6-8-46-44-116748/1481082405129-783-116.0952381-Neutral.htm) |
| 578 | 1153.63 | 379.16944 | 381.17540 | 145.11112 | 2.00596 | 379156 | 1 | 1 | [19](http://www.mycompoundid.org/mycompoundid_IsoMS/myid_search_res/1481082404607-2016-12-6-8-46-44-116748/1481082405130-297-145.1111201-Neutral.htm) |
| 579 | 1156.45 | 487.09989 | 489.10508 | 253.04157 | 2.00519 | 20350 | 1 | 1 | [3](http://www.mycompoundid.org/mycompoundid_IsoMS/myid_search_res/1481082404607-2016-12-6-8-46-44-116748/1481082405131-831-253.0415735-Neutral.htm) |
| 580 | 1161.51 | 368.10170 | 370.10507 | 268.08675 | 2.00337 | 83175 | 2 | 2 | [9](http://www.mycompoundid.org/mycompoundid_IsoMS/myid_search_res/1481082404607-2016-12-6-8-46-44-116748/1481082405132-825-268.08675-Neutral.htm) |
| 581 | 1161.60 | 353.09554 | 355.10164 | 119.03722 | 2.00610 | 35100 | 1 | 1 | [7](http://www.mycompoundid.org/mycompoundid_IsoMS/myid_search_res/1481082404607-2016-12-6-8-46-44-116748/1481082405132-551-119.037223-Neutral.htm) |
| 582 | 1176.41 | 335.64035 | 337.64751 | 203.16406 | 2.00716 | 56400 | 2 | 2 | [4](http://www.mycompoundid.org/mycompoundid_IsoMS/myid_search_res/1481082404607-2016-12-6-8-46-44-116748/1481082405133-26-203.1640603-Neutral.htm) |
| 583 | 1176.71 | 338.08845 | 340.09375 | 104.03013 | 2.00530 | 9610 | 1 | 1 | [3](http://www.mycompoundid.org/mycompoundid_IsoMS/myid_search_res/1481082404607-2016-12-6-8-46-44-116748/1481082405133-102-104.030127-Neutral.htm) |
| 584 | 1183.58 | 693.16709 | 697.18175 | 226.05775 | 4.01466 | 12700 | 1 | 2 | [18](http://www.mycompoundid.org/mycompoundid_IsoMS/myid_search_res/1481082404607-2016-12-6-8-46-44-116748/1481082405134-327-226.057751-Neutral.htm) |
| 585 | 1187.80 | 346.08548 | 348.09120 | 112.02716 | 2.00572 | 32000 | 1 | 1 | [9](http://www.mycompoundid.org/mycompoundid_IsoMS/myid_search_res/1481082404607-2016-12-6-8-46-44-116748/1481082405135-407-112.0271575-Neutral.htm) |
| 586 | 1190.73 | 711.13194 | 715.14952 | 244.02260 | 4.01758 | 10500 | 1 | 2 | [2](http://www.mycompoundid.org/mycompoundid_IsoMS/myid_search_res/1481082404607-2016-12-6-8-46-44-116748/1481082405136-959-244.022597-Neutral.htm) |
| 587 | 1190.80 | 311.08533 | 313.09345 | 154.05402 | 2.00812 | 1102188 | 2 | 2 | [1](http://www.mycompoundid.org/mycompoundid_IsoMS/myid_search_res/1481082404607-2016-12-6-8-46-44-116748/1481082405136-537-154.054023-Neutral.htm) |
| 588 | 1196.40 | 427.16945 | 429.17523 | 193.11113 | 2.00578 | 371219 | 1 | 1 | [13](http://www.mycompoundid.org/mycompoundid_IsoMS/myid_search_res/1481082404607-2016-12-6-8-46-44-116748/1481082405137-174-193.1111291-Neutral.htm) |
| 589 | 1197.20 | 472.22522 | 474.23277 | 238.16690 | 2.00755 | 7365 | 1 | 1 | [1](http://www.mycompoundid.org/mycompoundid_IsoMS/myid_search_res/1481082404607-2016-12-6-8-46-44-116748/1481082405137-755-238.166899-Neutral.htm) |
| 590 | 1197.95 | 343.08621 | 345.09177 | 109.02789 | 2.00556 | 12800 | 1 | 1 | [3](http://www.mycompoundid.org/mycompoundid_IsoMS/myid_search_res/1481082404607-2016-12-6-8-46-44-116748/1481082405138-881-109.027888-Neutral.htm) |
| 591 | 1198.60 | 359.10529 | 361.11103 | 125.04697 | 2.00575 | 39700 | 1 | 1 | [15](http://www.mycompoundid.org/mycompoundid_IsoMS/myid_search_res/1481082404607-2016-12-6-8-46-44-116748/1481082405138-767-125.046965-Neutral.htm) |
| 592 | 1199.57 | 335.16715 | 337.17351 | 202.21767 | 2.00635 | 20450 | 2 | 2 | [1](http://www.mycompoundid.org/mycompoundid_IsoMS/myid_search_res/1481082404607-2016-12-6-8-46-44-116748/1481082405139-330-202.2176685-Neutral.htm) |
| 593 | 1202.35 | 327.09071 | 329.09775 | 186.06478 | 2.00705 | 36100 | 2 | 2 | [16](http://www.mycompoundid.org/mycompoundid_IsoMS/myid_search_res/1481082404607-2016-12-6-8-46-44-116748/1481082405139-370-186.064776-Neutral.htm) |
| 594 | 1203.83 | 397.15846 | 399.16358 | 163.10014 | 2.00512 | 11875 | 1 | 1 | [16](http://www.mycompoundid.org/mycompoundid_IsoMS/myid_search_res/1481082404607-2016-12-6-8-46-44-116748/1481082405140-236-163.1001353-Neutral.htm) |
| 595 | 1204.07 | 355.06430 | 357.07099 | 242.01196 | 2.00668 | 12953 | 2 | 2 | [7](http://www.mycompoundid.org/mycompoundid_IsoMS/myid_search_res/1481082404607-2016-12-6-8-46-44-116748/1481082405141-207-242.0119645-Neutral.htm) |
| 596 | 1215.62 | 403.14390 | 405.15022 | 169.08558 | 2.00633 | 11850 | 1 | 1 | [11](http://www.mycompoundid.org/mycompoundid_IsoMS/myid_search_res/1481082404607-2016-12-6-8-46-44-116748/1481082405141-436-169.085575-Neutral.htm) |
| # | RT (s) | mz_light | mz_heavy | mz | distance | int_light | nCharge | nTag | Possible hits |
| 597 | 1219.76 | 355.06379 | 357.07005 | 242.01094 | 2.00626 | 8680 | 2 | 2 | [7](http://www.mycompoundid.org/mycompoundid_IsoMS/myid_search_res/1481082404607-2016-12-6-8-46-44-116748/1481082405142-48-242.010938-Neutral.htm) |
| 598 | 1225.89 | 325.09802 | 327.10475 | 182.07941 | 2.00673 | 661500 | 2 | 2 | [35](http://www.mycompoundid.org/mycompoundid_IsoMS/myid_search_res/1481082404607-2016-12-6-8-46-44-116748/1481082405144-307-182.079408-Neutral.htm) |
| 599 | 1226.60 | 324.59677 | 326.60342 | 181.07691 | 2.00665 | 5635547 | 2 | 2 | [4](http://www.mycompoundid.org/mycompoundid_IsoMS/myid_search_res/1481082404607-2016-12-6-8-46-44-116748/1481082405146-486-181.0769078-Neutral.htm) |
| 600 | 1227.31 | 414.12389 | 416.13048 | 180.06557 | 2.00658 | 35853 | 1 | 1 | [10](http://www.mycompoundid.org/mycompoundid_IsoMS/myid_search_res/1481082404607-2016-12-6-8-46-44-116748/1481082405147-190-180.0655725-Neutral.htm) |
| 601 | 1229.02 | 373.08570 | 375.09071 | 139.02738 | 2.00501 | 15050 | 1 | 1 | [9](http://www.mycompoundid.org/mycompoundid_IsoMS/myid_search_res/1481082404607-2016-12-6-8-46-44-116748/1481082405148-840-139.027379-Neutral.htm) |
| 602 | 1229.78 | 386.10619 | 388.11146 | 152.04787 | 2.00527 | 26500 | 1 | 1 | [31](http://www.mycompoundid.org/mycompoundid_IsoMS/myid_search_res/1481082404607-2016-12-6-8-46-44-116748/1481082405148-103-152.047867-Neutral.htm) |
| 603 | 1237.79 | 363.17355 | 365.17966 | 129.11523 | 2.00611 | 30328 | 1 | 1 | [10](http://www.mycompoundid.org/mycompoundid_IsoMS/myid_search_res/1481082404607-2016-12-6-8-46-44-116748/1481082405150-719-129.1152295-Neutral.htm) |
| 604 | 1238.67 | 335.17841 | 337.18567 | 101.12009 | 2.00726 | 444575 | 1 | 1 | [1](http://www.mycompoundid.org/mycompoundid_IsoMS/myid_search_res/1481082404607-2016-12-6-8-46-44-116748/1481082405150-394-101.1200904-Neutral.htm) |
| 605 | 1239.81 | 515.21134 | 517.21741 | 562.30605 | 2.00607 | 9200 | 2 | 2 | [1](http://www.mycompoundid.org/mycompoundid_IsoMS/myid_search_res/1481082404607-2016-12-6-8-46-44-116748/1481082405150-435-562.306047-Neutral.htm) |
| 606 | 1240.64 | 800.38542 | 802.39352 | 566.32710 | 2.00810 | 15000 | 1 | 1 | [4](http://www.mycompoundid.org/mycompoundid_IsoMS/myid_search_res/1481082404607-2016-12-6-8-46-44-116748/1481082405151-73-566.327102-Neutral.htm) |
| 607 | 1243.70 | 378.08280 | 380.08899 | 144.02448 | 2.00619 | 27225 | 1 | 1 | [1](http://www.mycompoundid.org/mycompoundid_IsoMS/myid_search_res/1481082404607-2016-12-6-8-46-44-116748/1481082405152-862-144.0244789-Neutral.htm) |
| 608 | 1249.23 | 363.17292 | 365.17969 | 129.11460 | 2.00677 | 13150 | 1 | 1 | [10](http://www.mycompoundid.org/mycompoundid_IsoMS/myid_search_res/1481082404607-2016-12-6-8-46-44-116748/1481082405152-410-129.1145965-Neutral.htm) |
| 609 | 1252.74 | 335.17933 | 337.18635 | 101.12101 | 2.00702 | 576656 | 1 | 1 | [1](http://www.mycompoundid.org/mycompoundid_IsoMS/myid_search_res/1481082404607-2016-12-6-8-46-44-116748/1481082405153-986-101.1210075-Neutral.htm) |
| 610 | 1263.07 | 437.17342 | 439.18081 | 203.11510 | 2.00739 | 30275 | 1 | 1 | [22](http://www.mycompoundid.org/mycompoundid_IsoMS/myid_search_res/1481082404607-2016-12-6-8-46-44-116748/1481082405154-321-203.1151035-Neutral.htm) |
| 611 | 1263.47 | 335.17842 | 337.18496 | 101.12010 | 2.00653 | 57878 | 1 | 1 | [1](http://www.mycompoundid.org/mycompoundid_IsoMS/myid_search_res/1481082404607-2016-12-6-8-46-44-116748/1481082405155-461-101.1201012-Neutral.htm) |
| 612 | 1269.01 | 373.12257 | 375.12872 | 278.12850 | 2.00615 | 17950 | 2 | 2 | [17](http://www.mycompoundid.org/mycompoundid_IsoMS/myid_search_res/1481082404607-2016-12-6-8-46-44-116748/1481082405156-911-278.1285035-Neutral.htm) |
| 613 | 1278.93 | 681.29384 | 683.29907 | 447.23552 | 2.00523 | 16313 | 1 | 1 | [6](http://www.mycompoundid.org/mycompoundid_IsoMS/myid_search_res/1481082404607-2016-12-6-8-46-44-116748/1481082405156-249-447.2355168-Neutral.htm) |
| 614 | 1280.03 | 345.62470 | 347.63089 | 223.13277 | 2.00619 | 11600 | 2 | 2 | [2](http://www.mycompoundid.org/mycompoundid_IsoMS/myid_search_res/1481082404607-2016-12-6-8-46-44-116748/1481082405157-203-223.132766-Neutral.htm) |
| 615 | 1281.84 | 690.79093 | 692.79794 | 913.46522 | 2.00701 | 26420 | 2 | 2 | [1](http://www.mycompoundid.org/mycompoundid_IsoMS/myid_search_res/1481082404607-2016-12-6-8-46-44-116748/1481082405157-226-913.465215-Neutral.htm) |
| 616 | 1282.81 | 275.08522 | 277.09202 | 82.05380 | 2.00680 | 16538 | 2 | 2 | [4](http://www.mycompoundid.org/mycompoundid_IsoMS/myid_search_res/1481082404607-2016-12-6-8-46-44-116748/1481082405158-587-82.05379738-Neutral.htm) |
| 617 | 1282.90 | 690.45901 | 692.46494 | 456.40069 | 2.00593 | 30138 | 1 | 1 | [12](http://www.mycompoundid.org/mycompoundid_IsoMS/myid_search_res/1481082404607-2016-12-6-8-46-44-116748/1481082405158-138-456.4006903-Neutral.htm) |
| 618 | 1284.23 | 598.10105 | 600.10475 | 364.04273 | 2.00370 | 11340 | 1 | 1 | [6](http://www.mycompoundid.org/mycompoundid_IsoMS/myid_search_res/1481082404607-2016-12-6-8-46-44-116748/1481082405158-514-364.0427295-Neutral.htm) |
| 619 | 1286.04 | 331.56011 | 333.56734 | 195.00358 | 2.00723 | 10300 | 2 | 2 | [3](http://www.mycompoundid.org/mycompoundid_IsoMS/myid_search_res/1481082404607-2016-12-6-8-46-44-116748/1481082405159-702-195.003582-Neutral.htm) |
| 620 | 1289.10 | 327.10907 | 329.11622 | 186.10150 | 2.00715 | 19775 | 2 | 2 | [15](http://www.mycompoundid.org/mycompoundid_IsoMS/myid_search_res/1481082404607-2016-12-6-8-46-44-116748/1481082405160-256-186.1015035-Neutral.htm) |
| # | RT (s) | mz_light | mz_heavy | mz | distance | int_light | nCharge | nTag | Possible hits |
| 621 | 1294.28 | 321.12757 | 323.13420 | 174.13850 | 2.00663 | 143875 | 2 | 2 | [9](http://www.mycompoundid.org/mycompoundid_IsoMS/myid_search_res/1481082404607-2016-12-6-8-46-44-116748/1481082405160-889-174.1385036-Neutral.htm) |
| 622 | 1298.78 | 302.07811 | 304.08615 | 136.03958 | 2.00803 | 6970 | 2 | 2 | [10](http://www.mycompoundid.org/mycompoundid_IsoMS/myid_search_res/1481082404607-2016-12-6-8-46-44-116748/1481082405161-444-136.039582-Neutral.htm) |
| 623 | 1302.79 | 356.59542 | 358.60206 | 245.07421 | 2.00664 | 159528 | 2 | 2 | [6](http://www.mycompoundid.org/mycompoundid_IsoMS/myid_search_res/1481082404607-2016-12-6-8-46-44-116748/1481082405162-417-245.0742089-Neutral.htm) |
| 624 | 1303.75 | 357.59491 | 359.60199 | 247.07317 | 2.00708 | 17300 | 2 | 2 | [1](http://www.mycompoundid.org/mycompoundid_IsoMS/myid_search_res/1481082404607-2016-12-6-8-46-44-116748/1481082405162-920-247.073172-Neutral.htm) |
| 625 | 1306.20 | 435.19433 | 437.20016 | 201.13601 | 2.00583 | 23763 | 1 | 1 | [12](http://www.mycompoundid.org/mycompoundid_IsoMS/myid_search_res/1481082404607-2016-12-6-8-46-44-116748/1481082405163-322-201.1360106-Neutral.htm) |
| 626 | 1308.32 | 354.11613 | 356.12235 | 120.05781 | 2.00622 | 190583 | 1 | 1 | [13](http://www.mycompoundid.org/mycompoundid_IsoMS/myid_search_res/1481082404607-2016-12-6-8-46-44-116748/1481082405163-515-120.0578089-Neutral.htm) |
| 627 | 1310.36 | 331.60383 | 333.61060 | 195.09102 | 2.00677 | 50994 | 2 | 2 | [30](http://www.mycompoundid.org/mycompoundid_IsoMS/myid_search_res/1481082404607-2016-12-6-8-46-44-116748/1481082405164-992-195.0910173-Neutral.htm) |
| 628 | 1310.46 | 367.14712 | 369.15390 | 133.08880 | 2.00678 | 14675 | 1 | 1 | [6](http://www.mycompoundid.org/mycompoundid_IsoMS/myid_search_res/1481082404607-2016-12-6-8-46-44-116748/1481082405165-727-133.0887988-Neutral.htm) |
| 629 | 1311.31 | 488.15178 | 490.15874 | 254.09346 | 2.00696 | 21775 | 1 | 1 | [8](http://www.mycompoundid.org/mycompoundid_IsoMS/myid_search_res/1481082404607-2016-12-6-8-46-44-116748/1481082405166-340-254.0934591-Neutral.htm) |
| 630 | 1313.29 | 325.60921 | 327.61556 | 183.10179 | 2.00634 | 26088 | 2 | 2 | [8](http://www.mycompoundid.org/mycompoundid_IsoMS/myid_search_res/1481082404607-2016-12-6-8-46-44-116748/1481082405166-666-183.101788-Neutral.htm) |
| 631 | 1314.07 | 284.60082 | 286.60668 | 101.08500 | 2.00586 | 10300 | 2 | 2 | [20](http://www.mycompoundid.org/mycompoundid_IsoMS/myid_search_res/1481082404607-2016-12-6-8-46-44-116748/1481082405167-910-101.085004-Neutral.htm) |
| 632 | 1315.72 | 973.28293 | 975.28353 | 739.22461 | 2.00060 | 10300 | 1 | 1 | [1](http://www.mycompoundid.org/mycompoundid_IsoMS/myid_search_res/1481082404607-2016-12-6-8-46-44-116748/1481082405168-315-739.224606-Neutral.htm) |
| 633 | 1320.01 | 354.11670 | 356.12358 | 120.05838 | 2.00689 | 821875 | 1 | 1 | [13](http://www.mycompoundid.org/mycompoundid_IsoMS/myid_search_res/1481082404607-2016-12-6-8-46-44-116748/1481082405168-9-120.0583782-Neutral.htm) |
| 634 | 1325.81 | 338.59329 | 340.60018 | 209.06993 | 2.00689 | 24400 | 2 | 2 | [42](http://www.mycompoundid.org/mycompoundid_IsoMS/myid_search_res/1481082404607-2016-12-6-8-46-44-116748/1481082405169-489-209.069934-Neutral.htm) |
| 635 | 1331.07 | 345.60138 | 347.60789 | 223.08612 | 2.00652 | 34638 | 2 | 2 | [30](http://www.mycompoundid.org/mycompoundid_IsoMS/myid_search_res/1481082404607-2016-12-6-8-46-44-116748/1481082405170-238-223.0861163-Neutral.htm) |
| 636 | 1332.36 | 527.25500 | 529.26273 | 293.19668 | 2.00772 | 22566 | 1 | 1 | [7](http://www.mycompoundid.org/mycompoundid_IsoMS/myid_search_res/1481082404607-2016-12-6-8-46-44-116748/1481082405172-606-293.1966845-Neutral.htm) |
| 637 | 1333.22 | 515.13009 | 519.14304 | 48.02075 | 4.01294 | 10443 | 1 | 2 | [18](http://www.mycompoundid.org/mycompoundid_IsoMS/myid_search_res/1481082404607-2016-12-6-8-46-44-116748/1481082405172-841-48.02075175-Neutral.htm) |
| 638 | 1335.09 | 573.24449 | 575.25077 | 339.18617 | 2.00628 | 10400 | 1 | 1 | [1](http://www.mycompoundid.org/mycompoundid_IsoMS/myid_search_res/1481082404607-2016-12-6-8-46-44-116748/1481082405173-156-339.186173-Neutral.htm) |
| 639 | 1336.96 | 349.19354 | 351.19983 | 115.13522 | 2.00630 | 22750 | 1 | 1 | [1](http://www.mycompoundid.org/mycompoundid_IsoMS/myid_search_res/1481082404607-2016-12-6-8-46-44-116748/1481082405173-794-115.135216-Neutral.htm) |
| 640 | 1337.59 | 630.93376 | 632.93945 | 793.75088 | 2.00569 | 9965 | 2 | 2 | [1](http://www.mycompoundid.org/mycompoundid_IsoMS/myid_search_res/1481082404607-2016-12-6-8-46-44-116748/1481082405173-20-793.750884-Neutral.htm) |
| 641 | 1343.16 | 631.09962 | 633.10616 | 794.08261 | 2.00654 | 30003 | 2 | 2 | [1](http://www.mycompoundid.org/mycompoundid_IsoMS/myid_search_res/1481082404607-2016-12-6-8-46-44-116748/1481082405174-421-794.0826065-Neutral.htm) |
| 642 | 1346.90 | 353.07408 | 355.08062 | 238.03152 | 2.00655 | 12300 | 2 | 2 | [11](http://www.mycompoundid.org/mycompoundid_IsoMS/myid_search_res/1481082404607-2016-12-6-8-46-44-116748/1481082405175-657-238.031516-Neutral.htm) |
| 643 | 1349.36 | 630.93330 | 632.93979 | 793.74996 | 2.00649 | 58481 | 2 | 2 | [1](http://www.mycompoundid.org/mycompoundid_IsoMS/myid_search_res/1481082404607-2016-12-6-8-46-44-116748/1481082405176-827-793.7499558-Neutral.htm) |
| 644 | 1350.47 | 331.60387 | 333.61080 | 195.09110 | 2.00693 | 344250 | 2 | 2 | [30](http://www.mycompoundid.org/mycompoundid_IsoMS/myid_search_res/1481082404607-2016-12-6-8-46-44-116748/1481082405176-735-195.0911003-Neutral.htm) |
| # | RT (s) | mz_light | mz_heavy | mz | distance | int_light | nCharge | nTag | Possible hits |
| 645 | 1352.47 | 297.59040 | 299.59735 | 127.06417 | 2.00694 | 57313 | 2 | 2 | [20](http://www.mycompoundid.org/mycompoundid_IsoMS/myid_search_res/1481082404607-2016-12-6-8-46-44-116748/1481082405177-429-127.0641674-Neutral.htm) |
| 646 | 1353.41 | 577.15698 | 581.16941 | 110.04764 | 4.01243 | 21538 | 1 | 2 | [8](http://www.mycompoundid.org/mycompoundid_IsoMS/myid_search_res/1481082404607-2016-12-6-8-46-44-116748/1481082405179-374-110.0476404-Neutral.htm) |
| 647 | 1357.84 | 886.24530 | 888.25468 | 1304.37397 | 2.00938 | 7980 | 2 | 2 | [2](http://www.mycompoundid.org/mycompoundid_IsoMS/myid_search_res/1481082404607-2016-12-6-8-46-44-116748/1481082405180-803-1304.373966-Neutral.htm) |
| 648 | 1358.13 | 352.60937 | 354.61549 | 237.10210 | 2.00612 | 28288 | 2 | 2 | [11](http://www.mycompoundid.org/mycompoundid_IsoMS/myid_search_res/1481082404607-2016-12-6-8-46-44-116748/1481082405180-872-237.1020961-Neutral.htm) |
| 649 | 1359.12 | 994.27031 | 996.27870 | 760.21199 | 2.00839 | 11500 | 1 | 1 | [1](http://www.mycompoundid.org/mycompoundid_IsoMS/myid_search_res/1481082404607-2016-12-6-8-46-44-116748/1481082405181-476-760.211985-Neutral.htm) |
| 650 | 1361.39 | 588.26278 | 590.26994 | 354.20446 | 2.00716 | 14938 | 1 | 1 | [10](http://www.mycompoundid.org/mycompoundid_IsoMS/myid_search_res/1481082404607-2016-12-6-8-46-44-116748/1481082405181-751-354.2044581-Neutral.htm) |
| 651 | 1361.52 | 287.08075 | 289.08682 | 106.04486 | 2.00607 | 7935 | 2 | 2 | [1](http://www.mycompoundid.org/mycompoundid_IsoMS/myid_search_res/1481082404607-2016-12-6-8-46-44-116748/1481082405182-80-106.0448553-Neutral.htm) |
| 652 | 1362.27 | 308.58151 | 310.58939 | 149.04638 | 2.00788 | 29494 | 2 | 2 | [17](http://www.mycompoundid.org/mycompoundid_IsoMS/myid_search_res/1481082404607-2016-12-6-8-46-44-116748/1481082405182-350-149.0463824-Neutral.htm) |
| 653 | 1363.36 | 313.59304 | 315.59988 | 159.06944 | 2.00684 | 37797 | 2 | 2 | [10](http://www.mycompoundid.org/mycompoundid_IsoMS/myid_search_res/1481082404607-2016-12-6-8-46-44-116748/1481082405183-827-159.0694381-Neutral.htm) |
| 654 | 1364.45 | 305.57144 | 307.57810 | 143.02623 | 2.00666 | 152469 | 2 | 2 | [2](http://www.mycompoundid.org/mycompoundid_IsoMS/myid_search_res/1481082404607-2016-12-6-8-46-44-116748/1481082405183-71-143.0262309-Neutral.htm) |
| 655 | 1367.48 | 577.15696 | 581.17035 | 110.04762 | 4.01339 | 27553 | 1 | 2 | [8](http://www.mycompoundid.org/mycompoundid_IsoMS/myid_search_res/1481082404607-2016-12-6-8-46-44-116748/1481082405184-59-110.0476247-Neutral.htm) |
| 656 | 1375.96 | 344.10595 | 346.11224 | 110.04763 | 2.00629 | 33750 | 1 | 1 | [8](http://www.mycompoundid.org/mycompoundid_IsoMS/myid_search_res/1481082404607-2016-12-6-8-46-44-116748/1481082405185-907-110.0476275-Neutral.htm) |
| 657 | 1378.47 | 328.13459 | 330.14174 | 188.15255 | 2.00715 | 9450 | 2 | 2 | [4](http://www.mycompoundid.org/mycompoundid_IsoMS/myid_search_res/1481082404607-2016-12-6-8-46-44-116748/1481082405185-151-188.1525475-Neutral.htm) |
| 658 | 1379.15 | 504.18334 | 506.18996 | 270.12502 | 2.00662 | 19950 | 1 | 1 | [11](http://www.mycompoundid.org/mycompoundid_IsoMS/myid_search_res/1481082404607-2016-12-6-8-46-44-116748/1481082405186-350-270.125016-Neutral.htm) |
| 659 | 1385.41 | 302.07785 | 304.08488 | 136.03906 | 2.00703 | 36913 | 2 | 2 | [10](http://www.mycompoundid.org/mycompoundid_IsoMS/myid_search_res/1481082404607-2016-12-6-8-46-44-116748/1481082405187-843-136.0390614-Neutral.htm) |
| 660 | 1388.40 | 772.32794 | 774.33575 | 538.26962 | 2.00781 | 28100 | 1 | 1 | [7](http://www.mycompoundid.org/mycompoundid_IsoMS/myid_search_res/1481082404607-2016-12-6-8-46-44-116748/1481082405188-804-538.2696225-Neutral.htm) |
| 661 | 1388.47 | 515.13085 | 519.14448 | 48.02151 | 4.01363 | 304875 | 1 | 2 | [18](http://www.mycompoundid.org/mycompoundid_IsoMS/myid_search_res/1481082404607-2016-12-6-8-46-44-116748/1481082405188-217-48.02150756-Neutral.htm) |
| 662 | 1394.29 | 847.32132 | 851.34155 | 380.21198 | 4.02023 | 2096563 | 1 | 2 | [4](http://www.mycompoundid.org/mycompoundid_IsoMS/myid_search_res/1481082404607-2016-12-6-8-46-44-116748/1481082405189-123-380.2119805-Neutral.htm) |
| 663 | 1394.42 | 612.26697 | 616.28060 | 145.15763 | 4.01364 | 55681 | 1 | 2 | [3](http://www.mycompoundid.org/mycompoundid_IsoMS/myid_search_res/1481082404607-2016-12-6-8-46-44-116748/1481082405190-571-145.1576293-Neutral.htm) |
| 664 | 1398.37 | 454.24100 | 456.24746 | 220.18268 | 2.00645 | 222864 | 1 | 1 | [3](http://www.mycompoundid.org/mycompoundid_IsoMS/myid_search_res/1481082404607-2016-12-6-8-46-44-116748/1481082405191-955-220.1826822-Neutral.htm) |
| 665 | 1400.16 | 826.36916 | 828.37345 | 592.31084 | 2.00429 | 7090 | 1 | 1 | [5](http://www.mycompoundid.org/mycompoundid_IsoMS/myid_search_res/1481082404607-2016-12-6-8-46-44-116748/1481082405191-676-592.310844-Neutral.htm) |
| 666 | 1405.36 | 363.21032 | 365.21693 | 129.15200 | 2.00661 | 383521 | 1 | 1 | [1](http://www.mycompoundid.org/mycompoundid_IsoMS/myid_search_res/1481082404607-2016-12-6-8-46-44-116748/1481082405192-500-129.151997-Neutral.htm) |
| 667 | 1408.01 | 331.08998 | 333.09706 | 194.06333 | 2.00708 | 10671 | 2 | 2 | [1](http://www.mycompoundid.org/mycompoundid_IsoMS/myid_search_res/1481082404607-2016-12-6-8-46-44-116748/1481082405192-910-194.0633288-Neutral.htm) |
| 668 | 1412.89 | 620.24786 | 622.25051 | 386.18954 | 2.00265 | 24200 | 1 | 1 | [5](http://www.mycompoundid.org/mycompoundid_IsoMS/myid_search_res/1481082404607-2016-12-6-8-46-44-116748/1481082405192-443-386.1895355-Neutral.htm) |
| # | RT (s) | mz_light | mz_heavy | mz | distance | int_light | nCharge | nTag | Possible hits |
| 669 | 1414.54 | 947.22806 | 949.23212 | 713.16974 | 2.00406 | 11600 | 1 | 1 | [3](http://www.mycompoundid.org/mycompoundid_IsoMS/myid_search_res/1481082404607-2016-12-6-8-46-44-116748/1481082405193-819-713.169742-Neutral.htm) |
| 670 | 1415.41 | 297.08133 | 299.08729 | 126.04601 | 2.00597 | 34594 | 2 | 2 | [3](http://www.mycompoundid.org/mycompoundid_IsoMS/myid_search_res/1481082404607-2016-12-6-8-46-44-116748/1481082405194-151-126.0460114-Neutral.htm) |
| 671 | 1416.03 | 338.61192 | 340.61882 | 209.10720 | 2.00690 | 359375 | 2 | 2 | [18](http://www.mycompoundid.org/mycompoundid_IsoMS/myid_search_res/1481082404607-2016-12-6-8-46-44-116748/1481082405194-538-209.1072018-Neutral.htm) |
| 672 | 1418.70 | 760.13421 | 762.14043 | 526.07589 | 2.00622 | 23725 | 1 | 1 | [2](http://www.mycompoundid.org/mycompoundid_IsoMS/myid_search_res/1481082404607-2016-12-6-8-46-44-116748/1481082405195-908-526.0758853-Neutral.htm) |
| 673 | 1418.87 | 760.33615 | 762.34165 | 526.27783 | 2.00550 | 14600 | 1 | 1 | [17](http://www.mycompoundid.org/mycompoundid_IsoMS/myid_search_res/1481082404607-2016-12-6-8-46-44-116748/1481082405195-252-526.2778285-Neutral.htm) |
| 674 | 1419.44 | 950.41676 | 952.42586 | 716.35844 | 2.00909 | 16750 | 1 | 1 | [2](http://www.mycompoundid.org/mycompoundid_IsoMS/myid_search_res/1481082404607-2016-12-6-8-46-44-116748/1481082405196-595-716.3584435-Neutral.htm) |
| 675 | 1420.81 | 573.47092 | 575.47781 | 678.82520 | 2.00689 | 57300 | 2 | 2 | [1](http://www.mycompoundid.org/mycompoundid_IsoMS/myid_search_res/1481082404607-2016-12-6-8-46-44-116748/1481082405196-105-678.8252038-Neutral.htm) |
| 676 | 1422.21 | 757.34630 | 759.35453 | 523.28798 | 2.00823 | 6500 | 1 | 1 | [9](http://www.mycompoundid.org/mycompoundid_IsoMS/myid_search_res/1481082404607-2016-12-6-8-46-44-116748/1481082405196-968-523.287979-Neutral.htm) |
| 677 | 1422.72 | 479.20212 | 481.20688 | 245.14380 | 2.00476 | 8210 | 1 | 1 | [1](http://www.mycompoundid.org/mycompoundid_IsoMS/myid_search_res/1481082404607-2016-12-6-8-46-44-116748/1481082405197-461-245.143795-Neutral.htm) |
| 678 | 1422.84 | 908.41991 | 910.42194 | 674.36159 | 2.00203 | 6650 | 1 | 1 | [5](http://www.mycompoundid.org/mycompoundid_IsoMS/myid_search_res/1481082404607-2016-12-6-8-46-44-116748/1481082405197-259-674.361594-Neutral.htm) |
| 679 | 1422.91 | 573.21914 | 575.22804 | 339.16082 | 2.00891 | 42200 | 1 | 1 | [2](http://www.mycompoundid.org/mycompoundid_IsoMS/myid_search_res/1481082404607-2016-12-6-8-46-44-116748/1481082405198-584-339.160817-Neutral.htm) |
| 680 | 1425.70 | 323.60645 | 325.61317 | 179.09626 | 2.00672 | 12753 | 2 | 2 | [27](http://www.mycompoundid.org/mycompoundid_IsoMS/myid_search_res/1481082404607-2016-12-6-8-46-44-116748/1481082405198-761-179.096262-Neutral.htm) |
| 681 | 1427.03 | 327.12655 | 329.13383 | 186.13647 | 2.00728 | 28450 | 2 | 2 | [4](http://www.mycompoundid.org/mycompoundid_IsoMS/myid_search_res/1481082404607-2016-12-6-8-46-44-116748/1481082405199-771-186.1364685-Neutral.htm) |
| 682 | 1427.52 | 331.08949 | 333.09666 | 194.06234 | 2.00718 | 19534 | 2 | 2 | [5](http://www.mycompoundid.org/mycompoundid_IsoMS/myid_search_res/1481082404607-2016-12-6-8-46-44-116748/1481082405199-114-194.0623384-Neutral.htm) |
| 683 | 1436.73 | 426.17300 | 428.17975 | 192.11468 | 2.00674 | 83602 | 1 | 1 | [10](http://www.mycompoundid.org/mycompoundid_IsoMS/myid_search_res/1481082404607-2016-12-6-8-46-44-116748/1481082405201-193-192.1146837-Neutral.htm) |
| 684 | 1437.72 | 319.57332 | 321.57954 | 171.03000 | 2.00622 | 42131 | 2 | 2 | [2](http://www.mycompoundid.org/mycompoundid_IsoMS/myid_search_res/1481082404607-2016-12-6-8-46-44-116748/1481082405201-239-171.0300026-Neutral.htm) |
| 685 | 1439.19 | 361.07257 | 363.07864 | 254.02849 | 2.00607 | 36500 | 2 | 2 | [5](http://www.mycompoundid.org/mycompoundid_IsoMS/myid_search_res/1481082404607-2016-12-6-8-46-44-116748/1481082405202-498-254.028494-Neutral.htm) |
| 686 | 1442.11 | 623.22074 | 625.22677 | 389.16242 | 2.00603 | 61775 | 1 | 1 | [23](http://www.mycompoundid.org/mycompoundid_IsoMS/myid_search_res/1481082404607-2016-12-6-8-46-44-116748/1481082405202-652-389.162419-Neutral.htm) |
| 687 | 1443.07 | 293.59553 | 295.60257 | 119.07442 | 2.00704 | 20775 | 2 | 2 | [11](http://www.mycompoundid.org/mycompoundid_IsoMS/myid_search_res/1481082404607-2016-12-6-8-46-44-116748/1481082405203-808-119.07442-Neutral.htm) |
| 688 | 1446.23 | 822.36222 | 824.36827 | 588.30390 | 2.00605 | 55663 | 1 | 1 | [8](http://www.mycompoundid.org/mycompoundid_IsoMS/myid_search_res/1481082404607-2016-12-6-8-46-44-116748/1481082405204-619-588.3039008-Neutral.htm) |
| 689 | 1449.13 | 832.23448 | 834.23320 | 598.17616 | 1.99872 | 25500 | 1 | 1 | [4](http://www.mycompoundid.org/mycompoundid_IsoMS/myid_search_res/1481082404607-2016-12-6-8-46-44-116748/1481082405204-520-598.1761555-Neutral.htm) |
| 690 | 1449.94 | 854.21634 | 856.21590 | 620.15802 | 1.99956 | 18700 | 1 | 1 | [1](http://www.mycompoundid.org/mycompoundid_IsoMS/myid_search_res/1481082404607-2016-12-6-8-46-44-116748/1481082405205-806-620.158021-Neutral.htm) |
| 691 | 1461.50 | 483.26699 | 485.27431 | 249.20867 | 2.00732 | 35800 | 1 | 1 | [1](http://www.mycompoundid.org/mycompoundid_IsoMS/myid_search_res/1481082404607-2016-12-6-8-46-44-116748/1481082405206-419-249.208671-Neutral.htm) |
| 692 | 1465.87 | 865.19813 | 867.20610 | 631.13981 | 2.00797 | 7260 | 1 | 1 | [1](http://www.mycompoundid.org/mycompoundid_IsoMS/myid_search_res/1481082404607-2016-12-6-8-46-44-116748/1481082405207-533-631.139806-Neutral.htm) |
| # | RT (s) | mz_light | mz_heavy | mz | distance | int_light | nCharge | nTag | Possible hits |
| 693 | 1469.02 | 620.23595 | 622.24172 | 386.17763 | 2.00577 | 30000 | 1 | 1 | [15](http://www.mycompoundid.org/mycompoundid_IsoMS/myid_search_res/1481082404607-2016-12-6-8-46-44-116748/1481082405207-25-386.177634-Neutral.htm) |
| 694 | 1469.04 | 923.42970 | 925.43666 | 689.37138 | 2.00696 | 40225 | 1 | 1 | [3](http://www.mycompoundid.org/mycompoundid_IsoMS/myid_search_res/1481082404607-2016-12-6-8-46-44-116748/1481082405208-212-689.3713788-Neutral.htm) |
| 695 | 1472.09 | 623.22101 | 625.22737 | 389.16269 | 2.00636 | 109919 | 1 | 1 | [14](http://www.mycompoundid.org/mycompoundid_IsoMS/myid_search_res/1481082404607-2016-12-6-8-46-44-116748/1481082405209-861-389.1626873-Neutral.htm) |
| 696 | 1474.50 | 826.36430 | 828.37156 | 592.30598 | 2.00726 | 7655 | 1 | 1 | [3](http://www.mycompoundid.org/mycompoundid_IsoMS/myid_search_res/1481082404607-2016-12-6-8-46-44-116748/1481082405210-855-592.3059768-Neutral.htm) |
| 697 | 1476.06 | 621.15781 | 625.17156 | 154.04847 | 4.01374 | 21375 | 1 | 2 | [12](http://www.mycompoundid.org/mycompoundid_IsoMS/myid_search_res/1481082404607-2016-12-6-8-46-44-116748/1481082405210-672-154.0484734-Neutral.htm) |
| 698 | 1483.37 | 792.31717 | 794.31760 | 558.25885 | 2.00043 | 8260 | 1 | 1 | [12](http://www.mycompoundid.org/mycompoundid_IsoMS/myid_search_res/1481082404607-2016-12-6-8-46-44-116748/1481082405211-282-558.258854-Neutral.htm) |
| 699 | 1484.39 | 578.23513 | 580.24152 | 344.17681 | 2.00638 | 53263 | 1 | 1 | [1](http://www.mycompoundid.org/mycompoundid_IsoMS/myid_search_res/1481082404607-2016-12-6-8-46-44-116748/1481082405211-16-344.1768138-Neutral.htm) |
| 700 | 1486.25 | 669.32514 | 673.33790 | 202.21580 | 4.01276 | 15800 | 1 | 2 | [1](http://www.mycompoundid.org/mycompoundid_IsoMS/myid_search_res/1481082404607-2016-12-6-8-46-44-116748/1481082405213-250-202.215801-Neutral.htm) |
| 701 | 1486.89 | 570.29100 | 572.29316 | 336.23268 | 2.00216 | 11450 | 1 | 1 | [100](http://www.mycompoundid.org/mycompoundid_IsoMS/myid_search_res/1481082404607-2016-12-6-8-46-44-116748/1481082405213-728-336.232678-Neutral.htm) |
| 702 | 1492.33 | 485.21034 | 487.21635 | 251.15202 | 2.00602 | 47225 | 1 | 1 | [5](http://www.mycompoundid.org/mycompoundid_IsoMS/myid_search_res/1481082404607-2016-12-6-8-46-44-116748/1481082405217-808-251.1520153-Neutral.htm) |
| 703 | 1493.26 | 685.32688 | 687.33352 | 451.26856 | 2.00664 | 11730 | 1 | 1 | [24](http://www.mycompoundid.org/mycompoundid_IsoMS/myid_search_res/1481082404607-2016-12-6-8-46-44-116748/1481082405217-220-451.2685635-Neutral.htm) |
| 704 | 1497.86 | 456.21945 | 458.22670 | 222.16113 | 2.00725 | 52984 | 1 | 1 | [2](http://www.mycompoundid.org/mycompoundid_IsoMS/myid_search_res/1481082404607-2016-12-6-8-46-44-116748/1481082405218-868-222.1611332-Neutral.htm) |
| 705 | 1500.28 | 403.06362 | 405.06748 | 169.00530 | 2.00387 | 41691 | 1 | 1 | [9](http://www.mycompoundid.org/mycompoundid_IsoMS/myid_search_res/1481082404607-2016-12-6-8-46-44-116748/1481082405219-206-169.0052958-Neutral.htm) |
| 706 | 1506.00 | 468.21979 | 470.22597 | 234.16147 | 2.00618 | 18230 | 1 | 1 | [4](http://www.mycompoundid.org/mycompoundid_IsoMS/myid_search_res/1481082404607-2016-12-6-8-46-44-116748/1481082405219-450-234.161474-Neutral.htm) |
| 707 | 1506.67 | 584.30725 | 586.30957 | 350.24893 | 2.00232 | 9120 | 1 | 1 | [59](http://www.mycompoundid.org/mycompoundid_IsoMS/myid_search_res/1481082404607-2016-12-6-8-46-44-116748/1481082405220-605-350.2489345-Neutral.htm) |
| 708 | 1506.92 | 759.35082 | 761.35516 | 525.29250 | 2.00434 | 15900 | 1 | 1 | [9](http://www.mycompoundid.org/mycompoundid_IsoMS/myid_search_res/1481082404607-2016-12-6-8-46-44-116748/1481082405222-720-525.2925008-Neutral.htm) |
| 709 | 1511.93 | 757.34615 | 759.35216 | 523.28783 | 2.00601 | 36700 | 1 | 1 | [9](http://www.mycompoundid.org/mycompoundid_IsoMS/myid_search_res/1481082404607-2016-12-6-8-46-44-116748/1481082405223-373-523.287833-Neutral.htm) |
| 710 | 1513.80 | 735.34388 | 737.35017 | 501.28556 | 2.00629 | 94963 | 1 | 1 | [28](http://www.mycompoundid.org/mycompoundid_IsoMS/myid_search_res/1481082404607-2016-12-6-8-46-44-116748/1481082405223-528-501.2855619-Neutral.htm) |
| 711 | 1516.31 | 556.27458 | 558.27698 | 322.21626 | 2.00240 | 9940 | 1 | 1 | [53](http://www.mycompoundid.org/mycompoundid_IsoMS/myid_search_res/1481082404607-2016-12-6-8-46-44-116748/1481082405224-994-322.216256-Neutral.htm) |
| 712 | 1520.00 | 865.19981 | 867.20924 | 631.14149 | 2.00943 | 14050 | 1 | 1 | [1](http://www.mycompoundid.org/mycompoundid_IsoMS/myid_search_res/1481082404607-2016-12-6-8-46-44-116748/1481082405227-116-631.1414875-Neutral.htm) |
| 713 | 1521.27 | 759.35280 | 761.35771 | 525.29448 | 2.00491 | 24800 | 1 | 1 | [8](http://www.mycompoundid.org/mycompoundid_IsoMS/myid_search_res/1481082404607-2016-12-6-8-46-44-116748/1481082405227-848-525.294481-Neutral.htm) |
| 714 | 1523.31 | 711.34181 | 713.35179 | 477.28349 | 2.00998 | 7930 | 1 | 1 | [25](http://www.mycompoundid.org/mycompoundid_IsoMS/myid_search_res/1481082404607-2016-12-6-8-46-44-116748/1481082405227-586-477.283487-Neutral.htm) |
| 715 | 1525.89 | 598.32315 | 600.32440 | 364.26483 | 2.00125 | 9490 | 1 | 1 | [37](http://www.mycompoundid.org/mycompoundid_IsoMS/myid_search_res/1481082404607-2016-12-6-8-46-44-116748/1481082405228-565-364.2648256-Neutral.htm) |
| 716 | 1527.21 | 348.11694 | 350.12275 | 228.11724 | 2.00582 | 27250 | 2 | 2 | [2](http://www.mycompoundid.org/mycompoundid_IsoMS/myid_search_res/1481082404607-2016-12-6-8-46-44-116748/1481082405230-219-228.117235-Neutral.htm) |
| # | RT (s) | mz_light | mz_heavy | mz | distance | int_light | nCharge | nTag | Possible hits |
| 717 | 1529.00 | 482.23612 | 484.24262 | 248.17780 | 2.00650 | 239048 | 1 | 1 | [1](http://www.mycompoundid.org/mycompoundid_IsoMS/myid_search_res/1481082404607-2016-12-6-8-46-44-116748/1481082405230-652-248.1778029-Neutral.htm) |
| 718 | 1531.21 | 584.18886 | 586.19478 | 350.13054 | 2.00592 | 65250 | 1 | 1 | [15](http://www.mycompoundid.org/mycompoundid_IsoMS/myid_search_res/1481082404607-2016-12-6-8-46-44-116748/1481082405230-963-350.130536-Neutral.htm) |
| 719 | 1531.63 | 440.22542 | 442.23200 | 206.16710 | 2.00658 | 289097 | 1 | 1 | [1](http://www.mycompoundid.org/mycompoundid_IsoMS/myid_search_res/1481082404607-2016-12-6-8-46-44-116748/1481082405231-120-206.1670985-Neutral.htm) |
| 720 | 1532.61 | 761.35818 | 763.36785 | 527.29986 | 2.00967 | 13400 | 1 | 1 | [16](http://www.mycompoundid.org/mycompoundid_IsoMS/myid_search_res/1481082404607-2016-12-6-8-46-44-116748/1481082405231-967-527.299863-Neutral.htm) |
| 721 | 1543.21 | 519.28734 | 521.29393 | 285.22902 | 2.00659 | 23750 | 1 | 1 | [6](http://www.mycompoundid.org/mycompoundid_IsoMS/myid_search_res/1481082404607-2016-12-6-8-46-44-116748/1481082405233-166-285.2290225-Neutral.htm) |
| 722 | 1549.90 | 533.33992 | 535.34661 | 299.28160 | 2.00669 | 57257 | 1 | 1 | [14](http://www.mycompoundid.org/mycompoundid_IsoMS/myid_search_res/1481082404607-2016-12-6-8-46-44-116748/1481082405233-286-299.2815992-Neutral.htm) |
| 723 | 1553.12 | 669.35654 | 671.36305 | 435.29822 | 2.00651 | 6390 | 1 | 1 | [17](http://www.mycompoundid.org/mycompoundid_IsoMS/myid_search_res/1481082404607-2016-12-6-8-46-44-116748/1481082405234-927-435.298221-Neutral.htm) |
| 724 | 1566.31 | 535.35505 | 537.36188 | 301.29673 | 2.00683 | 21875 | 1 | 1 | [8](http://www.mycompoundid.org/mycompoundid_IsoMS/myid_search_res/1481082404607-2016-12-6-8-46-44-116748/1481082405235-740-301.2967315-Neutral.htm) |
| 725 | 1568.14 | 547.35599 | 549.36172 | 313.29767 | 2.00573 | 14800 | 1 | 1 | [10](http://www.mycompoundid.org/mycompoundid_IsoMS/myid_search_res/1481082404607-2016-12-6-8-46-44-116748/1481082405236-15-313.297667-Neutral.htm) |
| 726 | 1569.01 | 454.23984 | 456.24643 | 220.18152 | 2.00659 | 60752 | 1 | 1 | [3](http://www.mycompoundid.org/mycompoundid_IsoMS/myid_search_res/1481082404607-2016-12-6-8-46-44-116748/1481082405236-439-220.1815198-Neutral.htm) |
| 727 | 1569.71 | 417.12120 | 419.12817 | 183.06288 | 2.00697 | 60613 | 1 | 1 | [15](http://www.mycompoundid.org/mycompoundid_IsoMS/myid_search_res/1481082404607-2016-12-6-8-46-44-116748/1481082405236-428-183.0628771-Neutral.htm) |
| 728 | 1569.99 | 666.36798 | 668.37369 | 432.30966 | 2.00571 | 13950 | 1 | 1 | [4](http://www.mycompoundid.org/mycompoundid_IsoMS/myid_search_res/1481082404607-2016-12-6-8-46-44-116748/1481082405237-236-432.309658-Neutral.htm) |
| 729 | 1574.10 | 489.31422 | 491.32056 | 255.25590 | 2.00634 | 104750 | 1 | 1 | [2](http://www.mycompoundid.org/mycompoundid_IsoMS/myid_search_res/1481082404607-2016-12-6-8-46-44-116748/1481082405238-910-255.2558965-Neutral.htm) |
| 730 | 1580.99 | 515.32889 | 517.33456 | 281.27057 | 2.00567 | 7580 | 1 | 1 | [4](http://www.mycompoundid.org/mycompoundid_IsoMS/myid_search_res/1481082404607-2016-12-6-8-46-44-116748/1481082405238-968-281.270565-Neutral.htm) |
| 731 | 1582.57 | 455.13753 | 457.14468 | 221.07921 | 2.00715 | 512983 | 1 | 1 | [4](http://www.mycompoundid.org/mycompoundid_IsoMS/myid_search_res/1481082404607-2016-12-6-8-46-44-116748/1481082405239-579-221.0792067-Neutral.htm) |
| 732 | 1602.43 | 687.34515 | 689.35050 | 453.28683 | 2.00535 | 6765 | 1 | 1 | [22](http://www.mycompoundid.org/mycompoundid_IsoMS/myid_search_res/1481082404607-2016-12-6-8-46-44-116748/1481082405240-686-453.286833-Neutral.htm) |
| 733 | 1619.19 | 713.35902 | 715.36405 | 479.30070 | 2.00503 | 21000 | 1 | 1 | [27](http://www.mycompoundid.org/mycompoundid_IsoMS/myid_search_res/1481082404607-2016-12-6-8-46-44-116748/1481082405241-815-479.300698-Neutral.htm) |
| 734 | 1620.14 | 517.34507 | 519.35134 | 283.28675 | 2.00627 | 73725 | 1 | 1 | [10](http://www.mycompoundid.org/mycompoundid_IsoMS/myid_search_res/1481082404607-2016-12-6-8-46-44-116748/1481082405242-522-283.2867538-Neutral.htm) |
| 735 | 1631.72 | 713.35865 | 715.36492 | 479.30033 | 2.00626 | 19620 | 1 | 1 | [27](http://www.mycompoundid.org/mycompoundid_IsoMS/myid_search_res/1481082404607-2016-12-6-8-46-44-116748/1481082405243-476-479.3003345-Neutral.htm) |
| 736 | 1634.05 | 575.35016 | 577.35687 | 341.29184 | 2.00671 | 32300 | 1 | 1 | [16](http://www.mycompoundid.org/mycompoundid_IsoMS/myid_search_res/1481082404607-2016-12-6-8-46-44-116748/1481082405244-697-341.291839-Neutral.htm) |
| 737 | 1689.44 | 671.34698 | 673.35294 | 437.28866 | 2.00596 | 7760 | 1 | 1 | [9](http://www.mycompoundid.org/mycompoundid_IsoMS/myid_search_res/1481082404607-2016-12-6-8-46-44-116748/1481082405245-664-437.2886599-Neutral.htm) |
| 738 | 1707.69 | 671.34857 | 673.35182 | 437.29025 | 2.00325 | 7600 | 1 | 1 | [9](http://www.mycompoundid.org/mycompoundid_IsoMS/myid_search_res/1481082404607-2016-12-6-8-46-44-116748/1481082405245-928-437.290247-Neutral.htm) |
| 739 | 1707.69 | 697.36235 | 699.37029 | 463.30403 | 2.00794 | 5620 | 1 | 1 | [19](http://www.mycompoundid.org/mycompoundid_IsoMS/myid_search_res/1481082404607-2016-12-6-8-46-44-116748/1481082405246-681-463.304029-Neutral.htm) |
|  |  |  |  |  |  |  |  |  |  |

**Table S2.** List of the compounds presented in Figure 4 of these nine metabolic pathways.

| KEGG ID | | | Compound name | | KEGG ID | | | Compound name | | |
| --- | --- | --- | --- | --- | --- | --- | --- | --- | --- | --- |
| C00010 | | Coenzyme A | | | C00011 | | Carbon dioxide | | | |
| C00012 | | Peptide | | | C00014 | | Ammonia | | | |
| C00015 | | Uridine 5'-diphosphate | | | C00019 | | S-Adenosylmethionine | | | |
| C00020 | | Adenosine monophosphate | | | C00022 | | Pyruvic acid | | | |
| C00024 | | Acetyl-CoA | | | C00025 | | L-Glutamic acid | | | |
| C00026 | | Oxoglutaric acid | | | C00029 | | Uridine diphosphate glucose | | | |
| C00033 | | Acetic acid | | | C00036 | | Oxalacetic acid | | | |
| C00037 | | Glycine | | | C00039 | | DNA | | | |
| C00041 | | L-Alanine | | | C00042 | | Succinic acid | | | |
| C00046 | | RNA | | | C00048 | | Glyoxylic acid | | | |
| C00049 | | L-Aspartic acid | | | C00054 | | Adenosine 3',5'-diphosphate | | | |
| C00055 | | Cytidine monophosphate | | | C00058 | | Formic acid | | | |
| C00062 | | L-Arginine | | | C00063 | | Cytidine triphosphate | | | |
| C00064 | | L-Glutamine | | | C00068 | | Thiamine pyrophosphate | | | |
| C00075 | | Uridine triphosphate | | | C00077 | | Ornithine | | | |
| C00078 | | L-Tryptophan | | | C00079 | | L-Phenylalanine | | | |
| C00082 | | L-Tyrosine | | | C00083 | | Malonyl-CoA | | | |
| C00086 | | Urea | | | C00088 | | Nitrite | | | |
| C00094 | | Sulfite | | | C00097 | | L-Cysteine | | | |
| C00099 | | Beta-Alanine | | | C00100 | | Propionyl-CoA | | | |
| KEGG ID | | | Compound name | | KEGG ID | | | | | Compound name |
| C00105 | | | Uridine 5'-monophosphate | | C00106 | | | | | Uracil |
| C00108 | | | 2-Aminobenzoic acid | | C00112 | | | | | CDP |
| C00119 | | | Phosphoribosyl pyrophosphate | | C00122 | | | | | Fumaric acid |
| C00134 | | | Putrescine | | C00135 | | | | | L-Histidine |
| C00136 | | | Butanoyl-CoA | | C00141 | | | | | Alpha-ketoisovaleric acid |
| C00148 | | | L-Proline | | C00152 | | | | | L-Asparagine |
| C00155 | | | L-Homocysteine | | C00164 | | | | | Acetoacetic acid |
| C00169 | | | Carbamoylphosphate | | C00178 | | | | | Thymine |
| C00179 | | | Agmatine | | C00183 | | | | | L-Valine |
| C00187 | | | Cholesterol | | C00192 | | | | | Hydroxylamine |
| C00213 | | | Sarcosine | | C00214 | | | | | Thymidine |
| C00222 | | | Malonic semialdehyde | | C00227 | | | | | Acetylphosphate |
| C00229 | | | Acyl-carrier protein | | C00232 | | | | | Succinic acid semialdehyde |
| C00239 | | | dCMP | | C00241 | | | | | Amide |
| C00244 | | | Nitrate | | C00245 | | | | | Taurine |
| C00246 | | | Butyric acid | | C00295 | | | | | Orotic acid |
| C00299 | | | Uridine | | C00300 | | | | | Creatine |
| C00315 | | | Spermidine | | C00327 | | | | | Citrulline |
| C00332 | | | Acetoacetyl-CoA | | C00334 | | | | | Gamma-Aminobutyric acid |
| C00337 | | | 4,5-Dihydroorotic acid | | C00342 | | | | | Thioredoxin |
| C00343 | | | Thioredoxin disulfide | | C00352 | | | | | Glucosamine 6-phosphate |
| C00356 | | | 3-Hydroxy-3-methylglutaryl-CoA | | C00363 | | | | | dTDP |
| C00364 | | | 5-Thymidylic acid | | C00365 | | | | | dUMP |
| KEGG ID | | | Compound name | | KEGG ID | | | | | Compound name |
| C00380 | | Cytosine | | | C00383 | | Malonic acid | | | |
| C00386 | | Carnosine | | | C00402 | | D-Aspartic acid | | | |
| C00429 | | Dihydrouracil | | | C00431 | | 5-Aminopentanoic acid | | | |
| C00433 | | 2,5-Dioxopentanoate | | | C00436 | | N-Carbamoylputrescine | | | |
| C00437 | | N-Acetylornithine | | | C00438 | | Ureidosuccinic acid | | | |
| C00458 | | dCTP | | | C00459 | | Thymidine 5'-triphosphate | | | |
| C00460 | | Deoxyuridine triphosphate | | | C00475 | | Cytidine | | | |
| C00488 | | Formamide | | | C00497 | | (R)-Malate | | | |
| C00506 | | Cysteic acid | | | C00519 | | Hypotaurine | | | |
| C00522 | | (R)-Pantoate | | | C00526 | | Deoxyuridine | | | |
| C00533 | | Nitric oxide | | | C00542 | | Allocystathionine | | | |
| C00555 | | 4-Aminobutyraldehyde | | | C00581 | | Guanidoacetic acid | | | |
| C00593 | | Sulfoacetaldehyde | | | C00606 | | 3-Sulfinoalanine | | | |
| C00624 | | N-Acetyl-L-alanine | | | C00672 | | Deoxyribose 1-phosphate | | | |
| C00695 | | Cholic acid | | | C00697 | | Nitrogen | | | |
| C00705 | | dCDP | | | C00706 | | Amine | | | |
| C00726 | | Nitrile | | | C00741 | | Diacetyl | | | |
| C00750 | | Spermine | | | C00763 | | D-Proline | | | |
| C00791 | | Creatinine | | | C00804 | | Propynoic acid | | | |
| C00810 | | (R)-Acetoin | | | C00813 | | Barbiturate | | | |
| C00831 | | Pantetheine | | | C00864 | | Pantothenic acid | | | |
| C00877 | | Crotonoyl-CoA | | | C00881 | | Deoxycytidine | | | |
| C00882 | | Dephospho-CoA | | | C00884 | | Homocarnosine | | | |
| KEGG ID | | | Compound name | | KEGG ID | | | | Compound name | |
| C00887 | | | Nitrous oxide | | C00894 | | | Acrylyl-CoA | | |
| C00900 | | | 2-Acetolactate | | C00906 | | | Dihydrothymine | | |
| C00940 | | | 2-Keto-glutaramic acid | | C00966 | | | 2-Dehydropantoate | | |
| C00986 | | | 1,3-Diaminopropane | | C00989 | | | 4-Hydroxybutyric acid | | |
| C01010 | | | Urea-1-carboxylate | | C01013 | | | Hydroxypropionic acid | | |
| C01035 | | | 4-Guanidinobutanoic acid | | C01042 | | | N-Acetyl-L-aspartic acid | | |
| C01043 | | | N-Carbamoylsarcosine | | C01053 | | | (R)-4-Dehydropantoate | | |
| C01073 | | | N-Acetyl-beta-alanine | | C01088 | | | (R)-3,3-Dimethylmalate | | |
| C01089 | | | (R)-3-Hydroxybutyric acid | | C01103 | | | Orotidylic acid | | |
| C01110 | | | 5-Amino-2-oxopentanoic acid | | C01134 | | | Pantetheine 4'-phosphate | | |
| C01137 | | | S-Adenosylmethioninamine | | C01144 | | | (S)-3-Hydroxybutanoyl-CoA | | |
| C01157 | | | Hydroxyproline | | C01165 | | | L-Glutamic-gamma-semialdehyde | | |
| C01168 | | | Pseudouridine 5'-phosphate | | C01250 | | | N-Acetyl-L-glutamate 5-semialdehyde | | |
| C01262 | | | Anserine | | C01301 | | | 3a,7a,12a-Trihydroxy-5b-cholestan-26-al | | |
| C01346 | | | dUDP | | C01353 | | | Carbonic acid | | |
| C01358 | | | NH4OH | | C01368 | | | 3'-UMP | | |
| C01384 | | | Maleic acid | | C01412 | | | Butanal | | |
| C01417 | | | Cyanate | | C01563 | | | Carbamic acid | | |
| C01678 | | | Cysteamine | | C01682 | | | Nopaline | | |
| C01769 | | | (S)-Acetoin | | C01794 | | | Choloyl-CoA | | |
| C01837 | | | Nitroethane | | C01877 | | | 4-Oxoproline | | |
| C01921 | | | Glycocholic acid | | C01959 | | | Taurocyamine | | |
| C02067 | | | Pseudouridine | | C02170 | | | Methylmalonic acid | | |
| KEGG ID | | | Compound name | | KEGG ID | | | Compound name | | |
| C02291 | | | L-Cystathionine | | | C02305 | | Phosphocreatine | | |
| C02331 | | | Vinylacetyl-CoA | | | C02335 | | Beta-Alanyl-CoA | | |
| C02354 | | 2',3'-Cyclic CMP | | | | C02355 | 2',3'-Cyclic UMP | | | |
| C02362 | | 2-Oxosuccinamate | | | | C02376 | 5-Methylcytosine | | | |
| C02411 | | Glutaconyl-1-CoA | | | | C02466 | Trimetaphosphate | | | |
| C02527 | | Butanoylphosphate | | | | C02528 | Chenodeoxycholic acid | | | |
| C02565 | | N-Methylhydantoin | | | | C02630 | 2-Hydroxyglutarate | | | |
| C02642 | | Ureidopropionic acid | | | | C02647 | 4-Guanidinobutanal | | | |
| C02714 | | N-Acetylputrescine | | | | C02946 | 4-Acetamidobutanoic acid | | | |
| C03044 | | (R,R)-Butane-2,3-diol | | | | C03046 | (S,S)-Butane-2,3-diol | | | |
| C03058 | | 2-Hydroxyglutaryl-CoA | | | | C03078 | 4-Guanidinobutanamide | | | |
| C03090 | | 5-Phosphoribosylamine | | | | C03149 | N-Phosphotaurocyamine | | | |
| C03166 | | Phosphoguanidinoacetate | | | | C03287 | L-Glutamic acid 5-phosphate | | | |
| C03296 | | N2-Succinyl-L-arginine | | | | C03406 | Argininosuccinic acid | | | |
| C03415 | | N2-Succinyl-L-ornithine | | | | C03440 | cis-4-Hydroxy-D-proline | | | |
| C03492 | | D-4'-Phosphopantothenate | | | | C03561 | 3-Hydroxybutyryl-CoA | | | |
| C03564 | | 1-Pyrroline-2-carboxylic acid | | | | C03594 | 7a-Hydroxycholesterol | | | |
| C03618 | | L-threo-3-Methylaspartate | | | | C03688 | Apo-[acyl-carrier-protein] | | | |
| C03771 | | 5-Guanidino-2-oxopentanoate | | | | C03794 | Adenylsuccinic acid | | | |
| C03912 | | (S)-1-Pyrroline-5-carboxylate | | | | C03997 | 5-Hydroxymethyldeoxycytidylate | | | |
| C04039 | | 2,3-Dihydroxy-3-methylbutanoate | | | | C04079 | D-Pantothenoyl-L-cysteine | | | |
| C04133 | | N-Acetyl-L-glutamyl 5-phosphate | | | | C04137 | N2-(D-1-Carboxyethyl)-L-arginine | | | |
| C04281 | | Pyrroline hydroxycarboxylic acid | | | | C04282 | 1-Pyrroline-4-hydroxy-2-carboxylate | | | |
| KEGG ID | | | | Compound name | KEGG ID | | | | Compound name | |
| C04352 | 4-Phosphopantothenoylcysteine | | | | C04546 | | (R)-3-((R)-3-Hydroxybutanoyloxy)butanoate | | | |
| C04554 | 3alpha,7alpha-Dihydroxy-5beta-cholestanate | | | | C04722 | | 3a,7a,12a-Trihydroxy-5b-cholestanoic acid | | | |
| C05100 | Ureidoisobutyric acid | | | | C05122 | | Taurocholic acid | | | |
| C05123 | 2-Hydroxyethanesulfonate | | | | C05125 | | 2-(a-Hydroxyethyl)thiamine diphosphate | | | |
| C05145 | 3-Aminoisobutanoic acid | | | | C05167 | | alpha-Amino acid | | | |
| C05281 | 5-Methylbarbiturate | | | | C05337 | | Chenodeoxycholoyl-CoA | | | |
| C05340 | beta-Alanyl-L-arginine | | | | C05341 | | beta-Alanyl-L-lysine | | | |
| C05444 | 3alpha,7alpha,26-Trihydroxy-5beta-cholestane | | | | C05445 | | 3a,7a-Dihydroxy-5b-cholestan-26-al | | | |
| C05446 | 3alpha,7alpha,12alpha,26-Tetrahydroxy-5beta-cholestane | | | | C05447 | | 3a,7a-Dihydroxy-5b-cholest-24-enoyl-CoA | | | |
| C05448 | 3a,7a,12a-Trihydroxy-5b-cholestanoyl-CoA | | | | C05449 | | 3a,7a-Dihydroxy-5b-24-oxocholestanoyl-CoA | | | |
| C05450 | 3a,7a,12a,24-Tetrahydroxy-5b-cholestanoyl-CoA | | | | C05451 | | 7a-Hydroxy-5b-cholestan-3-one | | | |
| C05452 | 3a,7a-Dihydroxy-5b-cholestane | | | | C05453 | | 7a,12a-Dihydroxy-5b-cholestan-3-one | | | |
| C05454 | 5-b-Cholestane-3a ,7a ,12a-triol | | | | C05455 | | 7a-Hydroxy-cholestene-3-one | | | |
| C05460 | 3a,7a,12a-Trihydroxy-5b-cholest-24-enoyl-CoA | | | | C05465 | | Taurochenodesoxycholic acid | | | |
| C05466 | Chenodeoxycholic acid glycine conjugate | | | | C05467 | | 3a,7a,12a-Trihydroxy-5b-24-oxocholestanoyl-CoA | | | |
| C05468 | 5b-Cyprinol sulfate | | | | C05665 | | 3-Aminopropionaldehyde | | | |
| C05668 | 3-Hydroxypropionyl-CoA | | | | C05822 | | 3'-CMP | | | |
| C05844 | 5-L-Glutamyl-taurine | | | | C05931 | | N-Succinyl-L-glutamate | | | |
| C05932 | N2-Succinyl-L-glutamic acid 5-semialdehyde | | | | C05933 | | N-(o)-Hydroxyarginine | | | |
| C05936 | N4-Acetylaminobutanal | | | | C05938 | | L-4-Hydroxyglutamate semialdehyde | | | |
| C05939 | Linatine | | | | C05941 | | 2-Oxo-4-hydroxy-5-aminovalerate | | | |
| C05942 | | | Pyrrole-2-carboxylic | | C05944 | | Pantothenol | | | |
| KEGG ID | | | Compound name | | KEGG ID | | | Compound name | | |
| C05945 | | | L-Arginine phosphate | | C05946 | | D-4-Hydroxy-2-oxoglutarate | | | |
| C05947 | | L-erythro-4-Hydroxyglutamate | | | C06059 | | Cyclic amidines | | | |
| C06060 | | Amidines | | | C06142 | | 1-Butanol | | | |
| C06143 | | Poly-beta-hydroxybutyrate | | | C06144 | | 3-Butynoate | | | |
| C06145 | | 3-Butyn-1-al | | | C06146 | | 3-Butyn-1-ol | | | |
| C06198 | | P1,P4-Bis(5'-uridyl) tetraphosphate | | | C06341 | | 7-a,27-dihydroxycholesterol | | | |
| C06735 | | Aminoacetaldehyde | | | C11038 | | 2'-Deoxy-5-hydroxymethylcytidine-5'-diphosphate | | | |
| C11039 | | 2'-Deoxy-5-hydroxymethylcytidine-5'-triphosphate | | | C13550 | | 24-Hydroxycholesterol | | | |
| C14179 | | Sulfoacetate | | | C15518 | | (24S)-Cholest-5-ene-3beta,7alpha,24-triol | | | |
| C15519 | | 25-Hydroxycholesterol | | | C15520 | | 7-a,25-Dihydroxycholesterol | | | |
| C15607 | | 3-Oxo-3-ureidopropanoate | | | C15610 | | Cholest-5-ene-3beta,26-diol | | | |
| C15613 | | (25R)-3alpha,7alpha,12alpha-Trihydroxy-5beta-cholestan-26-oyl-CoA | | | C15699 | | Gamma-glutamyl-L-putrescine | | | |
| C15700 | | gamma-Glutamyl-gamma-aminobutyraldehyde | | | C15767 | | 4-(Glutamylamino) butanoate | | | |
| C17331 | | 7alpha,24-Dihydroxy-4-cholesten-3-one | | | C17332 | | alpha,25-Dihydroxy-4-cholesten-3-one | | | |
| C17333 | | 3 beta-Hydroxy-5-  cholestenoate | | | C17335 | | 3 beta,7 alpha-Dihydroxy-5-  cholestenoate | | | |
| C17336 | | 7 alpha,26-Dihydroxy-4-cholesten-3-one | | | C17337 | | 7 alpha-Hydroxy-3-oxo-4-cholestenoate | | | |
| C17339 | | 4-Cholesten-7alpha,12alpha-diol-3-one | | | C17343 | | (25S)-3alpha,7alpha,12alpha-Trihydroxy-5beta-cholestan-26-oyl-CoA | | | |
| C17345 | | (25R)-3alpha,7alpha-Dihydroxy-5beta-cholestanoyl-CoA | | | C17346 | | (25S)-3alpha,7alpha-Dihydroxy-5beta-cholestanoyl-CoA | | | |
| C18091 | | Ethylnitronate | | |  | |  | | | |
