# Supplementary material for: The Impact of GFP Reporter Gene Transduction and Expression on Metabolomics of Placental Mesenchymal Stem Cells Determined by UHPLC-Q/TOF-MS
Source: Stem Cells Int. 2017 Nov 5;2017:3167985. doi: 10.1155/2017/3167985 (PMC5694582; doi:10.1155/2017/3167985)
Supplement: Supplementary file 2 [file 3167985.f2.pdf]

Supplemental Data

The impact of GFP reporter gene transduction and expression on metabolomics of placental mesenchymal stem cells determined by UHPLC-Q/TOF-MS

Jinfeng Yang[1]*, Nan Wang[2]*, Deying Chen[1], Jiong Yu[1], Qiaoling Pan[1], Dan Wang[1], Jingqi Liu[1], Xiaowei Shi[3], Xiaotian Dong[1], Hongcui Cao[1]†, Liang Li[2], Lanjuan Li[1]

1 State Key Laboratory for the Diagnosis and Treatment of Infectious Diseases, The First Affiliated Hospital, College of Medicine, Zhejiang University; Collaborative Innovation Center for Diagnosis and Treatment of Infectious Diseases, 79 Qingchun Rd., Hangzhou City 310003, China

2 Collaborative Innovation Center for the Diagnosis and Treatment of Infectious Diseases, Zhejiang University, Hangzhou 310003, China; Department of Chemistry, University of Alberta, Edmonton, Alberta T6G 2G2, Canada

3 Chu Kochen Honors College, Zhejiang University, 866 Yuhangtang Rd., Hangzhou City310058, China

* These authors contributed equally to this work.

†Correspondence author:

Hongcui Cao, the State Key Laboratory for Diagnosis and Treatment of Infectious Diseases, The First Affiliated Hospital, College of Medicine, Zhejiang University; Collaborative Innovation Center for Diagnosis and Treatment of Infectious Diseases,79 Qingchun Rd., Hangzhou City 310003, China.Tel: 86-571-87236451; Fax: 86-571-87236459

E-mail: hccao@zju.edu.cn

Figure S1.

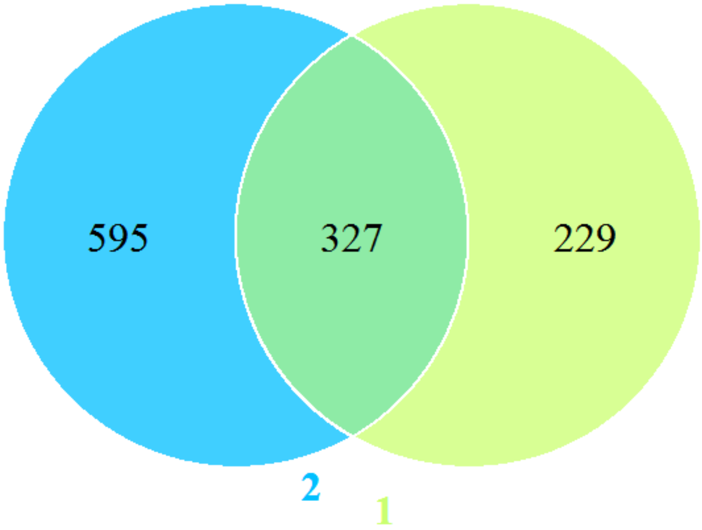

The Venn diagram shows the numbers of peak pairs detected using 9/1 MeOH/$CHCl_3$ (method 1) and 1/1 MeOH/H_2O (method 2) as extraction solvent respectively.

Table S1A. Identification of cellular metabolites based on the mass and retention time matches against the dansyl standard library.

Supplemental Table S1A. Identification of cellular metabolites based on the mass and retention time matches against the dansyl standard library.

#	Input mass	Input RT(min)	Calibrated RT (min)	HMDB.No.	Name	Accurate mass	mz_light	library RT	Mass error (Da)	RT error (min)
1	375.0800	3.08	1.80	HMDB00224	O-Phosphoethanolamine	141.0191	375.0774	2.02	0.0026	0.22
2	359.0735	3.35	1.95	HMDB00251	Taurine	125.0147	359.0730	2.24	0.0005	0.29
3	388.1080	3.48	2.03	HMDB00157	Hypoxanthine + H2O	136.0385	388.1098	2.12	0.0018	0.09
4	517.1505	3.57	2.09	HMDB00133	Guanosine	283.0917	517.1500	2.22	0.0005	0.13
5	359.0735	3.59	2.09	HMDB00251	Taurine	125.0147	359.0730	2.24	0.0005	0.15
6	517.1506	3.78	2.21	HMDB00133	Guanosine	283.0917	517.1500	2.22	0.0006	0.01
7	517.1502	3.99	2.33	HMDB00133	Guanosine	283.0917	517.1500	2.22	0.0002	0.11
8	408.1709	4.03	2.35	HMDB00517	L-Arginine	174.1117	408.1700	2.44	0.0009	0.09
9	403.1427	4.19	2.45	HMDB00001	1-Methylhistidine	169.0851	403.1434	2.17	0.0007	0.28
10	403.1427	4.19	2.45	HMDB00479	3-methyl-histidine	169.0851	403.1434	2.01	0.0007	0.44
11	408.1705	4.37	2.73	HMDB00517	L-Arginine	174.1117	408.1700	2.44	0.0005	0.29
12	366.1125	4.45	2.87	HMDB00168	L-Asparagine	132.0535	366.1118	3	0.0007	0.13
13	380.1279	4.91	3.63	HMDB00641	L-Glutamine	146.0691	380.1275	3.32	0.0004	0.31
14	380.1279	4.91	3.63	HMDB03423	D-Glutamine	146.0691	380.1275	3.32	0.0004	0.31
15	399.1044	5.14	4.00	HMDB02005	Methionine Sulfoxide	165.0460	399.1043	3.72	0.0001	0.28
16	399.1044	5.14	4.00	HMDB02005	Methionine Sulfoxide - Isomer	165.0460	399.1043	4.2	0.0001	0.20
17	353.1166	5.23	4.15	HMDB00719	L-Homoserine	119.0582	353.1166	4.05	0.0000	0.10
18	339.1030	5.23	4.15	HMDB00187	L-Serine	105.0426	339.1009	4.4	0.0021	0.25

#	Input mass	Input RT(min)	Calibrated RT (min)	HMDB.No.	Name	Accurate mass	mz_light	library RT	Mass error (Da)	RT error (min)
19	399.1046	5.41	4.44	HMDB02005	Methionine Sulfoxide - Isomer	165.0460	399.1043	4.2	0.0003	0.24
20	353.1157	5.47	4.50	HMDB00719	L-Homoserine	119.0582	353.1166	4.05	0.0009	0.45
21	339.1016	5.58	4.61	HMDB00187	L-Serine	105.0426	339.1009	4.4	0.0007	0.21
22	381.1122	5.61	4.63	HMDB00148	L-Glutamic Acid	147.0532	381.1115	5.05	0.0007	0.42
23	367.0991	5.66	4.68	HMDB00191	L-Aspartic Acid	133.0375	367.0958	5.16	0.0033	0.48
24	365.1186	5.68	4.70	HMDB00725	Trans-4-Hydroxyl-L-Proline	131.0582	365.1166	5.17	0.0020	0.47
25	365.1187	5.86	4.87	HMDB00725	Trans-4-Hydroxyl-L-Proline	131.0582	365.1166	5.17	0.0021	0.30
26	365.1183	6.06	5.06	HMDB00725	Trans-4-Hydroxyl-L-Proline	131.0582	365.1166	5.17	0.0017	0.11
27	381.1125	6.12	5.12	HMDB00148	L-Glutamic Acid	147.0532	381.1115	5.05	0.0010	0.07
28	365.1175	6.25	5.24	HMDB00725	Trans-4-Hydroxyl-L-Proline	131.0582	365.1166	5.17	0.0009	0.07
29	353.1176	6.31	5.30	HMDB00167	L-Threonine	119.0582	353.1166	5.79	0.0010	0.49
30	353.1178	6.62	5.59	HMDB00167	L-Threonine	119.0582	353.1166	5.79	0.0012	0.20
31	353.1176	6.92	5.94	HMDB00167	L-Threonine	119.0582	353.1166	5.79	0.0010	0.15
32	295.1116	7.02	6.09	HMDB00149	Ethanolamine	61.0528	295.1111	6	0.0005	0.09
33	395.1274	7.07	6.18	HMDB00510	Aminoadipic acid	161.0688	395.1271	5.97	0.0003	0.21
34	309.0913	7.14	6.29	HMDB00123	Glycine	75.0320	309.0903	6.59	0.0010	0.30
35	323.1066	7.54	6.94	HMDB00056	Beta-Alanine	89.0477	323.1060	7.24	0.0006	0.30
36	323.1069	7.91	7.55	HMDB00056	Beta-Alanine	89.0477	323.1060	7.24	0.0009	0.31
37	323.1069	7.91	7.55	HMDB00161	L-Alanine	89.0477	323.1060	7.57	0.0009	0.02
38	323.1073	8.36	7.97	HMDB00161	L-Alanine	89.0477	323.1060	7.57	0.0013	0.40
39	337.1217	8.42	8.03	HMDB00112	Gamma-Aminobutyric acid	103.0633	337.1216	7.79	0.0001	0.24

#	Input mass	Input RT(min)	Calibrated RT (min)	HMDB.No.	Name	Accurate mass	mz_light	library RT	Mass error (Da)	RT error (min)
40	337.1243	8.62	8.21	HMDB00112	Gamma-Aminobutyric acid	103.0633	337.1216	7.79	0.0027	0.42
41	337.1243	8.62	8.21	HMDB03911	3-Aminoisobutanoic acid	103.0633	337.1216	8.67	0.0027	0.46
42	351.1377	8.81	8.39	HMDB03355	5-Aminopentanoic acid	117.0790	351.1373	8.68	0.0004	0.29
43	363.1018	9.66	9.16	HMDB00148	L-Glutamic Acid - H2O	147.0532	363.1009	9.46	0.0009	0.30
44	337.1234	9.78	9.27	HMDB00452	L-Alpha-aminobutyric acid	103.0633	337.1216	9.13	0.0018	0.14
45	337.1234	9.78	9.27	HMDB00650	D-Alpha-aminobutyric acid	103.0633	337.1216	9.23	0.0018	0.04
46	337.1234	9.78	9.27	HMDB01906	2-Aminoisobutyric acid	103.0633	337.1216	8.91	0.0018	0.36
47	370.0991	9.88	9.36	HMDB00157	Hypoxanthine - Isomer	136.0385	370.0968	9.65	0.0023	0.29
48	363.1019	9.91	9.39	HMDB00148	L-Glutamic Acid - H2O	147.0532	363.1009	9.46	0.0010	0.07
49	337.1233	9.96	9.43	HMDB00452	L-Alpha-aminobutyric acid	103.0633	337.1216	9.13	0.0017	0.30
50	337.1233	9.96	9.43	HMDB00650	D-Alpha-aminobutyric acid	103.0633	337.1216	9.23	0.0017	0.20
51	337.1216	10.12	9.58	HMDB00452	L-Alpha-aminobutyric acid	103.0633	337.1216	9.13	0.0000	0.45
52	337.1216	10.12	9.58	HMDB00650	D-Alpha-aminobutyric acid	103.0633	337.1216	9.23	0.0000	0.35
53	399.1235	10.48	9.90	HMDB00897	7-Methylguanine	165.0651	399.1234	10.32	0.0001	0.42
54	399.1235	10.48	9.90	HMDB03282	1-Methylguanine	165.0651	399.1234	9.57	0.0001	0.33
55	349.1229	10.64	10.05	HMDB00162	L-Proline	115.0633	349.1216	10.18	0.0013	0.13
56	349.1226	10.99	10.35	HMDB00162	L-Proline	115.0633	349.1216	10.18	0.0010	0.17
57	422.1703	11.29	10.59	HMDB28844	Glycyl-Isoleucine	188.1161	422.1744	10.78	0.0041	0.19
58	351.1380	11.53	10.78	HMDB00883	L-Valine	117.0790	351.1373	10.81	0.0007	0.03
59	383.1107	11.70	10.92	HMDB00696	L-Methionine	149.0510	383.1094	10.89	0.0013	0.03
60	346.0862	11.73	10.95	HMDB00300	Uracil	112.0273	346.0856	11.34	0.0006	0.39

#	Input mass	Input RT(min)	Calibrated RT (min)	HMDB.No.	Name	Accurate mass	mz_light	library RT	Mass error (Da)	RT error (min)
61	351.1381	11.76	10.98	HMDB00883	L-Valine	117.0790	351.1373	10.81	0.0008	0.17
62	422.1734	12.30	11.54	HMDB00759	Glycyl-L-Leucine	188.1161	422.1744	11.22	0.0010	0.32
63	438.1492	12.46	11.71	HMDB00929	L-Tryptophan	204.0899	438.1482	11.44	0.0010	0.27
64	456.1571	12.73	12.00	HMDB28848	Glycyl-Phenylalanine	222.1004	456.1588	11.65	0.0017	0.35
65	383.1296	13.29	12.59	HMDB02099	6-Methyladenine	149.0701	383.1285	12.22	0.0011	0.37
66	383.1296	13.29	12.59	HMDB02099	6-Methyladenine - Isomer	149.0701	383.1285	12.74	0.0011	0.15
67	399.1387	13.46	12.77	HMDB00159	L-Phenylalanine	165.0790	399.1373	12.74	0.0014	0.03
68	365.1540	13.69	12.96	HMDB00172	L-Isoleucine	131.0946	365.1529	13.06	0.0011	0.10
69	365.1540	13.69	12.96	HMDB00557	L-Alloisoleucine	131.0946	365.1529	13.2	0.0011	0.24
70	365.1540	13.69	12.96	HMDB00687	L-leucine	131.0946	365.1529	13.36	0.0011	0.40
71	365.1538	13.95	13.18	HMDB00172	L-Isoleucine	131.0946	365.1529	13.06	0.0009	0.12
72	365.1538	13.95	13.18	HMDB00557	L-Alloisoleucine	131.0946	365.1529	13.2	0.0009	0.02
73	365.1538	13.95	13.18	HMDB00687	L-leucine	131.0946	365.1529	13.36	0.0009	0.18
74	364.6260	14.16	13.36	HMDB04987	Alpha-Aspartyl-lysine	261.1325	364.6246	13.61	0.0014	0.25
75	365.1495	14.24	13.42	HMDB00172	L-Isoleucine	131.0946	365.1529	13.06	0.0034	0.36
76	365.1495	14.24	13.42	HMDB00557	L-Alloisoleucine	131.0946	365.1529	13.2	0.0034	0.22
77	365.1495	14.24	13.42	HMDB00687	L-leucine	131.0946	365.1529	13.36	0.0034	0.06
78	345.0932	14.65	13.77	HMDB00099	L-Cystathionine	222.0674	345.0920	13.34	0.0012	0.43
79	345.0932	14.65	13.77	HMDB00099	L-Cystathionine - Isomer	222.0674	345.0920	13.69	0.0012	0.08
80	345.0932	14.65	13.77	HMDB00455	Allocystathionine	222.0674	345.0920	13.33	0.0012	0.44
81	345.0932	14.65	13.77	HMDB00455	Allocystathionine - Isomer	222.0674	345.0920	13.61	0.0012	0.16

#	Input mass	Input RT(min)	Calibrated RT (min)	HMDB.No.	Name	Accurate mass	mz_light	library RT	Mass error (Da)	RT error (min)
82	416.1171	14.80	13.90	HMDB00755	Hydroxyphenyllactici acid	182.0579	416.1162	14.39	0.0009	0.49
83	354.0713	15.05	14.11	HMDB00192	L-Cystine	240.0238	354.0702	14.11	0.0011	0.00
84	300.1039	16.35	16.29	HMDB00214	Ornithine	132.0899	300.1033	16.58	0.0006	0.29
85	307.1128	17.06	17.47	HMDB00182	L-Lysine	146.1055	307.1111	17.47	0.0017	0.00
86	389.1281	17.77	18.56	HMDB00177	L-Histidine	155.0695	389.1278	18.09	0.0003	0.47
87	324.5968	20.44	22.66	HMDB00158	L-Tyrosine	181.0739	324.5953	22.65	0.0015	0.01
88	324.5968	20.44	22.66	HMDB06050	o-Tyrosine	181.0739	324.5953	22.38	0.0015	0.28
89	289.0777	24.19	26.41	HMDB00957	pyrocatechol	110.0368	289.0767	26.7	0.0010	0.29

Table S1B. Identification of cellular metabolites by searching the accurate mass of the peak pairs against the HMDB library.

#	RT (s)	mz_light	mz_heavy	mz	distance	int_light	nCharge	nTag	Possible hits
1	128.55	375.07841	377.08489	141.02009	2.00648	1090000	1	1	1
2	130.55	367.09644	369.10373	133.03812	2.00730	1100000	1	1	3
3	135.60	449.11410	451.11910	215.05578	2.00500	29175	1	1	1
4	135.83	381.11109	383.11661	147.05277	2.00552	14850	1	1	6
5	136.16	366.11233	368.11887	132.05401	2.00654	3630000	1	1	4
6	151.91	380.12827	382.13458	146.06995	2.00631	5385000	1	1	4
7	152.99	280.10057	282.10707	46.04225	2.00650	533125	1	1	1
8	154.60	449.11495	451.12088	215.05663	2.00594	295375	1	1	1
9	167.81	449.11502	451.12073	215.05670	2.00571	358969	1	1	1
10	182.60	366.11259	368.11900	132.05427	2.00641	4930000	1	1	4
11	184.50	389.12732	391.13404	155.06900	2.00672	18850	1	1	1
12	185.26	381.11133	383.11619	147.05301	2.00485	30650	1	1	6
13	197.89	389.12769	391.13316	155.06937	2.00546	14900	1	1	1
14	200.90	359.07348	361.07845	125.01516	2.00497	150650	1	1	1
15	211.31	389.12757	391.13309	155.06925	2.00552	23100	1	1	1
16	212.64	380.12853	382.13487	146.07021	2.00634	7150000	1	1	4
17	214.39	517.15048	519.15673	283.09216	2.00626	57238	1	1	2
18	215.35	359.07352	361.07922	125.01520	2.00569	257066	1	1	1
19	220.65	339.10168	341.10819	105.04336	2.00652	947000	1	1	2
20	227.01	517.15056	519.15586	283.09224	2.00530	40500	1	1	2
21	230.26	365.11736	367.12365	131.05904	2.00628	3980000	1	1	8

#	RT (s)	mz_light	mz_heavy	mz	distance	int_light	nCharge	nTag	Possible hits
22	239.27	517.15025	519.15680	283.09193	2.00655	123781	1	1	2
23	241.67	408.17093	410.17733	174.11261	2.00640	422000	1	1	2
24	243.67	339.10156	341.10796	105.04324	2.00640	716000	1	1	2
25	245.84	293.10647	295.11258	59.04815	2.00611	36719	1	1	1
26	245.96	380.12882	382.13501	146.07050	2.00619	1151250	1	1	4
27	251.50	403.14269	405.14879	169.08437	2.00611	18700	1	1	2
28	252.68	485.15814	487.16431	251.09982	2.00618	10600	1	1	3
29	253.98	397.12472	399.13073	163.06640	2.00602	70800	1	1	1
30	259.28	381.11129	383.11740	147.05297	2.00611	98400	1	1	6
31	261.28	424.11749	426.12373	190.05917	2.00624	12200	1	1	1
32	262.06	408.17053	410.17718	174.11221	2.00664	910375	1	1	2
33	262.60	414.12169	416.12685	180.06337	2.00516	14375	1	1	17
34	263.63	509.16934	511.17527	275.11102	2.00592	10886	1	1	2
35	267.30	366.11246	368.11905	132.05414	2.00658	2732500	1	1	4
36	270.46	502.13862	504.14406	268.08030	2.00544	48378	1	1	4
37	274.87	414.12151	416.12753	180.06319	2.00602	25619	1	1	17
38	275.78	501.11490	505.12821	34.00556	4.01330	8610	1	2	1
39	278.70	365.11775	367.12414	131.05943	2.00639	5270000	1	1	8
40	288.82	502.13914	504.14539	268.08082	2.00625	67813	1	1	4
41	294.79	380.12789	382.13457	146.06957	2.00668	8270000	1	1	4
42	303.29	422.20971	424.21525	188.15139	2.00555	12800	1	1	1
43	307.52	367.09617	369.10188	133.03785	2.00571	73800	1	1	3
44	308.56	399.10441	401.11003	165.04609	2.00562	87138	1	1	1
45	313.95	353.11661	355.12308	119.05829	2.00647	17095	1	1	3

#	RT (s)	mz_light	mz_heavy	mz	distance	int_light	nCharge	nTag	Possible hits
46	315.35	422.20975	424.21408	188.15143	2.00433	18800	1	1	1
47	323.31	515.17349	517.17615	281.11517	2.00267	16400	1	1	4
48	324.66	399.10460	401.11050	165.04628	2.00591	108188	1	1	1
49	328.26	353.11570	355.12276	119.05738	2.00706	29640	1	1	3
50	329.51	436.20105	438.20708	202.14273	2.00603	152100	1	1	2
51	334.98	339.10164	341.10806	105.04332	2.00642	4457328	1	1	2
52	336.70	381.11222	383.11861	147.05390	2.00639	4248125	1	1	6
53	341.05	485.15870	487.16574	251.10038	2.00704	5730	1	1	3
54	342.77	501.15562	503.16298	267.09730	2.00736	757001	1	1	3
55	348.06	399.10454	401.11017	165.04622	2.00563	88742	1	1	1
56	348.18	436.20196	438.20781	202.14364	2.00585	303438	1	1	2
57	356.79	309.09095	311.09723	75.03263	2.00628	4960000	1	1	1
58	357.74	403.14387	405.14969	169.08555	2.00581	281438	1	1	2
59	367.31	381.11247	383.11880	147.05415	2.00633	5609727	1	1	6
60	374.81	365.11747	367.12385	131.05915	2.00639	4229414	1	1	8
61	378.65	353.11761	355.12394	119.05929	2.00633	2992813	1	1	3
62	381.46	348.10231	350.10893	114.04399	2.00662	725734	1	1	2
63	388.10	462.16913	464.17576	228.11081	2.00663	12300	1	1	1
64	388.72	519.19061	521.19662	285.13229	2.00600	22688	1	1	1
65	397.93	348.10208	350.10861	114.04376	2.00653	541836	1	1	2
66	399.93	379.13280	381.13921	145.07448	2.00641	215625	1	1	6
67	402.54	422.18553	424.19106	188.12721	2.00553	11240	1	1	1
68	407.85	387.12216	389.12880	153.06384	2.00664	26000	1	1	1
69	408.69	519.19132	521.19627	285.13300	2.00495	21717	1	1	1

#	RT (s)	mz_light	mz_heavy	mz	distance	int_light	nCharge	nTag	Possible hits
70	415.48	353.11755	355.12423	119.05923	2.00668	2729063	1	1	3
71	421.02	295.11164	297.11822	61.05332	2.00658	5788438	1	1	1
72	421.38	462.17009	464.17678	228.11177	2.00668	296900	1	1	1
73	424.37	395.12736	397.13316	161.06904	2.00580	20125	1	1	1
74	424.86	323.10599	325.11256	89.04767	2.00657	39700	1	1	4
75	445.30	362.11810	364.12644	128.05978	2.00834	1001738	1	1	1
76	447.57	462.17023	464.17701	228.11191	2.00678	545286	1	1	1
77	449.16	279.07998	281.08641	45.02166	2.00643	57400	1	1	1
78	452.34	323.10658	325.11269	89.04826	2.00611	208720	1	1	4
79	453.95	350.15306	352.15991	116.09474	2.00684	269000	1	1	1
80	455.15	422.18630	424.19207	188.12798	2.00576	216875	1	1	1
81	462.69	348.10128	350.10738	114.04296	2.00610	108031	1	1	2
82	466.48	394.14419	396.15015	160.08587	2.00596	555063	1	1	1
83	483.12	351.13712	353.14330	117.07880	2.00618	30788	1	1	5
84	493.83	483.14478	485.14973	249.08646	2.00496	20163	1	1	2
85	504.20	406.14277	408.14745	172.08445	2.00468	28370	1	1	2
86	505.02	337.12174	339.12804	103.06342	2.00631	53450	1	1	8
87	508.30	353.11728	355.12351	119.05896	2.00623	416250	1	1	3
88	511.82	531.14922	533.15339	297.09090	2.00418	12890	1	1	1
89	516.31	395.12676	397.13366	161.06844	2.00690	13638	1	1	1
90	526.08	406.14310	408.14897	172.08478	2.00587	42838	1	1	2
91	526.29	408.15879	410.16518	174.10047	2.00639	85094	1	1	1
92	528.64	351.13765	353.14416	117.07933	2.00651	221750	1	1	5
93	530.33	381.11275	383.11933	147.05443	2.00658	1034250	1	1	6

#	RT (s)	mz_light	mz_heavy	mz	distance	int_light	nCharge	nTag	Possible hits
94	532.98	363.10161	365.10799	129.04329	2.00637	4520000	1	1	5
95	535.65	517.14990	519.15657	283.09158	2.00667	18878	1	1	2
96	542.90	478.12954	480.13613	244.07122	2.00659	2737188	1	1	3
97	543.40	366.10158	368.10735	132.04326	2.00578	128463	1	1	7
98	547.62	457.08936	459.09627	446.06208	2.00691	26747	2	2	1
99	547.71	502.13779	504.14503	268.07947	2.00724	16053	1	1	4
100	547.94	385.10861	387.11500	151.05029	2.00639	732813	1	1	3
101	553.31	344.10654	346.11273	110.04822	2.00619	56450	1	1	1
102	556.07	363.10205	365.10843	129.04373	2.00638	6634063	1	1	5
103	558.30	395.12784	397.13457	161.06952	2.00672	7980000	1	1	1
104	561.38	362.11731	364.12320	128.05899	2.00589	138625	1	1	1
105	562.51	457.08994	459.09592	446.06323	2.00598	11015	2	2	1
106	565.43	309.12698	311.13281	75.06866	2.00583	35750	1	1	2
107	574.29	321.09117	323.09805	87.03285	2.00688	24000	1	1	1
108	579.77	363.10184	365.10809	129.04352	2.00625	4109891	1	1	5
109	588.15	436.20103	438.20623	202.14271	2.00520	94138	1	1	2
110	592.53	453.16876	455.17427	219.11044	2.00551	11355	1	1	2
111	594.61	363.10185	365.10838	129.04353	2.00652	6160379	1	1	5
112	595.81	481.08373	483.08967	247.02541	2.00594	11970	1	1	1
113	603.78	460.11871	462.12532	226.06039	2.00661	2648125	1	1	1
114	607.40	337.12161	339.12866	103.06329	2.00705	30138	1	1	8
115	608.53	379.13319	381.13957	145.07487	2.00638	4882500	1	1	6
116	613.48	366.10172	368.10709	132.04340	2.00537	36813	1	1	7
117	618.83	398.12742	400.13418	164.06910	2.00676	15704	1	1	6

#	RT (s)	mz_light	mz_heavy	mz	distance	int_light	nCharge	nTag	Possible hits
118	625.50	321.12682	323.13226	87.06850	2.00544	61700	1	1	1
119	628.59	399.12352	401.13031	165.06520	2.00679	28125	1	1	4
120	634.51	523.15941	525.16617	289.10109	2.00676	10650	1	1	1
121	637.92	409.14260	411.14805	175.08428	2.00545	14850	1	1	1
122	650.67	370.09800	372.10423	136.03968	2.00623	7773750	1	1	1
123	651.38	428.13809	430.14467	194.07977	2.00658	6630	1	1	1
124	659.53	349.12263	351.12898	115.06431	2.00635	11411250	1	1	2
125	661.17	477.16393	479.16971	243.10561	2.00578	2880000	1	1	1
126	661.65	629.21084	633.22356	162.10150	4.01272	46294	1	2	1
127	668.38	513.11465	517.13032	46.00531	4.01567	12400	1	2	1
128	680.64	380.16324	382.16945	146.10492	2.00621	23225	1	1	4
129	681.13	447.10422	449.10983	426.09179	2.00562	97300	2	2	1
130	681.48	408.15890	410.16512	174.10058	2.00622	147438	1	1	1
131	691.52	351.13804	353.14453	117.07972	2.00649	4883750	1	1	5
132	694.42	792.31510	794.32160	558.25678	2.00650	38200	1	1	1
133	696.70	337.12183	339.12738	103.06351	2.00555	71066	1	1	8
134	701.98	383.11071	385.11669	149.05239	2.00598	3057500	1	1	1
135	703.73	346.08617	348.09312	112.02785	2.00696	589969	1	1	1
136	705.57	351.13814	353.14451	117.07982	2.00637	5506875	1	1	5
137	713.83	321.09108	323.09733	87.03276	2.00625	99513	1	1	1
138	722.45	660.14851	662.15682	426.09019	2.00831	6610	1	1	1
139	727.18	447.10405	449.11097	426.09146	2.00692	877000	2	2	1
140	728.14	377.10763	379.11261	286.09863	2.00498	6090	2	2	1
141	734.50	409.14398	411.15035	175.08566	2.00638	834750	1	1	1

#	RT (s)	mz_light	mz_heavy	mz	distance	int_light	nCharge	nTag	Possible hits
142	736.07	508.18198	510.18851	274.12366	2.00652	139066	1	1	1
143	737.72	422.17337	424.18035	188.11505	2.00698	27056	1	1	3
144	740.86	395.12823	397.13450	161.06991	2.00627	423375	1	1	1
145	741.29	447.10423	449.11084	426.09182	2.00661	337000	2	2	1
146	747.39	438.14918	440.15526	204.09086	2.00608	369203	1	1	1
147	748.48	629.21024	633.22259	162.10090	4.01235	28744	1	2	1
148	754.58	508.18119	510.18854	274.12287	2.00735	16000	1	1	1
149	755.28	400.08638	402.09188	166.02806	2.00550	44934	1	1	3
150	766.89	319.11190	321.11744	85.05358	2.00553	112100	1	1	1
151	778.61	409.14393	411.15026	175.08561	2.00633	5070000	1	1	1
152	784.41	321.11600	323.12256	174.11536	2.00656	726929	2	2	1
153	784.70	426.12069	428.12700	192.06237	2.00631	30900	1	1	1
154	792.72	448.19043	450.19675	214.13211	2.00632	14950	1	1	1
155	797.27	383.12959	385.13434	149.07127	2.00475	26300	1	1	4
156	802.46	360.10231	362.11009	126.04399	2.00778	1398938	1	1	2
157	805.99	371.63317	373.64010	275.14970	2.00693	24234	2	2	2
158	807.82	399.13874	401.14507	165.08042	2.00633	6773984	1	1	4
159	821.19	365.15396	367.16031	131.09564	2.00635	7231250	1	1	6
160	829.88	377.11666	379.12301	143.05834	2.00635	101000	1	1	1
161	837.18	365.15381	367.16020	131.09549	2.00640	11103628	1	1	6
162	861.13	307.11155	309.11857	73.05323	2.00702	486000	1	1	3
163	862.62	397.12573	399.13122	163.06741	2.00550	15900	1	1	1
164	862.94	612.26765	616.28052	145.15831	4.01287	55600	1	2	1
165	867.32	597.11651	599.12135	363.05819	2.00484	37600	1	1	2

#	RT (s)	mz_light	mz_heavy	mz	distance	int_light	nCharge	nTag	Possible hits
166	867.40	626.28276	630.29547	159.17342	4.01272	6790	1	2	1
167	867.75	322.07429	324.08036	88.01597	2.00607	19650	1	1	2
168	871.61	300.08643	302.09315	132.05623	2.00671	9500	2	2	1
169	872.61	309.58359	311.58829	151.05055	2.00469	13000	2	2	3
170	878.84	345.09318	347.10010	222.06971	2.00692	37000	2	2	1
171	883.24	555.15155	557.15731	321.09323	2.00576	231500	1	1	1
172	888.22	416.11709	418.12230	182.05877	2.00521	12350	1	1	6
173	892.63	612.26689	616.27972	145.15755	4.01283	15275	1	2	1
174	892.89	629.21068	633.22377	162.10134	4.01309	43625	1	2	1
175	893.58	462.20597	464.21145	228.14765	2.00548	21413	1	1	2
176	902.75	354.07128	356.07781	240.02593	2.00653	182000	2	2	1
177	905.49	387.10050	389.10642	153.04218	2.00592	48838	1	1	2
178	905.98	363.13816	365.14459	129.07984	2.00643	3792500	1	1	4
179	911.64	612.26781	616.28023	145.15847	4.01241	50188	1	2	1
180	927.44	502.09665	504.10388	268.03833	2.00723	19863	1	1	1
181	928.09	496.18981	498.19654	262.13149	2.00673	18156	1	1	2
182	929.06	393.14769	395.15427	159.08937	2.00658	33403	1	1	6
183	931.97	431.12581	433.13110	197.06749	2.00529	20250	1	1	2
184	938.40	411.10457	413.11191	177.04625	2.00734	8470	1	1	1
185	940.82	502.09687	504.10423	268.03855	2.00736	17363	1	1	1
186	948.03	612.26605	616.27923	145.15671	4.01318	11100	1	2	1
187	949.91	351.13795	353.14454	117.07963	2.00659	612125	1	1	5
188	957.72	350.64614	352.65257	233.17564	2.00643	42431	2	2	1
189	966.82	501.11493	505.12830	34.00559	4.01336	60559	1	2	1

#	RT (s)	mz_light	mz_heavy	mz	distance	int_light	nCharge	nTag	Possible hits
190	966.88	368.09964	370.10638	268.08265	2.00673	61681	2	2	3
191	967.86	413.15322	415.16196	179.09490	2.00874	26650	1	1	2
192	971.73	311.59287	313.59969	155.06910	2.00682	77994	2	2	1
193	975.74	371.63248	373.63850	275.14832	2.00602	10238	2	2	2
194	977.06	342.63030	344.63687	217.14397	2.00657	14375	2	2	1
195	980.38	346.09891	348.10579	224.08118	2.00688	10500	2	2	3
196	980.97	300.10391	302.11049	132.09119	2.00657	40300	2	2	2
197	987.45	494.68875	496.69480	521.26086	2.00605	6890	2	2	1
198	1004.84	407.16383	409.17006	173.10551	2.00623	44875	1	1	4
199	1005.29	379.16895	381.17392	145.11063	2.00498	26941	1	1	1
200	1006.92	372.09032	374.09661	138.03200	2.00629	106109	1	1	4
201	1014.64	441.14752	443.15374	207.08920	2.00623	35781	1	1	3
202	1016.48	427.13226	429.13809	193.07394	2.00583	18200	1	1	3
203	1019.82	312.08485	314.09115	156.05306	2.00630	839000	2	2	3
204	1026.08	457.14136	459.14810	223.08304	2.00675	15000	1	1	1
205	1028.54	407.16419	409.17041	173.10587	2.00622	146750	1	1	4
206	1034.98	413.15418	415.16000	179.09586	2.00581	573781	1	1	2
207	1037.61	347.11234	349.11957	226.10804	2.00724	13122	2	2	1
208	1039.57	367.60760	369.61494	267.09855	2.00734	318000	2	2	2
209	1042.57	356.09962	358.10434	244.08259	2.00472	7290	2	2	1
210	1047.28	375.60520	377.61174	283.09376	2.00654	88753	2	2	2
211	1048.79	366.10141	368.10676	132.04309	2.00535	34500	1	1	7
212	1063.17	379.16995	381.17633	145.11163	2.00639	2143750	1	1	1
213	1066.14	389.12813	391.13395	155.06981	2.00582	90838	1	1	1

#	RT (s)	mz_light	mz_heavy	mz	distance	int_light	nCharge	nTag	Possible hits
214	1067.29	312.59492	314.60143	157.07320	2.00651	563481	2	2	2
215	1070.56	339.09654	341.10372	210.07643	2.00718	11269	2	2	1
216	1078.28	356.09410	358.10140	244.07156	2.00729	28069	2	2	3
217	1086.58	382.10946	384.11536	296.10227	2.00590	7533	2	2	1
218	1093.63	389.11939	391.12549	310.12215	2.00609	6790	2	2	1
219	1097.31	487.15238	489.15885	253.09406	2.00647	16150	1	1	1
220	1098.67	541.14297	543.14710	307.08465	2.00413	9710	1	1	1
221	1100.11	356.09358	358.10081	244.07053	2.00723	40294	2	2	2
222	1100.38	298.08804	300.09521	128.05945	2.00717	538375	2	2	1
223	1109.55	466.17945	468.18461	232.12113	2.00516	11031	1	1	1
224	1110.73	356.09422	358.10090	244.07180	2.00668	248125	2	2	3
225	1117.69	328.13365	330.14152	188.15066	2.00786	9650	2	2	1
226	1124.66	423.14240	425.14653	189.08408	2.00414	8380	1	1	1
227	1130.88	348.11413	350.12264	228.11163	2.00850	32100	2	2	1
228	1134.33	328.11714	330.12356	188.11764	2.00643	57016	2	2	3
229	1140.53	386.10673	388.11380	152.04841	2.00707	8720	1	1	9
230	1143.63	528.16246	532.17549	61.05312	4.01303	52644	1	2	1
231	1145.62	321.10926	323.11535	174.10188	2.00609	35200	2	2	1
232	1153.10	292.10594	294.11229	116.09524	2.00635	41381	2	2	1
233	1153.63	379.16944	381.17540	145.11112	2.00596	379156	1	1	1
234	1183.58	693.16709	697.18175	226.05775	4.01466	12700	1	2	1
235	1187.80	346.08548	348.09120	112.02716	2.00572	32000	1	1	1
236	1196.40	427.16945	429.17523	193.11113	2.00578	371219	1	1	2
237	1199.57	335.16715	337.17351	202.21767	2.00635	20450	2	2	1

#	RT (s)	mz_light	mz_heavy	mz	distance	int_light	nCharge	nTag	Possible hits
238	1215.62	403.14390	405.15022	169.08558	2.00633	11850	1	1	2
239	1225.89	325.09802	327.10475	182.07941	2.00673	661500	2	2	4
240	1227.31	414.12389	416.13048	180.06557	2.00658	35853	1	1	3
241	1229.02	373.08570	375.09071	139.02738	2.00501	15050	1	1	2
242	1229.78	386.10619	388.11146	152.04787	2.00527	26500	1	1	9
243	1263.07	437.17342	439.18081	203.11510	2.00739	30275	1	1	1
244	1269.01	373.12257	375.12872	278.12850	2.00615	17950	2	2	3
245	1284.23	598.10105	600.10475	364.04273	2.00370	11340	1	1	1
246	1298.78	302.07811	304.08615	136.03958	2.00803	6970	2	2	1
247	1306.20	435.19433	437.20016	201.13601	2.00583	23763	1	1	1
248	1308.32	354.11613	356.12235	120.05781	2.00622	190583	1	1	2
249	1320.01	354.11670	356.12358	120.05838	2.00689	821875	1	1	2
250	1325.81	338.59329	340.60018	209.06993	2.00689	24400	2	2	1
251	1331.07	345.60138	347.60789	223.08612	2.00652	34638	2	2	1
252	1352.47	297.59040	299.59735	127.06417	2.00694	57313	2	2	2
253	1353.41	577.15698	581.16941	110.04764	4.01243	21538	1	2	1
254	1362.27	308.58151	310.58939	149.04638	2.00788	29494	2	2	1
255	1363.36	313.59304	315.59988	159.06944	2.00684	37797	2	2	1
256	1367.48	577.15696	581.17035	110.04762	4.01339	27553	1	2	1
257	1375.96	344.10595	346.11224	110.04763	2.00629	33750	1	1	1
258	1378.47	328.13459	330.14174	188.15255	2.00715	9450	2	2	1
259	1385.41	302.07785	304.08488	136.03906	2.00703	36913	2	2	1
260	1394.42	612.26697	616.28060	145.15763	4.01364	55681	1	2	1
261	1425.70	323.60645	325.61317	179.09626	2.00672	12753	2	2	2

#	RT (s)	mz_light	mz_heavy	mz	distance	int_light	nCharge	nTag	Possible hits
262	1486.25	669.32514	673.33790	202.21580	4.01276	15800	1	2	1
263	1486.89	570.29100	572.29316	336.23268	2.00216	11450	1	1	22
264	1493.26	685.32688	687.33352	451.26856	2.00664	11730	1	1	2
265	1499.84	320.06730	322.07331	172.01796	2.00601	1060000	2	2	1
266	1500.01	319.06653	321.07326	170.01643	2.00673	2623328	2	2	1
267	1500.28	403.06362	405.06748	169.00530	2.00387	41691	1	1	1
268	1506.67	584.30725	586.30957	350.24893	2.00232	9120	1	1	5
269	1513.80	735.34388	737.35017	501.28556	2.00629	94963	1	1	5
270	1523.31	711.34181	713.35179	477.28349	2.00998	7930	1	1	2
271	1532.61	761.35818	763.36785	527.29986	2.00967	13400	1	1	4
272	1541.38	470.23568	472.24189	236.17736	2.00621	69206	1	1	1
273	1549.90	533.33992	535.34661	299.28160	2.00669	57257	1	1	3
274	1566.31	535.35505	537.36188	301.29673	2.00683	21875	1	1	1
275	1574.10	489.31422	491.32056	255.25590	2.00634	104750	1	1	1
276	1580.99	515.32889	517.33456	281.27057	2.00567	7580	1	1	1
277	1602.43	687.34515	689.35050	453.28683	2.00535	6765	1	1	2
278	1619.19	713.35902	715.36405	479.30070	2.00503	21000	1	1	4
279	1631.72	713.35865	715.36492	479.30033	2.00626	19620	1	1	4
280	1689.44	671.34698	673.35294	437.28866	2.00596	7760	1	1	1
281	1707.69	671.34857	673.35182	437.29025	2.00325	7600	1	1	1

Table S1C. Identification of cellular metabolites by searching the accurate mass of the peak pairs against the EML library with one reaction in MCID.

#	RT (s)	mz_light	mz_heavy	mz	distance	int_light	nCharge	nTag	Possible hits
1	125.47	425.18512	427.19137	191.12680	2.00625	94200	1	1	2
2	126.55	426.16865	428.17552	192.11033	2.00687	15700	1	1	4
3	128.55	375.07841	377.08489	141.02009	2.00648	1090000	1	1	6
4	130.55	367.09644	369.10373	133.03812	2.00730	1100000	1	1	28
5	134.16	411.16900	413.17542	177.11068	2.00641	30500	1	1	4
6	135.60	449.11410	451.11910	215.05578	2.00500	29175	1	1	4
7	135.83	381.11109	383.11661	147.05277	2.00552	14850	1	1	51
8	136.16	366.11233	368.11887	132.05401	2.00654	3630000	1	1	12
9	150.91	675.24145	677.24905	441.18313	2.00760	11385	1	1	7
10	151.91	380.12827	382.13458	146.06995	2.00631	5385000	1	1	29
11	152.99	280.10057	282.10707	46.04225	2.00650	533125	1	1	20
12	154.60	449.11495	451.12088	215.05663	2.00594	295375	1	1	4
13	157.45	474.14296	476.15005	240.08464	2.00708	11725	1	1	25
14	166.46	675.24238	677.24879	441.18406	2.00640	30724	1	1	7
15	167.81	449.11502	451.12073	215.05670	2.00571	358969	1	1	4
16	170.33	474.14361	476.15037	240.08529	2.00676	13441	1	1	29
17	182.09	573.13205	575.13766	339.07373	2.00561	121653	1	1	4
18	182.60	366.11259	368.11900	132.05427	2.00641	4930000	1	1	12
19	182.93	589.12591	591.13139	355.06759	2.00548	13294	1	1	8
20	184.50	389.12732	391.13404	155.06900	2.00672	18850	1	1	14

#	RT (s)	mz_light	mz_heavy	mz	distance	int_light	nCharge	nTag	Possible hits
21	185.26	381.11133	383.11619	147.05301	2.00485	30650	1	1	51
22	187.53	410.17510	412.18165	176.11678	2.00655	8520	1	1	12
23	193.02	675.24287	677.24785	441.18455	2.00498	13244	1	1	7
24	197.89	389.12769	391.13316	155.06937	2.00546	14900	1	1	14
25	200.90	359.07348	361.07845	125.01516	2.00497	150650	1	1	5
26	201.63	535.17213	537.17810	301.11381	2.00597	17100	1	1	10
27	201.90	582.25895	584.26552	348.20063	2.00657	15100	1	1	1
28	206.70	675.24158	677.24828	441.18326	2.00670	13200	1	1	7
29	208.77	388.10798	390.11436	154.04966	2.00638	591813	1	1	11
30	211.31	389.12757	391.13309	155.06925	2.00552	23100	1	1	14
31	212.64	380.12853	382.13487	146.07021	2.00634	7150000	1	1	29
32	214.39	517.15048	519.15673	283.09216	2.00626	57238	1	1	38
33	215.35	359.07352	361.07922	125.01520	2.00569	257066	1	1	5
34	216.54	513.18919	515.19486	279.13087	2.00566	16439	1	1	15
35	218.29	675.24422	677.24892	441.18590	2.00470	17150	1	1	7
36	220.65	339.10168	341.10819	105.04336	2.00652	947000	1	1	21
37	223.22	389.09338	391.10004	155.03506	2.00666	185500	1	1	5
38	227.01	517.15056	519.15586	283.09224	2.00530	40500	1	1	39
39	230.26	365.11736	367.12365	131.05904	2.00628	3980000	1	1	60
40	231.94	715.24816	717.25234	481.18984	2.00418	7860	1	1	6
41	235.66	783.23635	785.24108	549.17803	2.00474	42800	1	1	2
42	239.27	517.15025	519.15680	283.09193	2.00655	123781	1	1	38
43	241.67	408.17093	410.17733	174.11261	2.00640	422000	1	1	6
44	243.67	339.10156	341.10796	105.04324	2.00640	716000	1	1	21

#	RT (s)	mz_light	mz_heavy	mz	distance	int_light	nCharge	nTag	Possible hits
45	245.84	293.10647	295.11258	59.04815	2.00611	36719	1	1	1
46	245.96	380.12882	382.13501	146.07050	2.00619	1151250	1	1	29
47	246.46	463.12992	465.13591	229.07160	2.00599	21056	1	1	8
48	251.50	403.14269	405.14879	169.08437	2.00611	18700	1	1	11
49	252.68	485.15814	487.16431	251.09982	2.00618	10600	1	1	39
50	253.98	397.12472	399.13073	163.06640	2.00602	70800	1	1	5
51	256.27	675.24271	677.24920	441.18439	2.00649	48894	1	1	7
52	257.36	380.13084	382.13712	146.07252	2.00627	1395000	1	1	10
53	258.35	513.18902	515.19378	279.13070	2.00476	9205	1	1	16
54	259.28	381.11129	383.11740	147.05297	2.00611	98400	1	1	51
55	261.28	424.11749	426.12373	190.05917	2.00624	12200	1	1	15
56	262.06	408.17053	410.17718	174.11221	2.00664	910375	1	1	6
57	262.21	493.17368	495.17912	259.11536	2.00544	11975	1	1	10
58	262.60	414.12169	416.12685	180.06337	2.00516	14375	1	1	87
59	263.63	509.16934	511.17527	275.11102	2.00592	10886	1	1	16
60	265.42	417.15810	419.16464	183.09978	2.00654	18738	1	1	8
61	267.30	366.11246	368.11905	132.05414	2.00658	2732500	1	1	12
62	267.96	587.14581	589.15073	353.08749	2.00492	7780	1	1	6
63	270.46	502.13862	504.14406	268.08030	2.00544	48378	1	1	23
64	270.70	535.18251	537.18613	301.12419	2.00362	11400	1	1	2
65	273.53	675.24242	677.24899	441.18410	2.00656	57500	1	1	7
66	274.20	657.23177	659.23769	423.17345	2.00592	14500	1	1	14
67	274.21	513.18572	515.19603	279.12740	2.01031	11050	1	1	3
68	274.87	414.12151	416.12753	180.06319	2.00602	25619	1	1	87

#	RT (s)	mz_light	mz_heavy	mz	distance	int_light	nCharge	nTag	Possible hits
69	275.23	489.13251	491.13872	255.07419	2.00621	89125	1	1	16
70	275.78	501.11490	505.12821	34.00556	4.01330	8610	1	2	8
71	277.53	417.15907	419.16418	183.10075	2.00510	38134	1	1	8
72	278.70	365.11775	367.12414	131.05943	2.00639	5270000	1	1	60
73	283.75	702.25257	704.25909	468.19425	2.00652	6130	1	1	5
74	287.55	408.17227	410.17845	174.11395	2.00618	1516654	1	1	3
75	288.82	502.13914	504.14539	268.08082	2.00625	67813	1	1	23
76	290.72	479.15977	481.16644	245.10145	2.00667	10735	1	1	14
77	294.79	380.12789	382.13457	146.06957	2.00668	8270000	1	1	29
78	297.76	702.25317	704.25987	468.19485	2.00670	14100	1	1	5
79	300.73	535.18142	537.18586	301.12310	2.00444	24900	1	1	2
80	303.29	422.20971	424.21525	188.15139	2.00555	12800	1	1	4
81	304.28	489.13277	491.13910	255.07445	2.00633	119188	1	1	16
82	305.88	513.18877	515.19398	279.13045	2.00521	21138	1	1	16
83	307.52	367.09617	369.10188	133.03785	2.00571	73800	1	1	28
84	308.56	399.10441	401.11003	165.04609	2.00562	87138	1	1	7
85	312.66	587.13034	589.13600	353.07202	2.00566	39200	1	1	6
86	313.95	353.11661	355.12308	119.05829	2.00647	17095	1	1	35
87	315.35	422.20975	424.21408	188.15143	2.00433	18800	1	1	4
88	316.47	372.14849	374.15487	138.09017	2.00638	18000	1	1	3
89	319.31	381.11415	383.11874	147.05583	2.00459	1200625	1	1	4
90	323.31	515.17349	517.17615	281.11517	2.00267	16400	1	1	32
91	324.58	436.15304	438.15853	202.09472	2.00549	15250	1	1	19
92	324.66	399.10460	401.11050	165.04628	2.00591	108188	1	1	7

#	RT (s)	mz_light	mz_heavy	mz	distance	int_light	nCharge	nTag	Possible hits
93	326.59	535.17258	537.17856	301.11426	2.00598	13145	1	1	10
94	326.75	499.17528	501.17960	265.11696	2.00433	22900	1	1	39
95	328.26	353.11570	355.12276	119.05738	2.00706	29640	1	1	35
96	328.76	388.10771	390.11345	154.04939	2.00574	60800	1	1	11
97	329.03	513.19053	515.19597	279.13221	2.00544	91972	1	1	15
98	329.51	436.20105	438.20708	202.14273	2.00603	152100	1	1	4
99	331.76	691.26982	693.27982	457.21150	2.01000	11200	1	1	8
100	331.76	731.21454	733.22323	497.15622	2.00869	6310	1	1	8
101	334.98	339.10164	341.10806	105.04332	2.00642	4457328	1	1	21
102	335.26	747.22248	749.22513	513.16416	2.00265	18500	1	1	7
103	336.70	381.11222	383.11861	147.05390	2.00639	4248125	1	1	55
104	338.70	769.20559	771.20556	535.14727	1.99997	15800	1	1	2
105	341.05	485.15870	487.16574	251.10038	2.00704	5730	1	1	39
106	341.33	436.15645	438.16104	202.09813	2.00459	18200	1	1	1
107	342.77	501.15562	503.16298	267.09730	2.00736	757001	1	1	50
108	348.06	399.10454	401.11017	165.04622	2.00563	88742	1	1	7
109	348.18	436.20196	438.20781	202.14364	2.00585	303438	1	1	4
110	353.42	691.27264	693.28047	457.21432	2.00784	11950	1	1	7
111	354.25	576.21113	578.21645	342.15281	2.00531	10695	1	1	11
112	356.79	309.09095	311.09723	75.03263	2.00628	4960000	1	1	21
113	357.74	403.14387	405.14969	169.08555	2.00581	281438	1	1	11
114	358.65	438.13543	440.13997	204.07711	2.00454	21425	1	1	5
115	364.88	587.12900	589.13489	353.07068	2.00589	42675	1	1	4
116	367.31	381.11247	383.11880	147.05415	2.00633	5609727	1	1	55

#	RT (s)	mz_light	mz_heavy	mz	distance	int_light	nCharge	nTag	Possible hits
117	372.45	449.25752	451.26373	215.19920	2.00621	10503	1	1	2
118	372.65	576.21203	578.21794	342.15371	2.00592	13803	1	1	11
119	374.81	365.11747	367.12385	131.05915	2.00639	4229414	1	1	60
120	378.65	353.11761	355.12394	119.05929	2.00633	2992813	1	1	35
121	379.30	292.11189	294.11810	58.05357	2.00621	263000	1	1	1
122	379.46	478.16426	480.16989	244.10594	2.00562	7360	1	1	15
123	381.46	348.10231	350.10893	114.04399	2.00662	725734	1	1	12
124	388.10	462.16913	464.17576	228.11081	2.00663	12300	1	1	8
125	388.42	417.15819	419.16465	183.09987	2.00646	29669	1	1	8
126	388.72	519.19061	521.19662	285.13229	2.00600	22688	1	1	9
127	389.47	290.56655	292.57175	113.01646	2.00520	15850	2	2	1
128	390.47	319.58780	321.59381	171.05896	2.00601	18225	2	2	1
129	393.56	555.15756	557.16224	321.09924	2.00468	27994	1	1	9
130	395.40	373.14270	375.14998	139.08438	2.00728	12800	1	1	2
131	397.93	348.10208	350.10861	114.04376	2.00653	541836	1	1	12
132	398.11	587.12937	589.13618	353.07105	2.00681	94400	1	1	4
133	399.69	290.56659	292.57278	113.01654	2.00619	10397	2	2	1
134	399.93	379.13280	381.13921	145.07448	2.00641	215625	1	1	54
135	402.54	422.18553	424.19106	188.12721	2.00553	11240	1	1	7
136	406.94	319.58814	321.59383	171.05964	2.00569	12450	2	2	1
137	407.85	387.12216	389.12880	153.06384	2.00664	26000	1	1	10
138	408.69	519.19132	521.19627	285.13300	2.00495	21717	1	1	10
139	413.02	647.24939	649.25565	413.19107	2.00625	8815	1	1	6
140	415.11	411.14062	413.14637	177.08230	2.00576	27588	1	1	6

#	RT (s)	mz_light	mz_heavy	mz	distance	int_light	nCharge	nTag	Possible hits
141	415.48	353.11755	355.12423	119.05923	2.00668	2729063	1	1	35
142	420.86	431.17413	433.17983	197.11581	2.00570	15038	1	1	3
143	421.02	295.11164	297.11822	61.05332	2.00658	5788438	1	1	15
144	421.38	462.17009	464.17678	228.11177	2.00668	296900	1	1	8
145	421.46	405.15862	407.16505	171.10030	2.00644	14600	1	1	6
146	421.87	673.26040	675.27058	439.20208	2.01018	23475	1	1	5
147	424.37	395.12736	397.13316	161.06904	2.00580	20125	1	1	48
148	424.86	323.10599	325.11256	89.04767	2.00657	39700	1	1	38
149	427.87	625.19888	627.20489	391.14056	2.00602	12600	1	1	21
150	429.28	363.14903	365.15467	129.09071	2.00563	298375	1	1	4
151	430.94	555.15792	557.16372	321.09960	2.00580	13672	1	1	8
152	433.15	491.19429	493.20221	257.13597	2.00792	16100	1	1	6
153	433.20	505.22178	507.22515	271.16346	2.00336	20065	1	1	1
154	433.40	479.23225	481.23903	245.17393	2.00678	245810	1	1	6
155	439.02	600.20052	602.20699	366.14220	2.00646	10503	1	1	11
156	445.30	362.11810	364.12644	128.05978	2.00834	1001738	1	1	16
157	447.57	462.17023	464.17701	228.11191	2.00678	545286	1	1	8
158	448.02	303.11583	305.12255	69.05751	2.00671	9495	1	1	2
159	448.90	477.18100	479.18642	243.12268	2.00541	33975	1	1	8
160	449.16	279.07998	281.08641	45.02166	2.00643	57400	1	1	8
161	450.73	334.12235	336.12878	100.06403	2.00643	374194	1	1	3
162	452.34	323.10658	325.11269	89.04826	2.00611	208720	1	1	38
163	453.89	587.12965	589.13567	353.07133	2.00602	102000	1	1	6
164	453.95	350.15306	352.15991	116.09474	2.00684	269000	1	1	9

#	RT (s)	mz_light	mz_heavy	mz	distance	int_light	nCharge	nTag	Possible hits
165	454.49	491.19474	493.20177	257.13642	2.00703	23984	1	1	6
166	455.15	422.18630	424.19207	188.12798	2.00576	216875	1	1	7
167	456.17	378.22080	380.22800	144.16248	2.00720	28700	1	1	2
168	456.67	436.15357	438.16042	202.09525	2.00685	11839	1	1	19
169	458.73	337.15906	339.16563	103.10074	2.00657	3258477	1	1	5
170	461.16	789.30865	791.31321	555.25033	2.00457	27581	1	1	3
171	462.18	787.30244	789.30771	553.24412	2.00528	66400	1	1	4
172	462.69	348.10128	350.10738	114.04296	2.00610	108031	1	1	12
173	466.48	394.14419	396.15015	160.08587	2.00596	555063	1	1	35
174	470.91	450.16884	452.17380	216.11052	2.00496	15625	1	1	15
175	471.48	600.20092	602.20808	366.14260	2.00716	17388	1	1	12
176	473.45	463.23755	465.24337	229.17923	2.00581	32647	1	1	2
177	474.92	539.21007	541.21466	305.15175	2.00459	9565	1	1	8
178	478.71	436.15412	438.15983	202.09580	2.00571	15388	1	1	19
179	479.43	436.22513	438.23001	202.16681	2.00488	10288	1	1	3
180	483.12	351.13712	353.14330	117.07880	2.00618	30788	1	1	38
181	493.83	483.14478	485.14973	249.08646	2.00496	20163	1	1	41
182	496.63	431.17472	433.18042	197.11640	2.00570	12825	1	1	3
183	500.00	413.11983	415.12594	179.06151	2.00611	37131	1	1	11
184	504.20	406.14277	408.14745	172.08445	2.00468	28370	1	1	9
185	505.02	337.12174	339.12804	103.06342	2.00631	53450	1	1	45
186	508.30	353.11728	355.12351	119.05896	2.00623	416250	1	1	35
187	509.37	361.19461	363.20096	127.13629	2.00635	96453	1	1	1
188	510.88	587.12257	589.13433	353.06425	2.01176	8330	1	1	28

#	RT (s)	mz_light	mz_heavy	mz	distance	int_light	nCharge	nTag	Possible hits
189	511.82	531.14922	533.15339	297.09090	2.00418	12890	1	1	10
190	512.08	478.16402	480.17014	244.10570	2.00611	19550	1	1	15
191	512.43	367.13185	369.13840	133.07353	2.00655	16581	1	1	40
192	514.37	392.16390	394.17012	158.10558	2.00622	30991	1	1	6
193	516.31	503.19628	505.20026	269.13796	2.00398	10105	1	1	8
194	516.31	395.12676	397.13366	161.06844	2.00690	13638	1	1	46
195	523.10	625.19991	627.20721	391.14159	2.00730	12050	1	1	22
196	523.74	476.22200	478.22800	242.16368	2.00600	20650	1	1	4
197	523.80	450.16974	452.17552	216.11142	2.00578	14538	1	1	15
198	526.08	406.14310	408.14897	172.08478	2.00587	42838	1	1	9
199	526.29	408.15879	410.16518	174.10047	2.00639	85094	1	1	35
200	528.64	351.13765	353.14416	117.07933	2.00651	221750	1	1	38
201	528.76	587.12950	589.13479	353.07118	2.00529	24994	1	1	6
202	530.33	381.11275	383.11933	147.05443	2.00658	1034250	1	1	55
203	532.04	503.19910	505.20247	269.14078	2.00337	12200	1	1	1
204	532.98	363.10161	365.10799	129.04329	2.00637	4520000	1	1	28
205	535.65	517.14990	519.15657	283.09158	2.00667	18878	1	1	38
206	539.26	450.17027	452.17599	216.11195	2.00573	21500	1	1	15
207	541.82	578.21176	580.21804	344.15344	2.00629	26750	1	1	1
208	542.90	478.12954	480.13613	244.07122	2.00659	2737188	1	1	31
209	543.40	366.10158	368.10735	132.04326	2.00578	128463	1	1	69
210	547.71	502.13779	504.14503	268.07947	2.00724	16053	1	1	20
211	547.86	535.17745	537.18634	301.11913	2.00889	14325	1	1	15
212	547.94	385.10861	387.11500	151.05029	2.00639	732813	1	1	11

#	RT (s)	mz_light	mz_heavy	mz	distance	int_light	nCharge	nTag	Possible hits
213	552.27	587.12946	589.13574	353.07114	2.00628	156000	1	1	4
214	553.31	344.10654	346.11273	110.04822	2.00619	56450	1	1	8
215	554.14	558.19607	560.20463	324.13775	2.00856	10245	1	1	5
216	555.42	367.13277	369.13812	133.07445	2.00535	16944	1	1	40
217	556.07	363.10205	365.10843	129.04373	2.00638	6634063	1	1	28
218	558.30	395.12784	397.13457	161.06952	2.00672	7980000	1	1	48
219	559.32	463.23679	465.24223	229.17847	2.00544	10650	1	1	2
220	560.51	513.18949	515.19453	279.13117	2.00504	11713	1	1	15
221	561.38	362.11731	364.12320	128.05899	2.00589	138625	1	1	16
222	563.01	732.33810	734.34289	498.27978	2.00480	17800	1	1	33
223	564.36	549.19295	551.19793	315.13463	2.00498	11139	1	1	13
224	565.43	309.12698	311.13281	75.06866	2.00583	35750	1	1	13
225	574.29	321.09117	323.09805	87.03285	2.00688	24000	1	1	18
226	579.10	438.16918	440.17402	204.11086	2.00484	8060	1	1	11
227	579.77	363.10184	365.10809	129.04352	2.00625	4109891	1	1	28
228	581.51	551.17596	553.18254	317.11764	2.00659	99375	1	1	3
229	584.85	476.18510	478.19128	242.12678	2.00618	141875	1	1	6
230	584.94	569.17431	571.17938	335.11599	2.00507	13688	1	1	9
231	585.03	578.21321	580.21622	344.15489	2.00300	11700	1	1	1
232	588.15	436.20103	438.20623	202.14271	2.00520	94138	1	1	4
233	592.53	453.16876	455.17427	219.11044	2.00551	11355	1	1	18
234	594.09	691.16617	695.17843	224.05683	4.01226	12338	1	2	2
235	594.61	363.10185	365.10838	129.04353	2.00652	6160379	1	1	28
236	595.81	481.08373	483.08967	247.02541	2.00594	11970	1	1	3

#	RT (s)	mz_light	mz_heavy	mz	distance	int_light	nCharge	nTag	Possible hits
237	596.07	478.20075	480.20758	244.14243	2.00682	25897	1	1	6
238	598.45	671.24212	673.24947	437.18380	2.00734	34500	1	1	2
239	599.55	376.17954	378.18633	142.12122	2.00679	29025	1	1	2
240	603.78	460.11871	462.12532	226.06039	2.00661	2648125	1	1	26
241	606.93	540.13514	542.14105	306.07682	2.00591	16914	1	1	4
242	607.40	337.12161	339.12866	103.06329	2.00705	30138	1	1	45
243	608.13	420.15869	422.16536	186.10037	2.00667	36350	1	1	15
244	608.53	379.13319	381.13957	145.07487	2.00638	4882500	1	1	54
245	613.48	366.10172	368.10709	132.04340	2.00537	36813	1	1	69
246	614.16	438.16979	440.17505	204.11147	2.00526	32606	1	1	11
247	614.43	346.08759	348.09384	224.05853	2.00625	12625	2	2	3
248	617.42	478.19988	480.20616	244.14156	2.00628	65075	1	1	6
249	618.81	334.12196	336.12847	100.06364	2.00651	39788	1	1	3
250	618.83	398.12742	400.13418	164.06910	2.00676	15704	1	1	74
251	621.05	550.19827	552.20489	316.13995	2.00662	214214	1	1	4
252	625.50	321.12682	323.13226	87.06850	2.00544	61700	1	1	29
253	625.72	571.20509	573.21510	337.14677	2.01001	25078	1	1	7
254	628.03	558.19572	560.20162	324.13740	2.00590	17055	1	1	5
255	628.59	399.12352	401.13031	165.06520	2.00679	28125	1	1	36
256	629.05	355.09357	357.10015	242.07050	2.00658	848267	2	2	11
257	630.08	628.22743	630.23068	394.16911	2.00324	14600	1	1	4
258	631.62	506.17530	508.18207	272.11698	2.00677	14688	1	1	10
259	634.25	550.19819	552.20519	316.13987	2.00700	364500	1	1	4
260	634.62	540.13524	542.14216	306.07692	2.00692	52522	1	1	4

#	RT (s)	mz_light	mz_heavy	mz	distance	int_light	nCharge	nTag	Possible hits
261	637.92	409.14260	411.14805	175.08428	2.00545	14850	1	1	41
262	638.36	820.31584	822.32288	586.25752	2.00704	12800	1	1	8
263	639.06	427.13570	429.14172	193.07738	2.00602	23281	1	1	7
264	639.25	376.13238	378.13899	142.07406	2.00661	50213	1	1	13
265	643.67	355.09417	357.09975	242.07170	2.00558	153213	2	2	7
266	648.89	578.21120	580.21766	344.15288	2.00646	24962	1	1	1
267	650.67	370.09800	372.10423	136.03968	2.00623	7773750	1	1	10
268	650.67	603.14825	605.15460	369.08993	2.00635	82197	1	1	7
269	651.38	428.13809	430.14467	194.07977	2.00658	6630	1	1	33
270	656.17	805.32125	807.32686	571.26293	2.00561	16900	1	1	4
271	657.15	563.21558	565.22122	329.15726	2.00565	14823	1	1	3
272	659.53	349.12263	351.12898	115.06431	2.00635	11411250	1	1	33
273	661.17	477.16393	479.16971	243.10561	2.00578	2880000	1	1	1
274	661.65	629.21084	633.22356	162.10150	4.01272	46294	1	2	15
275	662.03	496.13527	498.14096	524.15389	2.00570	8080	2	2	1
276	666.62	515.13002	519.14308	48.02068	4.01305	33563	1	2	18
277	668.38	513.11465	517.13032	46.00531	4.01567	12400	1	2	20
278	671.02	362.10080	364.10711	256.08497	2.00631	7163	2	2	3
279	672.40	599.22560	601.23295	365.16728	2.00736	87600	1	1	19
280	674.19	550.19817	552.20432	316.13985	2.00614	55000	1	1	4
281	676.19	649.25401	651.25491	415.19569	2.00090	11700	1	1	5
282	677.13	570.21298	572.22247	336.15466	2.00949	11800	1	1	8
283	678.30	515.13076	519.14331	48.02142	4.01255	36200	1	2	18
284	678.75	678.25991	680.26688	444.20159	2.00697	43325	1	1	1

#	RT (s)	mz_light	mz_heavy	mz	distance	int_light	nCharge	nTag	Possible hits
285	680.35	420.15876	422.16564	186.10044	2.00688	63300	1	1	15
286	680.64	380.16324	382.16945	146.10492	2.00621	23225	1	1	23
287	681.13	447.10422	449.10983	426.09179	2.00562	97300	2	2	9
288	681.48	408.15890	410.16512	174.10058	2.00622	147438	1	1	35
289	682.61	695.34016	697.34159	461.28184	2.00143	7380	1	1	10
290	682.80	622.22753	624.23503	388.16921	2.00751	24450	1	1	1
291	685.20	335.14252	337.14806	101.08420	2.00554	79800	1	1	20
292	685.64	376.13304	378.13938	142.07472	2.00634	181500	1	1	13
293	687.98	805.32424	807.33089	571.26592	2.00665	115225	1	1	3
294	688.41	486.16893	488.17453	252.11061	2.00560	13500	1	1	9
295	691.52	351.13804	353.14453	117.07972	2.00649	4883750	1	1	38
296	693.11	578.21074	580.21793	344.15242	2.00719	15550	1	1	1
297	694.42	792.31510	794.32160	558.25678	2.00650	38200	1	1	12
298	696.70	337.12183	339.12738	103.06351	2.00555	71066	1	1	45
299	697.36	706.27382	708.27929	472.21550	2.00547	31391	1	1	2
300	698.11	605.15542	607.16323	371.09710	2.00781	7215	1	1	9
301	698.99	601.14498	603.15104	367.08666	2.00605	16688	1	1	8
302	701.87	654.25767	656.26397	420.19935	2.00630	22788	1	1	2
303	701.98	383.11071	385.11669	149.05239	2.00598	3057500	1	1	9
304	703.45	578.22115	580.22578	344.16283	2.00463	11800	1	1	9
305	703.73	346.08617	348.09312	112.02785	2.00696	589969	1	1	10
306	704.92	691.26804	693.27476	457.20972	2.00672	8290	1	1	11
307	705.57	351.13814	353.14451	117.07982	2.00637	5506875	1	1	38
308	709.16	657.24312	659.25336	423.18480	2.01024	9410	1	1	6

#	RT (s)	mz_light	mz_heavy	mz	distance	int_light	nCharge	nTag	Possible hits
309	711.08	447.10361	449.11050	213.04529	2.00689	39900	1	1	4
310	713.17	599.22892	601.23610	365.17060	2.00718	19250	1	1	19
311	713.83	321.09108	323.09733	87.03276	2.00625	99513	1	1	18
312	721.17	614.23327	616.23907	380.17495	2.00580	9450	1	1	9
313	722.45	660.14851	662.15682	426.09019	2.00831	6610	1	1	8
314	724.12	335.14244	337.14875	101.08412	2.00632	49275	1	1	20
315	726.66	367.07878	369.08469	133.02046	2.00591	185845	1	1	4
316	727.18	447.10405	449.11097	426.09146	2.00692	877000	2	2	9
317	727.46	783.17843	785.18340	549.12011	2.00498	6100	1	1	2
318	727.51	578.21502	580.21962	344.15670	2.00460	13280	1	1	2
319	728.14	377.10763	379.11261	286.09863	2.00498	6090	2	2	12
320	730.10	457.16462	459.17205	223.10630	2.00743	30800	1	1	20
321	730.32	641.25014	643.25686	407.19182	2.00671	46400	1	1	4
322	734.50	409.14398	411.15035	175.08566	2.00638	834750	1	1	41
323	734.70	527.19868	529.20534	293.14036	2.00666	91413	1	1	10
324	736.07	508.18198	510.18851	274.12366	2.00652	139066	1	1	1
325	736.41	335.14219	337.14889	101.08387	2.00669	50306	1	1	20
326	736.76	585.21978	587.22641	351.16146	2.00663	507248	1	1	3
327	737.72	422.17337	424.18035	188.11505	2.00698	27056	1	1	23
328	738.87	641.25814	643.26461	407.19982	2.00647	11100	1	1	1
329	739.11	469.15806	471.16534	470.19948	2.00728	9060	2	2	5
330	739.75	367.07858	369.08466	133.02026	2.00608	194884	1	1	4
331	740.65	479.17446	481.18111	245.11614	2.00665	9580	1	1	7
332	740.86	395.12823	397.13450	161.06991	2.00627	423375	1	1	48

#	RT (s)	mz_light	mz_heavy	mz	distance	int_light	nCharge	nTag	Possible hits
333	741.29	447.10423	449.11084	426.09182	2.00661	337000	2	2	9
334	742.78	676.27248	678.27837	442.21416	2.00590	96675	1	1	3
335	742.80	448.10391	450.11023	428.09119	2.00631	76550	2	2	2
336	747.39	438.14918	440.15526	204.09086	2.00608	369203	1	1	11
337	747.69	415.11075	417.11678	362.10486	2.00603	9695	2	2	3
338	747.78	396.13705	398.14364	324.15746	2.00659	3176922	2	2	9
339	748.20	397.13817	399.14448	326.15970	2.00630	713000	2	2	2
340	748.48	629.21024	633.22259	162.10090	4.01235	28744	1	2	15
341	750.37	614.23032	616.23736	380.17200	2.00704	29297	1	1	12
342	750.43	376.13226	378.13863	142.07394	2.00637	26238	1	1	13
343	754.58	508.18119	510.18854	274.12287	2.00735	16000	1	1	2
344	755.02	812.33035	814.33889	578.27203	2.00854	8675	1	1	1
345	755.28	400.08638	402.09188	166.02806	2.00550	44934	1	1	18
346	756.50	362.10149	364.10718	256.08635	2.00569	7800	2	2	11
347	757.00	547.14276	549.14991	313.08444	2.00715	11715	1	1	11
348	762.09	335.14241	337.14891	101.08409	2.00651	70341	1	1	20
349	762.22	624.25196	626.25838	390.19364	2.00643	12300	1	1	4
350	763.81	456.15706	458.16104	222.09874	2.00399	12300	1	1	23
351	764.25	464.17393	466.17955	230.11561	2.00562	12400	1	1	9
352	765.75	381.14841	383.15460	147.09009	2.00618	207281	1	1	39
353	766.89	319.11190	321.11744	85.05358	2.00553	112100	1	1	16
354	768.19	477.18137	479.18770	243.12305	2.00634	54691	1	1	8
355	768.40	526.16437	528.17133	292.10605	2.00695	20563	1	1	14
356	772.95	452.18433	454.19186	218.12601	2.00752	12081	1	1	6

#	RT (s)	mz_light	mz_heavy	mz	distance	int_light	nCharge	nTag	Possible hits
357	776.08	527.19909	529.20616	293.14077	2.00707	13200	1	1	5
358	776.15	454.19720	456.20543	220.13888	2.00823	10549	1	1	1
359	778.61	409.14393	411.15026	175.08561	2.00633	5070000	1	1	41
360	781.51	860.38274	862.39057	626.32442	2.00783	6760	1	1	7
361	781.90	335.14242	337.14930	101.08410	2.00688	99600	1	1	20
362	782.16	477.18115	479.18681	243.12283	2.00566	11300	1	1	8
363	784.30	469.18384	471.18967	470.25104	2.00582	5370	2	2	8
364	784.41	321.11600	323.12256	174.11536	2.00656	726929	2	2	5
365	784.70	426.12069	428.12700	192.06237	2.00631	30900	1	1	36
366	785.16	393.13701	395.14349	318.15739	2.00648	287482	2	2	3
367	788.47	452.18432	454.19119	218.12600	2.00687	31444	1	1	6
368	790.21	458.07414	460.07965	224.01582	2.00552	8630	1	1	3
369	792.25	680.06674	682.07652	446.00842	2.00978	23000	1	1	3
370	792.72	448.19043	450.19675	214.13211	2.00632	14950	1	1	7
371	793.25	351.10226	353.11063	117.04394	2.00837	17700	1	1	1
372	795.39	392.12912	394.13686	316.14159	2.00774	39000	2	2	3
373	796.29	592.22619	594.23353	358.16787	2.00734	17900	1	1	1
374	797.27	383.12959	385.13434	149.07127	2.00475	26300	1	1	7
375	798.45	458.17665	460.18294	224.11833	2.00630	23025	1	1	10
376	799.33	335.14272	337.14927	101.08440	2.00655	11100	1	1	20
377	802.46	360.10231	362.11009	126.04399	2.00778	1398938	1	1	14
378	802.61	311.08412	313.09081	154.05160	2.00669	625969	2	2	2
379	805.99	371.63317	373.64010	275.14970	2.00693	24234	2	2	13
380	807.82	399.13874	401.14507	165.08042	2.00633	6773984	1	1	48

#	RT (s)	mz_light	mz_heavy	mz	distance	int_light	nCharge	nTag	Possible hits
381	810.68	592.22670	594.23280	358.16838	2.00611	18078	1	1	2
382	819.61	453.13465	455.13834	219.07633	2.00369	13650	1	1	41
383	821.19	365.15396	367.16031	131.09564	2.00635	7231250	1	1	36
384	827.89	815.30511	817.31591	581.24679	2.01080	14550	1	1	19
385	828.22	731.28506	733.29231	497.22674	2.00726	14700	1	1	1
386	828.28	727.25793	731.27375	260.14859	4.01582	12700	1	2	1
387	829.56	729.27325	733.28755	262.16391	4.01430	16200	1	2	2
388	829.88	377.11666	379.12301	143.05834	2.00635	101000	1	1	25
389	837.18	365.15381	367.16020	131.09549	2.00640	11103628	1	1	36
390	843.36	392.14721	394.15303	316.17778	2.00582	12500	2	2	3
391	843.55	423.15840	425.16419	189.10008	2.00580	15175	1	1	28
392	846.22	381.09418	383.10011	147.03586	2.00593	173772	1	1	4
393	849.58	681.31077	683.31661	447.25245	2.00583	13700	1	1	7
394	861.13	307.11155	309.11857	73.05323	2.00702	486000	1	1	18
395	862.24	377.10525	379.11226	286.09386	2.00701	34981	2	2	9
396	862.62	397.12573	399.13122	163.06741	2.00550	15900	1	1	5
397	862.65	403.14363	405.15039	338.17063	2.00676	45588	2	2	7
398	862.94	612.26765	616.28052	145.15831	4.01287	55600	1	2	3
399	864.42	539.19087	541.19735	305.13255	2.00648	28775	1	1	7
400	866.68	335.10656	337.11286	101.04824	2.00630	172125	1	1	29
401	867.32	597.11651	599.12135	363.05819	2.00484	37600	1	1	24
402	867.40	626.28276	630.29547	159.17342	4.01272	6790	1	2	2
403	867.75	322.07429	324.08036	88.01597	2.00607	19650	1	1	30
404	870.47	442.16381	444.17074	208.10549	2.00692	19975	1	1	3

#	RT (s)	mz_light	mz_heavy	mz	distance	int_light	nCharge	nTag	Possible hits
405	871.61	300.08643	302.09315	132.05623	2.00671	9500	2	2	8
406	872.61	309.58359	311.58829	151.05055	2.00469	13000	2	2	11
407	873.34	549.16267	551.16896	315.10435	2.00628	10920	1	1	21
408	873.43	344.14207	346.14833	110.08375	2.00627	31025	1	1	3
409	875.13	404.19996	406.20562	170.14164	2.00566	38622	1	1	4
410	876.59	320.65375	322.66024	173.19086	2.00649	39325	2	2	3
411	876.64	531.15325	533.16055	297.09493	2.00730	10400	1	1	8
412	876.80	373.62652	375.63122	279.13640	2.00471	16400	2	2	1
413	881.41	531.16020	533.16827	297.10188	2.00807	146000	1	1	3
414	882.64	532.20464	534.21182	298.14632	2.00718	14425	1	1	2
415	883.24	555.15155	557.15731	321.09323	2.00576	231500	1	1	1
416	883.89	641.33796	643.34475	407.27964	2.00679	389486	1	1	4
417	886.90	311.14183	313.14841	77.08351	2.00658	32300	1	1	6
418	887.71	577.15819	579.16426	343.09987	2.00606	28050	1	1	15
419	888.22	416.11709	418.12230	182.05877	2.00521	12350	1	1	45
420	892.63	612.26689	616.27972	145.15755	4.01283	15275	1	2	3
421	892.89	629.21068	633.22377	162.10134	4.01309	43625	1	2	15
422	893.52	547.15553	549.16331	313.09721	2.00778	113909	1	1	1
423	893.58	462.20597	464.21145	228.14765	2.00548	21413	1	1	3
424	894.27	575.15253	577.15904	341.09421	2.00651	157531	1	1	17
425	898.52	307.09538	309.10136	146.07412	2.00598	37775	2	2	10
426	898.76	296.09557	298.10130	62.03725	2.00573	36038	1	1	19
427	901.44	355.07114	357.07674	242.02564	2.00560	25100	2	2	1
428	901.65	377.10569	379.11283	286.09474	2.00714	14888	2	2	3

#	RT (s)	mz_light	mz_heavy	mz	distance	int_light	nCharge	nTag	Possible hits
429	902.66	577.16032	579.16296	343.10200	2.00264	22900	1	1	16
430	902.75	354.07128	356.07781	240.02593	2.00653	182000	2	2	3
431	903.66	547.15317	549.16149	313.09485	2.00832	25900	1	1	7
432	905.14	553.14761	555.15005	319.08929	2.00244	36900	1	1	8
433	905.49	387.10050	389.10642	153.04218	2.00592	48838	1	1	20
434	905.67	630.22134	632.22736	396.16302	2.00602	7580	1	1	16
435	905.90	408.19522	410.20151	174.13690	2.00629	32319	1	1	9
436	905.98	363.13816	365.14459	129.07984	2.00643	3792500	1	1	30
437	909.12	385.12002	387.12659	151.06170	2.00657	239100	1	1	1
438	911.64	612.26781	616.28023	145.15847	4.01241	50188	1	2	3
439	911.76	549.16349	551.16956	315.10517	2.00607	36300	1	1	25
440	913.07	375.60561	377.61117	283.09459	2.00556	145594	2	2	5
441	913.71	311.08429	313.09097	154.05195	2.00668	564250	2	2	2
442	914.91	368.11636	370.12331	268.11608	2.00695	29650	2	2	11
443	915.53	419.10733	421.11317	185.04901	2.00583	19150	1	1	1
444	918.93	553.14887	555.15162	319.09055	2.00274	15400	1	1	8
445	920.46	547.15576	549.16231	313.09744	2.00655	51000	1	1	1
446	922.46	377.10491	379.11250	286.09318	2.00759	28931	2	2	9
447	922.66	581.14068	585.15624	114.03134	4.01556	6650	1	2	28
448	927.44	502.09665	504.10388	268.03833	2.00723	19863	1	1	8
449	928.09	496.18981	498.19654	262.13149	2.00673	18156	1	1	6
450	929.06	393.14769	395.15427	159.08937	2.00658	33403	1	1	42
451	931.87	335.62226	337.62879	203.12789	2.00653	24719	2	2	11
452	931.97	431.12581	433.13110	197.06749	2.00529	20250	1	1	39

#	RT (s)	mz_light	mz_heavy	mz	distance	int_light	nCharge	nTag	Possible hits
453	932.57	349.61986	351.62644	231.12307	2.00659	39338	2	2	5
454	938.40	411.10457	413.11191	177.04625	2.00734	8470	1	1	16
455	938.70	330.12701	332.13301	96.06869	2.00601	38466	1	1	7
456	938.75	369.10803	371.11399	270.09942	2.00596	15325	2	2	2
457	939.31	531.15215	533.15871	297.09383	2.00656	28613	1	1	5
458	940.82	502.09687	504.10423	268.03855	2.00736	17363	1	1	8
459	946.26	588.11535	590.12096	354.05703	2.00561	22850	1	1	4
460	946.41	376.11681	378.12213	284.11699	2.00532	5010	2	2	2
461	948.03	612.26605	616.27923	145.15671	4.01318	11100	1	2	3
462	949.05	354.10245	356.10966	240.08827	2.00720	5845	2	2	5
463	949.68	396.19421	398.20137	162.13589	2.00716	14300	1	1	5
464	949.91	351.13795	353.14454	117.07963	2.00659	612125	1	1	38
465	951.93	473.13769	475.14418	239.07937	2.00649	62000	1	1	17
466	952.27	395.10982	397.11534	161.05150	2.00552	177273	1	1	4
467	952.50	373.62452	375.63130	279.13240	2.00677	17275	2	2	15
468	952.81	531.15248	533.15894	297.09416	2.00647	19200	1	1	5
469	954.46	975.42724	977.43386	741.36892	2.00663	66403	1	1	1
470	954.70	410.21035	412.21610	176.15203	2.00575	16000	1	1	3
471	957.72	350.64614	352.65257	233.17564	2.00643	42431	2	2	2
472	961.60	336.09837	338.10343	204.08010	2.00506	12000	2	2	2
473	961.72	384.14912	386.15526	300.18161	2.00614	26781	2	2	1
474	962.70	547.15402	549.16222	313.09570	2.00820	16863	1	1	5
475	963.36	406.16400	408.17079	344.21136	2.00679	10100	2	2	1
476	964.43	652.27057	654.27866	418.21225	2.00809	85325	1	1	6

#	RT (s)	mz_light	mz_heavy	mz	distance	int_light	nCharge	nTag	Possible hits
477	965.82	549.16203	551.16848	315.10371	2.00645	12750	1	1	22
478	965.91	638.28272	640.28315	404.22440	2.00042	14600	1	1	3
479	966.44	423.16065	425.16662	189.10233	2.00597	941125	1	1	2
480	966.80	468.21629	470.22236	234.15797	2.00607	17025	1	1	3
481	966.82	501.11493	505.12830	34.00559	4.01336	60559	1	2	8
482	966.88	368.09964	370.10638	268.08265	2.00673	61681	2	2	19
483	967.62	288.57292	290.57910	109.02919	2.00618	8278	2	2	1
484	967.71	369.10361	371.11033	270.09058	2.00672	16900	2	2	9
485	967.73	658.32700	660.32767	424.26868	2.00067	8040	1	1	13
486	967.86	413.15322	415.16196	179.09490	2.00874	26650	1	1	27
487	969.36	405.13650	407.14416	342.15636	2.00766	6730	2	2	10
488	970.53	439.13607	441.14411	205.07775	2.00804	19925	1	1	9
489	971.73	311.59287	313.59969	155.06910	2.00682	77994	2	2	14
490	972.10	652.28883	654.29284	418.23051	2.00402	9550	1	1	2
491	972.44	680.28829	682.29486	446.22997	2.00657	50625	1	1	6
492	975.74	371.63248	373.63850	275.14832	2.00602	10238	2	2	10
493	976.01	547.15392	549.16252	313.09560	2.00860	13400	1	1	5
494	976.26	654.31392	658.32739	187.20458	4.01347	15910	1	2	3
495	977.06	342.63030	344.63687	217.14397	2.00657	14375	2	2	6
496	978.92	656.31406	658.32610	422.25574	2.01205	11700	1	1	5
497	980.38	346.09891	348.10579	224.08118	2.00688	10500	2	2	36
498	980.97	300.10391	302.11049	132.09119	2.00657	40300	2	2	17
499	984.42	421.15291	423.16061	187.09459	2.00770	14750	1	1	7
500	986.49	659.25008	661.25609	425.19176	2.00602	25250	1	1	11

#	RT (s)	mz_light	mz_heavy	mz	distance	int_light	nCharge	nTag	Possible hits
501	987.45	494.68875	496.69480	521.26086	2.00605	6890	2	2	8
502	990.39	395.12300	397.13138	322.12937	2.00837	7260	2	2	8
503	993.03	553.14345	555.14980	319.08513	2.00635	12250	1	1	4
504	994.46	297.57796	299.58451	127.03929	2.00654	27500	2	2	6
505	995.99	322.11739	324.12401	176.11815	2.00661	10248	2	2	1
506	1001.48	488.17759	490.18306	254.11927	2.00548	33175	1	1	5
507	1004.47	445.15332	447.16018	211.09500	2.00686	19300	1	1	29
508	1004.84	407.16383	409.17006	173.10551	2.00623	44875	1	1	29
509	1005.29	379.16895	381.17392	145.11063	2.00498	26941	1	1	19
510	1006.47	363.61797	365.62474	259.11930	2.00677	8575	2	2	12
511	1006.92	372.09032	374.09661	138.03200	2.00629	106109	1	1	19
512	1014.64	441.14752	443.15374	207.08920	2.00623	35781	1	1	25
513	1016.48	427.13226	429.13809	193.07394	2.00583	18200	1	1	24
514	1019.82	312.08485	314.09115	156.05306	2.00630	839000	2	2	17
515	1020.29	311.08446	313.09110	154.05228	2.00663	6781875	2	2	1
516	1022.50	308.11324	310.11967	148.10984	2.00643	904500	2	2	2
517	1026.08	457.14136	459.14810	223.08304	2.00675	15000	1	1	28
518	1028.54	407.16419	409.17041	173.10587	2.00622	146750	1	1	29
519	1030.24	760.26788	762.27474	526.20956	2.00686	84600	1	1	3
520	1034.98	413.15418	415.16000	179.09586	2.00581	573781	1	1	27
521	1037.61	347.11234	349.11957	226.10804	2.00724	13122	2	2	12
522	1038.06	485.17523	487.18131	251.11691	2.00608	8820	1	1	5
523	1038.57	738.22036	740.22077	504.16204	2.00042	12800	1	1	2
524	1039.57	367.60760	369.61494	267.09855	2.00734	318000	2	2	45

#	RT (s)	mz_light	mz_heavy	mz	distance	int_light	nCharge	nTag	Possible hits
525	1041.76	320.63544	322.64184	173.15424	2.00640	22450	2	2	5
526	1042.57	356.09962	358.10434	244.08259	2.00472	7290	2	2	13
527	1043.27	355.62002	357.62708	243.12339	2.00706	10045	2	2	8
528	1045.56	477.21655	479.22447	243.15823	2.00793	19638	1	1	2
529	1047.28	375.60520	377.61174	283.09376	2.00654	88753	2	2	40
530	1048.45	409.12535	411.13071	175.06703	2.00536	65332	1	1	6
531	1048.79	366.10141	368.10676	132.04309	2.00535	34500	1	1	69
532	1056.57	444.15898	446.16564	210.10066	2.00666	13613	1	1	23
533	1058.57	355.63779	357.64365	243.15894	2.00585	23969	2	2	2
534	1059.90	424.22565	426.23126	190.16733	2.00561	10000	1	1	1
535	1060.28	652.29872	656.31119	185.18938	4.01247	50459	1	2	1
536	1061.25	728.24226	730.24889	494.18394	2.00663	198156	1	1	3
537	1061.46	347.14263	349.14677	113.08431	2.00415	7780	1	1	16
538	1063.17	379.16995	381.17633	145.11163	2.00639	2143750	1	1	19
539	1063.71	341.11110	343.11957	214.10556	2.00847	21950	2	2	1
540	1066.14	389.12813	391.13395	155.06981	2.00582	90838	1	1	14
541	1067.29	312.59492	314.60143	157.07320	2.00651	563481	2	2	28
542	1069.45	934.41386	936.42235	700.35554	2.00849	5980	1	1	5
543	1070.30	299.09585	301.10258	130.07506	2.00673	22216	2	2	18
544	1070.56	339.09654	341.10372	210.07643	2.00718	11269	2	2	16
545	1077.06	455.16362	457.16953	221.10530	2.00591	26306	1	1	16
546	1077.31	347.08938	349.09607	226.06212	2.00670	667375	2	2	11
547	1078.28	356.09410	358.10140	244.07156	2.00729	28069	2	2	32
548	1083.48	348.11498	350.11959	228.11331	2.00461	7400	2	2	2

#	RT (s)	mz_light	mz_heavy	mz	distance	int_light	nCharge	nTag	Possible hits
549	1086.58	382.10946	384.11536	296.10227	2.00590	7533	2	2	18
550	1091.08	429.15197	431.15815	195.09365	2.00618	17263	1	1	1
551	1093.29	411.14110	413.14666	177.08278	2.00556	130438	1	1	6
552	1093.63	389.11939	391.12549	310.12215	2.00609	6790	2	2	6
553	1094.23	372.17318	374.18009	138.11486	2.00691	11744	1	1	3
554	1096.58	341.64071	343.64715	215.16477	2.00644	17750	2	2	5
555	1097.31	487.15238	489.15885	253.09406	2.00647	16150	1	1	9
556	1097.57	761.31529	763.32157	527.25697	2.00629	5790	1	1	12
557	1098.63	759.30885	761.31309	525.25053	2.00424	21400	1	1	7
558	1098.67	541.14297	543.14710	307.08465	2.00413	9710	1	1	14
559	1100.11	356.09358	358.10081	244.07053	2.00723	40294	2	2	29
560	1100.38	298.08804	300.09521	128.05945	2.00717	538375	2	2	16
561	1104.59	335.10835	337.11448	202.10007	2.00612	16400	2	2	1
562	1107.66	421.17993	423.18596	187.12161	2.00603	299000	1	1	22
563	1109.55	466.17945	468.18461	232.12113	2.00516	11031	1	1	6
564	1110.73	356.09422	358.10090	244.07180	2.00668	248125	2	2	32
565	1117.69	328.13365	330.14152	188.15066	2.00786	9650	2	2	4
566	1124.66	423.14240	425.14653	189.08408	2.00414	8380	1	1	1
567	1128.03	326.63526	328.64112	185.15387	2.00586	13700	2	2	2
568	1129.61	321.16280	323.16964	87.10448	2.00684	148638	1	1	10
569	1130.88	348.11413	350.12264	228.11163	2.00850	32100	2	2	8
570	1134.33	328.11714	330.12356	188.11764	2.00643	57016	2	2	24
571	1140.53	386.10673	388.11380	152.04841	2.00707	8720	1	1	31
572	1143.63	528.16246	532.17549	61.05312	4.01303	52644	1	2	15

#	RT (s)	mz_light	mz_heavy	mz	distance	int_light	nCharge	nTag	Possible hits
573	1145.62	321.10926	323.11535	174.10188	2.00609	35200	2	2	35
574	1146.24	532.24870	534.25537	298.19038	2.00667	15350	1	1	18
575	1148.74	349.15752	351.16314	115.09920	2.00562	10925	1	1	21
576	1150.70	336.11439	338.12125	204.11214	2.00686	7110	2	2	11
577	1153.10	292.10594	294.11229	116.09524	2.00635	41381	2	2	9
578	1153.63	379.16944	381.17540	145.11112	2.00596	379156	1	1	19
579	1156.45	487.09989	489.10508	253.04157	2.00519	20350	1	1	3
580	1161.51	368.10170	370.10507	268.08675	2.00337	83175	2	2	9
581	1161.60	353.09554	355.10164	119.03722	2.00610	35100	1	1	7
582	1176.41	335.64035	337.64751	203.16406	2.00716	56400	2	2	4
583	1176.71	338.08845	340.09375	104.03013	2.00530	9610	1	1	3
584	1183.58	693.16709	697.18175	226.05775	4.01466	12700	1	2	18
585	1187.80	346.08548	348.09120	112.02716	2.00572	32000	1	1	9
586	1190.73	711.13194	715.14952	244.02260	4.01758	10500	1	2	2
587	1190.80	311.08533	313.09345	154.05402	2.00812	1102188	2	2	1
588	1196.40	427.16945	429.17523	193.11113	2.00578	371219	1	1	13
589	1197.20	472.22522	474.23277	238.16690	2.00755	7365	1	1	1
590	1197.95	343.08621	345.09177	109.02789	2.00556	12800	1	1	3
591	1198.60	359.10529	361.11103	125.04697	2.00575	39700	1	1	15
592	1199.57	335.16715	337.17351	202.21767	2.00635	20450	2	2	1
593	1202.35	327.09071	329.09775	186.06478	2.00705	36100	2	2	16
594	1203.83	397.15846	399.16358	163.10014	2.00512	11875	1	1	16
595	1204.07	355.06430	357.07099	242.01196	2.00668	12953	2	2	7
596	1215.62	403.14390	405.15022	169.08558	2.00633	11850	1	1	11

#	RT (s)	mz_light	mz_heavy	mz	distance	int_light	nCharge	nTag	Possible hits
597	1219.76	355.06379	357.07005	242.01094	2.00626	8680	2	2	7
598	1225.89	325.09802	327.10475	182.07941	2.00673	661500	2	2	35
599	1226.60	324.59677	326.60342	181.07691	2.00665	5635547	2	2	4
600	1227.31	414.12389	416.13048	180.06557	2.00658	35853	1	1	10
601	1229.02	373.08570	375.09071	139.02738	2.00501	15050	1	1	9
602	1229.78	386.10619	388.11146	152.04787	2.00527	26500	1	1	31
603	1237.79	363.17355	365.17966	129.11523	2.00611	30328	1	1	10
604	1238.67	335.17841	337.18567	101.12009	2.00726	444575	1	1	1
605	1239.81	515.21134	517.21741	562.30605	2.00607	9200	2	2	1
606	1240.64	800.38542	802.39352	566.32710	2.00810	15000	1	1	4
607	1243.70	378.08280	380.08899	144.02448	2.00619	27225	1	1	1
608	1249.23	363.17292	365.17969	129.11460	2.00677	13150	1	1	10
609	1252.74	335.17933	337.18635	101.12101	2.00702	576656	1	1	1
610	1263.07	437.17342	439.18081	203.11510	2.00739	30275	1	1	22
611	1263.47	335.17842	337.18496	101.12010	2.00653	57878	1	1	1
612	1269.01	373.12257	375.12872	278.12850	2.00615	17950	2	2	17
613	1278.93	681.29384	683.29907	447.23552	2.00523	16313	1	1	6
614	1280.03	345.62470	347.63089	223.13277	2.00619	11600	2	2	2
615	1281.84	690.79093	692.79794	913.46522	2.00701	26420	2	2	1
616	1282.81	275.08522	277.09202	82.05380	2.00680	16538	2	2	4
617	1282.90	690.45901	692.46494	456.40069	2.00593	30138	1	1	12
618	1284.23	598.10105	600.10475	364.04273	2.00370	11340	1	1	6
619	1286.04	331.56011	333.56734	195.00358	2.00723	10300	2	2	3
620	1289.10	327.10907	329.11622	186.10150	2.00715	19775	2	2	15

#	RT (s)	mz_light	mz_heavy	mz	distance	int_light	nCharge	nTag	Possible hits
621	1294.28	321.12757	323.13420	174.13850	2.00663	143875	2	2	9
622	1298.78	302.07811	304.08615	136.03958	2.00803	6970	2	2	10
623	1302.79	356.59542	358.60206	245.07421	2.00664	159528	2	2	6
624	1303.75	357.59491	359.60199	247.07317	2.00708	17300	2	2	1
625	1306.20	435.19433	437.20016	201.13601	2.00583	23763	1	1	12
626	1308.32	354.11613	356.12235	120.05781	2.00622	190583	1	1	13
627	1310.36	331.60383	333.61060	195.09102	2.00677	50994	2	2	30
628	1310.46	367.14712	369.15390	133.08880	2.00678	14675	1	1	6
629	1311.31	488.15178	490.15874	254.09346	2.00696	21775	1	1	8
630	1313.29	325.60921	327.61556	183.10179	2.00634	26088	2	2	8
631	1314.07	284.60082	286.60668	101.08500	2.00586	10300	2	2	20
632	1315.72	973.28293	975.28353	739.22461	2.00060	10300	1	1	1
633	1320.01	354.11670	356.12358	120.05838	2.00689	821875	1	1	13
634	1325.81	338.59329	340.60018	209.06993	2.00689	24400	2	2	42
635	1331.07	345.60138	347.60789	223.08612	2.00652	34638	2	2	30
636	1332.36	527.25500	529.26273	293.19668	2.00772	22566	1	1	7
637	1333.22	515.13009	519.14304	48.02075	4.01294	10443	1	2	18
638	1335.09	573.24449	575.25077	339.18617	2.00628	10400	1	1	1
639	1336.96	349.19354	351.19983	115.13522	2.00630	22750	1	1	1
640	1337.59	630.93376	632.93945	793.75088	2.00569	9965	2	2	1
641	1343.16	631.09962	633.10616	794.08261	2.00654	30003	2	2	1
642	1346.90	353.07408	355.08062	238.03152	2.00655	12300	2	2	11
643	1349.36	630.93330	632.93979	793.74996	2.00649	58481	2	2	1
644	1350.47	331.60387	333.61080	195.09110	2.00693	344250	2	2	30

#	RT (s)	mz_light	mz_heavy	mz	distance	int_light	nCharge	nTag	Possible hits
645	1352.47	297.59040	299.59735	127.06417	2.00694	57313	2	2	20
646	1353.41	577.15698	581.16941	110.04764	4.01243	21538	1	2	8
647	1357.84	886.24530	888.25468	1304.37397	2.00938	7980	2	2	2
648	1358.13	352.60937	354.61549	237.10210	2.00612	28288	2	2	11
649	1359.12	994.27031	996.27870	760.21199	2.00839	11500	1	1	1
650	1361.39	588.26278	590.26994	354.20446	2.00716	14938	1	1	10
651	1361.52	287.08075	289.08682	106.04486	2.00607	7935	2	2	1
652	1362.27	308.58151	310.58939	149.04638	2.00788	29494	2	2	17
653	1363.36	313.59304	315.59988	159.06944	2.00684	37797	2	2	10
654	1364.45	305.57144	307.57810	143.02623	2.00666	152469	2	2	2
655	1367.48	577.15696	581.17035	110.04762	4.01339	27553	1	2	8
656	1375.96	344.10595	346.11224	110.04763	2.00629	33750	1	1	8
657	1378.47	328.13459	330.14174	188.15255	2.00715	9450	2	2	4
658	1379.15	504.18334	506.18996	270.12502	2.00662	19950	1	1	11
659	1385.41	302.07785	304.08488	136.03906	2.00703	36913	2	2	10
660	1388.40	772.32794	774.33575	538.26962	2.00781	28100	1	1	7
661	1388.47	515.13085	519.14448	48.02151	4.01363	304875	1	2	18
662	1394.29	847.32132	851.34155	380.21198	4.02023	2096563	1	2	4
663	1394.42	612.26697	616.28060	145.15763	4.01364	55681	1	2	3
664	1398.37	454.24100	456.24746	220.18268	2.00645	222864	1	1	3
665	1400.16	826.36916	828.37345	592.31084	2.00429	7090	1	1	5
666	1405.36	363.21032	365.21693	129.15200	2.00661	383521	1	1	1
667	1408.01	331.08998	333.09706	194.06333	2.00708	10671	2	2	1
668	1412.89	620.24786	622.25051	386.18954	2.00265	24200	1	1	5

#	RT (s)	mz_light	mz_heavy	mz	distance	int_light	nCharge	nTag	Possible hits
669	1414.54	947.22806	949.23212	713.16974	2.00406	11600	1	1	3
670	1415.41	297.08133	299.08729	126.04601	2.00597	34594	2	2	3
671	1416.03	338.61192	340.61882	209.10720	2.00690	359375	2	2	18
672	1418.70	760.13421	762.14043	526.07589	2.00622	23725	1	1	2
673	1418.87	760.33615	762.34165	526.27783	2.00550	14600	1	1	17
674	1419.44	950.41676	952.42586	716.35844	2.00909	16750	1	1	2
675	1420.81	573.47092	575.47781	678.82520	2.00689	57300	2	2	1
676	1422.21	757.34630	759.35453	523.28798	2.00823	6500	1	1	9
677	1422.72	479.20212	481.20688	245.14380	2.00476	8210	1	1	1
678	1422.84	908.41991	910.42194	674.36159	2.00203	6650	1	1	5
679	1422.91	573.21914	575.22804	339.16082	2.00891	42200	1	1	2
680	1425.70	323.60645	325.61317	179.09626	2.00672	12753	2	2	27
681	1427.03	327.12655	329.13383	186.13647	2.00728	28450	2	2	4
682	1427.52	331.08949	333.09666	194.06234	2.00718	19534	2	2	5
683	1436.73	426.17300	428.17975	192.11468	2.00674	83602	1	1	10
684	1437.72	319.57332	321.57954	171.03000	2.00622	42131	2	2	2
685	1439.19	361.07257	363.07864	254.02849	2.00607	36500	2	2	5
686	1442.11	623.22074	625.22677	389.16242	2.00603	61775	1	1	23
687	1443.07	293.59553	295.60257	119.07442	2.00704	20775	2	2	11
688	1446.23	822.36222	824.36827	588.30390	2.00605	55663	1	1	8
689	1449.13	832.23448	834.23320	598.17616	1.99872	25500	1	1	4
690	1449.94	854.21634	856.21590	620.15802	1.99956	18700	1	1	1
691	1461.50	483.26699	485.27431	249.20867	2.00732	35800	1	1	1
692	1465.87	865.19813	867.20610	631.13981	2.00797	7260	1	1	1

#	RT (s)	mz_light	mz_heavy	mz	distance	int_light	nCharge	nTag	Possible hits
693	1469.02	620.23595	622.24172	386.17763	2.00577	30000	1	1	15
694	1469.04	923.42970	925.43666	689.37138	2.00696	40225	1	1	3
695	1472.09	623.22101	625.22737	389.16269	2.00636	109919	1	1	14
696	1474.50	826.36430	828.37156	592.30598	2.00726	7655	1	1	3
697	1476.06	621.15781	625.17156	154.04847	4.01374	21375	1	2	12
698	1483.37	792.31717	794.31760	558.25885	2.00043	8260	1	1	12
699	1484.39	578.23513	580.24152	344.17681	2.00638	53263	1	1	1
700	1486.25	669.32514	673.33790	202.21580	4.01276	15800	1	2	1
701	1486.89	570.29100	572.29316	336.23268	2.00216	11450	1	1	100
702	1492.33	485.21034	487.21635	251.15202	2.00602	47225	1	1	5
703	1493.26	685.32688	687.33352	451.26856	2.00664	11730	1	1	24
704	1497.86	456.21945	458.22670	222.16113	2.00725	52984	1	1	2
705	1500.28	403.06362	405.06748	169.00530	2.00387	41691	1	1	9
706	1506.00	468.21979	470.22597	234.16147	2.00618	18230	1	1	4
707	1506.67	584.30725	586.30957	350.24893	2.00232	9120	1	1	59
708	1506.92	759.35082	761.35516	525.29250	2.00434	15900	1	1	9
709	1511.93	757.34615	759.35216	523.28783	2.00601	36700	1	1	9
710	1513.80	735.34388	737.35017	501.28556	2.00629	94963	1	1	28
711	1516.31	556.27458	558.27698	322.21626	2.00240	9940	1	1	53
712	1520.00	865.19981	867.20924	631.14149	2.00943	14050	1	1	1
713	1521.27	759.35280	761.35771	525.29448	2.00491	24800	1	1	8
714	1523.31	711.34181	713.35179	477.28349	2.00998	7930	1	1	25
715	1525.89	598.32315	600.32440	364.26483	2.00125	9490	1	1	37
716	1527.21	348.11694	350.12275	228.11724	2.00582	27250	2	2	2

#	RT (s)	mz_light	mz_heavy	mz	distance	int_light	nCharge	nTag	Possible hits
717	1529.00	482.23612	484.24262	248.17780	2.00650	239048	1	1	1
718	1531.21	584.18886	586.19478	350.13054	2.00592	65250	1	1	15
719	1531.63	440.22542	442.23200	206.16710	2.00658	289097	1	1	1
720	1532.61	761.35818	763.36785	527.29986	2.00967	13400	1	1	16
721	1543.21	519.28734	521.29393	285.22902	2.00659	23750	1	1	6
722	1549.90	533.33992	535.34661	299.28160	2.00669	57257	1	1	14
723	1553.12	669.35654	671.36305	435.29822	2.00651	6390	1	1	17
724	1566.31	535.35505	537.36188	301.29673	2.00683	21875	1	1	8
725	1568.14	547.35599	549.36172	313.29767	2.00573	14800	1	1	10
726	1569.01	454.23984	456.24643	220.18152	2.00659	60752	1	1	3
727	1569.71	417.12120	419.12817	183.06288	2.00697	60613	1	1	15
728	1569.99	666.36798	668.37369	432.30966	2.00571	13950	1	1	4
729	1574.10	489.31422	491.32056	255.25590	2.00634	104750	1	1	2
730	1580.99	515.32889	517.33456	281.27057	2.00567	7580	1	1	4
731	1582.57	455.13753	457.14468	221.07921	2.00715	512983	1	1	4
732	1602.43	687.34515	689.35050	453.28683	2.00535	6765	1	1	22
733	1619.19	713.35902	715.36405	479.30070	2.00503	21000	1	1	27
734	1620.14	517.34507	519.35134	283.28675	2.00627	73725	1	1	10
735	1631.72	713.35865	715.36492	479.30033	2.00626	19620	1	1	27
736	1634.05	575.35016	577.35687	341.29184	2.00671	32300	1	1	16
737	1689.44	671.34698	673.35294	437.28866	2.00596	7760	1	1	9
738	1707.69	671.34857	673.35182	437.29025	2.00325	7600	1	1	9
739	1707.69	697.36235	699.37029	463.30403	2.00794	5620	1	1	19

Table S2. List of the compounds presented in Figure 4 of these nine metabolic pathways.

KEGG ID	Compound name	KEGG ID	Compound name
C00010	Coenzyme A	C00011	Carbon dioxide
C00012	Peptide	C00014	Ammonia
C00015	Uridine 5'-diphosphate	C00019	S-Adenosylmethionine
C00020	Adenosine monophosphate	C00022	Pyruvic acid
C00024	Acetyl-CoA	C00025	L-Glutamic acid
C00026	Oxoglutaric acid	C00029	Uridine diphosphate glucose
C00033	Acetic acid	C00036	Oxalacetic acid
C00037	Glycine	C00039	DNA
C00041	L-Alanine	C00042	Succinic acid
C00046	RNA	C00048	Glyoxylic acid
C00049	L-Aspartic acid	C00054	Adenosine 3',5'-diphosphate
C00055	Cytidine monophosphate	C00058	Formic acid
C00062	L-Arginine	C00063	Cytidine triphosphate
C00064	L-Glutamine	C00068	Thiamine pyrophosphate
C00075	Uridine triphosphate	C00077	Ornithine
C00078	L-Tryptophan	C00079	L-Phenylalanine
C00082	L-Tyrosine	C00083	Malonyl-CoA
C00086	Urea	C00088	Nitrite
C00094	Sulfite	C00097	L-Cysteine
C00099	Beta-Alanine	C00100	Propionyl-CoA

KEGG ID	Compound name	KEGG ID	Compound name
C00105	Uridine 5'-monophosphate	C00106	Uracil
C00108	2-Aminobenzoic acid	C00112	CDP
C00119	Phosphoribosyl pyrophosphate	C00122	Fumaric acid
C00134	Putrescine	C00135	L-Histidine
C00136	Butanoyl-CoA	C00141	Alpha-ketoisovaleric acid
C00148	L-Proline	C00152	L-Asparagine
C00155	L-Homocysteine	C00164	Acetoacetic acid
C00169	Carbamoylphosphate	C00178	Thymine
C00179	Agmatine	C00183	L-Valine
C00187	Cholesterol	C00192	Hydroxylamine
C00213	Sarcosine	C00214	Thymidine
C00222	Malonic semialdehyde	C00227	Acetylphosphate
C00229	Acyl-carrier protein	C00232	Succinic acid semialdehyde
C00239	dCMP	C00241	Amide
C00244	Nitrate	C00245	Taurine
C00246	Butyric acid	C00295	Orotic acid
C00299	Uridine	C00300	Creatine
C00315	Spermidine	C00327	Citrulline
C00332	Acetoacetyl-CoA	C00334	Gamma-Aminobutyric acid
C00337	4,5-Dihydroorotic acid	C00342	Thioredoxin
C00343	Thioredoxin disulfide	C00352	Glucosamine 6-phosphate
C00356	3-Hydroxy-3-methylglutaryl-CoA	C00363	dTDP
C00364	5-Thymidylic acid	C00365	dUMP

KEGG ID	Compound name	KEGG ID	Compound name
C00380	Cytosine	C00383	Malonic acid
C00386	Carnosine	C00402	D-Aspartic acid
C00429	Dihydrouracil	C00431	5-Aminopentanoic acid
C00433	2,5-Dioxopentanoate	C00436	N-Carbamoylputrescine
C00437	N-Acetylornithine	C00438	Ureidosuccinic acid
C00458	dCTP	C00459	Thymidine 5'-triphosphate
C00460	Deoxyuridine triphosphate	C00475	Cytidine
C00488	Formamide	C00497	(R)-Malate
C00506	Cysteic acid	C00519	Hypotaurine
C00522	(R)-Pantoate	C00526	Deoxyuridine
C00533	Nitric oxide	C00542	Allocystathionine
C00555	4-Aminobutyraldehyde	C00581	Guanidoacetic acid
C00593	Sulfoacetaldehyde	C00606	3-Sulfinoalanine
C00624	N-Acetyl-L-alanine	C00672	Deoxyribose 1-phosphate
C00695	Cholic acid	C00697	Nitrogen
C00705	dCDP	C00706	Amine
C00726	Nitrile	C00741	Diacetyl
C00750	Spermine	C00763	D-Proline
C00791	Creatinine	C00804	Propynoic acid
C00810	(R)-Acetoin	C00813	Barbiturate
C00831	Pantetheine	C00864	Pantothenic acid
C00877	Crotonoyl-CoA	C00881	Deoxycytidine
C00882	Dephospho-CoA	C00884	Homocarnosine

KEGG ID	Compound name	KEGG ID	Compound name
C00887	Nitrous oxide	C00894	Acrylyl-CoA
C00900	2-Acetolactate	C00906	Dihydrothymine
C00940	2-Keto-glutaramic acid	C00966	2-Dehydropantoate
C00986	1,3-Diaminopropane	C00989	4-Hydroxybutyric acid
C01010	Urea-1-carboxylate	C01013	Hydroxypropionic acid
C01035	4-Guanidinobutanoic acid	C01042	N-Acetyl-L-aspartic acid
C01043	N-Carbamoylsarcosine	C01053	(R)-4-Dehydropantoate
C01073	N-Acetyl-beta-alanine	C01088	(R)-3,3-Dimethylmalate
C01089	(R)-3-Hydroxybutyric acid	C01103	Orotidylic acid
C01110	5-Amino-2-oxopentanoic acid	C01134	Pantetheine 4'-phosphate
C01137	S-Adenosylmethioninamine	C01144	(S)-3-Hydroxybutanoyl-CoA
C01157	Hydroxyproline	C01165	L-Glutamic-gamma-semialdehyde
C01168	Pseudouridine 5'-phosphate	C01250	N-Acetyl-L-glutamate 5-semialdehyde
C01262	Anserine	C01301	3a,7a,12a-Trihydroxy-5b-cholestan-26-al
C01346	dUDP	C01353	Carbonic acid
C01358	NH4OH	C01368	3'-UMP
C01384	Maleic acid	C01412	Butanal
C01417	Cyanate	C01563	Carbamic acid
C01678	Cysteamine	C01682	Nopaline
C01769	(S)-Acetoin	C01794	Choloyl-CoA
C01837	Nitroethane	C01877	4-Oxoproline
C01921	Glycocholic acid	C01959	Taurocyamine
C02067	Pseudouridine	C02170	Methylmalonic acid

KEGG ID	Compound name	KEGG ID	Compound name
C02291	L-Cystathionine	C02305	Phosphocreatine
C02331	Vinylacetyl-CoA	C02335	Beta-Alanyl-CoA
C02354	2',3'-Cyclic CMP	C02355	2',3'-Cyclic UMP
C02362	2-Oxosuccinamate	C02376	5-Methylcytosine
C02411	Glutaconyl-1-CoA	C02466	Trimetaphosphate
C02527	Butanoylphosphate	C02528	Chenodeoxycholic acid
C02565	N-Methylhydantoin	C02630	2-Hydroxyglutarate
C02642	Ureidopropionic acid	C02647	4-Guanidinobutanal
C02714	N-Acetylputrescine	C02946	4-Acetamidobutanoic acid
C03044	(R,R)-Butane-2,3-diol	C03046	(S,S)-Butane-2,3-diol
C03058	2-Hydroxyglutaryl-CoA	C03078	4-Guanidinobutanamide
C03090	5-Phosphoribosylamine	C03149	N-Phosphotaurocyamine
C03166	Phosphoguanidinoacetate	C03287	L-Glutamic acid 5-phosphate
C03296	N2-Succinyl-L-arginine	C03406	Argininosuccinic acid
C03415	N2-Succinyl-L-ornithine	C03440	cis-4-Hydroxy-D-proline
C03492	D-4'-Phosphopantothenate	C03561	3-Hydroxybutyryl-CoA
C03564	1-Pyrroline-2-carboxylic acid	C03594	7a-Hydroxycholesterol
C03618	L-threo-3-Methylaspartate	C03688	Apo-[acyl-carrier-protein]
C03771	5-Guanidino-2-oxopentanoate	C03794	Adenylsuccinic acid
C03912	(S)-1-Pyrroline-5-carboxylate	C03997	5-Hydroxymethyldeoxycytidylate
C04039	2,3-Dihydroxy-3-methylbutanoate	C04079	D-Pantothenoyl-L-cysteine
C04133	N-Acetyl-L-glutamyl 5-phosphate	C04137	N2-(D-1-Carboxyethyl)-L-arginine
C04281	Pyrroline hydroxycarboxylic acid	C04282	1-Pyrroline-4-hydroxy-2-carboxylate

KEGG ID	Compound name	KEGG ID	Compound name
C04352	4-Phosphopantothenoylcysteine	C04546	(R)-3-((R)-3-Hydroxybutanoyloxy)butanoate
C04554	3alpha,7alpha-Dihydroxy-5beta-cholestanate	C04722	3a,7a,12a-Trihydroxy-5b-cholestanoic acid
C05100	Ureidoisobutyric acid	C05122	Taurocholic acid
C05123	2-Hydroxyethanesulfonate	C05125	2-(a-Hydroxyethyl)thiamine diphosphate
C05145	3-Aminoisobutanoic acid	C05167	alpha-Amino acid
C05281	5-Methylbarbiturate	C05337	Chenodeoxycholoyl-CoA
C05340	beta-Alanyl-L-arginine	C05341	beta-Alanyl-L-lysine
C05444	3alpha,7alpha,26-Trihydroxy-5beta-cholestane	C05445	3a,7a-Dihydroxy-5b-cholestan-26-al
C05446	3alpha,7alpha,12alpha,26-Tetrahydroxy-5beta-cholestane	C05447	3a,7a-Dihydroxy-5b-cholest-24-enoyl-CoA
C05448	3a,7a,12a-Trihydroxy-5b-cholestanoyl-CoA	C05449	3a,7a-Dihydroxy-5b-24-oxocholestanoyl-CoA
C05450	3a,7a,12a,24-Tetrahydroxy-5b-cholestanoyl-CoA	C05451	7a-Hydroxy-5b-cholestan-3-one
C05452	3a,7a-Dihydroxy-5b-cholestane	C05453	7a,12a-Dihydroxy-5b-cholestan-3-one
C05454	5-b-Cholestane-3a ,7a ,12a-triol	C05455	7a-Hydroxy-cholestene-3-one
C05460	3a,7a,12a-Trihydroxy-5b-cholest-24-enoyl-CoA	C05465	Taurochenodesoxycholic acid
C05466	Chenodeoxycholic acid glycine conjugate	C05467	3a,7a,12a-Trihydroxy-5b-24-oxocholestanoyl-CoA
C05468	5b-Cyprinol sulfate	C05665	3-Aminopropionaldehyde
C05668	3-Hydroxypropionyl-CoA	C05822	3'-CMP
C05844	5-L-Glutamyl-taurine	C05931	N-Succinyl-L-glutamate
C05932	N2-Succinyl-L-glutamic acid 5-semialdehyde	C05933	N-(o)-Hydroxyarginine
C05936	N4-Acetylaminobutanal	C05938	L-4-Hydroxyglutamate semialdehyde
C05939	Linatine	C05941	2-Oxo-4-hydroxy-5-aminovalerate
C05942	Pyrrole-2-carboxylic	C05944	Pantothenol

KEGG ID	Compound name	KEGG ID	Compound name
C05945	L-Arginine phosphate	C05946	D-4-Hydroxy-2-oxoglutarate
C05947	L-erythro-4-Hydroxyglutamate	C06059	Cyclic amidines
C06060	Amidines	C06142	1-Butanol
C06143	Poly-beta-hydroxybutyrate	C06144	3-Butynoate
C06145	3-Butyn-1-al	C06146	3-Butyn-1-ol
C06198	P1,P4-Bis(5'-uridyl) tetraphosphate	C06341	7-a,27-dihydroxycholesterol
C06735	Aminoacetaldehyde	C11038	2'-Deoxy-5-hydroxymethylcytidine-5'-diphosphate
C11039	2'-Deoxy-5-hydroxymethylcytidine-5'-triphosphate	C13550	24-Hydroxycholesterol
C14179	Sulfoacetate	C15518	(24S)-Cholest-5-ene-3beta,7alpha,24-triol
C15519	25-Hydroxycholesterol	C15520	7-a,25-Dihydroxycholesterol
C15607	3-Oxo-3-ureidopropanoate	C15610	Cholest-5-ene-3beta,26-diol
C15613	(25R)-3alpha,7alpha,12alpha-Trihydroxy-5beta-cholestan-26-oyl-CoA	C15699	Gamma-glutamyl-L-putrescine
C15700	gamma-Glutamyl-gamma-aminobutyraldehyde	C15767	4-(Glutamylamino) butanoate
C17331	7alpha,24-Dihydroxy-4-cholesten-3-one	C17332	alpha,25-Dihydroxy-4-cholesten-3-one
C17333	3 beta-Hydroxy-5-cholestenoate	C17335	3 beta,7 alpha-Dihydroxy-5-cholestenoate
C17336	7 alpha,26-Dihydroxy-4-cholesten-3-one	C17337	7 alpha-Hydroxy-3-oxo-4-cholestenoate
C17339	4-Cholesten-7alpha,12alpha-diol-3-one	C17343	(25S)-3alpha,7alpha,12alpha-Trihydroxy-5beta-cholestan-26-oyl-CoA
C17345	(25R)-3alpha,7alpha-Dihydroxy-5beta-cholestanoyl-CoA	C17346	(25S)-3alpha,7alpha-Dihydroxy-5beta-cholestanoyl-CoA
C18091	Ethylnitronate		
